# Global burden of diarrhea disease in the older adult and its attributable risk factors from 1990 to 2021: a comprehensive analysis from the global burden of disease study 2021

**DOI:** 10.3389/fpubh.2025.1541492

**Published:** 2025-04-04

**Authors:** Wen-Zhuo Zhao, Jing-Yi Wang, Min-Na Zhang, Shang-Nong Wu, Wei-Jie Dai, Xiao-Zhong Yang, Hong-Gang Wang

**Affiliations:** Department of Gastroenterology, The Affiliated Huaian No. 1 People's Hospital of Nanjing Medical University, Huai'an, Jiangsu, China

**Keywords:** diarrhea disease, epidemiology, EAPC, global burden of disease, older adult

## Abstract

**Introduction:**

Diarrhea disease among the older adult is an underappreciated global health issue despite its substantial burden. This study provides a comprehensive analysis of the epidemiological trends of diarrhea in individuals over 65 years, examining incidence, prevalence, mortality, and disability-adjusted life-years (DALYs) from 1990 to 2021.

**Methods:**

Utilizing data from the Global Burden of Diseases (GBD) 2021, this cross-sectional study assesses the older adult population across 204 countries and territories. The analysis includes metrics such as incidence, prevalence, mortality, DALYs, and estimated annual percentage changes (EAPCs), stratified by region, country, age, sex, and Sociodemographic Index (SDI).

**Results:**

A nearly 200% increase in incidence and prevalence was observed worldwide, with the highest rise in those over 95 years. Mortality and DALYs have declined, especially in the 65–69 age group. High SDI regions showed the largest increase in incidence rates and are the only areas with increasing mortality and DALYs trends. Unsafe water sources emerged as the primary risk factor for diarrhea-related deaths among the older adult.

**Discussion:**

The burden of diarrhea in the older adult has significantly increased, particularly in high-income regions, warranting targeted interventions. The positive correlation between EAPC and the Human Development Index underscores the need for improved water safety to mitigate the disease burden. This study's findings are crucial for shaping public health strategies and informing policy decisions regarding the older adult population.

## 1 Introduction

Diarrhea disease remains a significant challenge in global health. The latest global burden of diseases (GBD) 2021 report indicates that since 2010, it has been among the top causes of illness across all genders globally. In 2021, with 467 million new cases (95% UI, 411–522), it was the second most common cause of illness globally, surpassed only by upper respiratory infections (1). Fortunately, over the past 30 years, significant progress has been made globally in reducing the disease burden of Diarrhea disease. It has dropped from being the fifth leading cause of death worldwide in 1990 to the fourteenth leading cause in 2021 (2). The global health community has extensively focused on Diarrhea disease in children, leading to numerous studies and the creation of effective interventions. These initiatives include enhancements in sanitation and hygiene, wider availability of potable water, vaccination campaigns, and the advocacy of proper nutrition and medical care. Such comprehensive efforts have yielded substantial improvements in reducing the impact of Diarrhea diseases on children across the globe (3–10). However, the problem of Diarrhea disease in the older adult is often neglected and lacks adequate focus.

According to existing evidence, about 75% of diarrhea-related deaths in 2016 occurred in individuals over the age of 5, particularly in the older adult population aged 70 and above, where the disease burden caused by Diarrhea disease is particularly significant (11). Given the accelerating trend of global population aging, the number of people aged 70 and above has increased significantly by 50% from 1990 to 2016 (11). In-depth research on Diarrhea disease trends in the older adult is crucial for public health and healthcare sectors. Currently, no comprehensive report exists on the long-term global epidemiology of this disease in the older adult. This study leverages the GBD database for an extensive analysis of Diarrhea disease incidence, prevalence, mortality, and DALYs among the older adult from 1990 to 2021, including a discussion of risk factors. We aim to spark innovation in prevention and treatment strategies, reducing the health risks posed by Diarrhea disease to the older adult, through this detailed examination of GBD 2021 data.

## 2 Methods

### 2.1 Overview and data collection

We have collected data on older adult patients with Diarrhea disease aged 65 and above worldwide through the Global Health Data Exchange query tool developed by the GBD collaborators (1, 2). The 2021 GBD study assessed the incidence, prevalence, disability-adjusted life years (DALYs) for 371 diseases and injuries, and mortality rates for 288 diseases and injuries in 204 countries and territories and 811 sub-national regions from 1990 to 2021, along with the corresponding rates and uncertainty intervals (1, 2). To encapsulate the older adult-specific Diarrhea disease burden, our study segmented the older adult population into seven age brackets: 65–69, 70–74, 75–79, 80–84, 85–89, 90–94 years old, and 95 years and above. We amassed data on incidence, prevalence, mortality, and DALYs related to Diarrhea disease at global, regional, and national scales. The GBD database omits race and ethnicity, as these are not collected in its framework. We used linear regression to determine the estimated annual percentage change (EAPC) for these metrics (12). We also collected data on the global risk factors contributing to deaths from Diarrhea disease in the older adult (11). This study followed the Strengthening the Reporting of Observational Studies in Epidemiology (STROBE) reporting guidelines.

### 2.2 Sociodemographic index

The sociodemographic index (SDI) is an indicator used to gauge the level of development of a country or region, taking into account data such as fertility rates, education levels, and per capita income (13–15). SDI ranges from 0 to 1; higher levels indicate greater socioeconomic development (15). It has been reported that the SDI is associated with disease incidence and mortality rates. In this study, countries and regions were categorized into five SDI quintiles (low, low-middle, middle, high-middle, and high) to explore the relationship between the burden of Diarrhea disease in the older adult and socio-economic development.

### 2.3 Risk factors

In the GBD 2021 study, lack of access to handwashing facilities, unsafe sanitation, and unsafe water sources contributed to diarrhea-related deaths and DALYs among the older adult. The precise definitions of these risk factors, as well as the methods for quantifying their percentage contributions to diarrhea-related deaths and DALYs among the older adult, have been published in previous studies (11, 16–18).

### 2.4 Statistical analysis

Incidence, prevalence, mortality, and DALYs are the main indicators used to describe the burden of Diarrhea disease in the older adult. Each rate is calculated per 100,000 population and is accompanied by a 95% uncertainty interval as determined by the GBD methodology (19). We conducted an in-depth analysis of the dynamic changes in the burden of Diarrhea disease in the older adult by calculating the EAPC to identify the trends in disease burden over time (20). The 95% CI for the EAPC is calculated via linear regression. A negative EAPC and a 95% CI upper limit indicate a decreasing trend, while a positive EAPC and a 95% CI lower limit suggest an increasing trend in rates (12). Gaussian curve analysis assesses the relationship between EAPC and age-standardized rates (ASR) and the human development index (HDI) (21). Exponential Smoothing (ES), a time series forecasting technique, predicts future trends by focusing on recent data patterns. It effectively smooths short-term variations to reveal underlying long-term trends or cycles (22). The population attributable fractions (PAFs) of the risk factors were quantified by estimating the exposure distributions of the risk factors, the relative risks associated with each risk factor and outcome, and determining the theoretical minimum risk exposure level (TMREL). The PAF is the fraction of older adult diarrhea disease DALYs and deaths that would have been reduced if the exposure to the risk factor had been at the TMREL. The attributable burden was computed by multiplying the location-year-age-sex-specific PAFs of risk factors by corresponding rates of diarrhea disease DALYs or mortality (11, 16–18). All calculations were performed using R Studio version 4.1.2 (R Project for Statistical Computing). All *P*-values were two-sided, with *P* < 0.05 considered to indicate statistical significance.

## 3 Results

### 3.1 Diarrhea disease in the older adult: global trends

#### 3.1.1 Incidence

**Figure 1:**
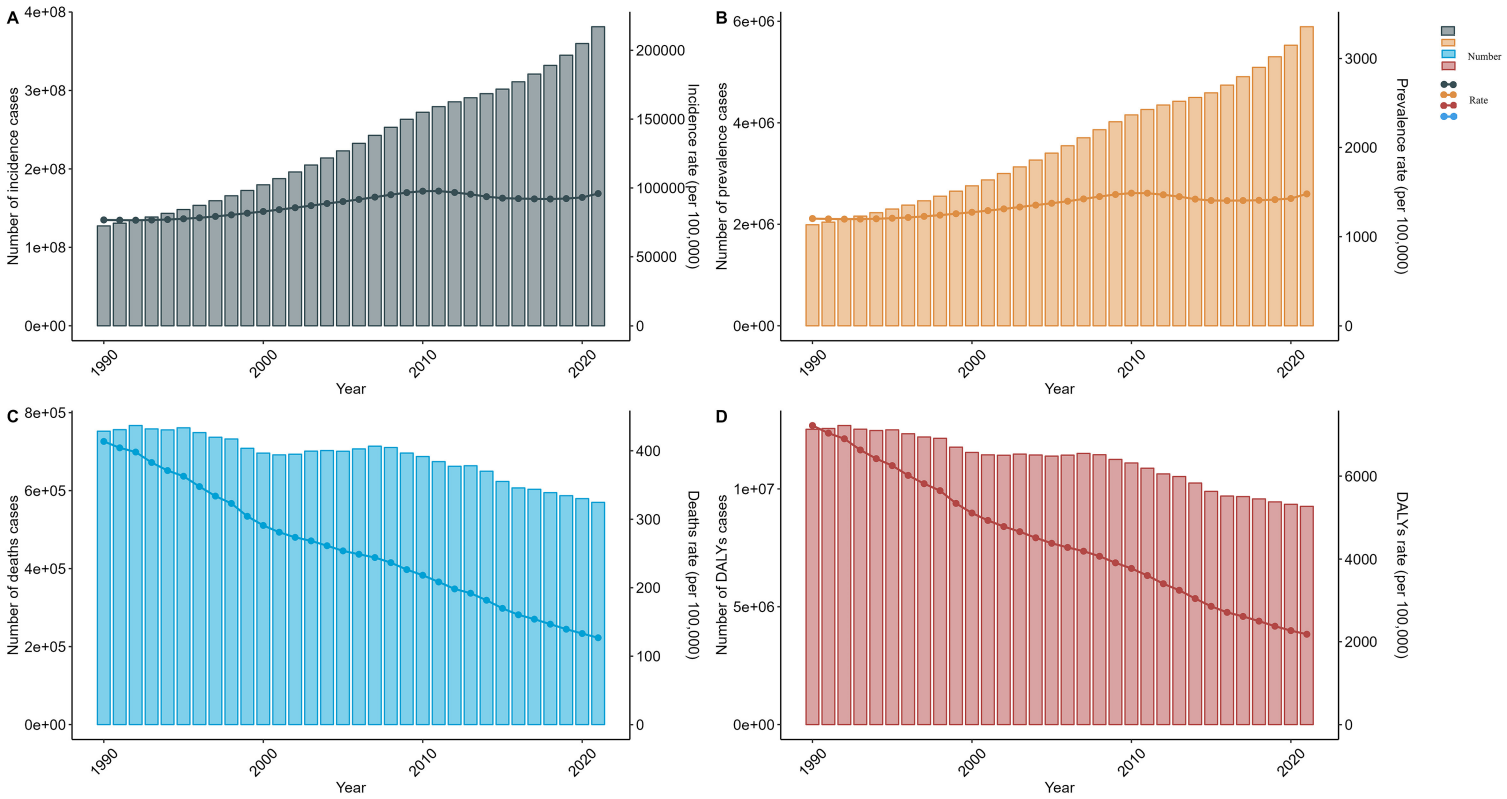
Trends in the incidence, prevalence, mortality, and DALYs for older adult diarrhea from 1990 to 2021. **(A)** Trends in incident cases and incidence rate. **(B)** Trends in prevalence cases and prevalence rate. **(C)** Trends in death cases and death rate. **(D)** Trends in DALYs cases and DALYs rate.

**Figure 2:**
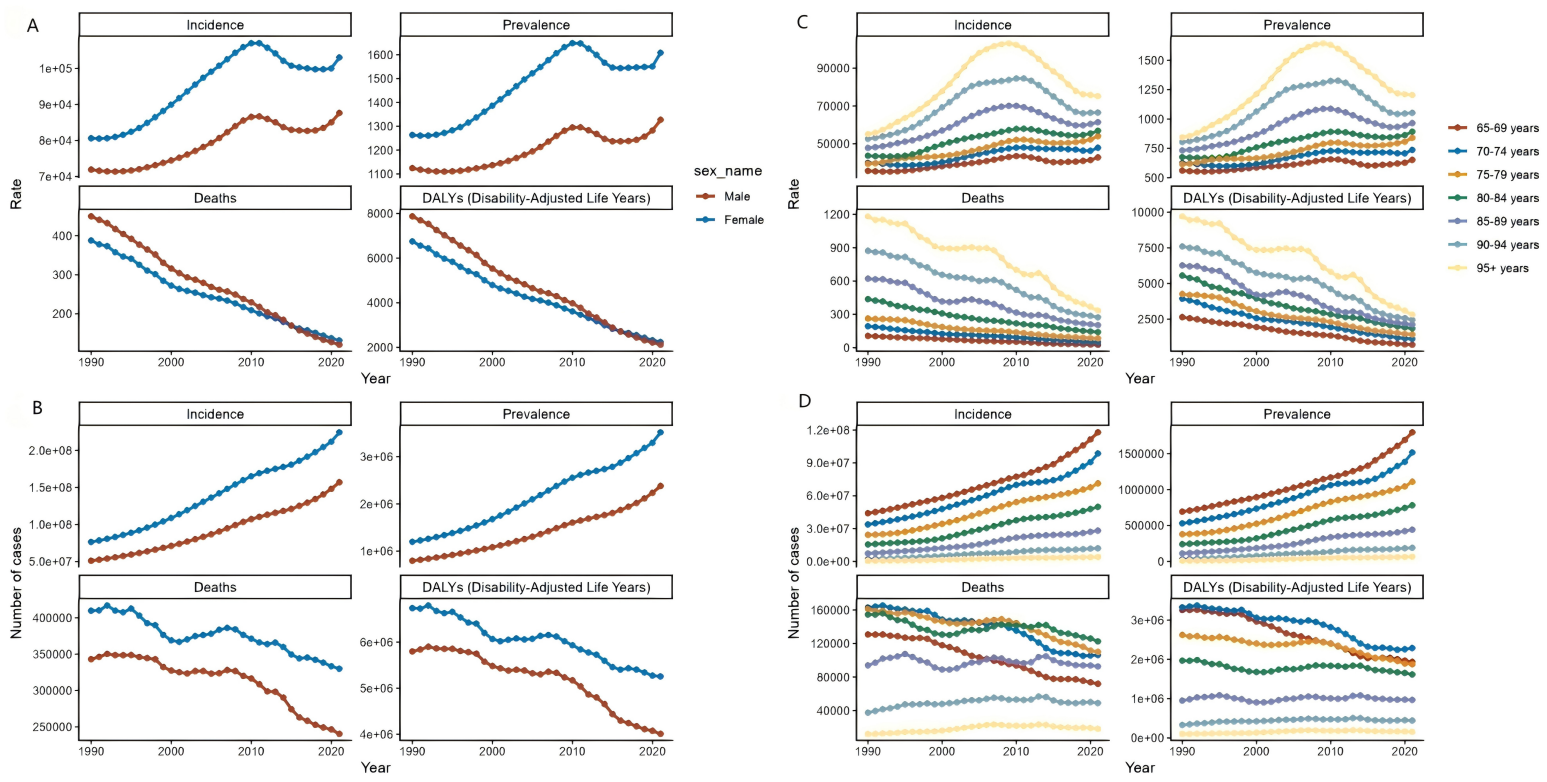
Trends in incidence, prevalence, mortality, and DALYs for Diarrhea disease in the older adult, stratified by age group and sex group, from 1990 to 2021. **(A)** Trends in incidence rate, prevalence rate, death rate, and DALYs rate for Diarrhea disease in the older adult, stratified by sex group. **(B)** Trends in incident cases, prevalence cases, death cases, and DALYs cases for Diarrhea disease in the older adult, stratified by sex group. **(C)** Trends in incidence rate, prevalence rate, death rate, and DALYs rate for Diarrhea disease in the older adult, stratified by age group. **(D)** Trends in incident cases, prevalence cases, death cases, and DALYs cases for Diarrhea disease in the older adult, stratified by age group.

In 2021, the global older adult Diarrhea disease incidents reached 381,192,184 (95% UI, 326, 402, 466–437, 756, 869), a 199.41% increase since 1990. The incidence rate rose from 76,826.9 (95% UI, 62,910.32–92,984.03) in 1990 to 95,984.87 (95% UI, 82,220.77–110,027.64) in 2021, with an EAPC of 0.83 (95% CI, 0.68–0.98). The over-95 age group had the highest increase in rates (EAPC 1.31, 95% CI, 0.71–1.92), while the 65–69 group had the lowest (EAPC 0.64, 95% CI, 0.50–0.78). In 2021, the over-95s had an incidence rate of 75,202.72 per 100,000 (95% UI, 60,919.53–89,373.53). Incidents for both genders increased until 2010, with women leading, then declined and rebounded by 2021, showing a bimodal pattern (Table 1, Figures 1A, 2).

**Table 1:** Incidence of Diarrhea disease in the older adult from 1990 to 2021 at the global, sex, age-group, and SDI regional levels.

	**Rate per 100 000 (95% UI)**			
	**1990**		**2021**		**1990–2021**
**Dimension**	**Incidence cases**	**Incidence rate**	**Incidence cases**	**Incidence rate**	**EAPC** ^*^
Global	127,316,258 (103,979,419–154,688,625)	76,826.9 (62,910.32–92,984.03)	381,192,184 (326,402,466–437,756,869)	95,984.87 (82,220.77–110,027.64)	0.83 (0.68–0.98)
**Sex**
Female	76,343,860 (62,474,066–92,122,729)	80,596.98 (66,174.4–96,850.72)	224,313,594 (193,981,855–253,757,619)	103,014.99 (89,092.8–116,523.27)	0.93 (0.74–1.12)
Male	50,972,398 (41,470,691–62,529,114)	71,901.53 (58,619.07–87,854.94)	156,878,590 (132,240,701–184,332,896)	87,604.4 (73,947.92–102,626.85)	0.72 (0.61–0.83)
**Age**
65–69 years	43,957,511 (36,427,782–52,260,684)	35,561.61 (29,470.06–42,278.87)	117,804,628 (101,072,270–134,035,458)	42,707.26 (36,641.34–48,591.36)	0.64 (0.5–0.78)
70–74 years	33,715,318 (27,445,567–41,498,756)	39,823.83 (32,418.13–49,017.47)	98,453,378 (83,641,338–114,614,454)	47,830.23 (40,634.31–55,681.54)	0.84 (0.7–0.97)
75–79 years	24,207,995 (18,575,942–30,908,332)	39,327.09 (30,177.54–50,212.13)	71,149,702 (58,768,097–84,721,746)	53,948.53 (44,560.31–64,239.4)	1 (0.88–1.11)
80–84 years	15,411,712 (12,245,955–19,063,215)	43,565.53 (34,616.63–53,887.53)	497,459,65 (42,121,782–57,522,654)	56,798.62 (48,093.53–65,677.83)	1 (0.81–1.2)
85–89 years	7,212,014 (5,930,791–8,646,898)	47,726.68 (39,247.98–57,222.27)	28,052,932 (24,099,460–32,135,941)	61,355.72 (52,708.91–70,285.83)	0.95 (0.62–1.28)
90–94 years	2,250,941 (1,774,743–2,786,047)	52,528.41 (41,415.77–65,015.76)	11,886,799 (9,965,902–13,763,668)	66,446.14 (55,708.5–76,937.67)	1 (0.51–1.49)
95+ years	560,768 (416,995–738,936)	55,080.59 (40,958.66–72,580.91)	4,098,780 (3,320,303–4,871,134)	75,202.72 (60,919.53–89,373.53)	1.31 (0.71–1.92)
**SDI region**
High	17,285,558 (14,382,393–20,563,635)	31,277.84 (25,858.7–37,350.45)	48,085,212 (41,837,874–55,269,678)	41,195.19 (35,677.91–47,372.93)	1.55 (0.7–2.4)
High Middle	8,699,722 (7,026,533–10,560,436)	20,565.25 (16,563.96–24,951.17)	23,024,321 (19,796,096–26,483,218)	23,823.1 (20,388.08–27,424.96)	0.91 (0.61–1.21)
Middle	34,744,244 (28,188,018–42,513,472)	87,541.93 (71,219.29–106,683.21)	109,190,736 (92,335,276–126,827,404)	92,295.77 (78,181.96–107,014.8)	0.07 (0–0.14)
Low Middle	49,649,516 (40,242,483–60,506,470)	219,187.29 (178,266.92–266,073.05)	148,320,366 (125,925,447–171,931,525)	251,006 (213,266.28–290,165.89)	0.36 (0.28–0.44)
Low	16,876,053 (13,809,826–20,641,070)	205,593.18 (168,718.64–250,224.58)	52,406,900 (44,913,806–60,074,112)	279,669.05 (239,846.36–320,211.21)	0.89 (0.76–1.02)

EAPC, estimated annual percentage change; SDI, Sociodemographic Index; UI, uncertainty interval. ^*^EAPC is expressed as 95% CI.

#### 3.1.2 Prevalence

From 1990 to 2021, global Diarrhea disease cases among the older adult increased by 195.95%, from 1,991,057 (95% UI, 1,682,776–2,331,376) to 5,892,437 (95% UI, 5,254,674–6,532,443) people. The prevalence rate also increased from 1,202.83 (95% UI, 1,018.76–1,404.84) to 1,479.68 (95% UI, 1,320.82–1,638.13) per 100,000 by 2021, with an EAPC of 0.75 (95% CI, 0.62–0.89). The age group 95 and above had the sharpest rise in prevalence (EAPC 1.46, 95% CI, 0.84–2.09). Females had significantly higher prevalence rates and case numbers than males, with an EAPC for females of 0.89 (95% CI, 0.72–1.07) and for males of 0.57 (95% CI, 0.48–0.67) (Figures 1B, 2 and Supplementary Table S1).

#### 3.1.3 Mortality

Over 32 years, Diarrhea disease-related deaths among the older adult globally fell 24.3%, from 752,336 in 1990 (95% UI, 484,580–1,141,072) to 569,830 in 2021 (95% UI, 347,143–880,194). The mortality rate declined from 413.47 per 100,000 in 1990 (95% UI, 264.96–632.11) to 126.76 per 100,000 in 2021 (95% UI, 76.8–196.98), with the EAPC at −3.67 (95% CI, −3.81 to −3.53). The 65–69 age group showed the largest reduction of 75.40%. In both 1990 and 2021, the 70–74 and 80–84 age groups had the most deaths. Mortality rates for both genders converged by 2015, and by 2021, women's rate slightly exceeded men's (Figures 1C, 2 and Supplementary Table S2).

#### 3.1.4 DALYs

Between 1990 and 2021, there was a 26.11% reduction in global DALYs for older adult Diarrhea disease, from 12,530,559 DALYs (95% UI, 8,017,128–19,219,924) in 1990 to 9,259,412 DALYs (95% UI, 5,757,427–14,175,665) in 2021. The 65–69 age group experienced the largest reduction at 31.12%. The DALYs rate decreased annually for all age groups, reaching a record low in 2021, especially for the 65–69 group (EAPC of −4.23, 95% CI, −4.45 to −4.01). DALYs rate for both older adult males and females have declined since 1990, with males showing a steeper decrease (EAPC, −4.15, 95% CI, −4.33 to −3.97) compared to females (EAPC, −3.45, 95% CI, −3.55 to −3.35) (Figures 1D, 2 and Supplementary Table S3).

### 3.2 Diarrhea disease in the older adult: SDI regional trends

#### 3.2.1 Incidence

**Figure 3:**
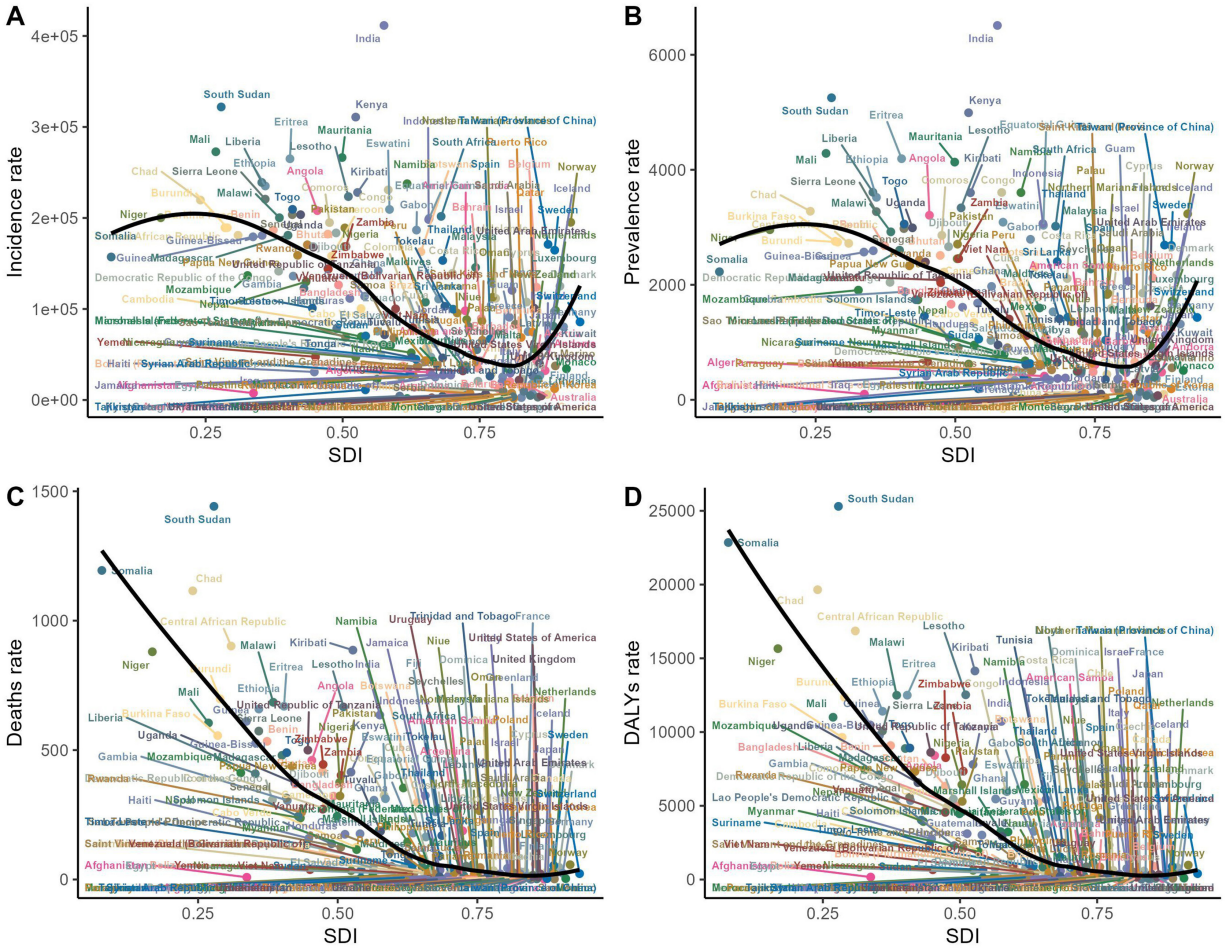
The relationship between incidence, prevalence, mortality, DALYs, and SDI for older adult diarrhea in 2021. **(A)** The relationship between incidence rate and SDI. **(B)** The relationship between prevalence rate and SDI. **(C)** The relationship between mortality rate and SDI. **(D)** The relationship between DALYs rate and SDI.

**Figure 4:**
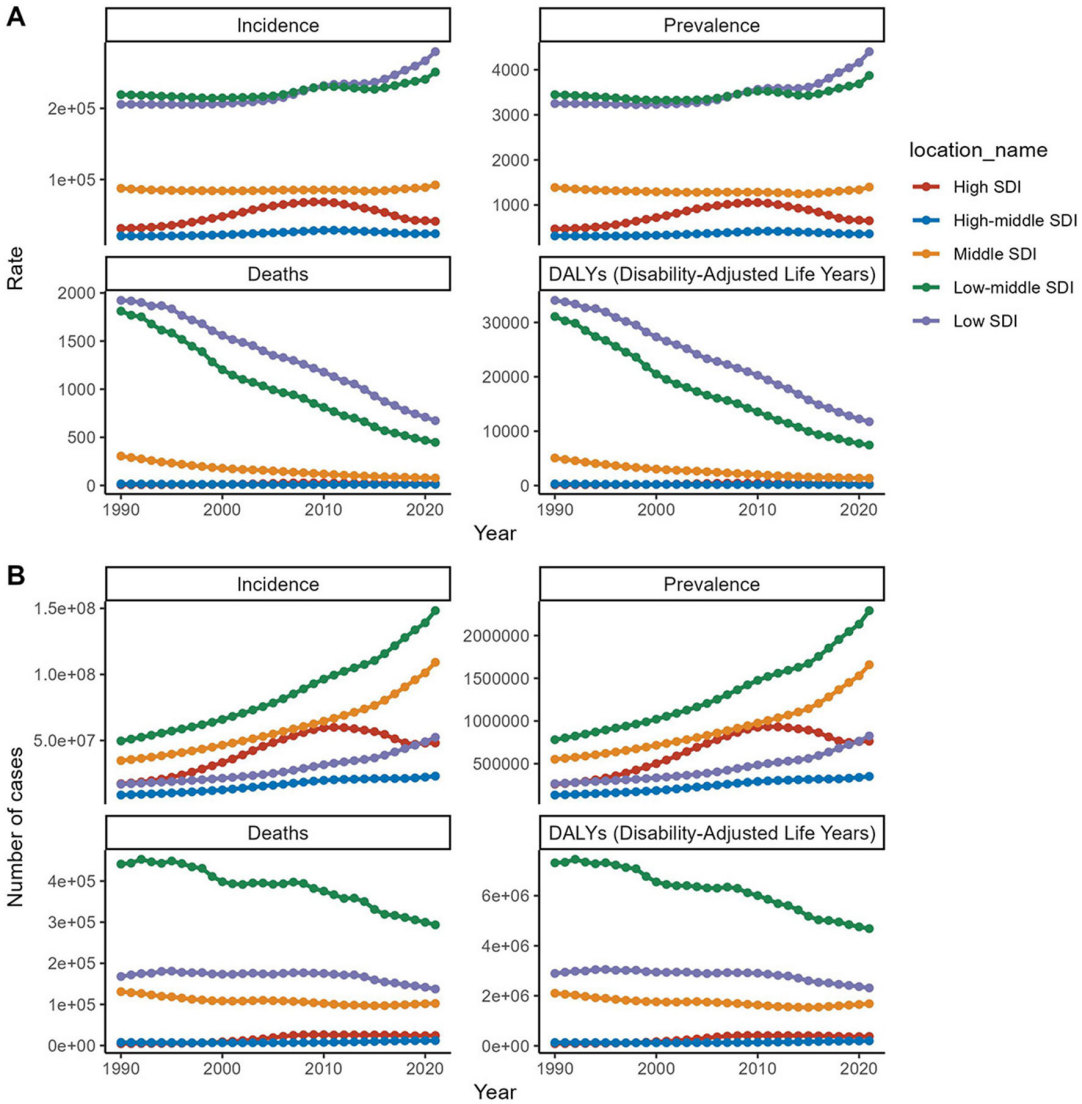
Trends in incidence, prevalence, mortality, and DALYs for diarrhea disease in the older adult, stratified by SDI, from 1990 to 2021. **(A)** Trends in incidence rate, prevalence rate, death rate, and DALYs rate for diarrhea disease in the older adult, stratified by SDI. **(B)** Trends in incident cases, prevalence cases, death cases, and DALYs cases for diarrhea disease in the older adult, stratified by SDI.

From 1990 to 2021, incident cases and incidence rates of Diarrhea disease in the older adult rose across all SDI regions. The low-middle SDI region reported the highest number of cases, increasing nearly 200% from 49,649,516 in 1990 (95% UI, 40,242,483–60,506,470) to 148,320,366 in 2021 (95% UI, 125,925,447–171,931,525). In terms of rates, the low SDI region led from around 2010, with a growing gap. However, the high SDI region showed the largest increase in incidence rates (EAPC, 1.55; 95% CI, 0.7–2.4). As SDI rises, older adult diarrhea incidence drops, but paradoxically climbs at higher SDI levels (Table 1, Figures 3A, 4).

#### 3.2.2 Prevalence

Low and low-middle SDI regions showed notably higher prevalence rates at 4,402.16 (95% UI, 3,918.87–4,877.61) and 3,873.07 (95% UI, 3,422.88–4,335.62) per 100,000 people, respectively. The low-middle SDI region also has the highest total number of prevalent cases, reaching 2,292,539 in 2021 (95% UI, 2,023,718–2,572,233). In contrast, the high SDI region experiences the largest increase in prevalence rates, with an EAPC of 1.75 (95% CI, 0.92–2.59). The prevalence rate's link to SDI mirrors the incidence-mortality relationship with SDI (Figures 3B, 4 and Supplementary Table S1).

#### 3.2.3 Mortality

Among the five SDI regions, only the high SDI region has seen an increase in the mortality rate related to Diarrhea disease in the older adult (EAPC, 4.67, 95% CI, 3.56–5.8). The most significant decrease is observed in the low-middle SDI region (EAPC, −4.5; 95% CI, −4.63 to −4.38). Despite the decrease in the number of deaths related to Diarrhea disease in the low-middle SDI region, it still has the highest number of deaths (293,494; 95% UI, 176,812–480,335). Mortality rates are negatively correlated with the SDI (Figures 3C, 4 and Supplementary Table S2).

#### 3.2.4 DALYs

In 2021, the low-middle SDI region had the highest number of DALYs (disability-adjusted life years) related to Diarrhea disease in the older adult (4,684,576; 95% UI, 2,858,834–7,577,174). It also experienced the largest reduction in DALYs, with a decrease of 35.96% from 1990 to 2021, and an EAPC of −4.63 (95% CI, −4.75 to −4.52). The high SDI region was the only one to see an increase in the DALYs rate related to Diarrhea disease in the older adult, with an increase of 115.4%, and it had the highest EAPC (3.68, 95% CI, 2.69–4.67). DALYs rate are inversely related to the SDI (Figures 3D, 4 and Supplementary Table S3).

### 3.3 Diarrhea disease in the older adult: GBD regions trends

#### 3.3.1 Incidence

**Figure 5:**
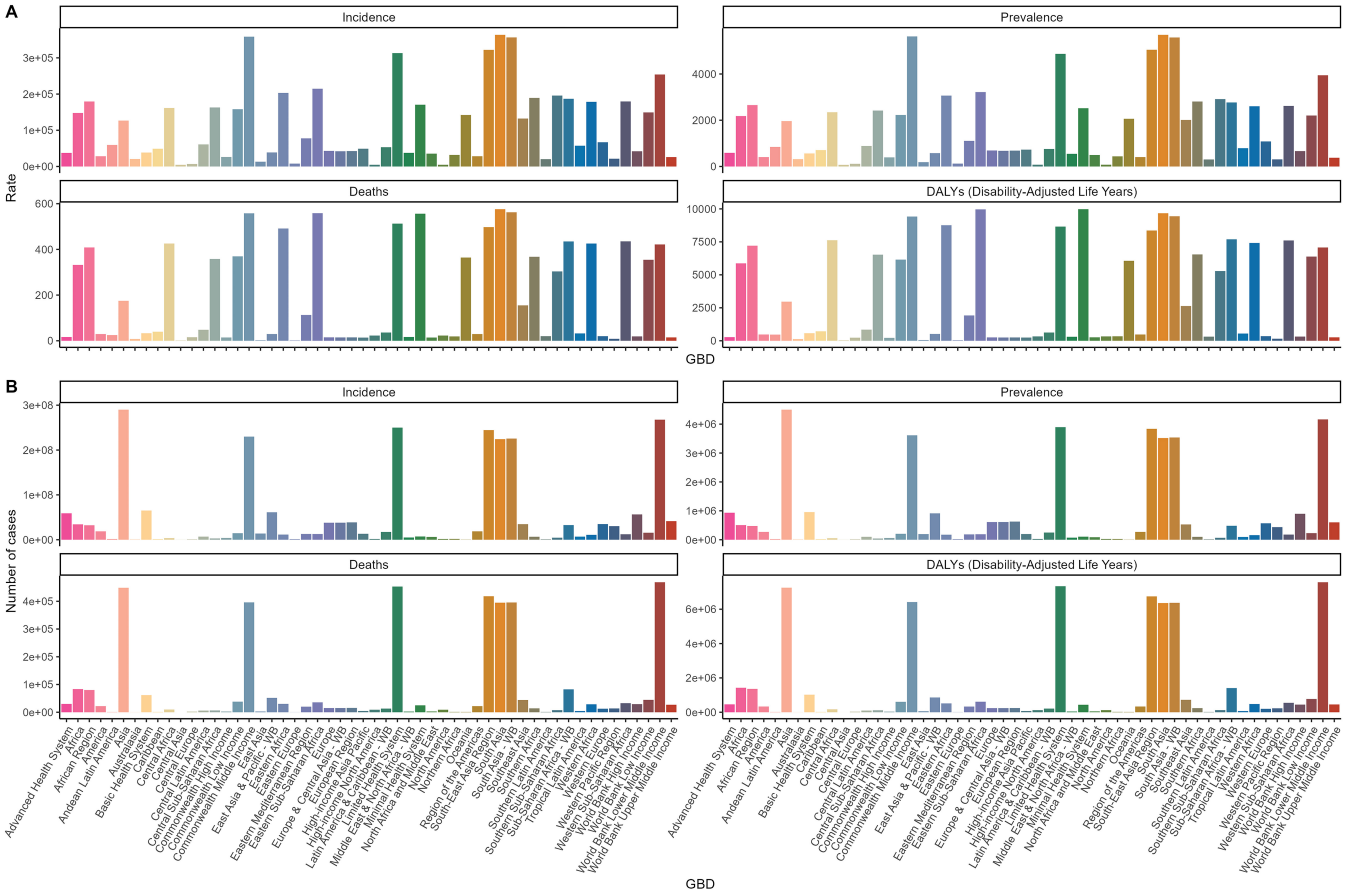
Trends in incidence, prevalence, mortality, and DALYs for diarrhea disease in the older adult, stratified by GBD regions, in 2021. **(A)** Trends in incidence rate, prevalence rate, death rate, and DALYs rate for diarrhea disease in the older adult, stratified by GBD regions. **(B)** Trends in incident cases, prevalence cases, death cases, and DALYs cases for diarrhea disease in the older adult, stratified by GBD regions.

In 2021, Asia, particularly South and Southeast Asia, had the highest older adult Diarrhea disease cases (289,973,661; 95% UI, 245,782,601–335,537,189) and incidence rates (363,457.58; 95% UI, 308,719.99–420,492.65). Central Europe had the largest increase in incidence rate (EAPC, 6.34; 95% CI, 5.67–7.02), while high-income North America saw the largest decrease (EAPC, −4.1; 95% CI, −5.42 to −2.76) (Figure 5 and Supplementary Table S4).

#### 3.3.2 Prevalence

South Asia led in older adult Diarrhea disease prevalence at 5,690.88 per 100,000 (95% UI, 4,993.91–6,356.53) with a modest EAPC of 0.67 (95% CI, 0.56–0.79). Asia and lower-middle-income countries had notably higher case numbers than other regions. Central Europe had the highest prevalence rate increase (EAPC, 6.9; 95% CI, 6.2–7.62) (Figure 5 and Supplementary Table S1).

#### 3.3.3 Mortality

Lower-middle-income regions, especially South and Southeast Asia, had the highest Diarrhea disease deaths among the older adult at 468,695 (95% UI, 276,411–751,039). South Asia had the highest mortality rate at 576.06 per 100,000 (95% UI, 342.1–956.67). High-income North America had the largest increase in mortality rate (EAPC, 8.95; 95% CI, 6.75–11.2), and East Asia had the largest decrease (EAPC, −7.9; 95% CI, −8.27 to −7.52) (Figure 5 and Supplementary Table S2).

#### 3.3.4 DALYs

The highest DALYs for older adult Diarrhea disease were in lower-middle-income regions at 7,573,076 (95% UI, 4,543,415–12,018,863), and the lowest in Central Asia at 950 per 100,000 (95% UI, 740–1,232). Central Asia also had the lowest DALYs rate at 31.45 per 100,000 (95% UI, 24.47–40.85). North America had the highest EAPC for DALYs at 7.94 (95% CI, 5.98–9.94), and East Asia the lowest at −7.05 (95% CI, −7.4 to −6.69) (Figure 5 and Supplementary Table S3).

### 3.4 Diarrhea disease in the older adult: national trends

#### 3.4.1 Incidence

**Figure 6:**
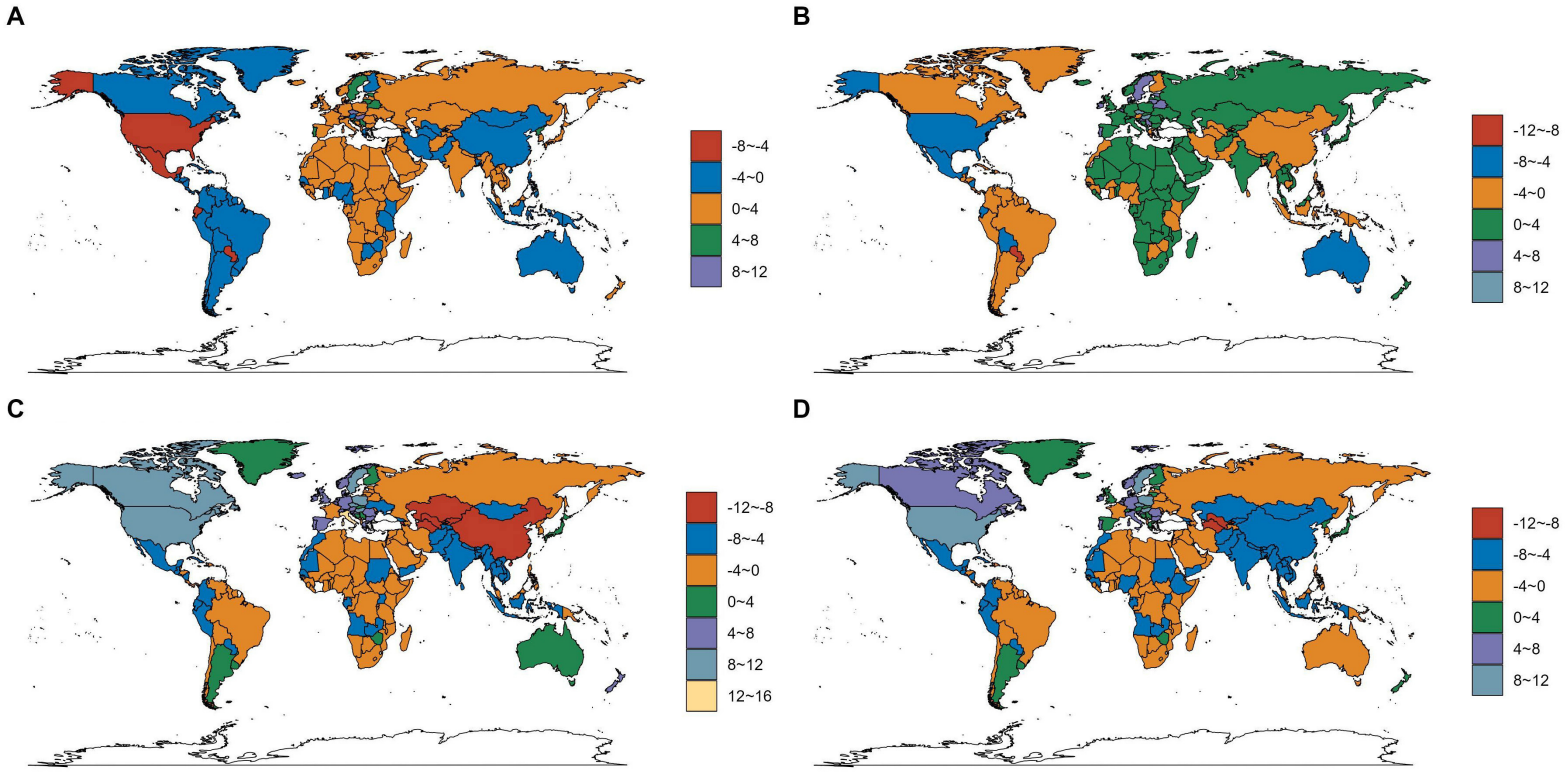
The EAPC in incidence, prevalence, mortality, and DALYs for older adult diarrhea across 204 countries and territories. **(A)** The EAPC for the incidence rate. **(B)** The EAPC for the prevalence rate. **(C)** The EAPC for the death rate. **(D)** The EAPC for the DALYs rate.

**Figure 7:**
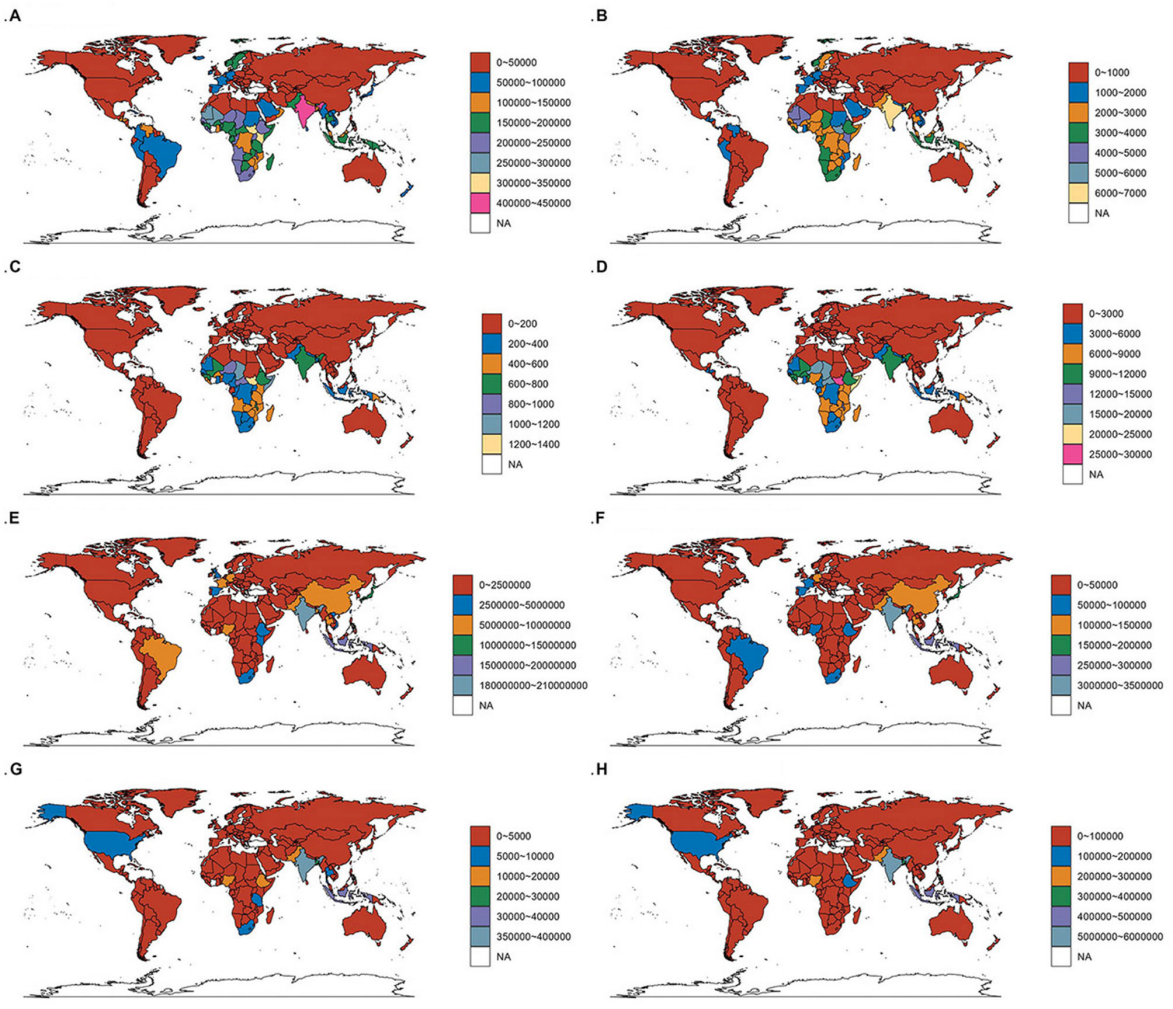
Incidence, prevalence, mortality, and DALYs for diarrhea disease among the older adult across 204 countries and territories. **(A–D)** Incidence rate, prevalence rate, death rate, and DALYs rate for diarrhea disease among the older adult across 204 countries and territories. **(E–H)** Incident cases, prevalence cases, death cases, and DALYs cases for diarrhea disease among the older adult across 204 countries and territories.

In 2021, India reported the highest older adult Diarrhea disease cases (206,921,994; 95% UI, 175,207,727–239,954,622) and incidence rate at 411.55 per 100,000 people (95% UI, 348.59–476.35). The Czech Republic saw the largest increase in incidence rate (EAPC, 10.96; 95% CI, 9.85–12.08), Paraguay the largest decrease (EAPC, −7.83; 95% CI, −8.58 to −7.08) (Figures 6A, 7A, E and Supplementary Table S4).

#### 3.4.2 Prevalence

India led in prevalence with 3,280,966 cases (95% UI, 2,861,330–3,667,613) and a rate of 6,509.88 per 100,000 people (95% UI, 5,691–7,266.68), EAPC 0.83 (95% CI, 0.71–0.96). Paraguay had the largest decrease in prevalence rate (EAPC, −8.83; 95% CI, −9.63 to −8.03), the Czech Republic the largest increase (EAPC, 11.86; 95% CI, 10.63–13.11) (Figures 6B, 7B, F and Supplementary Table S1).

#### 3.4.3 Mortality

India reported the most older adult Diarrhea disease-related deaths (354,493; 95% UI, 206,953–586,779). South Sudan had the highest mortality rate (1,441.68; 95% CI, 722.12–2,645.42) per 100,000. Italy saw the greatest increase in mortality rate (EAPC, 12.35; 95% CI, 10.36–14.36) (Figures 6C, 7C, G and Supplementary Table S2).

#### 3.4.4 DALYs

India topped DALYs with 5,739,336 (95% UI, 3,462,316-9,400,221). South Sudan had the highest DALYs rate (25,303.65; 95% UI, 12,755.31–47,006.21) per 100,000. Poland saw the largest increase in DALYs rate (EAPC, 10.01; 95% CI, 8.1–11.96), Turkmenistan the largest decrease (EAPC, −8.41; 95% CI, −9.24 to −7.58) (Figures 6D, 7D, H and Supplementary Table S3).

### 3.5 Risk factors for diarrhea disease in the older adult

The GBD database has identified the following three risk factors for diarrhea in the older adult: no access to hand washing facility, unsafe sanitation, and unsafe water source. We have conducted an analysis of the mortality and DALY rates related to these risk factors for older adult diarrhea.

**Figure 8:**
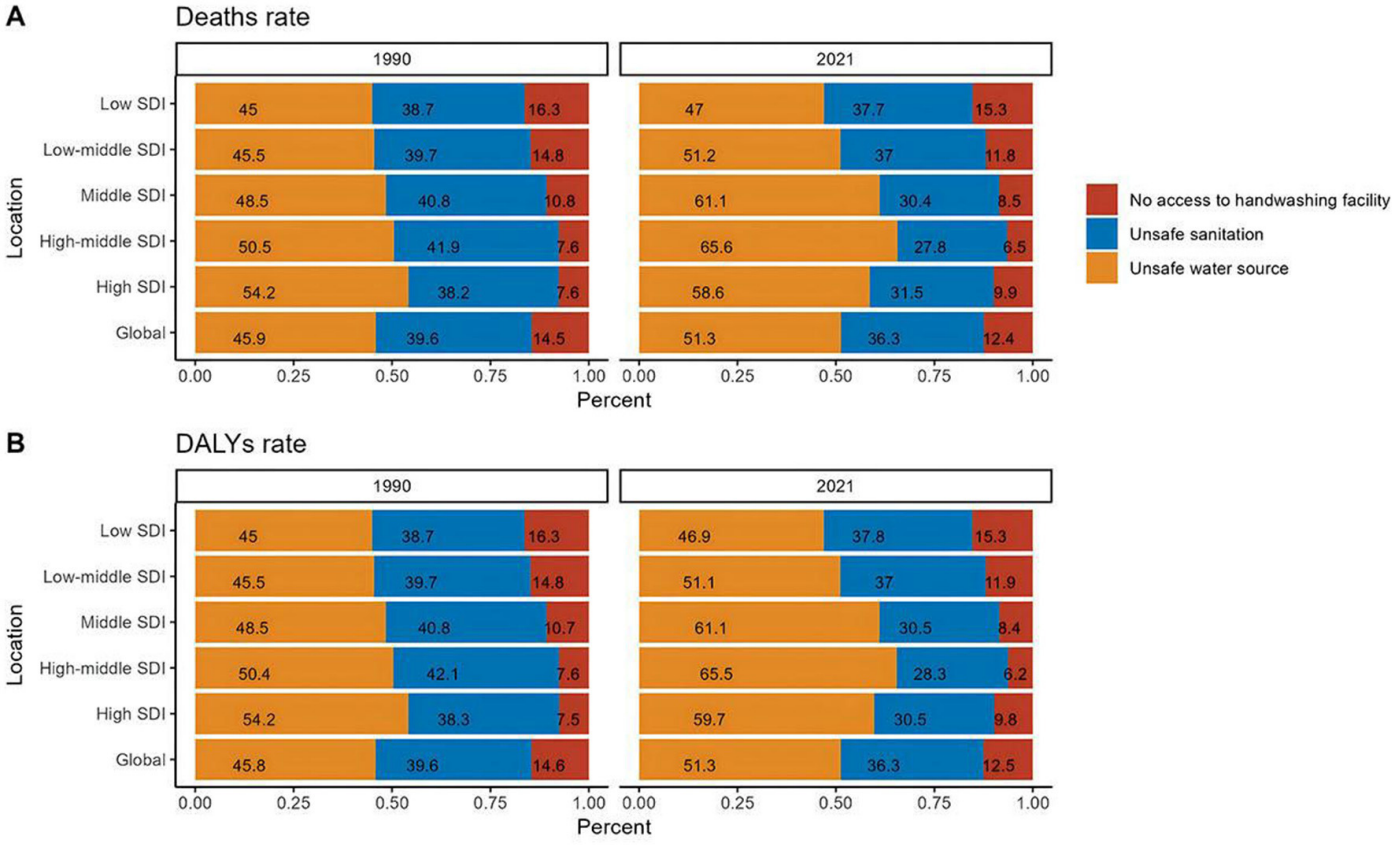
Comparative analysis of the proportion of risk factors for diarrhea disease mortality and DALYs rate among the older adult population in 1990 vs. 2021. **(A)** Comparative analysis of the proportion of risk factors for diarrhea disease-related mortality among the older adult population in 1990 vs. 2021. **(B)** Comparative analysis of the proportional contribution of risk factors to diarrhea disease-related DALYs rate among the older adult population in 1990 and 2021.

Between 1990 and 2021, unsafe water source were the primary cause of diarrhea-related deaths among the older adult, with their share of global mortality increasing from 45.9% to 51.3% by 2021. This issue was most pronounced in middle-high SDI regions, where it accounted for over 65% of such deaths. Despite a general rise in mortality rates due to unsafe water across all SDI regions, the lack of hand washing facility and unsafe sanitation, while declining, still made up half of the mortality rate in low SDI areas in 2021. The data underscores the need for improved water safety, particularly in middle-high SDI regions, to lessen the disease burden of diarrhea. Regarding DALY rates, unsafe water source remained the key risk factor, with over 50% of the global burden in 2021. There was a notable increase in the proportion of unsafe water source in middle-high SDI regions, where improvements in social infrastructure have not eliminated water safety as a major public health concern. In contrast, low SDI regions showed little change in risk factors over the 31-year period, highlighting the persistent challenge of addressing diarrhea-related DALYs (Figure 8 and Supplementary Table S5).

## 4 Discussion

This study indicates that between 1990 and 2021, there have been significant changes in the burden and risk factors of Diarrhea disease among the older adult at the global and regional levels. Diarrhea disease is a globally prevalent illness with a high incidence rate (1), often triggered by infections in the gastrointestinal tract from bacteria, viruses, or parasites (23). It can also be caused by non-infectious gastrointestinal conditions such as inflammatory bowel diseases (24). From 1990 to 2021, a significant decline was observed in both the mortality rate and the DALYs rate for older adult Diarrhea disease globally. Despite this, incidence and prevalence rates have paradoxically increased by nearly 200% during the same timeframe, with high SDI regions experiencing the steepest rise in incidence rates. Interestingly, within these regions, mortality and DALYs rate are also the only ones trending upwards. This increase could be linked to the rapid aging of populations in high SDI areas, the growing prevalence of chronic diseases among the older adult, and evolving healthcare standards in older adult care facilities.

Our research uncovers a complex relationship between the SDI and Diarrhea disease among the older adult, showing a decrease in incidence and prevalence as the SDI improves, but a surprising increase at higher SDI levels. This increase may be tied to the aging population and the higher prevalence of chronic diseases, which, along with their treatments, could trigger diarrhea. High-income countries face a significant burden of Clostridium difficile infections, which are challenging to treat and often antibiotic-resistant, frequently occurring in healthcare settings. The SDI-health outcome link is intricate, necessitating sophisticated strategies to combat the rising older adult Diarrhea disease burden in high SDI regions (11, 25–27).

Additionally, the widespread adoption of community older adult care institutions may have also influenced this phenomenon to a certain extent. As the older adult become more dependent on community older adult care, the collective living environment further increases the risk of transmission of infectious diseases. Therefore, despite the more advanced healthcare systems in high SDI areas, the prevention and treatment of Diarrhea disease in the older adult still face a series of new challenges. This requires us to not only focus on the treatment of diseases but also to pay attention to the implementation of preventive measures and to strengthen the control of infections within older adult care institutions. There is a significant negative correlation between SDI and the mortality rate related to older adult Diarrhea disease, as well as the DALYs rate related to Diarrhea disease, especially in low-middle SDI areas, where the downward trend in the mortality rate related to older adult Diarrhea disease is particularly significant. This positive change may be attributed to the region's progress in several key areas: significant improvement in sanitary conditions, positive changes in public health habits, the popularization of clean water sources, and the widespread implementation of rotavirus vaccination. This has enabled older adult Diarrhea disease to be prevented and better treated (11, 28–30).

This research pinpoints unsafe water sources as a principal risk factor for Diarrhea disease mortality and DALYs among the older adult, especially in areas characterized by higher SDI, where water safety is essential for mitigating the disease burden. Although there have been numerous studies reporting on the global burden of Diarrhea disease, as well as the incidence, mortality, and DALYs rate for Diarrhea disease in children and specific regions (3, 6, 9, 11, 18, 31), this study is the first to conduct a comprehensive analysis of the global and regional burden related to Diarrhea disease in the older adult. Leveraging the most recent epidemiological data from 1990 to 2021, which covers 204 countries and territories worldwide, this study thoroughly reveals the trends in the disease burden of older adult Diarrhea disease, with detailed breakdowns by GBD regions, SDI, different age groups, and gender. It is particularly worth emphasizing that this research comprehensively explores this topic at the national, regional, and global levels for the first time, providing a reference for academic research and the formulation of public health policies in this field. This study also has certain limitations. First, the GBD data may have reporting biases in low-SDI regions. Second, the study fails to consider key factors such as multimorbidity, medication use, and healthcare accessibility among the older adult. Additionally, the observational nature of the GBD data limits causal inferences. These issues highlight the need for future studies to incorporate individual-level clinical data to address these gaps.

## Data Availability

Publicly available datasets were analyzed in this study. This data can be found here: https://www.healthdata.org/research-analysis/gbd.
