# Supplementary material for: Global burden of diarrhea disease in the older adult and its attributable risk factors from 1990 to 2021: a comprehensive analysis from the global burden of disease study 2021
Source: Front Public Health. 2025 Apr 4;13:1541492. doi: 10.3389/fpubh.2025.1541492 (PMC12006145; doi:10.3389/fpubh.2025.1541492)
Supplement: Supplementary file 1 [file Table_1.docx]

**Supplementary Material**

eTable 1. Prevalence of diarrhea disease in the elderly at the global, sex, age-group levels, regional, ,and national levels from 1990 to 2021.

| **Location** | **Rate per 100 000(95% UI)** | | | | |
| --- | --- | --- | --- | --- | --- |
|  | **1990** | | **2021** | | **1990-2021** |
|  | **Prevalence cases** | **Prevalence rate** | **Prevalence cases** | **Prevalence rate** | **EAPC** |
| Global | 1991057 (1682776-2331376) | 1202.83 (1018.76-1404.84) | 5892437 (5254674-6532443) | 1479.68 (1320.82-1638.13) | 0.75 (0.62-0.89) |
| Sex |  |  |  |  |  |
| Female | 1195801 (1013446-1393943) | 1262.89 (1072.71-1467.94) | 3514785 (3157662-3864064) | 1607.8 (1444.86-1765.79) | 0.89 (0.72-1.07) |
| Male | 795255 (670343-936966) | 1123.79 (949.36-1320.67) | 2377652 (2087365-2694343) | 1326.38 (1166.74-1498.39) | 0.57 (0.48-0.67) |
| Age |  |  |  |  |  |
| <65 years | 0 (0-0) | 0 (0-0) | 0 (0-0) | 0 (0-0) | 0 (0-0) |
| 65-69 years | 691780 (591560-798602) | 559.65  (478.57-646.07) | 1795758 (1609442-1976649) | 651.01 (583.46-716.59) | 0.49 (0.37-0.61) |
| 70-74 years | 528847 (442140-621922) | 624.66  (522.25-734.6) | 1514870 (1331269-1683912) | 735.95 (646.75-818.07) | 0.74 (0.61-0.87) |
| 75-79 years | 378147 (300659-462650) | 614.32  (488.44-751.6) | 1106620 (948849-1268527) | 839.08 (719.46-961.85) | 0.96 (0.85-1.06) |
| 80-84 years | 238746 (196831-287810) | 674.88  (556.4-813.58) | 780702 (688422-885972) | 891.38 (786.02-1011.58) | 1.03 (0.85-1.21) |
| 85-89 years | 110558  (94135-129079) | 731.63  (622.96-854.2) | 440735 (394243-486788) | 963.95 (862.27-1064.67) | 1.04 (0.71-1.37) |
| 90-94 years | 34397  (27784-41098) | 802.7 (648.38-959.08) | 188113  (162330-211594) | 1051.54 (907.41-1182.79) | 1.13 (0.64-1.63) |
| 95+ years | 8581 (6587-10793) | 842.88 (647.02-1060.15) | 65638  (54667-75934) | 1204.29 (1003.01-1393.2) | 1.46 (0.84-2.09) |
| SDI region |  |  |  |  |  |
| High-middle SDI | 132865 (110789-156022) | 314.36 (261.64-370.05) | 350888 (314727-386456) | 361.31 (322.44-399.33) | 0.89 (0.63-1.15) |
| High SDI | 259324 (222186-300066) | 469.68 (398.39-548.05) | 763114 (691034-838889) | 650.87 (585.6-719.2) | 1.75 (0.92-2.59) |
| Low-middle SDI | 780542 (646284-920907) | 3448.04 (2867.33-4055.99) | 2292539 (2023718-2572233) | 3873.07 (3422.88-4335.62) | 0.25 (0.15-0.34) |
| Low SDI | 266228 (225707-311121) | 3250.29 (2767.44-3783.86) | 824842 (732301-915126) | 4402.16 (3918.87-4877.61) | 0.8 (0.65-0.95) |
| Middle SDI | 551124 (462509-649962) | 1388.06 (1168.04-1632.15) | 1658542 (1461461-1854957) | 1398.04 (1234.27-1561.37) | -0.12 (-0.21--0.02) |
| GBD region |  |  |  |  |  |
| Advanced Health System | 309165 (265540-357439) | 387.61 (329.29-452.03) | 933638 (847642-1025235) | 590.32 (532.13-651.51) | 2.12 (1.36-2.88) |
| Africa | 255708 (219291-295733) | 2437.29 (2096.5-2810.72) | 504328 (454970-557331) | 2179.37 (1969.61-2402.97) | -0.54 (-0.65--0.44) |
| African Region | 247602 (212057-286555) | 2983.93 (2563.17-3443.71) | 475547 (428296-526111) | 2655.12 (2396.55-2930.61) | -0.57 (-0.68--0.46) |
| America | 195120 (169193-223323) | 683.73 (593.48-782.29) | 270075 (243951-301238) | 406.03 (366.7-451.72) | -1.96 (-2.29--1.64) |
| Andean Latin America | 20886 (18867-23087) | 2498.09 (2263.97-2752.2) | 22534 (20404-24906) | 845.53 (765.54-934.06) | -4.5 (-4.98--4.03) |
| Asia | 1352941 (1121638-1605757) | 1726.66 (1436.05-2043.05) | 4500375 (3969457-5031775) | 1961.31 (1732.62-2189.04) | 0.41 (0.31-0.5) |
| Australasia | 7746 (6520-9117) | 657.17 (549.68-775.48) | 9294 (8222-10378) | 317.15 (277.84-357.13) | -2.46 (-3.85--1.06) |
| Basic Health System | 533311 (446260-629688) | 916.79 (769.32-1079.44) | 955725 (849975-1076368) | 564.16 (502-634.21) | -1.66 (-1.73--1.59) |
| Caribbean | 8170 (7533-8789) | 713.54 (658.26-766.03) | 17402 (15925-18940) | 708.25 (645.97-773.79) | -0.53 (-0.69--0.37) |
| Central Africa | 18524 (16773-20491) | 1853.7 (1680.6-2048.38) | 50644 (46369-55331) | 2346.09 (2149.43-2561.71) | 0.71 (0.56-0.85) |
| Central Asia | 1332 (1156-1511) | 82.25 (70.69-93.98) | 2102 (1748-2470) | 71.47 (59.09-84.26) | -0.31 (-0.48--0.13) |
| Central Europe | 1614 (1301-1987) | 23.54 (18.9-29.03) | 14393 (13181-15854) | 114.75 (104.8-126.49) | 6.9 (6.2-7.62) |
| Central Latin America | 72747 (62996-83449) | 2157.79 (1874.34-2467.58) | 99473 (91317-108603) | 885.07 (813.17-964.29) | -3.25 (-3.55--2.94) |
| Central Sub-Saharan Africa | 12163 (10904-13493) | 1609.38 (1443.6-1784.82) | 41896 (38546-45656) | 2417.62 (2225.93-2633.31) | 1.32 (1.16-1.47) |
| Commonwealth High Income | 33865 (27523-40735) | 449.25 (361.5-543.58) | 59047 (51905-66573) | 393.8 (343.39-446.27) | -0.16 (-1.71-1.42) |
| Commonwealth Low Income | 88529 (80376-96605) | 2695.89 (2453.21-2936.5) | 204266 (183514-228310) | 2227.07 (2005.66-2482.26) | -0.73 (-0.82--0.63) |
| Commonwealth Middle Income | 986328 (800511-1179392) | 4365.96 (3558.91-5203.79) | 3614664 (3166634-4046812) | 5624.22 (4938.43-6287.4) | 0.65 (0.52-0.78) |
| East Asia | 146174 (117709-178087) | 428.12 (345.86-520.33) | 194546 (164651-226993) | 187.18 (157.51-218.94) | -2.59 (-2.81--2.37) |
| East Asia & Pacific - WB | 434087 (358387-518752) | 808.82 (670.06-963.76) | 913340 (808533-1028496) | 577.57 (511.21-649.67) | -1.01 (-1.11--0.91) |
| Eastern Africa | 66105 (57336-75490) | 2602.7 (2261.9-2967.56) | 172202 (154423-191766) | 3062.59 (2752.26-3403.81) | 0.32 (0.23-0.42) |
| Eastern Europe | 9558 (7456-11868) | 88.86 (68.5-111.08) | 19842 (15868-24174) | 123.92 (97.6-152.31) | 1.11 (0.79-1.44) |
| Eastern Mediterranean Region | 102715 (84196-124159) | 1472.76 (1211.65-1774.33) | 182279 (158236-209268) | 1107.8 (963.64-1269.14) | -0.97 (-1.19--0.76) |
| Eastern Sub-Saharan Africa | 74651 (65051-84672) | 2781.43 (2428.76-3149.94) | 189352 (170502-210581) | 3216.19 (2901.57-3569.9) | 0.25 (0.15-0.34) |
| Europe | 185873 (160805-212938) | 369.53 (315.11-427.46) | 610863 (558029-668609) | 690.33 (626.54-759.81) | 2.82 (1.88-3.77) |
| Europe & Central Asia - WB | 186631 (161490-213781) | 362.93 (309.56-419.78) | 611909 (558909-669821) | 675.42 (612.98-743.44) | 2.81 (1.88-3.76) |
| European Region | 188415 (163092-215771) | 363.94 (310.53-420.8) | 626348 (572655-685498) | 684.55 (621.91-753.09) | 2.85 (1.91-3.8) |
| High-income Asia Pacific | 61838 (49415-75451) | 655.84 (522.49-802.49) | 196086 (170515-226791) | 726.65 (624.88-844.63) | 0.72 (0.51-0.92) |
| High-income North America | 32283 (24625-40584) | 187.64 (140.56-237.75) | 27100 (23787-30607) | 76.04 (66.44-85.87) | -3.82 (-5.07--2.56) |
| Latin America & Caribbean - WB | 163232 (143665-184553) | 1462.52 (1290.22-1650.61) | 247038 (222958-275447) | 757.07 (682.95-842.27) | -2.4 (-2.59--2.21) |
| Limited Health System | 1105790 (914724-1307329) | 3862.37 (3207.79-4551.28) | 3895908 (3435347-4348775) | 4869.95 (4303.65-5427.39) | 0.57 (0.46-0.69) |
| Middle East & North Africa - WB | 16936 (14653-19350) | 366.14 (316.91-418.19) | 71040 (64080-78535) | 545.55 (491.03-603.55) | 1.51 (1.27-1.76) |
| Minimal Health System | 41818 (38093-45744) | 1926.78 (1757.78-2106.48) | 104654 (96734-113377) | 2518.74 (2331.02-2725.73) | 0.75 (0.67-0.83) |
| North Africa and Middle East | 19054 (16508-21775) | 303.48 (263.18-346.43) | 86361 (77481-96217) | 495.56 (443.52-552.19) | 1.7 (1.58-1.81) |
| North America | 32294 (24636-40595) | 187.69 (140.61-237.8) | 27141 (23825-30653) | 76.16 (66.56-86) | -3.81 (-5.06--2.55) |
| Northern Africa | 7265 (6328-8315) | 311.73 (272.01-355.61) | 25782 (22965-28830) | 438.68 (390.21-490.61) | 0.89 (0.78-0.99) |
| Oceania | 2121 (1901-2347) | 2151.47 (1930.36-2379.7) | 5082 (4630-5551) | 2061.88 (1879.46-2249.78) | -0.41 (-0.48--0.33) |
| Region of the Americas | 195120 (169193-223323) | 683.73 (593.48-782.29) | 270075 (243951-301238) | 406.03 (366.7-451.72) | -1.96 (-2.29--1.64) |
| South-East Asia Region | 1003516 (826744-1187516) | 4032.6 (3334.52-4762.66) | 3838053 (3363186-4292174) | 5045.5 (4431.49-5632.86) | 0.59 (0.47-0.71) |
| South Asia | 900538 (735825-1071709) | 4368.02 (3584.14-5186.22) | 3519174 (3081727-3936784) | 5690.88 (4993.91-6356.53) | 0.67 (0.56-0.79) |
| South Asia - WB | 907389 (741808-1079169) | 4257.85 (3495.8-5052.07) | 3537898 (3098842-3956666) | 5577 (4895.01-6227.58) | 0.69 (0.58-0.81) |
| Southeast Asia | 223564 (186585-264964) | 2366.92 (1984.08-2796.38) | 528183 (469749-590341) | 2010.74 (1790.54-2242.44) | -0.56 (-0.71--0.4) |
| Southern Africa | 42826 (36479-49713) | 2628.14 (2243.26-3040.01) | 98091 (87676-109199) | 2808.62 (2515.96-3120.28) | 0.26 (0.13-0.38) |
| Southern Latin America | 15526 (14124-16968) | 724.19 (655.45-796.41) | 13366 (12227-14499) | 302.31 (275.23-329.06) | -2.56 (-3.73--1.37) |
| Southern Sub-Saharan Africa | 31572 (26284-37650) | 2986.73 (2490.01-3548.51) | 65712 (57141-75452) | 2913.48 (2538.26-3338.02) | 0.08 (-0.08-0.23) |
| Sub-Saharan Africa - WB | 249470 (213764-288663) | 3028.58 (2602.82-3494.64) | 481467 (434211-532637) | 2768.21 (2501.91-3055.74) | -0.49 (-0.6--0.38) |
| Tropical Latin America | 46192 (38415-54870) | 1256.13 (1049.52-1487.03) | 94895 (81535-111092) | 790.71 (679.96-923.82) | -1.45 (-1.8--1.1) |
| Western Africa | 120987 (101755-142856) | 4003.81 (3381.04-4707.5) | 157608 (141931-174486) | 2604.36 (2350.4-2877.56) | -1.75 (-1.88--1.63) |
| Western Europe | 174028 (151071-199232) | 586.57 (501.24-679.04) | 568461 (520113-622229) | 1083.07 (984.77-1190.95) | 2.68 (1.68-3.7) |
| Western Pacific Region | 240216 (195729-289321) | 511.01 (417.22-614.44) | 435870 (381376-495983) | 307.75 (268.2-350.9) | -1.5 (-1.62--1.38) |
| Western Sub-Saharan Africa | 129301 (109366-151805) | 3874.38 (3290.23-4531.48) | 177182 (159948-195510) | 2618.19 (2368.46-2882.94) | -1.62 (-1.74--1.5) |
| World Bank High Income | 301047 (259285-347488) | 455.98 (388.44-531) | 896493 (816354-984971) | 664.53 (601.15-733.82) | 1.94 (1.14-2.74) |
| World Bank Low Income | 83874 (74862-93497) | 1700.51 (1520.01-1893.15) | 228143 (207366-250280) | 2202.78 (2006.33-2411.36) | 0.76 (0.66-0.87) |
| World Bank Lower Middle Income | 1265885 (1047953-1499666) | 3187.57 (2650.11-3762.49) | 4164336 (3660174-4660837) | 3943.41 (3472.46-4406.37) | 0.58 (0.46-0.7) |
| World Bank Upper Middle Income | 339271 (285475-398817) | 587.37 (495.12-689.47) | 600931 (536491-671213) | 375.98 (335.29-419.79) | -1.44 (-1.5--1.37) |
| Country |  |  |  |  |  |
| Afghanistan | 189 (157-223) | 69.63 (57.91-81.96) | 305 (261-356) | 98.63 (84.45-114.96) | 1.32 (1.12-1.52) |
| Albania | 26 (22-31) | 32.02 (26.28-38.46) | 79 (63-97) | 35.26 (28.2-43.54) | 0.85 (0.54-1.17) |
| Algeria | 988 (841-1149) | 192.77 (164-223.58) | 5945 (5107-6787) | 412.25 (352.91-472.75) | 2.51 (2.45-2.56) |
| American Samoa | 8 (7-9) | 1026.95 (893.57-1170.65) | 25 (23-28) | 1335.37 (1201.07-1487.57) | 0.91 (0.77-1.04) |
| Andorra | 12 (9-15) | 464.27 (358.52-583.44) | 33 (28-40) | 480.9 (387.93-588.84) | 0.44 (-0.25-1.13) |
| Angola | 2502 (2201-2826) | 2079.58 (1830.29-2348.43) | 11813 (10742-12982) | 3209.45 (2919.86-3524.91) | 1.45 (1.27-1.64) |
| Antigua and Barbuda | 18 (16-19) | 670.91 (610.73-738.01) | 28 (25-31) | 607.03 (540.56-676.8) | -0.61 (-0.75--0.47) |
| Argentina | 7636 (6836-8585) | 513.99 (455.05-583.25) | 6188 (5525-6878) | 219.88 (194.38-246.25) | -2.12 (-3.55--0.66) |
| Armenia | 109 (94-128) | 126.99 (107.73-149.14) | 145 (119-173) | 74.38 (60.61-89.09) | -1.99 (-2.51--1.47) |
| Australia | 6340 (5335-7498) | 641.79 (536.77-760.62) | 4958 (4443-5524) | 201.18 (176.73-228.06) | -4.01 (-5.77--2.23) |
| Austria | 6283 (5169-7577) | 1038.15 (857.72-1245.41) | 6476 (5820-7288) | 653.54 (582.6-739.64) | -1.54 (-2.39--0.69) |
| Azerbaijan | 84 (68-102) | 53.81 (42.83-65.83) | 279 (217-352) | 82.03 (63.49-103.92) | 1.44 (1.27-1.62) |
| Bahamas | 20 (17-22) | 324.93 (288.53-363.07) | 43 (38-48) | 269.15 (236.07-302.57) | -0.58 (-0.75--0.42) |
| Bahrain | 28 (24-33) | 522.62 (444.63-609.72) | 365 (316-419) | 1364.06 (1180.91-1566.34) | 2.97 (2.6-3.35) |
| Bangladesh | 44780 (40684-49174) | 2475.5 (2252.09-2712.47) | 101195 (88003-116138) | 1662.36 (1447.47-1905.04) | -1.21 (-1.4--1.03) |
| Barbados | 72 (64-80) | 476.08 (425.34-529.17) | 120 (106-135) | 484.06 (422.84-546.97) | -0.39 (-0.5--0.27) |
| Belarus | 304 (219-400) | 59.62 (42.38-79.14) | 1449 (1132-1806) | 193.12 (149.1-242.77) | 4.52 (3.9-5.13) |
| Belgium | 5746 (4969-6550) | 719.03 (614.76-826.13) | 28481 (25483-31415) | 2117.69 (1890.2-2350.66) | 4.22 (3.03-5.43) |
| Belize | 24 (22-26) | 599.01 (542.99-658.74) | 58 (52-64) | 517.19 (466.47-570.12) | -0.76 (-0.96--0.56) |
| Benin | 1866 (1677-2080) | 2360.61 (2126.39-2626.04) | 4904 (4422-5406) | 2678.24 (2414.88-2947.98) | 0.24 (0.15-0.34) |
| Bermuda | 12 (11-14) | 456.31 (398.38-513.36) | 43 (36-50) | 657.58 (539.42-778.47) | 0.62 (0.4-0.84) |
| Bhutan | 243 (216-275) | 2966.68 (2639.08-3350.35) | 662 (590-750) | 2510.65 (2240.84-2839.76) | -0.78 (-0.93--0.62) |
| Bolivia (Plurinational State of) | 1210 (1031-1417) | 953.26 (815.93-1111.77) | 1390 (1224-1564) | 356.78 (314.93-400.53) | -4.08 (-4.56--3.6) |
| Bosnia and Herzegovina | 58 (47-70) | 39.15 (31.64-47.66) | 227 (192-266) | 69.22 (58.15-81.64) | 2.68 (2.45-2.92) |
| Botswana | 758 (654-872) | 3617.08 (3125.12-4160.61) | 1206 (1086-1348) | 2312.95 (2083.4-2581.83) | -1.65 (-1.92--1.38) |
| Brazil | 41433 (33972-49732) | 1158.59 (954.66-1385.76) | 93624 (80260-109706) | 797.08 (683.97-932.05) | -1.08 (-1.44--0.73) |
| Brunei Darussalam | 3 (2-3) | 76.75 (63.52-90) | 8 (7-9) | 63.97 (56.21-72.17) | -0.3 (-0.63-0.04) |
| Bulgaria | 122 (94-154) | 20.92 (16.14-26.47) | 185 (148-225) | 22.85 (18.26-27.84) | 0.67 (0.47-0.87) |
| Burkina Faso | 4917 (4437-5467) | 2960.41 (2674.39-3288.24) | 9396 (8493-10344) | 2756.05 (2496.81-3031.4) | -0.42 (-0.55--0.29) |
| Burundi | 1665 (1446-1904) | 1881.06 (1644.68-2145.23) | 4440 (3923-5002) | 2742.16 (2427.35-3084.75) | 1.17 (1.08-1.26) |
| Cabo Verde | 209 (186-236) | 2032.27 (1818.11-2280.02) | 226 (203-252) | 1347.26 (1207.07-1498.22) | -1.73 (-1.91--1.55) |
| Cambodia | 1882 (1665-2116) | 1152.67 (1020.93-1293.5) | 5715 (5087-6372) | 1201.85 (1071.55-1336.77) | -0.12 (-0.21--0.03) |
| Cameroon | 3836 (3433-4280) | 2499.45 (2240.16-2786.68) | 9478 (8479-10468) | 2220.14 (1987.61-2449.59) | -0.65 (-0.81--0.5) |
| Canada | 3182 (2445-3974) | 232.94 (175.57-294.84) | 3680 (3358-4005) | 95.77 (86.68-105.25) | -2.74 (-4.06--1.4) |
| Central African Republic | 580 (512-652) | 1601.13 (1413.88-1800.28) | 1701 (1536-1883) | 2720.27 (2457.68-3010.96) | 1.81 (1.76-1.86) |
| Chad | 3326 (2942-3745) | 2838.61 (2518.74-3191.73) | 6609 (5934-7330) | 3279.21 (2947.18-3629.23) | 0.43 (0.21-0.65) |
| Chile | 5821 (5198-6379) | 1306.31 (1166.17-1433.55) | 5711 (5207-6252) | 443.36 (403.17-485.24) | -3.83 (-4.69--2.96) |
| China | 132654 (104905-163935) | 403.85 (320.78-497.46) | 127846 (103926-154535) | 128.93 (104.44-155.94) | -3.82 (-3.92--3.72) |
| Colombia | 9171 (8346-9998) | 1297.29 (1181.7-1411.11) | 18536 (16725-20389) | 712.81 (639.53-786.45) | -2.77 (-3.16--2.38) |
| Comoros | 140 (122-159) | 1962.88 (1721.28-2231.02) | 523 (471-580) | 2860.43 (2582.83-3164.59) | 1.25 (1.02-1.49) |
| Congo | 713 (629-816) | 1853.86 (1637.13-2117.24) | 3084 (2769-3427) | 3605.28 (3240.5-3999.23) | 2.37 (2.23-2.5) |
| Cook Islands | 3 (3-4) | 696.87 (601.78-801.62) | 19 (17-22) | 1587.61 (1409.3-1793.48) | 2.8 (2.7-2.9) |
| Costa Rica | 2277 (2051-2534) | 2911.92 (2621.44-3237.61) | 5527 (4973-6124) | 2108.67 (1895.1-2335.55) | -1.34 (-1.64--1.05) |
| Croatia | 82 (69-96) | 30.97 (25.91-36.55) | 567 (496-647) | 108.15 (94.21-124.09) | 5.48 (5.01-5.95) |
| Cuba | 5126 (4649-5596) | 1092.79 (994.35-1191.79) | 8159 (7313-9061) | 914.35 (814.94-1019.56) | -1.69 (-2.07--1.31) |
| Cyprus | 314 (260-377) | 742.38 (613.16-891.99) | 2102 (1812-2413) | 1855.06 (1589.85-2130.15) | 4.29 (3.57-5.02) |
| Czechia | 179 (143-220) | 26 (20.75-31.94) | 5079 (4559-5648) | 397.92 (355.96-443.55) | 11.86 (10.63-13.11) |
| C么te d'Ivoire | 2555 (2279-2867) | 2086.68 (1862.76-2340.1) | 8150 (7408-9020) | 2259.77 (2053.39-2499.04) | 0.28 (0.06-0.5) |
| Democratic People's Republic of Korea | 1465 (1223-1731) | 255.58 (213.2-302.11) | 9234 (7644-10917) | 718.91 (586.51-860.64) | 4.64 (3.98-5.31) |
| Democratic Republic of the Congo | 7813 (6960-8760) | 1470.43 (1310.45-1648.37) | 23752 (21545-26260) | 2043.34 (1855.31-2257.92) | 0.99 (0.82-1.16) |
| Denmark | 3751 (3285-4274) | 832.93 (720.97-956.83) | 15071 (13331-16972) | 2168.45 (1907.63-2455.02) | 4.43 (3.39-5.48) |
| Djibouti | 85 (74-97) | 2140.46 (1861.98-2442.09) | 527 (466-595) | 2662.36 (2353.32-2999.41) | 0.51 (0.2-0.82) |
| Dominica | 12 (10-13) | 403.62 (359.51-455.84) | 10 (9-11) | 273.78 (240.4-308.13) | -1.63 (-1.73--1.53) |
| Dominican Republic | 701 (627-786) | 477.95 (428.82-535.19) | 1981 (1761-2231) | 457.23 (406.63-514.74) | -0.69 (-1.02--0.36) |
| Ecuador | 6862 (6167-7616) | 3157.39 (2845.8-3496.25) | 4823 (4284-5398) | 642.05 (571.34-717.77) | -6.55 (-7.23--5.86) |
| Egypt | 2469 (2043-2944) | 268.55 (222.58-319.76) | 7458 (6382-8566) | 339.96 (290.82-390.49) | 0.2 (-0.09-0.49) |
| El Salvador | 2866 (2563-3189) | 2222.2 (1990.01-2468.83) | 3103 (2719-3534) | 1032.54 (907.58-1171.4) | -3.4 (-3.91--2.88) |
| Equatorial Guinea | 109 (96-123) | 1646.42 (1450.96-1866.66) | 507 (460-564) | 3034.23 (2753.16-3371.73) | 1.98 (1.61-2.35) |
| Eritrea | 721 (626-824) | 2485.66 (2158.06-2840.13) | 3525 (3134-3978) | 4192.75 (3729.84-4728.88) | 1.46 (1.35-1.58) |
| Estonia | 54 (39-71) | 64.3 (45.67-85.45) | 415 (333-506) | 294.95 (230.97-361.64) | 6.01 (5.28-6.75) |
| Eswatini | 335 (289-391) | 3330.2 (2874.53-3882.83) | 617 (553-690) | 3088.59 (2771.64-3449.29) | -0.33 (-0.5--0.16) |
| Ethiopia | 21682 (17823-26041) | 3157.32 (2600.48-3786.78) | 56583 (48434-66021) | 3520.73 (3026.84-4098.23) | 0.13 (-0.04-0.29) |
| Fiji | 224 (198-257) | 1896.97 (1680.83-2172.62) | 437 (391-485) | 1530.75 (1371.61-1696.03) | -0.62 (-0.82--0.42) |
| Finland | 2641 (2273-3072) | 710.77 (605.92-833.41) | 2679 (2273-3128) | 373.1 (310.6-443.02) | -1.7 (-2.7--0.68) |
| France | 40699 (35640-46208) | 930.93 (806.46-1062.45) | 93266 (81070-106198) | 1221.12 (1045.68-1409.88) | 1.21 (0.24-2.2) |
| Gabon | 447 (393-504) | 1958.78 (1730.47-2207.86) | 1038 (929-1163) | 2790.9 (2500.19-3122.17) | 1.05 (0.96-1.14) |
| Gambia | 269 (238-305) | 2115.63 (1873.97-2392.08) | 668 (598-750) | 1879.18 (1683.81-2107.79) | -0.66 (-0.76--0.56) |
| Georgia | 255 (204-313) | 107.83 (84.32-133.94) | 281 (238-323) | 102.94 (86.61-119.33) | 0.93 (0.27-1.59) |
| Germany | 32005 (26800-37837) | 524.31 (430.19-632.76) | 145821 (132578-161084) | 1316.81 (1193.83-1457.92) | 3.96 (2.67-5.25) |
| Ghana | 4125 (3643-4659) | 1897.93 (1679.45-2142.34) | 11746 (10655-12961) | 1971.82 (1791.25-2175.23) | -0.54 (-0.74--0.34) |
| Greece | 3938 (3069-4892) | 543.99 (421.54-679.46) | 4277 (3501-5115) | 366.73 (292.71-449.37) | -0.51 (-1.18-0.15) |
| Greenland | 1 (1-1) | 77.86 (65.97-90.92) | 1 (1-1) | 40.46 (35.3-46.58) | -1.86 (-2.49--1.22) |
| Grenada | 12 (11-14) | 362.35 (323.24-403.19) | 16 (14-18) | 344.01 (304.15-384.26) | -0.87 (-1.09--0.65) |
| Guam | 23 (19-26) | 857.1 (731.21-1001.94) | 121 (108-136) | 1233.88 (1100.95-1384.25) | 1.39 (1.12-1.66) |
| Guatemala | 4845 (4516-5163) | 3790.07 (3537.43-4035.05) | 8119 (7516-8687) | 1681.19 (1558.24-1796.76) | -3.19 (-3.47--2.92) |
| Guinea | 3215 (2843-3622) | 2343.56 (2076.63-2635.8) | 5372 (4812-6010) | 2568.97 (2305.01-2866.3) | -0.08 (-0.23-0.07) |
| Guinea-Bissau | 299 (266-340) | 2096.53 (1865.69-2380.55) | 597 (537-662) | 2592.09 (2335.75-2871.81) | 0.48 (0.34-0.63) |
| Guyana | 97 (89-105) | 667.52 (614.1-722.62) | 146 (135-157) | 584.81 (542.7-629.7) | -1.39 (-1.69--1.09) |
| Haiti | 425 (368-487) | 358.1 (310.56-410.17) | 1050 (935-1181) | 413.23 (368.61-464.4) | 0.69 (0.2-1.19) |
| Honduras | 1768 (1586-1986) | 2154.59 (1936.52-2416.33) | 4260 (3781-4800) | 1609.11 (1431.57-1807.96) | -1.47 (-1.77--1.16) |
| Hungary | 221 (173-278) | 31.22 (24.41-39.58) | 3960 (3530-4448) | 357.06 (318.34-400.76) | 10.26 (9.39-11.14) |
| Iceland | 104 (88-122) | 712.18 (592.61-846.37) | 348 (305-395) | 1126.91 (974.02-1290.52) | 2.16 (1.18-3.15) |
| India | 763275 (616928-915435) | 4727.54 (3836.32-5659.91) | 3280966 (2861330-3667613) | 6509.88 (5691-7266.68) | 0.83 (0.71-0.96) |
| Indonesia | 154098 (122858-188745) | 4479.52 (3586.14-5470.63) | 273855 (231703-319935) | 3063.91 (2594.53-3573.23) | -1.3 (-1.48--1.12) |
| Iran (Islamic Republic of) | 4230 (3401-5112) | 447.81 (360.21-541.18) | 11699 (9685-13839) | 379.21 (313.33-448.54) | -0.66 (-0.78--0.53) |
| Iraq | 932 (812-1064) | 300.06 (261.51-341.23) | 3134 (2730-3545) | 371.71 (322.68-421.54) | 1.19 (0.96-1.43) |
| Ireland | 825 (655-989) | 414.05 (322.57-502.48) | 4073 (3547-4635) | 982 (844.09-1130.52) | 4.56 (3.49-5.65) |
| Israel | 1672 (1459-1900) | 670.38 (578.69-768.04) | 14105 (12778-15735) | 2064.49 (1862.74-2305.62) | 4.63 (3.4-5.87) |
| Italy | 14412 (10949-18329) | 326.61 (246.81-416.51) | 40395 (34449-46865) | 473.46 (401.14-551.49) | 2.25 (1.99-2.52) |
| Jamaica | 697 (635-766) | 822.58 (751.42-899.72) | 500 (448-554) | 370.45 (330.35-412.88) | -2.35 (-3.02--1.68) |
| Japan | 59829 (47473-73272) | 732.42 (578.93-900.16) | 186984 (161768-217418) | 878.18 (749.58-1025.93) | 0.92 (0.73-1.12) |
| Jordan | 103 (85-124) | 230.96 (190.56-277.44) | 1224 (1043-1427) | 462.52 (392.9-540.16) | 2.87 (2.49-3.25) |
| Kazakhstan | 306 (256-361) | 70.51 (58.6-83.64) | 534 (410-668) | 78.8 (59.83-99.12) | 0.53 (0.04-1.03) |
| Kenya | 15040 (12293-18179) | 4909.85 (4028.51-5907.92) | 41237 (35577-47699) | 4992.21 (4313.91-5769.75) | -0.06 (-0.18-0.07) |
| Kiribati | 51 (46-56) | 3808.59 (3432.48-4196.97) | 84 (76-93) | 3600.86 (3249.15-3976.23) | -0.49 (-0.58--0.4) |
| Kuwait | 44 (37-52) | 256.12 (215.38-300.18) | 305 (245-367) | 360.4 (286.97-438) | 2.61 (2.18-3.05) |
| Kyrgyzstan | 60 (49-71) | 57.01 (46.32-68.08) | 106 (87-129) | 63.75 (52.16-77.94) | 0.76 (0.57-0.95) |
| Lao People's Democratic Republic | 669 (577-767) | 855.37 (740.61-979.07) | 1796 (1586-2017) | 1084.7 (960.65-1214.77) | 0.92 (0.7-1.15) |
| Latvia | 89 (65-118) | 61.08 (44.01-81.73) | 283 (225-353) | 141.68 (111.04-178.36) | 3.66 (3.17-4.16) |
| Lebanon | 292 (252-341) | 345.03 (297.6-401.13) | 2300 (1991-2652) | 797.5 (687.88-922.94) | 3.08 (2.97-3.19) |
| Lesotho | 1274 (1107-1464) | 3716.31 (3236.29-4254.89) | 1396 (1234-1575) | 3337.5 (2953.97-3761.33) | -0.59 (-0.83--0.35) |
| Liberia | 967 (862-1069) | 2020.9 (1807.23-2229.52) | 2474 (2256-2731) | 3609.62 (3294.75-3979.51) | 1.79 (1.55-2.03) |
| Libya | 305 (256-357) | 423.93 (356.99-494.99) | 914 (799-1045) | 508.76 (442.5-583.38) | 0.77 (0.63-0.92) |
| Lithuania | 150 (116-188) | 79.1 (60.15-100.02) | 1179 (1008-1378) | 394.06 (332.05-465.64) | 6.25 (5.18-7.33) |
| Luxembourg | 208 (178-241) | 752.72 (636.62-875.58) | 720 (644-817) | 1292.44 (1147.59-1470.77) | 2.33 (1.26-3.4) |
| Madagascar | 2960 (2679-3264) | 1614.43 (1462.36-1777.54) | 8881 (8046-9844) | 2601.75 (2358.05-2882.54) | 1.5 (1.45-1.54) |
| Malawi | 3375 (2989-3803) | 2386.66 (2116.24-2685.87) | 7823 (7126-8624) | 2920.81 (2663.48-3214.42) | 0.44 (0.32-0.55) |
| Malaysia | 2533 (2233-2849) | 736.28 (651.61-824.86) | 27175 (24479-29989) | 2275.44 (2052.05-2506.42) | 3.8 (3.63-3.97) |
| Maldives | 26 (23-30) | 930.01 (816.43-1066.15) | 182 (163-203) | 1596.58 (1431.19-1775.08) | 1.97 (1.76-2.18) |
| Mali | 5139 (4668-5708) | 3593.44 (3266.71-3986.73) | 13370 (12088-14724) | 4285.23 (3881.16-4715.04) | 0.35 (0.22-0.49) |
| Malta | 73 (58-89) | 381.07 (296.13-471.99) | 266 (222-313) | 513.2 (419.18-614.81) | 1.44 (0.73-2.15) |
| Marshall Islands | 9 (8-11) | 1492.37 (1296.4-1734.77) | 9 (8-10) | 795.69 (708.36-888.9) | -2.31 (-2.48--2.14) |
| Mauritania | 1111 (988-1247) | 2704.71 (2409.32-3024.8) | 3449 (3121-3816) | 4133.14 (3744.31-4568.62) | 0.9 (0.76-1.03) |
| Mauritius | 218 (197-240) | 757.54 (686.3-833.7) | 408 (366-455) | 491.54 (440.24-547.7) | -1.29 (-1.63--0.95) |
| Mexico | 43688 (34663-53723) | 2517.62 (2007.26-3077.88) | 36573 (31513-42290) | 661.57 (570.87-760.89) | -4.4 (-4.78--4.02) |
| Micronesia (Federated States of) | 22 (19-26) | 1108.83 (971.14-1283.16) | 19 (17-21) | 791.19 (711.46-882.5) | -1.16 (-1.33--1) |
| Monaco | 16 (13-20) | 443.2 (341.94-555.69) | 26 (22-30) | 509.42 (419.32-609.62) | 0.93 (0.2-1.67) |
| Mongolia | 12 (10-14) | 30.31 (24.9-36.07) | 16 (12-19) | 22.27 (17.59-26.83) | -0.8 (-1.23--0.37) |
| Montenegro | 10 (7-12) | 36.76 (28.07-46.72) | 20 (16-26) | 41.19 (31.88-52.3) | 0.79 (0.62-0.95) |
| Morocco | 1721 (1486-2012) | 294.36 (254.57-342.85) | 4218 (3681-4793) | 300.91 (262.37-342.43) | -0.15 (-0.28--0.02) |
| Mozambique | 3403 (3010-3855) | 1604.94 (1420.78-1815.15) | 7265 (6548-8019) | 1907.24 (1720.76-2104.29) | 0.25 (0.14-0.35) |
| Myanmar | 7889 (6828-9022) | 910.58 (789.22-1039.18) | 18473 (16709-20440) | 952.1 (862.03-1052.16) | -0.14 (-0.23--0.05) |
| Namibia | 796 (691-917) | 3193.06 (2770.15-3673.89) | 1813 (1620-2029) | 3602.9 (3222.72-4029.87) | 0.39 (0.21-0.57) |
| Nauru | 1 (1-1) | 699.39 (607.18-802.35) | 1 (1-2) | 678.26 (600.84-759.6) | -0.46 (-0.7--0.21) |
| Nepal | 6772 (5969-7701) | 2043.12 (1802.89-2319.43) | 16966 (15284-18984) | 1737.96 (1570.53-1937.98) | -0.16 (-0.42-0.11) |
| Netherlands | 6266 (5089-7508) | 635.3 (508.11-772.84) | 33389 (28905-38009) | 1718.39 (1461.08-1981.17) | 3.91 (2.68-5.16) |
| New Zealand | 1406 (1096-1770) | 735.34 (566.96-930.34) | 4336 (3590-5130) | 944.64 (778.64-1121.84) | 2.05 (1.55-2.56) |
| Nicaragua | 1099 (966-1244) | 1780.83 (1565.75-2012.4) | 1288 (1160-1427) | 621.59 (559.97-689.1) | -4.19 (-4.59--3.8) |
| Niger | 2334 (2084-2620) | 2484.65 (2220.69-2786.38) | 8364 (7620-9152) | 2957.8 (2696.6-3231.8) | 0.65 (0.46-0.84) |
| Nigeria | 87803 (70771-108050) | 5106.93 (4135.51-6252.21) | 75489 (64778-87155) | 2453 (2111.84-2824.31) | -2.83 (-2.98--2.68) |
| Niue | 1 (1-1) | 1159.82 (1004.9-1338.85) | 1 (1-1) | 1186.2 (1043.2-1347.47) | 0.23 (0.1-0.36) |
| North Macedonia | 40 (33-48) | 53.27 (44.14-63.76) | 70 (57-85) | 43.19 (35.07-52.89) | -0.45 (-0.62--0.29) |
| Northern Mariana Islands | 5 (5-6) | 1243.43 (1082.49-1413.6) | 33 (30-37) | 1829.84 (1637.84-2038.91) | 0.88 (0.71-1.06) |
| Norway | 3955 (3169-4893) | 990.57 (788.78-1226.89) | 18496 (15713-21462) | 3234.01 (2732.78-3763.93) | 3.91 (3.49-4.33) |
| Oman | 190 (164-220) | 806.92 (697.38-930.58) | 1104 (969-1247) | 1849.89 (1624.92-2088.18) | 2.72 (2.29-3.14) |
| Pakistan | 85467 (68960-104679) | 3658.58 (2966.53-4461.46) | 119385 (100773-140082) | 2706.46 (2292.52-3167.32) | -1.14 (-1.35--0.93) |
| Palau | 4 (3-4) | 967.9 (839.79-1097.84) | 10 (8-11) | 1162.87 (1021.47-1336.71) | 0.39 (0.29-0.49) |
| Palestine | 91 (78-106) | 258.06 (220.59-300.14) | 302 (264-345) | 335.62 (292.54-383.95) | 1.26 (1.03-1.49) |
| Panama | 1141 (1030-1255) | 1738.39 (1572.3-1910.19) | 2841 (2582-3128) | 1358.31 (1235.7-1493.51) | -1.09 (-1.44--0.75) |
| Papua New Guinea | 1451 (1292-1619) | 2457.29 (2189.21-2740.8) | 3689 (3335-4063) | 2330.36 (2108.16-2565.53) | -0.5 (-0.59--0.41) |
| Paraguay | 4759 (4231-5373) | 4995.93 (4448.5-5633) | 1271 (1143-1406) | 501.62 (450.79-554.55) | -8.83 (-9.63--8.03) |
| Peru | 12814 (11302-14495) | 2611.41 (2310.32-2943.03) | 16321 (14696-18143) | 1076.88 (970.43-1196.62) | -3.66 (-4.06--3.26) |
| Philippines | 13728 (11259-16616) | 1277.02 (1051.94-1537.68) | 19170 (16215-22197) | 615.1 (521.69-710.66) | -2.75 (-3.01--2.5) |
| Poland | 413 (299-558) | 19.72 (14.16-26.64) | 1178 (990-1377) | 29.41 (24.61-34.5) | 2.57 (1.17-3.99) |
| Portugal | 1740 (1434-2073) | 255.08 (207.36-306.17) | 9044 (8163-10172) | 634.2 (565.46-718.87) | 4.66 (3.88-5.45) |
| Puerto Rico | 385 (316-456) | 234.42 (187.75-282.56) | 4035 (3599-4480) | 1030.86 (914.66-1154.45) | 5.34 (4.63-6.05) |
| Qatar | 6 (5-7) | 244.76 (208.02-283.46) | 209 (184-237) | 1151.37 (1011.81-1305.81) | 5.55 (5.31-5.78) |
| Republic of Korea | 1929 (1701-2173) | 172.28 (151.66-194.31) | 8924 (7988-9976) | 190.76 (169.51-214.36) | 1.1 (0.69-1.51) |
| Republic of Moldova | 106 (75-141) | 60.86 (43.02-81.09) | 297 (235-367) | 106.53 (83.36-132.97) | 2.41 (2.07-2.76) |
| Romania | 183 (143-228) | 14.85 (11.61-18.53) | 800 (690-917) | 38.15 (32.67-44.08) | 3.77 (3.5-4.05) |
| Russian Federation | 7534 (5944-9256) | 111.47 (86.95-137.74) | 12763 (10019-15717) | 118.42 (91.68-146.83) | 0.13 (-0.13-0.39) |
| Rwanda | 1386 (1206-1576) | 1361.46 (1185.98-1546.47) | 5462 (4872-6139) | 2428.47 (2168.78-2725.96) | 1.98 (1.77-2.19) |
| Saint Kitts and Nevis | 17 (15-18) | 839.89 (755.02-922.9) | 26 (24-29) | 977.58 (877.82-1069.95) | 0.14 (0.03-0.24) |
| Saint Lucia | 25 (22-27) | 605.75 (546.66-670.6) | 55 (49-61) | 521.34 (464.97-580.44) | -1.09 (-1.29--0.89) |
| Saint Vincent and the Grenadines | 24 (21-26) | 701.18 (633.91-774.7) | 32 (29-36) | 505.41 (456.18-558.84) | -1.39 (-1.61--1.18) |
| Samoa | 24 (21-28) | 727.73 (629.9-835.68) | 51 (46-59) | 905.5 (805.45-1030.1) | 0.55 (0.47-0.62) |
| San Marino | 9 (7-11) | 508.57 (395.98-628.89) | 21 (18-25) | 556.85 (461.28-667.36) | 0.81 (0.17-1.46) |
| Sao Tome and Principe | 36 (32-40) | 1386.07 (1237.77-1536.11) | 35 (31-39) | 912.71 (806.15-1025.58) | -1.77 (-2.12--1.41) |
| Saudi Arabia | 1619 (1399-1867) | 782.58 (678.64-900.71) | 6577 (5859-7355) | 1283.41 (1143.34-1435.19) | 2.27 (1.85-2.68) |
| Senegal | 3725 (3324-4186) | 3006.13 (2686.07-3370.34) | 8562 (7756-9398) | 2977.61 (2702.12-3266.56) | -0.46 (-0.6--0.32) |
| Serbia | 155 (125-192) | 33.37 (26.73-41.23) | 1569 (1361-1782) | 171.56 (148.01-195.3) | 7.05 (6.4-7.71) |
| Seychelles | 23 (20-27) | 891.31 (772.97-1022.67) | 52 (46-59) | 1131.5 (1001.92-1263.75) | 0.92 (0.73-1.11) |
| Sierra Leone | 2364 (2127-2638) | 2830.75 (2549.47-3149.59) | 4447 (4015-4937) | 3272.74 (2959.32-3627.01) | 0.09 (-0.02-0.21) |
| Singapore | 77 (69-84) | 85.85 (77.63-94.54) | 170 (153-187) | 40.76 (36.53-44.81) | -1.95 (-2.64--1.26) |
| Slovakia | 57 (47-69) | 19.73 (16.07-23.94) | 263 (222-310) | 50.87 (42.83-59.98) | 3.91 (3.68-4.13) |
| Slovenia | 41 (33-50) | 35.57 (28.45-43.26) | 186 (156-221) | 74.28 (61.65-88.55) | 2.91 (2.7-3.13) |
| Solomon Islands | 87 (76-99) | 1925.66 (1692.9-2181.53) | 198 (179-219) | 1740.87 (1580.76-1923.36) | -0.5 (-0.58--0.43) |
| Somalia | 1171 (1023-1332) | 1824.98 (1594.82-2071.51) | 4306 (3839-4824) | 2226.94 (1985.01-2494.84) | 0.36 (0.23-0.49) |
| South Africa | 24471 (19814-29970) | 3015.5 (2439.99-3680.59) | 55655 (47822-64714) | 3018.59 (2597.31-3501.05) | 0.3 (0.11-0.5) |
| South Sudan | 2814 (2427-3220) | 2679.06 (2315.12-3058.72) | 6302 (5657-7022) | 5252.24 (4720.78-5845.23) | 2.17 (2.09-2.25) |
| Spain | 15786 (13350-18163) | 565.37 (471.79-657.16) | 56648 (51084-62782) | 1038.13 (928.15-1156.04) | 2.75 (1.98-3.53) |
| Sri Lanka | 6636 (5769-7605) | 1600.77 (1397.69-1826.54) | 18238 (16256-20661) | 1436.65 (1282.27-1624.82) | -0.42 (-0.56--0.29) |
| Sudan | 1685 (1449-1953) | 436.76 (377.21-503.75) | 7559 (6711-8503) | 1056.62 (940.47-1184.85) | 2.74 (2.43-3.05) |
| Suriname | 72 (65-80) | 732.12 (666.35-815.3) | 165 (148-183) | 629.95 (566.16-698.11) | -0.58 (-0.66--0.49) |
| Sweden | 6460 (5200-7950) | 735.66 (587.93-911.29) | 34891 (29388-40718) | 2685.24 (2244.6-3150.97) | 5.64 (4.95-6.33) |
| Switzerland | 4797 (4182-5448) | 865.81 (746.03-994.27) | 14396 (12902-15972) | 1432.25 (1268.4-1606.62) | 2.71 (1.94-3.48) |
| Syrian Arab Republic | 323 (273-381) | 168.74 (142.04-198.61) | 1927 (1590-2294) | 379.06 (312.01-452.68) | 2.76 (2.58-2.94) |
| Taiwan (Province of China) | 12055 (10635-13387) | 1817.38 (1601.4-2020.1) | 57466 (50182-65041) | 2690.85 (2327.65-3067.35) | 3.6 (2.03-5.2) |
| Tajikistan | 157 (139-174) | 164.65 (146.17-183.07) | 348 (311-389) | 192.23 (171.63-214.78) | 0.24 (0.12-0.36) |
| Thailand | 18236 (15947-20829) | 1419.72 (1245.09-1617.39) | 117734 (106148-129934) | 2403.47 (2171.62-2647.69) | 2 (1.78-2.23) |
| Timor-Leste | 96 (83-110) | 1199.11 (1037.85-1380.63) | 549 (478-630) | 1457.21 (1274.53-1668.16) | 0.8 (0.67-0.94) |
| Togo | 1201 (1069-1346) | 2807.25 (2501.04-3142.74) | 3842 (3472-4226) | 3056.38 (2765.56-3358.09) | 0.09 (-0.04-0.23) |
| Tokelau | 1 (1-1) | 1334.07 (1137.85-1553.78) | 1 (1-1) | 1409 (1239.18-1606.1) | 0.02 (-0.17-0.22) |
| Tonga | 19 (17-22) | 867.54 (764.21-984.65) | 21 (19-24) | 613.02 (539.81-696.97) | -1.21 (-1.44--0.99) |
| Trinidad and Tobago | 148 (134-164) | 391.85 (355.17-432.69) | 320 (284-360) | 351.36 (310.54-395.74) | -0.35 (-0.52--0.17) |
| Tunisia | 670 (578-777) | 327.95 (283.33-379.61) | 3799 (3291-4351) | 652.68 (563.58-749.05) | 2.62 (2.4-2.85) |
| Turkmenistan | 76 (68-84) | 115.45 (102.42-128.34) | 85 (69-103) | 61.63 (49.21-74.72) | -2.77 (-3.06--2.48) |
| Tuvalu | 3 (2-3) | 1002.29 (863.74-1157.33) | 4 (4-5) | 952.67 (854.06-1068.03) | -0.35 (-0.47--0.23) |
| T眉rkiye | 2064 (1747-2404) | 164.21 (138.71-191.55) | 22275 (19667-25243) | 529.5 (465.11-603.21) | 4.63 (4.21-5.05) |
| Uganda | 5664 (4945-6440) | 2355.47 (2063.43-2670.96) | 15047 (13571-16769) | 2990.32 (2699.07-3324.02) | 0.85 (0.66-1.05) |
| Ukraine | 1322 (937-1746) | 45.63 (31.93-60.85) | 3456 (2610-4424) | 100.25 (74.25-129.7) | 2.85 (2.5-3.2) |
| United Arab Emirates | 123 (104-143) | 1126.34 (961.89-1311.31) | 1247 (1099-1392) | 1631.63 (1431.86-1825.65) | 1.35 (1.26-1.44) |
| United Kingdom | 22173 (17450-27247) | 476.12 (371.01-587.06) | 42937 (37231-49043) | 592.09 (508.04-680.78) | 0.99 (-0.69-2.69) |
| United Republic of Tanzania | 12516 (11369-13871) | 3041.53 (2768.59-3361.96) | 21789 (19780-24207) | 2396.7 (2179.71-2660.34) | -1.31 (-1.51--1.1) |
| United States of America | 29099 (21938-37038) | 182.71 (135-235.12) | 23418 (20311-26792) | 73.63 (63.62-84.24) | -4.49 (-6.03--2.92) |
| United States Virgin Islands | 8 (7-10) | 258.95 (221.76-298.68) | 27 (23-31) | 291.94 (248.33-339.09) | 0.13 (-0.01-0.28) |
| Uruguay | 2069 (1860-2285) | 1046.74 (939.23-1156.83) | 1466 (1314-1606) | 502.77 (449.24-552) | -1.99 (-3.01--0.96) |
| Uzbekistan | 272 (235-310) | 68.84 (58.76-78.98) | 308 (240-383) | 34.29 (26.63-42.79) | -2.25 (-2.48--2.02) |
| Vanuatu | 48 (42-55) | 2218.28 (1940.08-2527.24) | 128 (114-143) | 2082.06 (1851.8-2322.85) | -0.4 (-0.47--0.33) |
| Venezuela (Bolivarian Republic of) | 5891 (5370-6487) | 1496.39 (1364.75-1646.93) | 19228 (17428-21048) | 1403.19 (1271.54-1536.66) | -1.06 (-1.33--0.78) |
| Viet Nam | 17206 (14811-19972) | 987.65 (851.84-1143.68) | 44100 (39073-49738) | 1116.06 (988.72-1258.2) | 0.5 (0.29-0.7) |
| Yemen | 971 (843-1117) | 549.29 (477.7-630.72) | 3416 (3039-3831) | 665.37 (592.84-743.89) | 0.58 (0.32-0.83) |
| Zambia | 1975 (1758-2218) | 2037.91 (1818.82-2282.85) | 5477 (4976-6017) | 2455.01 (2232.67-2693.39) | 0.37 (0.29-0.46) |
| Zimbabwe | 3937 (3540-4403) | 2528.2 (2276.05-2823.77) | 5025 (4515-5574) | 2036.93 (1831.18-2258.64) | -1.32 (-1.59--1.04) |

eTable 2. Mortality of diarrhea disease in the elderly at the global, sex, age-group levels, regional, ,and national levels from 1990 to 2021.

| **Location** | **Rate per 100 000(95% UI)** | | | | |
| --- | --- | --- | --- | --- | --- |
|  | **1990** | | **2021** | | **1990-2021** |
|  | **Deaths cases** | **Deaths rate** | **Deaths cases** | **Deaths rate** | **EAPC** |
| Global | 752336 (484580-1141072) | 413.47 (264.96-632.11) | 569830 (347143-880194) | 126.76 (76.8-196.98) | -3.67 (-3.81--3.53) |
| Sex |  |  |  |  |  |
| Female | 409499 (221273-693561) | 387.37 (205.87-663.84) | 329749 (149858-606145) | 131.66 (59.05-243) | -3.33 (-3.43--3.23) |
| Male | 342837 (185075-571338) | 449.23 (241.52-753.23) | 240080 (125119-425964) | 120.57 (62.02-214.66) | -4.1 (-4.3--3.91) |
| Age |  |  |  |  |  |
| <65 years | 0 (0-0) | 0 (0-0) | 0 (0-0) | 0 (0-0) | 0 (0-0) |
| 65-69 years | 130697 (79864-214158) | 105.73 (64.61-173.25) | 71755 (41081-118143) | 26.01 (14.89-42.83) | -4.46 (-4.7--4.21) |
| 70-74 years | 162993 (100751-256040) | 192.52 (119-302.43) | 106180 (58974-172904) | 51.58 (28.65-84) | -3.99 (-4.15--3.83) |
| 75-79 years | 161055 (102324-248463) | 261.64 (166.23-403.64) | 109945 (64548-174684) | 83.37 (48.94-132.45) | -3.76 (-3.9--3.62) |
| 80-84 years | 154443 (102370-225811) | 436.58 (289.38-638.32) | 122428 (72154-191029) | 139.79 (82.38-218.11) | -3.52 (-3.59--3.45) |
| 85-89 years | 93798 (62989-132944) | 620.72 (416.84-879.78) | 92580 (59998-146386) | 202.48 (131.22-320.17) | -3.64 (-3.85--3.44) |
| 90-94 years | 37321 (24571-52385) | 870.93 (573.39-1222.48) | 48767 (31163-74430) | 272.61 (174.2-416.06) | -3.66 (-3.98--3.34) |
| 95+ years | 12028 (7821-16785) | 1181.43 (768.17-1648.66) | 18174 (11480-27007) | 333.45 (210.63-495.51) | -3.64 (-4.06--3.21) |
| SDI region |  |  |  |  |  |
| High-middle SDI | 7555 (4243-10552) | 16 (8.97-22.38) | 12035 (8368-15029) | 10.94 (7.53-13.75) | -0.86 (-1.34--0.37) |
| High SDI | 4389 (3778-4918) | 6.75 (5.81-7.56) | 24291 (20037-26847) | 18.13 (15.1-19.99) | 4.67 (3.56-5.8) |
| Low-middle SDI | 441272 (295040-674407) | 1811.42 (1207.23-2783.34) | 293494 (176812-480335) | 447.55 (268.77-734.66) | -4.5 (-4.63--4.38) |
| Low SDI | 167905 (108745-251834) | 1922.74 (1241.27-2898.76) | 137472 (81010-226923) | 672.63 (395.66-1112.04) | -3.32 (-3.54--3.11) |
| Middle SDI | 130968 (69223-205366) | 305.08 (160.98-479.65) | 102319 (54311-153283) | 77.71 (41.19-116.83) | -4.32 (-4.42--4.22) |
| GBD region |  |  |  |  |  |
| Advanced Health System | 5492 (4770-6143) | 5.91 (5.13-6.61) | 29871 (24635-33020) | 16.48 (13.71-18.17) | 4.71 (3.81-5.61) |
| Africa | 103186 (55069-165076) | 908.04 (482.94-1455.27) | 83593 (43507-128368) | 332.04 (172.65-509.52) | -3.29 (-3.52--3.06) |
| African Region | 99966 (53043-159071) | 1111.14 (587.64-1772.22) | 80117 (41784-123703) | 408.38 (212.89-630.19) | -3.29 (-3.53--3.05) |
| America | 15173 (13695-16607) | 46.87 (42.3-51.34) | 22029 (18936-24265) | 29.24 (25.28-32.24) | -0.52 (-0.98--0.05) |
| Andean Latin America | 1119 (740-1486) | 120.9 (79.82-160.94) | 746 (470-1250) | 24.97 (15.61-42.17) | -5.36 (-5.71--5.01) |
| Asia | 630554 (410688-969601) | 748.47 (486.4-1157.97) | 448761 (267565-717836) | 174.78 (103.85-281.35) | -4.53 (-4.66--4.4) |
| Australasia | 113 (100-126) | 8.16 (7.22-9.1) | 272 (221-307) | 7.79 (6.35-8.78) | 2 (0.82-3.2) |
| Basic Health System | 107912 (53911-174101) | 171.17 (85.59-276.74) | 61715 (33732-82837) | 32.93 (18.03-44.3) | -5.03 (-5.26--4.79) |
| Caribbean | 1167 (817-1635) | 90.23 (62.34-128.21) | 1100 (706-1660) | 39.53 (25.03-60.25) | -3.08 (-3.4--2.76) |
| Central Africa | 10682 (6222-16720) | 1001.79 (581.22-1568.51) | 9729 (4945-15533) | 425.54 (216.02-681.51) | -2.76 (-3.19--2.33) |
| Central Asia | 133 (114-156) | 6.91 (5.92-8.04) | 42 (32-57) | 1.34 (1.01-1.82) | -6.95 (-7.51--6.38) |
| Central Europe | 189 (165-224) | 2.55 (2.23-3.03) | 2144 (1879-2390) | 15.54 (13.66-17.34) | 7.83 (6.01-9.67) |
| Central Latin America | 7872 (7324-8442) | 214.7 (199.83-230.05) | 5959 (5208-6795) | 48.08 (42.03-54.86) | -4.51 (-5.15--3.85) |
| Central Sub-Saharan Africa | 7368 (4450-11868) | 942.8 (568.21-1518.6) | 6478 (3309-10761) | 358.13 (182.43-595.56) | -3.07 (-3.67--2.47) |
| Commonwealth High Income | 347 (312-374) | 3.77 (3.41-4.06) | 2516 (2107-2770) | 14.39 (12.09-15.85) | 5.71 (3.52-7.96) |
| Commonwealth Low Income | 46828 (26165-66975) | 1268.68 (705.06-1826.99) | 38151 (22909-70220) | 369.5 (221.03-681.88) | -4.21 (-4.33--4.09) |
| Commonwealth Middle Income | 533319 (364211-811134) | 2208.77 (1503.79-3377.37) | 396076 (233791-652534) | 557.9 (328.7-922.5) | -4.42 (-4.58--4.27) |
| East Asia | 9062 (3903-13787) | 24.66 (10.63-37.5) | 2680 (1496-5045) | 2.3 (1.28-4.34) | -7.9 (-8.27--7.52) |
| East Asia & Pacific - WB | 97804 (42882-167833) | 168.52 (74.06-289.72) | 51785 (25585-72509) | 29.45 (14.53-41.29) | -5.36 (-5.59--5.13) |
| Eastern Africa | 33304 (15396-55597) | 1221.77 (561.81-2043.47) | 30073 (14506-44618) | 491.26 (237.39-731.02) | -3.07 (-3.22--2.91) |
| Eastern Europe | 258 (245-267) | 2.02 (1.92-2.09) | 280 (256-302) | 1.5 (1.37-1.62) | -2.88 (-3.88--1.86) |
| Eastern Mediterranean Region | 34621 (16035-66027) | 457.6 (211.9-874.87) | 20016 (11144-36082) | 112.87 (62.64-203.21) | -4.68 (-4.8--4.55) |
| Eastern Sub-Saharan Africa | 40019 (18892-65847) | 1396.38 (656.77-2303.88) | 35589 (17175-52644) | 558.35 (270.01-828.17) | -3.11 (-3.27--2.94) |
| Europe | 3136 (2744-3556) | 5.37 (4.71-6.1) | 15143 (12546-16789) | 14.83 (12.35-16.42) | 4.36 (3.71-5.02) |
| Europe & Central Asia - WB | 3227 (2829-3650) | 5.42 (4.76-6.14) | 15148 (12553-16795) | 14.54 (12.11-16.1) | 4.22 (3.58-4.86) |
| European Region | 3266 (2862-3698) | 5.45 (4.79-6.18) | 15475 (12812-17147) | 14.71 (12.24-16.28) | 4.24 (3.6-4.88) |
| High-income Asia Pacific | 1204 (949-1466) | 11.3 (8.88-13.8) | 4543 (3408-5648) | 13.89 (10.44-17.4) | 1.3 (1-1.6) |
| High-income North America | 554 (478-599) | 2.57 (2.24-2.77) | 9007 (7513-9821) | 22.63 (19.07-24.61) | 8.95 (6.75-11.2) |
| Latin America & Caribbean - WB | 14654 (13188-16078) | 119.32 (107.38-130.97) | 13095 (11262-14899) | 36.04 (31.06-41.09) | -3.62 (-4.13--3.11) |
| Limited Health System | 609983 (407739-931273) | 1990.05 (1326.77-3051.82) | 453079 (271333-737606) | 512.74 (306.13-838.05) | -4.4 (-4.54--4.26) |
| Middle East & North Africa - WB | 2471 (1239-4833) | 49.17 (24.64-95.92) | 2366 (1406-4138) | 16.61 (9.82-29.03) | -3.22 (-3.45--3) |
| Minimal Health System | 28701 (15598-44430) | 1229.88 (665.76-1907.84) | 24946 (12834-38813) | 556.09 (285.4-865.83) | -2.61 (-2.85--2.36) |
| North Africa and Middle East | 3408 (1713-6906) | 49.59 (24.9-100.21) | 2747 (1432-4950) | 14.18 (7.37-25.55) | -3.95 (-4.12--3.78) |
| North America | 555 (479-599) | 2.57 (2.24-2.77) | 9007 (7512-9821) | 22.63 (19.07-24.6) | 8.95 (6.75-11.2) |
| Northern Africa | 1742 (951-3331) | 68.18 (37.09-129.84) | 1211 (712-2181) | 18.98 (11.12-34.14) | -3.94 (-4.09--3.8) |
| Oceania | 674 (347-1047) | 663.78 (341.45-1033.17) | 963 (506-1540) | 364.13 (190.5-582.28) | -1.51 (-1.68--1.33) |
| Region of the Americas | 15173 (13695-16607) | 46.87 (42.3-51.34) | 22029 (18936-24265) | 29.24 (25.28-32.24) | -0.52 (-0.98--0.05) |
| South-East Asia Region | 579236 (381166-888104) | 2187.8 (1437.12-3369.72) | 418182 (244492-673471) | 497.5 (290.33-805.2) | -4.71 (-4.81--4.6) |
| South Asia | 529769 (364053-797015) | 2407.73 (1648.92-3644.65) | 394956 (235002-652492) | 576.06 (342.1-956.67) | -4.63 (-4.77--4.5) |
| South Asia - WB | 531550 (365189-799940) | 2336.24 (1599.58-3537.61) | 395638 (235362-653502) | 562.31 (333.87-933.69) | -4.61 (-4.75--4.47) |
| Southeast Asia | 88627 (37463-155515) | 866.32 (367.31-1523.67) | 44126 (19719-63451) | 154.97 (69.53-222.9) | -5.25 (-5.51--5) |
| Southern Africa | 14823 (8076-23541) | 836.19 (453.82-1331.33) | 13913 (7303-20997) | 367.56 (192.49-554.26) | -2.5 (-2.73--2.28) |
| Southern Latin America | 576 (523-615) | 23.49 (21.35-25.1) | 1028 (887-1145) | 20.13 (17.44-22.41) | 0.52 (0.07-0.98) |
| Southern Sub-Saharan Africa | 6376 (3371-11011) | 535.82 (281.51-925.08) | 7513 (3956-12125) | 303.46 (159.84-488.79) | -1.57 (-1.81--1.33) |
| Sub-Saharan Africa - WB | 101816 (54071-162116) | 1140.65 (603.86-1820.24) | 82552 (42892-126619) | 434.65 (225.64-666.22) | -3.17 (-3.4--2.93) |
| Tropical Latin America | 3960 (3409-4419) | 97.62 (84.34-108.9) | 4300 (3653-4702) | 32.26 (27.6-35.2) | -3.31 (-3.8--2.82) |
| Western Africa | 42635 (22419-67911) | 1283.49 (671.43-2052.74) | 28667 (15978-45069) | 425.49 (236.67-669.45) | -3.67 (-3.95--3.39) |
| Western Europe | 2371 (2085-2554) | 6.65 (5.86-7.16) | 12627 (10296-14057) | 20.05 (16.45-22.28) | 4.83 (3.91-5.76) |
| Western Pacific Region | 19709 (9257-31566) | 38.64 (18.14-61.99) | 13627 (8826-20380) | 8.64 (5.55-12.99) | -4.67 (-4.93--4.42) |
| Western Sub-Saharan Africa | 47517 (24870-75789) | 1292.51 (672.9-2070.46) | 32729 (18147-50992) | 435.25 (241.09-678.59) | -3.61 (-3.87--3.35) |
| World Bank High Income | 5075 (4418-5579) | 6.67 (5.82-7.33) | 29237 (24142-32277) | 19.17 (15.98-21.1) | 4.92 (3.94-5.91) |
| World Bank Low Income | 52738 (27335-86468) | 932.57 (481.04-1532.24) | 44674 (22573-67224) | 354.38 (179.15-534.44) | -3.32 (-3.51--3.12) |
| World Bank Lower Middle Income | 659812 (428219-1008694) | 1598.56 (1034.97-2458.22) | 468695 (276411-751039) | 421.52 (247.91-678.37) | -4.22 (-4.33--4.1) |
| World Bank Upper Middle Income | 34462 (22825-55389) | 52.3 (34.65-83.97) | 27004 (19027-38213) | 14.6 (10.28-20.69) | -3.94 (-4.29--3.59) |
| Country |  |  |  |  |  |
| Afghanistan | 105 (25-290) | 36.37 (8.87-99.73) | 31 (7-84) | 8.68 (1.96-23.55) | -5.32 (-5.86--4.77) |
| Albania | 4 (2-8) | 4.79 (2.47-8.18) | 8 (4-14) | 3.17 (1.62-5.53) | -1.52 (-1.9--1.14) |
| Algeria | 82 (44-159) | 14.25 (7.54-27.52) | 152 (79-278) | 9.23 (4.72-17.01) | -1.39 (-1.51--1.28) |
| American Samoa | 2 (1-3) | 216.33 (123.42-354.76) | 4 (2-7) | 188.05 (99.13-309.23) | -0.08 (-0.33-0.16) |
| Andorra | 0 (0-0) | 1.19 (0.61-2.04) | 0 (0-0) | 1.53 (0.71-2.76) | 1.42 (1.1-1.74) |
| Angola | 2293 (1330-3732) | 1849.11 (1068.12-3010.15) | 1773 (911-2856) | 460.68 (235.76-742.41) | -4.42 (-4.77--4.07) |
| Antigua and Barbuda | 2 (1-2) | 47.59 (42.57-53.06) | 1 (1-1) | 15.05 (13.07-17.31) | -4.05 (-4.8--3.3) |
| Argentina | 296 (266-320) | 17.3 (15.5-18.74) | 584 (505-655) | 17.8 (15.43-19.96) | 1.02 (0.58-1.46) |
| Armenia | 7 (6-8) | 6.64 (5.71-7.61) | 2 (1-2) | 0.8 (0.66-0.94) | -8.55 (-9.24--7.86) |
| Australia | 86 (76-97) | 7.53 (6.63-8.46) | 183 (148-207) | 6.19 (5.01-7.01) | 1.22 (0.13-2.32) |
| Austria | 15 (13-17) | 2.07 (1.81-2.33) | 131 (104-151) | 10.88 (8.69-12.58) | 7.18 (6.35-8.01) |
| Azerbaijan | 11 (5-18) | 5.52 (2.62-9.24) | 9 (4-18) | 2.58 (1.17-5.11) | -3 (-3.26--2.73) |
| Bahamas | 2 (2-2) | 26.3 (23.12-29.98) | 2 (1-2) | 9.72 (7.86-11.94) | -3.81 (-4.56--3.06) |
| Bahrain | 1 (1-2) | 19.17 (9.67-31.34) | 3 (1-5) | 11.44 (5.38-18.93) | -1.72 (-1.96--1.49) |
| Bangladesh | 27668 (15553-39220) | 1325.71 (744.06-1898.72) | 22251 (10225-50483) | 321.75 (145.76-737) | -5 (-5.25--4.75) |
| Barbados | 5 (5-6) | 28.13 (25.44-31.04) | 3 (2-4) | 10.17 (8.28-12.33) | -3.66 (-4.24--3.08) |
| Belarus | 7 (6-9) | 1.24 (1.05-1.43) | 6 (5-8) | 0.76 (0.59-0.95) | -3.12 (-4.1--2.13) |
| Belgium | 105 (90-116) | 11.49 (9.89-12.67) | 927 (728-1064) | 59.85 (47.21-68.71) | 6.84 (6.04-7.65) |
| Belize | 3 (3-4) | 69.95 (62.31-77.54) | 3 (3-4) | 24.72 (20.89-28.51) | -3.94 (-4.45--3.42) |
| Benin | 1548 (842-2333) | 1685.28 (912.04-2562.68) | 1099 (518-1853) | 533.79 (250.96-905.75) | -3.71 (-3.79--3.62) |
| Bermuda | 1 (1-1) | 19.56 (17.54-21.58) | 0 (0-0) | 4.59 (3.75-5.8) | -4.86 (-5.64--4.07) |
| Bhutan | 191 (86-324) | 2215.37 (997.35-3794.34) | 126 (45-288) | 417.46 (148.41-963.22) | -5.56 (-5.96--5.17) |
| Bolivia (Plurinational State of) | 198 (89-345) | 144.18 (64.7-252.88) | 196 (103-373) | 46.14 (23.95-87.96) | -3.7 (-3.82--3.58) |
| Bosnia and Herzegovina | 9 (5-17) | 5.62 (2.91-10.74) | 22 (6-52) | 6.22 (1.75-14.49) | 0.85 (0.43-1.26) |
| Botswana | 166 (78-282) | 757.17 (354.37-1290.38) | 166 (75-267) | 297.55 (135.52-483.85) | -2.69 (-2.83--2.55) |
| Brazil | 3840 (3323-4315) | 97.25 (84.47-109.22) | 4244 (3606-4634) | 32.53 (27.84-35.45) | -3.27 (-3.76--2.78) |
| Brunei Darussalam | 1 (0-1) | 12.48 (6.86-21.3) | 1 (1-2) | 8.95 (4.22-14.4) | -0.49 (-0.67--0.3) |
| Bulgaria | 10 (8-11) | 1.56 (1.33-1.82) | 77 (64-91) | 8.25 (6.83-9.84) | 6.94 (5.07-8.83) |
| Burkina Faso | 2703 (1439-4371) | 1491.69 (788.87-2424.13) | 2141 (1093-3351) | 555.93 (282.94-876) | -3.31 (-3.41--3.2) |
| Burundi | 1132 (527-1785) | 1137.74 (527.17-1810.62) | 1193 (533-1991) | 698.14 (311.39-1167.16) | -1.72 (-1.83--1.62) |
| Cabo Verde | 58 (30-92) | 441.78 (228.63-709.64) | 24 (12-47) | 124.37 (60.52-241.59) | -4.43 (-4.8--4.06) |
| Cambodia | 1334 (629-2338) | 759.5 (358.04-1335.13) | 601 (283-953) | 116.23 (54.73-185.55) | -6.31 (-6.51--6.12) |
| Cameroon | 1493 (748-2542) | 878.91 (437.23-1505.56) | 1364 (619-2325) | 292.33 (132.75-499.09) | -3.49 (-3.7--3.28) |
| Canada | 71 (61-79) | 3.77 (3.25-4.19) | 1249 (1038-1410) | 27.37 (22.74-30.97) | 9.09 (6.69-11.55) |
| Central African Republic | 431 (242-724) | 1176.06 (659.15-1975.32) | 569 (281-903) | 901.96 (444.97-1431.9) | -0.6 (-0.72--0.47) |
| Chad | 2966 (1455-4876) | 2210.84 (1079.01-3661.38) | 2462 (1355-3941) | 1115.4 (611.75-1792.32) | -2.3 (-2.46--2.14) |
| Chile | 203 (181-220) | 40.09 (35.77-43.59) | 315 (267-351) | 21.46 (18.25-23.94) | -0.93 (-1.47--0.38) |
| China | 8958 (3811-13666) | 25.28 (10.77-38.55) | 2553 (1392-4883) | 2.27 (1.24-4.35) | -8.04 (-8.4--7.68) |
| Colombia | 523 (471-567) | 68.78 (61.86-74.78) | 691 (572-811) | 23.66 (19.52-27.85) | -4.29 (-4.9--3.68) |
| Comoros | 40 (19-66) | 535.3 (257.55-873.68) | 56 (21-101) | 274.02 (104.48-497.79) | -1.94 (-2.07--1.8) |
| Congo | 291 (140-506) | 712.16 (340.67-1240.06) | 296 (113-574) | 324.81 (123.85-632.57) | -2.36 (-2.66--2.06) |
| Cook Islands | 0 (0-0) | 49.52 (25.74-83.27) | 0 (0-1) | 33.82 (15.79-54.19) | -1.08 (-1.39--0.76) |
| Costa Rica | 63 (55-69) | 72.67 (64.34-80.64) | 64 (53-74) | 22.1 (18.42-25.57) | -3.67 (-4.2--3.14) |
| Croatia | 8 (7-9) | 2.99 (2.63-3.43) | 65 (54-75) | 11.22 (9.42-13.09) | 6.01 (4.01-8.05) |
| Cuba | 272 (248-293) | 48.38 (44-52.46) | 187 (160-213) | 17.21 (14.69-19.78) | -4.79 (-5.65--3.91) |
| Cyprus | 16 (8-32) | 32.64 (15.9-66.48) | 38 (20-59) | 29.62 (15.44-45.88) | -0.14 (-0.26--0.02) |
| Czechia | 33 (29-38) | 4.52 (3.92-5.24) | 414 (335-492) | 29.25 (23.71-34.79) | 9.22 (7.18-11.3) |
| C?te d'Ivoire | 913 (485-1390) | 712.14 (377.87-1085.63) | 1083 (508-1829) | 276.67 (130.11-467.27) | -2.58 (-2.8--2.35) |
| Democratic People's Republic of Korea | 26 (12-47) | 4.2 (1.94-7.56) | 47 (25-83) | 2.95 (1.54-5.23) | -1.17 (-1.44--0.91) |
| Democratic Republic of the Congo | 4074 (2427-6687) | 743.9 (442.15-1221.6) | 3740 (1503-6704) | 308.14 (123.53-553.4) | -2.88 (-3.69--2.07) |
| Denmark | 72 (63-79) | 13.72 (12.05-15.17) | 386 (319-437) | 47.12 (39.03-53.46) | 5.16 (4.15-6.18) |
| Djibouti | 33 (16-53) | 805.92 (398.76-1293.32) | 60 (24-105) | 289.29 (115.22-507.33) | -3.25 (-3.37--3.13) |
| Dominica | 2 (1-2) | 45.25 (21.67-66.5) | 1 (0-1) | 20.66 (10.3-35.5) | -2.59 (-2.86--2.31) |
| Dominican Republic | 161 (81-266) | 95.22 (47.85-158.82) | 247 (118-442) | 48.87 (23.41-87.66) | -2.42 (-2.53--2.31) |
| Ecuador | 371 (335-401) | 152.77 (137.63-165.76) | 116 (95-141) | 13.54 (11.02-16.55) | -7.72 (-8.25--7.18) |
| Egypt | 785 (382-1657) | 81.59 (39.58-171.8) | 501 (280-1009) | 22.33 (12.46-44.9) | -3.93 (-4.13--3.74) |
| El Salvador | 310 (156-532) | 216.88 (108.52-369.58) | 158 (86-288) | 45.77 (24.73-83.38) | -5.09 (-5.58--4.6) |
| Equatorial Guinea | 107 (54-200) | 1526.38 (764.74-2849.35) | 30 (13-52) | 164.46 (69.33-289.12) | -8.07 (-8.48--7.65) |
| Eritrea | 517 (210-897) | 1800.86 (732.32-3121.58) | 587 (184-1132) | 669.3 (210.1-1296.24) | -3.18 (-3.28--3.08) |
| Estonia | 1 (1-2) | 1.34 (1.14-1.54) | 1 (1-1) | 0.74 (0.6-0.9) | -3.69 (-4.89--2.47) |
| Eswatini | 114 (56-179) | 1038.7 (509.76-1638.01) | 66 (30-112) | 312.32 (141.2-532.42) | -3.81 (-4.03--3.6) |
| Ethiopia | 14012 (5028-26127) | 1945.95 (697.39-3627.37) | 11810 (5746-17681) | 658.57 (321.46-991.38) | -3.85 (-4.05--3.65) |
| Fiji | 59 (34-102) | 461.77 (260.77-790.19) | 81 (41-148) | 261.25 (132.32-476.95) | -1.72 (-1.95--1.5) |
| Finland | 31 (27-35) | 7.16 (6.17-7.93) | 97 (78-112) | 11.02 (8.91-12.63) | 2.33 (1.56-3.11) |
| France | 978 (847-1089) | 19.36 (16.78-21.57) | 1180 (951-1364) | 12.48 (10.09-14.43) | -1.84 (-2.7--0.98) |
| Gabon | 171 (79-314) | 664.77 (303.21-1229.64) | 70 (26-146) | 179.47 (65.74-376.55) | -4.26 (-4.37--4.16) |
| Gambia | 107 (59-174) | 766.78 (417.51-1240.27) | 124 (57-222) | 305.72 (141.25-550.96) | -2.96 (-3.08--2.84) |
| Georgia | 12 (11-15) | 4.39 (3.74-5.17) | 3 (2-3) | 0.77 (0.63-0.92) | -6.9 (-7.77--6.02) |
| Germany | 542 (466-602) | 7.14 (6.15-7.92) | 3096 (2477-3518) | 24.05 (19.33-27.32) | 7.02 (5.6-8.45) |
| Ghana | 2076 (1131-3654) | 881.32 (478.35-1551.61) | 1977 (1030-3306) | 306.28 (159.08-512.85) | -3.33 (-3.4--3.27) |
| Greece | 7 (6-8) | 0.8 (0.71-0.89) | 80 (65-89) | 4.78 (3.9-5.37) | 6.6 (6.12-7.09) |
| Greenland | 0 (0-1) | 23.36 (13-43.39) | 1 (0-1) | 22.7 (7.82-43.52) | 0.27 (0.08-0.45) |
| Grenada | 2 (1-2) | 38.15 (33.18-42.94) | 1 (1-1) | 11.45 (9.78-13.18) | -4.54 (-5.06--4.01) |
| Guam | 2 (1-3) | 73.45 (39.33-123.11) | 5 (3-9) | 43.41 (22.17-78.03) | -0.92 (-1.34--0.5) |
| Guatemala | 1275 (1174-1360) | 950.56 (873.78-1015.92) | 938 (812-1086) | 176.75 (152.32-205.51) | -5.09 (-5.7--4.48) |
| Guinea | 3362 (1442-6412) | 2210.24 (945.24-4216.47) | 1448 (685-2311) | 610.93 (287.9-983.23) | -3.95 (-4.11--3.78) |
| Guinea-Bissau | 235 (131-359) | 1542.89 (854.43-2374.46) | 128 (63-209) | 527.34 (261.56-866.31) | -3.43 (-3.7--3.15) |
| Guyana | 33 (29-37) | 205.69 (179.59-232.37) | 20 (15-25) | 72.86 (55.74-93.27) | -3.6 (-4.23--2.96) |
| Haiti | 473 (204-839) | 378.31 (162.81-673.19) | 428 (168-855) | 159.07 (62.04-317.73) | -2.56 (-2.85--2.27) |
| Honduras | 397 (220-697) | 450.81 (249.36-788.71) | 498 (258-828) | 174.71 (90.04-291.61) | -3.06 (-3.25--2.88) |
| Hungary | 21 (18-25) | 2.7 (2.29-3.16) | 270 (224-322) | 22.03 (18.37-26.21) | 9.54 (7.55-11.57) |
| Iceland | 1 (1-1) | 4.59 (3.94-5.15) | 4 (3-5) | 11.93 (9.19-13.81) | 5.03 (3.99-6.09) |
| India | 467224 (319913-706975) | 2742.49 (1871.19-4172.5) | 354493 (206953-586779) | 635.42 (370.53-1055.49) | -4.7 (-4.83--4.56) |
| Indonesia | 65199 (26842-112002) | 1792.59 (739.69-3082.48) | 30275 (12452-47681) | 324.16 (133.65-509.94) | -5.05 (-5.34--4.77) |
| Iran (Islamic Republic of) | 257 (121-552) | 26.78 (12.6-57.5) | 366 (183-714) | 10.48 (5.21-20.43) | -2.53 (-2.87--2.19) |
| Iraq | 147 (67-309) | 39.43 (18.19-82.78) | 128 (68-252) | 13.54 (7.15-26.66) | -3.72 (-3.89--3.55) |
| Ireland | 8 (7-9) | 2.98 (2.6-3.36) | 43 (34-50) | 8.48 (6.79-10.03) | 4.67 (3.78-5.57) |
| Israel | 27 (24-31) | 9.41 (8.2-10.62) | 303 (242-352) | 38.29 (30.68-44.37) | 5.78 (4.69-6.89) |
| Italy | 87 (75-94) | 1.65 (1.43-1.79) | 1951 (1533-2224) | 19.36 (15.32-22.04) | 12.35 (10.36-14.36) |
| Jamaica | 85 (76-92) | 81.87 (73.23-89.53) | 60 (47-74) | 36.55 (28.38-45.66) | -2.1 (-2.89--1.3) |
| Japan | 799 (683-860) | 8.46 (7.28-9.11) | 3424 (2627-3890) | 12.68 (9.87-14.34) | 1.85 (1.52-2.19) |
| Jordan | 6 (3-10) | 13.43 (7.19-21.77) | 17 (8-31) | 5.88 (2.74-10.64) | -3.01 (-3.23--2.79) |
| Kazakhstan | 33 (29-38) | 6.49 (5.64-7.36) | 6 (5-8) | 0.84 (0.69-1.02) | -8.93 (-9.77--8.07) |
| Kenya | 3872 (1945-6192) | 1150.22 (576.09-1845.11) | 3636 (1590-5915) | 414.81 (181.29-675.42) | -2.87 (-3.17--2.56) |
| Kiribati | 26 (13-39) | 1842.15 (916.76-2812.97) | 22 (10-33) | 886.15 (415.41-1369.32) | -2.08 (-2.29--1.87) |
| Kuwait | 1 (1-1) | 5.74 (4.79-6.52) | 3 (2-3) | 2.98 (2.37-3.62) | -0.1 (-1.09-0.9) |
| Kyrgyzstan | 12 (10-13) | 9.21 (7.96-10.69) | 2 (1-2) | 0.97 (0.78-1.21) | -8.59 (-9.21--7.95) |
| Lao People's Democratic Republic | 773 (370-1631) | 923.85 (442.53-1951.2) | 252 (137-469) | 138.22 (74.93-257.19) | -6.3 (-6.46--6.14) |
| Latvia | 2 (2-2) | 1.09 (0.94-1.25) | 2 (1-2) | 0.64 (0.52-0.77) | -3.11 (-4.2--2.01) |
| Lebanon | 32 (18-52) | 34.62 (19.41-56.73) | 86 (46-140) | 23.54 (12.66-38.48) | -0.78 (-1.14--0.42) |
| Lesotho | 655 (322-1069) | 1689.27 (831.28-2770.2) | 298 (116-565) | 666.86 (259.63-1263.56) | -2.8 (-2.99--2.6) |
| Liberia | 488 (275-827) | 906.53 (510.19-1543.25) | 306 (150-525) | 408.84 (198.94-703.76) | -3.12 (-3.49--2.75) |
| Libya | 46 (16-119) | 56.02 (19.86-143.79) | 31 (17-61) | 15.16 (8.06-29.39) | -3.71 (-4.26--3.16) |
| Lithuania | 4 (3-4) | 1.76 (1.52-2.03) | 8 (7-10) | 2.34 (1.91-2.78) | -0.63 (-2.29-1.06) |
| Luxembourg | 3 (3-3) | 9.31 (8.37-10.38) | 21 (17-24) | 31.93 (25.93-37.11) | 4.92 (4.18-5.67) |
| Madagascar | 1778 (1072-3222) | 897.21 (539.99-1628.38) | 1688 (698-3444) | 479.71 (198.27-978.55) | -1.64 (-1.8--1.47) |
| Malawi | 2369 (1176-4084) | 1605.5 (795.67-2775.11) | 1968 (957-3072) | 684.66 (332.88-1071.24) | -2.85 (-3--2.69) |
| Malaysia | 439 (256-840) | 110.13 (63.99-210.62) | 771 (349-1185) | 57.87 (26.16-89.39) | -2.37 (-2.53--2.21) |
| Maldives | 14 (7-24) | 469.88 (223.57-828.01) | 6 (3-10) | 49.45 (25.02-77.78) | -7.14 (-8.12--6.14) |
| Mali | 2658 (1497-4014) | 1737.72 (979.34-2628) | 2072 (839-4093) | 604.74 (244.4-1199.87) | -3.38 (-3.52--3.25) |
| Malta | 0 (0-0) | 1.79 (1.58-1.99) | 4 (3-4) | 5.18 (4.1-6.01) | 4.4 (3.47-5.34) |
| Marshall Islands | 3 (2-5) | 441.63 (224.06-789.65) | 2 (1-4) | 189.29 (112.28-329.41) | -2.44 (-2.61--2.27) |
| Mauritania | 405 (214-629) | 866.43 (458.26-1356.03) | 230 (112-395) | 244.75 (118.9-422.2) | -4 (-4.16--3.84) |
| Mauritius | 25 (23-28) | 81.51 (73.29-89.57) | 40 (36-44) | 42.84 (38.08-47.27) | -0.25 (-0.76-0.26) |
| Mexico | 4620 (4374-4811) | 241.14 (228.59-251.02) | 2722 (2416-3019) | 44.28 (39.24-49.21) | -4.82 (-5.74--3.89) |
| Micronesia (Federated States of) | 9 (5-16) | 398.78 (205.2-702.68) | 5 (3-9) | 209 (118.89-378.51) | -1.61 (-2.04--1.17) |
| Monaco | 0 (0-0) | 1.99 (0.88-4.34) | 0 (0-0) | 3.52 (1.4-5.79) | 2.25 (1.8-2.71) |
| Mongolia | 1 (0-1) | 1.57 (0.43-3.11) | 0 (0-0) | 0.26 (0.13-0.49) | -6.69 (-7.23--6.15) |
| Montenegro | 0 (0-0) | 0.59 (0.28-1.03) | 0 (0-0) | 0.54 (0.27-0.9) | -0.28 (-0.49--0.06) |
| Morocco | 380 (108-808) | 56.99 (16.35-121.2) | 228 (118-411) | 14.58 (7.55-26.15) | -4.27 (-4.45--4.1) |
| Mozambique | 2337 (1138-3842) | 1026.98 (498.01-1696.03) | 1668 (712-2986) | 412.11 (175.59-738.25) | -2.75 (-2.93--2.56) |
| Myanmar | 6390 (2328-13997) | 691.95 (251.56-1521.67) | 2447 (1198-3836) | 115.55 (56.45-181.77) | -5.79 (-6.07--5.51) |
| Namibia | 257 (130-418) | 1000.76 (503.94-1626.91) | 196 (90-330) | 353.71 (162.85-599.78) | -3.22 (-3.33--3.11) |
| Nauru | 0 (0-1) | 226.26 (121.59-396.87) | 0 (0-1) | 145.34 (73.25-282.39) | -1.18 (-1.46--0.9) |
| Nepal | 4464 (2640-7332) | 1273.96 (750.38-2093.76) | 2594 (1289-5241) | 237.51 (117.59-480.23) | -5.54 (-5.64--5.44) |
| Netherlands | 48 (42-53) | 3.98 (3.44-4.42) | 482 (394-541) | 20.25 (16.62-22.74) | 6.07 (5.09-7.05) |
| New Zealand | 26 (23-30) | 11.26 (9.65-12.85) | 89 (71-102) | 16.58 (13.27-18.89) | 4.44 (2.76-6.16) |
| Nicaragua | 105 (51-172) | 156.15 (76.04-255.96) | 76 (44-130) | 34.22 (19.78-58.53) | -4.71 (-4.87--4.55) |
| Niger | 2199 (1064-3692) | 2236.29 (1080.17-3766.48) | 2757 (1454-4511) | 879.84 (462.43-1450.82) | -3.19 (-3.56--2.82) |
| Nigeria | 22457 (11222-37736) | 1177.26 (586.54-1984.43) | 12935 (7460-21692) | 375.19 (216.15-630.63) | -3.9 (-4.3--3.49) |
| Niue | 0 (0-1) | 262.39 (143.96-484.92) | 0 (0-0) | 133.78 (72.66-251.66) | -2.24 (-2.71--1.78) |
| North Macedonia | 12 (6-20) | 14.67 (7.66-24.64) | 20 (9-38) | 11.56 (4.82-21.64) | -0.23 (-0.57-0.11) |
| Northern Mariana Islands | 1 (0-1) | 147.01 (83.58-240.99) | 3 (2-4) | 140.33 (75.94-219.82) | 0.36 (0.08-0.64) |
| Norway | 43 (36-47) | 9.14 (7.74-10.05) | 386 (317-431) | 57.29 (47.31-63.91) | 7.6 (6.15-9.07) |
| Oman | 9 (4-16) | 34.2 (15.44-61.34) | 8 (4-13) | 12.19 (5.75-20.5) | -3.09 (-3.48--2.7) |
| Pakistan | 30222 (13010-57217) | 1154.96 (494.29-2208.8) | 15492 (7540-28738) | 322.83 (156.54-600.33) | -4.29 (-4.52--4.07) |
| Palau | 2 (1-3) | 389.41 (202.58-672.52) | 2 (1-4) | 265.12 (135.88-459.88) | -0.61 (-0.86--0.37) |
| Palestine | 11 (5-18) | 27.35 (12.95-44.09) | 5 (2-8) | 5.08 (2.24-7.89) | -5.32 (-5.68--4.97) |
| Panama | 74 (65-84) | 102.51 (89.81-115.64) | 63 (49-78) | 26.71 (20.44-32.78) | -3.69 (-4.11--3.27) |
| Papua New Guinea | 476 (239-740) | 799.37 (400.43-1242.82) | 736 (371-1187) | 438.2 (220.09-707.84) | -1.42 (-1.6--1.23) |
| Paraguay | 120 (61-286) | 111.04 (56-264.55) | 55 (23-97) | 19.51 (8.25-34.22) | -5.28 (-5.85--4.71) |
| Peru | 551 (267-831) | 100.57 (48.69-152.8) | 434 (235-782) | 25.19 (13.56-45.75) | -4.93 (-5.35--4.5) |
| Philippines | 2832 (1341-6482) | 234.83 (110.64-537.28) | 2690 (1573-5049) | 78.5 (45.88-147.12) | -3.39 (-3.45--3.33) |
| Poland | 34 (31-37) | 1.52 (1.4-1.63) | 857 (740-952) | 19.47 (16.86-21.61) | 10.8 (8.49-13.16) |
| Portugal | 27 (24-30) | 3.38 (3.01-3.72) | 308 (250-348) | 18.14 (14.81-20.57) | 7.01 (6.35-7.67) |
| Puerto Rico | 34 (31-37) | 16.82 (15.21-18.38) | 71 (57-84) | 14.88 (11.98-17.65) | -1.24 (-2.16--0.3) |
| Qatar | 0 (0-0) | 12.63 (7.12-20.1) | 1 (1-2) | 7.6 (3.53-12.66) | -1.74 (-1.97--1.51) |
| Republic of Korea | 387 (159-626) | 31.68 (13.13-51.23) | 1069 (494-2026) | 20.1 (9.22-38.32) | -0.87 (-1.52--0.21) |
| Republic of Moldova | 7 (6-8) | 3.48 (3-3.96) | 5 (4-6) | 1.59 (1.29-1.95) | -3.97 (-4.87--3.07) |
| Romania | 32 (28-36) | 2.36 (2.07-2.67) | 307 (256-358) | 13.26 (11.03-15.52) | 6.88 (4.69-9.12) |
| Russian Federation | 214 (203-222) | 2.64 (2.51-2.73) | 244 (222-265) | 1.94 (1.76-2.1) | -2.87 (-3.83--1.91) |
| Rwanda | 703 (228-1361) | 665.48 (215.46-1291.09) | 614 (220-1226) | 260.22 (93.35-521.08) | -3.54 (-3.85--3.24) |
| Saint Kitts and Nevis | 4 (3-4) | 148.9 (134.59-165.48) | 1 (1-1) | 46.3 (39.1-53.92) | -3.62 (-4.22--3.02) |
| Saint Lucia | 3 (3-4) | 71.82 (65.42-78.91) | 3 (2-3) | 21.24 (17.3-25.37) | -4.56 (-5.49--3.62) |
| Saint Vincent and the Grenadines | 4 (4-5) | 102.39 (91.54-114.08) | 2 (2-2) | 28.37 (24.31-32.75) | -4.22 (-4.97--3.46) |
| Samoa | 5 (3-10) | 146.53 (78.36-263.84) | 7 (4-12) | 103.75 (57.22-188.83) | -0.84 (-1.16--0.52) |
| San Marino | 0 (0-0) | 2.66 (1.42-5.41) | 0 (0-0) | 2.13 (0.93-3.71) | 0.35 (-0.11-0.8) |
| Sao Tome and Principe | 16 (9-25) | 539.45 (314.54-849.19) | 5 (3-9) | 124.64 (62.84-211.1) | -5.14 (-5.5--4.78) |
| Saudi Arabia | 165 (74-306) | 70.57 (31.6-130.72) | 133 (63-238) | 25.77 (12.18-46.11) | -2.75 (-3.03--2.46) |
| Senegal | 1964 (1089-3173) | 1423.2 (786.23-2307.69) | 1023 (518-1665) | 315.26 (159.21-515.26) | -4.76 (-4.93--4.59) |
| Serbia | 14 (7-27) | 2.87 (1.49-5.62) | 45 (15-90) | 4.43 (1.45-8.97) | 2.68 (2.09-3.26) |
| Seychelles | 3 (2-6) | 105.53 (60.97-200.36) | 3 (1-4) | 51.48 (27-79.94) | -1.87 (-2.09--1.66) |
| Sierra Leone | 1282 (633-2095) | 1326.05 (649.07-2181.48) | 889 (409-1481) | 573.44 (262.04-966.84) | -2.82 (-3.27--2.37) |
| Singapore | 18 (16-20) | 18.52 (16.68-20.41) | 49 (40-55) | 10.54 (8.67-11.95) | -0.75 (-1.19--0.32) |
| Slovakia | 6 (3-11) | 2.02 (1.07-3.44) | 11 (4-20) | 2.02 (0.78-3.66) | 0.78 (0.45-1.11) |
| Slovenia | 2 (1-2) | 1.21 (1.01-1.41) | 16 (13-20) | 5.75 (4.59-7.16) | 6.18 (4.08-8.32) |
| Solomon Islands | 27 (15-49) | 588.65 (324.89-1055.18) | 31 (17-55) | 244.53 (137.63-436.05) | -2.51 (-2.77--2.25) |
| Somalia | 1362 (595-2506) | 2034.99 (884.57-3749.88) | 2295 (997-4051) | 1194.05 (519.1-2106.55) | -2.03 (-2.23--1.82) |
| South Africa | 4325 (2247-8235) | 464.57 (239.39-886.9) | 5651 (3021-10006) | 275.13 (147.14-487.56) | -1.53 (-1.76--1.3) |
| South Sudan | 1791 (841-3220) | 1566.42 (731.82-2821.15) | 1867 (939-3407) | 1441.68 (722.12-2645.42) | -0.23 (-0.42--0.05) |
| Spain | 192 (166-211) | 5.79 (5.03-6.38) | 1194 (947-1388) | 18.32 (14.58-21.29) | 4.95 (4.58-5.33) |
| Sri Lanka | 1662 (822-2721) | 358.8 (177.16-592.08) | 645 (295-1121) | 45.18 (20.55-79.3) | -6.23 (-6.7--5.77) |
| Sudan | 570 (142-1369) | 131.51 (32.83-315.35) | 291 (56-769) | 36.12 (6.92-95.63) | -4.21 (-4.63--3.79) |
| Suriname | 16 (9-27) | 145.31 (75.9-241.13) | 16 (9-31) | 53.83 (29.27-102.04) | -3.08 (-3.3--2.86) |
| Sweden | 27 (23-30) | 2.57 (2.16-2.87) | 838 (681-948) | 54.12 (44.02-61.31) | 11.63 (10.03-13.24) |
| Switzerland | 54 (46-61) | 8.14 (6.96-9.2) | 273 (210-319) | 22.69 (17.48-26.46) | 5.12 (4.33-5.92) |
| Syrian Arab Republic | 49 (24-92) | 23.02 (11.07-43.03) | 45 (24-80) | 7.93 (4.19-14.16) | -3.03 (-3.59--2.47) |
| Taiwan (Province of China) | 78 (70-85) | 11.19 (10.05-12.27) | 80 (67-93) | 3.37 (2.83-3.89) | -1.97 (-3.04--0.89) |
| Tajikistan | 19 (9-31) | 16.52 (7.57-27.17) | 11 (5-20) | 5.9 (2.79-10.7) | -4.22 (-4.85--3.59) |
| Thailand | 6318 (1506-19846) | 439.91 (104.54-1383.33) | 5241 (2520-9048) | 90.15 (43.2-155.35) | -4.98 (-5.54--4.42) |
| Timor-Leste | 80 (28-189) | 966.96 (337.49-2270.4) | 57 (29-110) | 134.53 (69.23-262.51) | -6.41 (-6.83--6) |
| Togo | 585 (312-929) | 1248.26 (663.63-1984.8) | 663 (325-1085) | 490.01 (239.37-803.47) | -2.86 (-3.08--2.64) |
| Tokelau | 0 (0-1) | 448.34 (241.79-802.63) | 0 (0-0) | 169.5 (95.48-304.06) | -2.9 (-3.38--2.42) |
| Tonga | 3 (1-5) | 104.78 (51.85-185.67) | 3 (1-6) | 73.11 (35.22-138.14) | -0.86 (-1.17--0.55) |
| Trinidad and Tobago | 26 (24-29) | 62.06 (56.31-68.38) | 16 (13-20) | 15.73 (12.21-19.29) | -4.99 (-5.57--4.4) |
| Tunisia | 44 (22-88) | 19.23 (9.62-38.6) | 69 (32-136) | 10.37 (4.74-20.59) | -1.66 (-1.89--1.43) |
| Turkmenistan | 8 (7-9) | 10.5 (9.28-11.95) | 2 (2-2) | 1.33 (1.05-1.67) | -9.08 (-9.99--8.17) |
| Tuvalu | 1 (1-2) | 405.33 (209.34-748.86) | 1 (1-2) | 186.55 (107.98-336.99) | -1.8 (-2.19--1.41) |
| Türkiye | 321 (171-643) | 22.44 (11.97-44.75) | 424 (202-633) | 8.73 (4.14-13.07) | -2.45 (-2.95--1.94) |
| Uganda | 3059 (1041-6870) | 1151.6 (389.68-2599.06) | 2066 (862-3492) | 372.78 (155.71-632.69) | -4.06 (-4.28--3.84) |
| Ukraine | 23 (21-25) | 0.68 (0.61-0.75) | 13 (10-17) | 0.33 (0.24-0.42) | -4.64 (-6.09--3.16) |
| United Arab Emirates | 3 (1-5) | 25.33 (11.26-43.21) | 6 (3-11) | 11.07 (4.69-18.41) | -0.91 (-1.48--0.33) |
| United Kingdom | 86 (78-92) | 1.5 (1.36-1.59) | 877 (739-947) | 10.07 (8.55-10.86) | 5.91 (2.81-9.11) |
| United Republic of Tanzania | 5539 (2896-8392) | 1245.17 (649.1-1892.53) | 5059 (2695-8808) | 499.76 (264.42-873.12) | -3.09 (-3.27--2.9) |
| United States of America | 483 (415-523) | 2.46 (2.13-2.65) | 7757 (6465-8454) | 22.04 (18.56-23.95) | 8.91 (6.74-11.13) |
| United States Virgin Islands | 1 (0-1) | 19.58 (9.24-29.75) | 1 (0-2) | 8.57 (4-16.26) | -2.7 (-2.83--2.56) |
| Uruguay | 77 (69-84) | 33.78 (30.25-37.1) | 129 (110-145) | 36.96 (31.65-41.75) | 1.35 (0.87-1.83) |
| Uzbekistan | 32 (26-37) | 6.57 (5.5-7.78) | 7 (6-9) | 0.79 (0.62-0.99) | -8.81 (-9.64--7.97) |
| Vanuatu | 14 (7-24) | 608.14 (314.46-1044.9) | 18 (9-31) | 271.65 (135.19-479.62) | -2.59 (-2.72--2.45) |
| Venezuela (Bolivarian Republic of) | 504 (454-549) | 118.11 (106.22-128.84) | 748 (571-943) | 50.11 (38.09-63.34) | -3.07 (-3.59--2.55) |
| Viet Nam | 3431 (1119-7872) | 178.51 (58.66-408.65) | 1038 (369-1964) | 24.18 (8.66-45.66) | -6.15 (-6.48--5.83) |
| Yemen | 391 (149-815) | 209.33 (79.9-435.1) | 216 (79-466) | 38.13 (13.97-82.29) | -5.73 (-5.97--5.48) |
| Zambia | 1448 (769-2391) | 1367.1 (721.27-2273.7) | 990 (510-1633) | 402.56 (207.4-666.28) | -4.19 (-4.67--3.7) |
| Zimbabwe | 860 (359-1486) | 512.55 (212.62-892.49) | 1137 (410-2174) | 444.5 (160.11-851.97) | 0.35 (-0.15-0.85) |

eTable 3. DALYs of diarrhea disease in the elderly at the global, sex, age-group levels, regional, ,and national levels from 1990 to 2021.

| **Location** | **Rate per 100 000(95% UI)** | | | | |
| --- | --- | --- | --- | --- | --- |
|  | **1990** | | **2021** | | **1990-2021** |
|  | **DALYs cases** | **DALYs rate** | **DALYs cases** | **DALYs rate** | **EAPC** |
| Global | 12530559 (8017128-19219924) | 7224.87 (4598.74-11173.96) | 9259412 (5757427-14175665) | 2182.72 (1354.8-3357.08) | -3.76 (-3.9--3.63) |
| Sex |  |  |  |  |  |
| Female | 6735328 (3579522-11551224) | 6745.89 (3519.24-11717.16) | 5253127 (2531251-9382593) | 2238.01 (1069.67-4012.57) | -3.45 (-3.55--3.35) |
| Male | 5795231 (3143837-9816752) | 7870.29 (4254.13-13408.52) | 4006286 (2151341-6963458) | 2114.12 (1124.1-3683.46) | -4.15 (-4.33--3.97) |
| Age |  |  |  |  |  |
| <65 years | 0 (0-0) | 0 (0-0) | 0 (0-0) | 0 (0-0) | 0 (0-0) |
| 65-69 years | 3254590 (2006588-5285508) | 2632.96 (1623.33-4275.97) | 1932869 (1186918-3055566) | 700.72 (430.29-1107.72) | -4.23 (-4.45--4.01) |
| 70-74 years | 3322976 (2081830-5194499) | 3925.03 (2459.01-6135.63) | 2285284 (1353098-3640323) | 1110.23 (657.36-1768.53) | -3.84 (-3.98--3.69) |
| 75-79 years | 2618161 (1678128-4024570) | 4253.33 (2726.2-6538.12) | 1873349 (1160007-2919953) | 1420.45 (879.56-2214.02) | -3.63 (-3.77--3.5) |
| 80-84 years | 1964356 (1307884-2859540) | 5552.8 (3697.1-8083.29) | 1611719 (996308-2466676) | 1840.22 (1137.56-2816.39) | -3.42 (-3.49--3.35) |
| 85-89 years | 946479 (640755-1337198) | 6263.48 (4240.3-8849.12) | 964032 (642733-1504478) | 2108.47 (1405.75-3290.5) | -3.54 (-3.74--3.34) |
| 90-94 years | 325215 (215558-455056) | 7589.27 (5030.3-10619.28) | 438305 (287185-660492) | 2450.09 (1605.34-3692.09) | -3.56 (-3.88--3.25) |
| 95+ years | 98782 (64369-137241) | 9702.71 (6322.57-13480.32) | 153856 (99664-225686) | 2822.88 (1828.6-4140.78) | -3.56 (-3.98--3.14) |
| SDI region |  |  |  |  |  |
| High-middle SDI | 131592 (79185-178830) | 293.9 (177.13-399.79) | 199178 (144673-245982) | 192.97 (139.24-239.74) | -1.02 (-1.36--0.69) |
| High SDI | 83706 (72223-96307) | 140.83 (120.74-163.29) | 374783 (323035-415840) | 303.28 (262.96-336.42) | 3.68 (2.69-4.67) |
| Low-middle SDI | 7315097 (4858697-11351881) | 31078.05 (20581.94-48486.94) | 4684576 (2858834-7577174) | 7446.61 (4543.09-12077.93) | -4.63 (-4.75--4.52) |
| Low SDI | 2893431 (1859173-4362181) | 34052.68 (21811.67-51653.67) | 2313681 (1378690-3775225) | 11731.15 (6990.66-19155.42) | -3.42 (-3.62--3.23) |
| Middle SDI | 2102628 (1137254-3309791) | 5070.12 (2743.1-7995.75) | 1683657 (969486-2442434) | 1342.47 (774.22-1950.82) | -4.27 (-4.37--4.16) |
| GBD region |  |  |  |  |  |
| Advanced Health System | 104845 (91269-120691) | 122.83 (106.28-142.3) | 459318 (396806-508571) | 274.25 (238.14-303.73) | 3.72 (2.93-4.53) |
| Africa | 1788196 (963130-2845624) | 16302 (8747.62-25995.94) | 1425289 (761519-2172815) | 5869.09 (3142.51-8927.93) | -3.38 (-3.59--3.16) |
| African Region | 1735185 (928353-2755060) | 19987.48 (10661.5-31813.19) | 1360931 (727040-2076823) | 7206.91 (3862.38-10980.79) | -3.38 (-3.61--3.16) |
| America | 250046 (228173-272798) | 829.77 (756.78-906.07) | 334031 (295785-366595) | 475.49 (423.35-522.45) | -1.06 (-1.42--0.7) |
| Andean Latin America | 18934 (13144-24328) | 2176.21 (1513.25-2808.97) | 12978 (9005-20458) | 466.49 (322.72-738.93) | -5.44 (-5.75--5.13) |
| Asia | 10427343 (6767578-16267276) | 12809.34 (8296.96-20116.58) | 7255591 (4428888-11495410) | 2962.55 (1809.36-4719.75) | -4.6 (-4.72--4.48) |
| Australasia | 2284 (1981-2626) | 180.25 (154.73-209.52) | 4078 (3463-4587) | 126.33 (106.98-143.25) | -0.28 (-0.96-0.41) |
| Basic Health System | 1734324 (895552-2794707) | 2852.81 (1476.5-4604.81) | 1020734 (603905-1343377) | 571.62 (339.28-753.57) | -4.95 (-5.18--4.71) |
| Caribbean | 19386 (13577-27437) | 1603.09 (1105.81-2302.94) | 18368 (12037-27444) | 716.96 (466.02-1082.03) | -3.03 (-3.33--2.72) |
| Central Africa | 187324 (108817-291690) | 17992.81 (10409.24-28028.39) | 170314 (88058-269325) | 7621.83 (3937.98-12096.25) | -2.8 (-3.22--2.38) |
| Central Asia | 2280 (1981-2599) | 128.03 (111.45-145.41) | 950 (740-1232) | 31.45 (24.47-40.85) | -6.06 (-6.57--5.55) |
| Central Europe | 3010 (2666-3570) | 42.4 (37.52-50.26) | 30209 (26900-33641) | 232.22 (207.4-258.67) | 7.29 (5.77-8.83) |
| Central Latin America | 127238 (119071-135537) | 3670.24 (3434.42-3909.92) | 97917 (87434-111813) | 841.96 (751.83-962.14) | -4.53 (-5.14--3.92) |
| Central Sub-Saharan Africa | 134406 (80759-215200) | 17415.67 (10443.86-27885.36) | 115967 (61116-189957) | 6526.19 (3429.11-10699.67) | -3.17 (-3.74--2.59) |
| Commonwealth High Income | 8241 (7030-9607) | 101.44 (85.25-119.97) | 35706 (30952-39045) | 220.22 (191.11-241.75) | 3.36 (1.51-5.25) |
| Commonwealth Low Income | 752224 (419369-1083606) | 21345.56 (11836.46-30996.4) | 606467 (366996-1109334) | 6152.99 (3716.24-11275.15) | -4.17 (-4.26--4.08) |
| Commonwealth Middle Income | 8894569 (6033983-13696811) | 37990.95 (25700.58-58845.65) | 6418949 (3863540-10426377) | 9416.41 (5679.27-15351.81) | -4.53 (-4.67--4.4) |
| East Asia | 162542 (78121-239629) | 456.66 (220.93-672.23) | 59069 (41208-93861) | 54.3 (38.02-85.7) | -7.05 (-7.4--6.69) |
| East Asia & Pacific - WB | 1585180 (723567-2711299) | 2833.57 (1298.69-4850.33) | 860387 (468707-1162221) | 517.95 (282.72-699.27) | -5.26 (-5.5--5.03) |
| Eastern Africa | 601677 (279155-1007964) | 22795.82 (10519.83-38244.24) | 515701 (260475-763457) | 8758.9 (4436.71-12996.97) | -3.24 (-3.38--3.1) |
| Eastern Europe | 5446 (5042-5942) | 46.47 (42.77-51.02) | 6567 (5763-7524) | 38.77 (33.73-44.83) | -2.18 (-2.97--1.39) |
| Eastern Mediterranean Region | 543589 (255712-1043261) | 7416.66 (3493.91-14264.34) | 328435 (189126-574734) | 1916.31 (1100.79-3346.46) | -4.53 (-4.62--4.43) |
| Eastern Sub-Saharan Africa | 719896 (341874-1193184) | 25866.53 (12239.26-42970.95) | 612754 (309771-898392) | 9961.86 (5052.95-14650.85) | -3.25 (-3.41--3.1) |
| Europe | 60236 (52608-69759) | 112.37 (97.47-131.1) | 239432 (206031-269799) | 253.05 (217.84-286.3) | 3.51 (2.85-4.17) |
| Europe & Central Asia - WB | 61778 (54133-71243) | 113 (98.39-131.22) | 239684 (206326-270000) | 247.87 (213.5-280.34) | 3.39 (2.74-4.03) |
| European Region | 62508 (54765-72024) | 113.57 (98.87-131.78) | 245048 (211100-276234) | 250.92 (216.28-283.99) | 3.41 (2.77-4.06) |
| High-income Asia Pacific | 22236 (17965-26965) | 223.83 (180.43-272.92) | 71425 (57602-87262) | 243.76 (196.16-299.82) | 0.74 (0.54-0.95) |
| High-income North America | 10835 (9301-12449) | 56.5 (47.89-65.98) | 123515 (107037-132844) | 331.51 (290.81-355.45) | 7.94 (5.98-9.94) |
| Latin America & Caribbean - WB | 239765 (218144-261576) | 2063.86 (1876.89-2253.13) | 211867 (188416-240559) | 623.83 (554.61-709.76) | -3.73 (-4.2--3.26) |
| Limited Health System | 10189765 (6769519-15742669) | 34301.27 (22726.2-53235.08) | 7341743 (4458605-11786421) | 8656.88 (5259.65-13950.61) | -4.5 (-4.62--4.37) |
| Middle East & North Africa - WB | 39085 (20533-73743) | 801.85 (421.8-1507.18) | 41074 (27320-66448) | 300.6 (199.8-484.9) | -2.96 (-3.16--2.77) |
| Minimal Health System | 497520 (272952-770347) | 21914.35 (11961.72-34007.24) | 434080 (226718-675421) | 9977.81 (5202.58-15519.43) | -2.62 (-2.85--2.4) |
| North Africa and Middle East | 53828 (27642-106192) | 807.76 (415.88-1586.83) | 48425 (29560-79937) | 261.87 (160.84-430.12) | -3.63 (-3.81--3.46) |
| North America | 10841 (9306-12457) | 56.52 (47.91-66.02) | 123512 (107039-132841) | 331.47 (290.78-355.4) | 7.94 (5.98-9.94) |
| Northern Africa | 27576 (15265-50775) | 1113.98 (615.02-2040.93) | 20988 (13155-36243) | 340.25 (213.27-585.26) | -3.71 (-3.88--3.54) |
| Oceania | 11633 (6107-17916) | 11578.58 (6072.1-17869.28) | 15651 (8297-24651) | 6064.46 (3206.28-9551.82) | -1.74 (-1.94--1.55) |
| Region of the Americas | 250046 (228173-272798) | 829.77 (756.78-906.07) | 334031 (295785-366595) | 475.49 (423.35-522.45) | -1.06 (-1.42--0.7) |
| South-East Asia Region | 9598324 (6339619-14940607) | 37305.99 (24591.35-58350.59) | 6748349 (4055467-10769035) | 8358.25 (5035.28-13400.2) | -4.79 (-4.88--4.7) |
| South Asia | 8794652 (6011185-13436237) | 41205.91 (28069.64-63384.31) | 6359884 (3923194-10381288) | 9667.7 (5973.51-15861.75) | -4.73 (-4.85--4.61) |
| South Asia - WB | 8822682 (6029672-13483276) | 39981.23 (27232.37-61521.38) | 6371843 (3930164-10397680) | 9439.87 (5832.27-15483.14) | -4.71 (-4.83--4.58) |
| Southeast Asia | 1415890 (617951-2494535) | 14329.96 (6278.99-25289.37) | 723951 (360011-1014551) | 2635.15 (1317.51-3687.5) | -5.2 (-5.46--4.93) |
| Southern Africa | 250644 (137799-394646) | 14722.01 (8064.22-23256.11) | 238930 (128854-347174) | 6545.13 (3528.88-9520.07) | -2.54 (-2.75--2.32) |
| Southern Latin America | 9901 (9084-10730) | 429.84 (392.82-467.46) | 14900 (13133-16411) | 312.45 (276.47-343.97) | -0.25 (-0.46--0.03) |
| Southern Sub-Saharan Africa | 101539 (54802-169889) | 8965.73 (4813.26-15013.54) | 125607 (69605-194410) | 5286.83 (2934.96-8162.18) | -1.5 (-1.73--1.27) |
| Sub-Saharan Africa - WB | 1766925 (946089-2808201) | 20510.56 (10947.15-32672.52) | 1407173 (749126-2140722) | 7691.53 (4104.89-11677.79) | -3.26 (-3.48--3.04) |
| Tropical Latin America | 64973 (57287-72009) | 1677.64 (1484.22-1858.63) | 68337 (59920-74251) | 545.87 (481.38-593.08) | -3.47 (-3.96--2.98) |
| Western Africa | 720976 (383955-1147823) | 22577.56 (11953.9-36091.84) | 479356 (273878-746163) | 7414.96 (4237.72-11546.53) | -3.74 (-4--3.48) |
| Western Europe | 47083 (40750-54108) | 146.24 (124.96-170.27) | 200021 (171888-226182) | 348.31 (298.42-396.82) | 3.81 (2.9-4.72) |
| Western Pacific Region | 333663 (170922-528047) | 679.61 (349.49-1076.57) | 230535 (162673-329719) | 155.44 (109.46-223.24) | -4.62 (-4.84--4.4) |
| Western Sub-Saharan Africa | 802567 (425222-1272498) | 22716.69 (11963.61-36204.57) | 548841 (312291-853582) | 7601.91 (4330.86-11816.16) | -3.67 (-3.92--3.42) |
| World Bank High Income | 96719 (84058-109928) | 138.98 (120.06-159.25) | 444561 (382917-491641) | 316.84 (274.62-350.52) | 3.88 (3.01-4.76) |
| World Bank Low Income | 939597 (487594-1541816) | 17077.16 (8815.6-28079.89) | 772653 (407738-1164594) | 6383.23 (3375.99-9628.46) | -3.39 (-3.56--3.22) |
| World Bank Lower Middle Income | 10921886 (7054913-16948915) | 27363.94 (17632-42726.96) | 7573076 (4543415-12018863) | 7071.32 (4245.1-11269.46) | -4.31 (-4.42--4.21) |
| World Bank Upper Middle Income | 565257 (388659-887501) | 894.45 (616.99-1401.05) | 451759 (335393-617089) | 258.49 (192.78-352.79) | -3.87 (-4.18--3.56) |
| Country |  |  |  |  |  |
| Afghanistan | 1892 (510-5037) | 671.59 (186.02-1776.91) | 561 (154-1465) | 166.8 (48.32-429.53) | -5.2 (-5.73--4.66) |
| Albania | 68 (37-113) | 76.4 (41.65-127.99) | 120 (66-203) | 49.54 (27.01-84.99) | -1.55 (-1.88--1.23) |
| Algeria | 1437 (804-2709) | 259.44 (142.69-487.07) | 2780 (1620-4682) | 179.32 (104.75-300.12) | -1.18 (-1.3--1.07) |
| American Samoa | 29 (17-47) | 3498.04 (2032.77-5721.77) | 59 (32-96) | 2888.18 (1556.85-4710.51) | -0.34 (-0.58--0.1) |
| Andorra | 2 (1-2) | 65.37 (45.24-92.25) | 5 (4-7) | 67.21 (46.87-93.42) | 0.48 (-0.08-1.05) |
| Angola | 41416 (23572-67744) | 33792.47 (19148.26-55284.73) | 31796 (16585-49946) | 8409.07 (4372.57-13219.45) | -4.46 (-4.8--4.11) |
| Antigua and Barbuda | 24 (21-26) | 797.5 (713.82-883.06) | 14 (13-16) | 298.93 (262.88-339.67) | -3.6 (-4.29--2.91) |
| Argentina | 5095 (4591-5602) | 317.57 (284.78-351.06) | 8514 (7452-9475) | 278.35 (243.73-309.99) | 0.31 (0.11-0.51) |
| Armenia | 120 (104-137) | 127.16 (110.84-144.62) | 43 (35-53) | 20.79 (16.72-26.43) | -7.32 (-7.83--6.82) |
| Australia | 1796 (1549-2083) | 170.38 (145.21-199.93) | 2609 (2194-2930) | 95.23 (79.74-108.02) | -1.34 (-2.14--0.53) |
| Austria | 844 (620-1117) | 136.29 (98.51-181.89) | 2113 (1754-2448) | 193.18 (158.81-226.98) | 1.54 (0.95-2.12) |
| Azerbaijan | 179 (91-292) | 102.16 (52.58-166.69) | 189 (98-344) | 53.81 (27.85-97.85) | -2.63 (-2.9--2.37) |
| Bahamas | 32 (28-36) | 484.68 (430.34-548.42) | 31 (26-37) | 184.34 (151.87-222.89) | -3.62 (-4.33--2.91) |
| Bahrain | 21 (12-32) | 376.16 (214.37-587.78) | 83 (57-119) | 315.06 (214.17-453.05) | -0.65 (-0.79--0.51) |
| Bangladesh | 420006 (237044-600332) | 21184.29 (11942.59-30710.78) | 331868 (152285-753366) | 5015.85 (2263.86-11524.95) | -4.91 (-5.08--4.73) |
| Barbados | 83 (76-91) | 490.14 (443.49-542.99) | 54 (45-64) | 199.37 (165.29-237.75) | -3.2 (-3.73--2.66) |
| Belarus | 157 (131-186) | 28.57 (23.53-34.26) | 253 (190-336) | 33.55 (24.77-44.92) | -0.29 (-1.07-0.5) |
| Belgium | 1919 (1657-2195) | 225.66 (193.34-260.15) | 13107 (10871-14993) | 905.33 (752.5-1039.78) | 5.65 (4.79-6.51) |
| Belize | 51 (47-57) | 1158.66 (1047.83-1285.38) | 53 (46-60) | 436.86 (377.31-498.67) | -3.78 (-4.27--3.28) |
| Benin | 24767 (13526-37763) | 28482.87 (15472.49-43862.28) | 17878 (8641-30810) | 9083.44 (4383.59-15711.97) | -3.73 (-3.82--3.64) |
| Bermuda | 11 (10-12) | 360.74 (322.66-399.01) | 9 (8-11) | 132.67 (105.35-165.12) | -3.41 (-3.99--2.83) |
| Bhutan | 3093 (1369-5407) | 36729.31 (16163.58-64633.67) | 1817 (688-4115) | 6336.97 (2386.73-14514.44) | -5.9 (-6.27--5.52) |
| Bolivia (Plurinational State of) | 3301 (1523-5746) | 2517.42 (1154.45-4407.52) | 3215 (1745-5977) | 791.77 (426.86-1479.08) | -3.88 (-3.98--3.79) |
| Bosnia and Herzegovina | 143 (78-267) | 92.75 (50.43-171.84) | 346 (115-777) | 101.07 (33.64-225.35) | 0.9 (0.54-1.27) |
| Botswana | 3012 (1432-5165) | 13991.49 (6642.72-24028.8) | 2846 (1371-4628) | 5248.14 (2532.55-8592.1) | -2.94 (-3.08--2.8) |
| Brazil | 62826 (55511-70007) | 1665.68 (1476.93-1855.15) | 67448 (59154-73369) | 550.57 (485.67-598.91) | -3.41 (-3.91--2.92) |
| Brunei Darussalam | 8 (5-13) | 200.16 (116.32-334.94) | 19 (9-30) | 147.05 (72.51-233.81) | -0.46 (-0.62--0.3) |
| Bulgaria | 171 (149-197) | 28.25 (24.56-32.56) | 1091 (911-1292) | 125.95 (104.59-150.58) | 6.12 (4.45-7.83) |
| Burkina Faso | 46930 (24977-76152) | 26745.83 (14128.16-43627.6) | 35509 (18528-55059) | 9647.27 (5021.86-15087.89) | -3.41 (-3.51--3.32) |
| Burundi | 19672 (9225-31421) | 20768.81 (9686.56-33481.26) | 20645 (9511-34492) | 12363.04 (5690.21-20690.74) | -1.83 (-1.92--1.73) |
| Cabo Verde | 859 (454-1354) | 7158.72 (3785.59-11412.4) | 353 (189-652) | 1934.78 (1044.82-3554.69) | -4.52 (-4.85--4.18) |
| Cambodia | 22024 (10586-38623) | 12905.92 (6204.87-22716.78) | 9985 (5070-15551) | 1998.88 (1018.34-3133.64) | -6.33 (-6.53--6.13) |
| Cameroon | 24995 (12682-42793) | 15268.69 (7683.56-26310.95) | 23472 (11237-38960) | 5206.25 (2498.01-8657.97) | -3.45 (-3.69--3.21) |
| Canada | 1278 (1086-1479) | 77.21 (64.03-91.21) | 15131 (12770-16991) | 351.37 (296.51-396.24) | 7.12 (4.99-9.3) |
| Central African Republic | 7988 (4408-13286) | 21902.03 (12073.19-36440.04) | 10595 (5270-17031) | 16855.25 (8380.2-27122.92) | -0.63 (-0.74--0.51) |
| Chad | 49409 (24308-81475) | 38728.64 (18949.44-64442.56) | 41938 (23297-66704) | 19651.48 (10874.11-31415.62) | -2.28 (-2.42--2.13) |
| Chile | 3527 (3174-3877) | 739.6 (663.53-817.2) | 4624 (4011-5131) | 335.66 (291.41-373.75) | -1.75 (-2.06--1.45) |
| China | 159485 (75220-236215) | 464.62 (220.58-687.19) | 50410 (33407-84169) | 47.85 (31.82-79.25) | -7.55 (-7.86--7.23) |
| Colombia | 8969 (8136-9733) | 1244.37 (1127.4-1355.51) | 11431 (9684-13257) | 420.78 (354.48-491.08) | -4.37 (-4.94--3.79) |
| Comoros | 728 (358-1180) | 9901.47 (4870.79-16081.15) | 957 (401-1693) | 4901 (2077.76-8753.39) | -2.07 (-2.2--1.94) |
| Congo | 5323 (2562-9287) | 13313.91 (6367.15-23261.82) | 5331 (2233-10273) | 6011.22 (2517.91-11598.47) | -2.41 (-2.69--2.13) |
| Cook Islands | 4 (2-7) | 803.12 (456.17-1297.9) | 8 (5-12) | 619.34 (371.64-906.98) | -0.67 (-0.94--0.4) |
| Costa Rica | 1131 (1008-1261) | 1408.85 (1254.01-1577.01) | 1432 (1218-1670) | 534.82 (453.51-626.18) | -3.13 (-3.59--2.67) |
| Croatia | 129 (114-146) | 47.73 (42.22-54.05) | 893 (756-1039) | 164.87 (139.45-191.82) | 5.59 (3.88-7.32) |
| Cuba | 4525 (4121-4900) | 872.74 (791.61-950.75) | 3482 (3013-4012) | 360.54 (309.51-417.69) | -4.29 (-5.07--3.51) |
| Cyprus | 265 (149-492) | 574.5 (327.09-1053.48) | 702 (459-974) | 582.6 (383.34-814.08) | 0.49 (0.28-0.7) |
| Czechia | 530 (462-609) | 75.93 (66.19-87.36) | 6110 (5074-7179) | 462.59 (384.48-543.98) | 8.76 (7.03-10.53) |
| C么te d'Ivoire | 16033 (8580-24260) | 12722.5 (6795.85-19297.42) | 18697 (9377-31433) | 4930.25 (2482.43-8276.57) | -2.67 (-2.88--2.46) |
| Democratic People's Republic of Korea | 560 (346-883) | 94.59 (58.57-148.65) | 1658 (1157-2395) | 121.71 (84.38-174.15) | 1.56 (1.23-1.88) |
| Democratic Republic of the Congo | 74968 (44337-122966) | 13838.37 (8166.5-22711.72) | 66463 (27664-118079) | 5574.31 (2317.53-9921.8) | -3.03 (-3.8--2.25) |
| Denmark | 1301 (1149-1470) | 270.61 (236.59-308.51) | 6088 (5280-6956) | 807.38 (697.41-926.31) | 4.69 (3.75-5.63) |
| Djibouti | 597 (298-957) | 14773.45 (7364.77-23750.52) | 1110 (481-1907) | 5459.13 (2375.41-9404.36) | -3.21 (-3.32--3.09) |
| Dominica | 25 (12-36) | 771.95 (392.83-1127.12) | 14 (8-23) | 350.16 (191.34-571.22) | -2.63 (-2.93--2.34) |
| Dominican Republic | 2451 (1284-4170) | 1531.53 (803.17-2627.06) | 3666 (1901-6419) | 775.78 (404.19-1361.11) | -2.43 (-2.55--2.32) |
| Ecuador | 6360 (5770-6899) | 2799.63 (2531.74-3047.16) | 2107 (1775-2529) | 265.45 (221.88-320.29) | -7.86 (-8.31--7.41) |
| Egypt | 12210 (6078-25007) | 1285.44 (638.72-2625.34) | 8670 (5143-16779) | 389.17 (230.84-751.82) | -3.74 (-3.95--3.52) |
| El Salvador | 4875 (2600-7871) | 3634.99 (1925.41-5831.37) | 2442 (1468-4210) | 774.02 (461.21-1326.4) | -5.22 (-5.68--4.75) |
| Equatorial Guinea | 1899 (942-3559) | 27622.34 (13633.42-51980.81) | 536 (253-903) | 3075.34 (1456.06-5212.85) | -7.97 (-8.38--7.56) |
| Eritrea | 9988 (4047-17634) | 34644.81 (14048.93-61098.55) | 10763 (3641-20563) | 12499.86 (4236.89-23979.94) | -3.25 (-3.34--3.16) |
| Estonia | 28 (24-33) | 30.69 (25.65-36.64) | 60 (44-80) | 41.93 (30.35-57.02) | 0.5 (-0.43-1.44) |
| Eswatini | 1929 (977-3023) | 18162.11 (9183.19-28690.73) | 1234 (594-2083) | 5962.98 (2877.9-10109.09) | -3.53 (-3.74--3.32) |
| Ethiopia | 265916 (96102-493062) | 37679.32 (13604.06-69839.8) | 195012 (98656-289202) | 11408.38 (5803.55-17043.28) | -4.21 (-4.4--4.01) |
| Fiji | 949 (538-1607) | 7580.68 (4299.31-12829.32) | 1279 (664-2278) | 4240.53 (2198.66-7538.74) | -1.71 (-1.93--1.48) |
| Finland | 664 (565-783) | 165.99 (138.94-198.79) | 1408 (1180-1613) | 174.95 (146.13-202.37) | 0.75 (0.03-1.47) |
| France | 15546 (13570-17643) | 328.1 (284.33-375.65) | 22225 (18552-26199) | 268.29 (221.15-321.49) | -0.62 (-1.43-0.2) |
| Gabon | 2812 (1289-5163) | 11427.38 (5205.49-21174.85) | 1248 (513-2515) | 3266.27 (1343.98-6616.59) | -4.1 (-4.21--4) |
| Gambia | 1865 (1034-3051) | 13830.02 (7660.75-22627.4) | 2064 (995-3694) | 5355.5 (2585-9627.35) | -3.06 (-3.18--2.94) |
| Georgia | 227 (195-264) | 87.76 (75.35-102.18) | 69 (54-86) | 23.85 (18.54-30.24) | -4.81 (-5.4--4.22) |
| Germany | 10216 (8806-11727) | 150.68 (127.82-176.73) | 50763 (42772-58048) | 429.69 (361.91-494.08) | 5.49 (4.31-6.69) |
| Ghana | 35959 (19551-62690) | 15728.7 (8517.84-27436.09) | 34191 (18215-56292) | 5461.41 (2902.9-8999.84) | -3.39 (-3.47--3.31) |
| Greece | 503 (361-693) | 67.69 (47.15-94.81) | 1315 (1101-1533) | 93.21 (75.72-113.33) | 1.57 (1.2-1.95) |
| Greenland | 4 (2-8) | 390 (219.07-697.29) | 10 (4-20) | 361.42 (126.97-714.75) | 0.13 (-0.03-0.29) |
| Grenada | 24 (22-28) | 626.51 (552.48-709.3) | 12 (10-13) | 229.46 (198.31-264.71) | -3.95 (-4.49--3.41) |
| Guam | 33 (19-55) | 1240.67 (714.65-2024.44) | 79 (48-128) | 746.73 (454.38-1195.78) | -1.15 (-1.49--0.82) |
| Guatemala | 22839 (21024-24442) | 17602.71 (16171.07-18887.02) | 15075 (12965-17484) | 3018.27 (2581.6-3515.21) | -5.45 (-6.04--4.85) |
| Guinea | 55705 (23859-107037) | 38074.83 (16259.21-73076.31) | 23715 (11590-38084) | 10482.28 (5108.29-16998.7) | -4.14 (-4.3--3.98) |
| Guinea-Bissau | 4239 (2358-6615) | 28513.29 (15770.24-44806.76) | 2318 (1186-3759) | 9765.41 (4993.21-15854.98) | -3.42 (-3.68--3.17) |
| Guyana | 555 (486-625) | 3586.28 (3121.62-4070.8) | 333 (257-426) | 1279.03 (982.48-1637.22) | -3.66 (-4.27--3.05) |
| Haiti | 8250 (3544-14740) | 6753.01 (2887.37-12093.46) | 7232 (2863-14491) | 2751.55 (1086.46-5512.54) | -2.71 (-2.99--2.42) |
| Honduras | 6567 (3742-11285) | 7827.08 (4450.01-13422.64) | 8450 (4555-14048) | 3110.3 (1675.86-5186.9) | -2.94 (-3.11--2.78) |
| Hungary | 344 (293-399) | 46.62 (39.96-53.88) | 4172 (3513-4890) | 364.96 (308.9-427.4) | 9.16 (7.53-10.81) |
| Iceland | 21 (17-25) | 133.18 (105.72-166.69) | 84 (71-100) | 254.35 (209.51-306.74) | 3.3 (2.35-4.25) |
| India | 7827838 (5331100-12041989) | 47127.1 (31968.88-72934.25) | 5739336 (3462316-9400221) | 10719.25 (6485.25-17614.25) | -4.8 (-4.91--4.69) |
| Indonesia | 1046208 (443909-1797754) | 29478.46 (12548.97-50694.66) | 498280 (224660-761222) | 5440.42 (2465.3-8294.52) | -5.04 (-5.33--4.75) |
| Iran (Islamic Republic of) | 4442 (2273-9088) | 465.94 (238.32-953.33) | 6127 (3664-10545) | 185.38 (111.55-315.6) | -2.76 (-2.98--2.54) |
| Iraq | 2134 (1059-4397) | 606.77 (304.49-1237.58) | 2202 (1306-4046) | 243.43 (145-444.46) | -3.2 (-3.36--3.04) |
| Ireland | 189 (154-230) | 85.94 (67.78-107.33) | 919 (762-1098) | 205.3 (167.69-249.43) | 4.33 (3.4-5.27) |
| Israel | 536 (460-619) | 201.41 (170.79-235.09) | 4982 (4165-5698) | 678.77 (566.18-781.35) | 5.02 (3.93-6.11) |
| Italy | 2629 (2084-3308) | 56.81 (44.34-72.35) | 26123 (21522-29346) | 277.89 (230.64-313.14) | 7.42 (6.38-8.47) |
| Jamaica | 1297 (1181-1405) | 1362.82 (1232.58-1485.81) | 877 (700-1087) | 582.86 (462.16-726.2) | -2.29 (-3.09--1.49) |
| Japan | 16170 (13641-18893) | 186.52 (156.19-220.06) | 56788 (47319-65328) | 241.59 (200.75-280.94) | 1.17 (0.91-1.43) |
| Jordan | 103 (60-162) | 222.98 (129.4-349.86) | 373 (237-575) | 134.76 (85.47-207.16) | -1.74 (-1.84--1.63) |
| Kazakhstan | 578 (503-654) | 121 (105.55-136.42) | 162 (129-201) | 23.1 (18.37-28.87) | -7.55 (-8.35--6.74) |
| Kenya | 65696 (33829-105029) | 20381.43 (10471.85-32680.04) | 66539 (31306-104799) | 7803.85 (3680.22-12296.8) | -2.7 (-2.98--2.42) |
| Kiribati | 417 (210-635) | 30297.99 (15271.9-46162.57) | 341 (162-522) | 14143.18 (6751.08-21733.45) | -2.2 (-2.43--1.97) |
| Kuwait | 20 (17-23) | 110.56 (94.59-128.07) | 66 (52-84) | 74.34 (57.84-94.7) | 0.59 (-0.13-1.31) |
| Kyrgyzstan | 192 (167-223) | 164.91 (143.46-190.53) | 41 (32-50) | 23.73 (18.88-29.49) | -7.63 (-8.22--7.03) |
| Lao People's Democratic Republic | 13294 (6466-28072) | 16286.76 (7910.64-34411.32) | 4037 (2249-7384) | 2297.57 (1281.53-4189.37) | -6.54 (-6.69--6.38) |
| Latvia | 41 (35-50) | 25.85 (21.42-31.54) | 53 (41-69) | 25.59 (19.18-34.13) | -0.46 (-1.18-0.27) |
| Lebanon | 478 (280-779) | 532.91 (311.77-872.59) | 1274 (796-1957) | 379.47 (239.67-579.1) | -0.72 (-0.98--0.46) |
| Lesotho | 10776 (5401-17477) | 29164.95 (14648.86-47594.68) | 5454 (2186-10009) | 12527.39 (5016.71-23017.4) | -2.54 (-2.69--2.38) |
| Liberia | 8323 (4717-14007) | 16122.67 (9112.07-27291.88) | 5136 (2611-8625) | 7105.26 (3604.61-11961.95) | -3.21 (-3.56--2.87) |
| Libya | 641 (255-1574) | 815.25 (326.67-1998.68) | 515 (316-904) | 264.31 (163.2-458.98) | -3.12 (-3.62--2.62) |
| Lithuania | 78 (66-92) | 38.6 (32.67-45.66) | 236 (188-297) | 75.41 (59.54-96.43) | 1.56 (0.23-2.9) |
| Luxembourg | 61 (53-70) | 204.48 (175.4-239.84) | 302 (257-347) | 502.82 (426.77-582.31) | 3.71 (2.92-4.52) |
| Madagascar | 30934 (18643-55766) | 16117.74 (9694.21-29099.38) | 30871 (13207-61509) | 8885.31 (3803.78-17698.49) | -1.59 (-1.74--1.44) |
| Malawi | 42557 (21092-73771) | 29390.5 (14540.66-51088.91) | 34788 (17459-53774) | 12497.21 (6270.7-19365.52) | -2.94 (-3.08--2.8) |
| Malaysia | 6784 (4135-12506) | 1802.5 (1094-3315.43) | 14295 (7895-21050) | 1129.59 (630.7-1664.99) | -1.7 (-1.86--1.53) |
| Maldives | 233 (113-416) | 8063.22 (3892.48-14411.49) | 104 (63-155) | 847.32 (517.71-1265.7) | -7.06 (-7.97--6.15) |
| Mali | 46746 (26429-70243) | 31301.25 (17718-47115.73) | 36455 (15449-71515) | 11003.84 (4663.4-21666.81) | -3.36 (-3.51--3.22) |
| Malta | 13 (11-17) | 65.75 (50.53-84.72) | 70 (57-83) | 117.82 (93.55-144.16) | 2.48 (1.74-3.23) |
| Marshall Islands | 50 (26-87) | 7380.46 (3808.52-13044.58) | 37 (22-65) | 3248.8 (1909.26-5666.29) | -2.34 (-2.58--2.11) |
| Mauritania | 6902 (3728-10727) | 15482.14 (8367.11-24264.05) | 3982 (2110-6592) | 4460.16 (2379.98-7384.22) | -4 (-4.13--3.87) |
| Mauritius | 428 (387-470) | 1418.79 (1280.03-1559.26) | 625 (564-687) | 699.2 (628.65-770.25) | -0.67 (-1.13--0.2) |
| Mexico | 71640 (67735-74925) | 3947.66 (3737.36-4129.27) | 43958 (39385-48986) | 760.67 (680.5-848.84) | -4.78 (-5.62--3.93) |
| Micronesia (Federated States of) | 138 (73-241) | 6409.88 (3398.86-11238.06) | 80 (46-140) | 3227.37 (1861.88-5667.45) | -1.85 (-2.29--1.41) |
| Monaco | 3 (2-5) | 72.34 (46.76-112.45) | 5 (4-8) | 95.84 (63.05-134.36) | 1.31 (0.71-1.91) |
| Mongolia | 14 (5-26) | 32.29 (11.26-60.57) | 5 (4-9) | 7.41 (4.88-11.72) | -5.41 (-5.81--5.01) |
| Montenegro | 4 (2-6) | 13.17 (8.44-20.15) | 7 (4-10) | 13.01 (8.52-19.19) | 0.1 (0.04-0.15) |
| Morocco | 5643 (1794-11658) | 885.76 (284.99-1827.84) | 3716 (2107-6338) | 248.15 (141.48-421.03) | -4.01 (-4.18--3.83) |
| Mozambique | 40128 (19553-66058) | 18125.67 (8794.8-30008.11) | 29372 (13065-52260) | 7440.63 (3306.17-13241.84) | -2.74 (-2.88--2.6) |
| Myanmar | 105884 (39225-230967) | 11775.37 (4348.05-25831.68) | 38551 (19689-60565) | 1885.78 (963.31-2966.57) | -6.03 (-6.29--5.76) |
| Namibia | 4640 (2377-7614) | 18267.54 (9350.39-30004.72) | 3440 (1676-5804) | 6455.05 (3156.63-10950.92) | -3.32 (-3.42--3.22) |
| Nauru | 6 (3-11) | 3931.18 (2146.19-6882.99) | 5 (3-10) | 2415.11 (1241.66-4660.93) | -1.41 (-1.64--1.17) |
| Nepal | 72293 (41985-118164) | 21054.12 (12177.52-34444.05) | 41252 (21018-80073) | 3931.14 (2002.12-7639.34) | -5.59 (-5.7--5.49) |
| Netherlands | 1261 (1031-1563) | 119.36 (95.19-151.69) | 8765 (7332-10183) | 410.6 (339.78-484.94) | 4.66 (3.61-5.72) |
| New Zealand | 488 (420-573) | 229.14 (194.79-273.26) | 1469 (1220-1682) | 295.68 (244.72-342.69) | 3.13 (2.08-4.18) |
| Nicaragua | 1685 (890-2658) | 2663.56 (1408.21-4197.46) | 1313 (821-2171) | 624.65 (389.06-1036.49) | -4.62 (-4.76--4.48) |
| Niger | 38703 (18825-65874) | 40039.47 (19433.36-68358.98) | 47283 (24785-77849) | 15656.56 (8199.72-26006.68) | -3.26 (-3.6--2.91) |
| Nigeria | 376929 (194256-631618) | 20648.52 (10618.48-34700.99) | 211925 (126118-355264) | 6431.25 (3817.62-10792.02) | -4.01 (-4.41--3.62) |
| Niue | 5 (3-8) | 3641.73 (2059.26-6535.22) | 2 (1-4) | 1962.29 (1115.54-3504.3) | -2.02 (-2.4--1.64) |
| North Macedonia | 185 (101-309) | 231.23 (126.12-385.44) | 323 (140-594) | 189.42 (82.54-348.21) | -0.2 (-0.45-0.04) |
| Northern Mariana Islands | 11 (7-18) | 2465.85 (1467.13-4001.22) | 45 (26-69) | 2327.45 (1341.43-3565.03) | 0.2 (0-0.39) |
| Norway | 914 (771-1090) | 215.45 (179.01-261.03) | 6104 (5151-6992) | 980.39 (822.34-1135.43) | 6.02 (4.99-7.05) |
| Oman | 161 (84-272) | 644.5 (335.64-1086.92) | 232 (163-328) | 377.31 (265.3-531.13) | -1.58 (-2.09--1.07) |
| Pakistan | 471422 (201389-905830) | 18828.52 (8017.67-36610.12) | 245611 (127005-448624) | 5298.4 (2733.5-9699.63) | -4.29 (-4.47--4.1) |
| Palau | 27 (14-47) | 6194.59 (3268.51-10732.79) | 36 (19-63) | 4177.35 (2183.78-7190.54) | -0.81 (-1.02--0.61) |
| Palestine | 163 (82-254) | 421.92 (212.98-663.54) | 104 (62-150) | 109.66 (66.36-158.42) | -4.26 (-4.6--3.91) |
| Panama | 1227 (1087-1375) | 1806.51 (1596.15-2034.95) | 1154 (941-1386) | 529.37 (429.85-636.24) | -3.46 (-3.87--3.05) |
| Papua New Guinea | 8384 (4205-13061) | 14115.14 (7076.46-22013.98) | 12039 (6209-19198) | 7318.12 (3768.04-11684.29) | -1.71 (-1.89--1.52) |
| Paraguay | 2147 (1300-4445) | 2129.67 (1293.47-4357.24) | 888 (446-1454) | 332.03 (167.81-543.28) | -6.17 (-6.56--5.79) |
| Peru | 9272 (5236-13400) | 1809.28 (1024.47-2627.25) | 7655 (4979-12648) | 481.35 (312.98-798.11) | -4.89 (-5.26--4.52) |
| Philippines | 45724 (22000-102686) | 3948.23 (1895.51-8844.92) | 42485 (25586-77734) | 1284.62 (774.89-2341.11) | -3.47 (-3.56--3.39) |
| Poland | 537 (495-578) | 24.93 (23.04-26.82) | 11186 (9858-12403) | 267.16 (236.32-295.88) | 10.01 (8.1-11.96) |
| Portugal | 562 (487-654) | 76.21 (65.21-89.93) | 4391 (3719-4988) | 279.8 (236.11-320.87) | 5.74 (5.09-6.4) |
| Puerto Rico | 537 (487-587) | 288.8 (260.42-317.64) | 1322 (1118-1556) | 313.65 (263.75-372.36) | 0.03 (-0.57-0.62) |
| Qatar | 5 (3-8) | 216.51 (134.63-334.06) | 41 (29-57) | 227.89 (162.01-314.81) | 0.22 (0.01-0.44) |
| Republic of Korea | 5781 (2555-9086) | 487.4 (217.24-763.89) | 13988 (6857-25875) | 275.39 (133.96-512.54) | -1.15 (-1.73--0.56) |
| Republic of Moldova | 130 (111-148) | 70.61 (60.45-81.06) | 114 (91-139) | 40.6 (32.36-49.9) | -2.96 (-3.69--2.22) |
| Romania | 502 (441-568) | 38.97 (34.08-44.2) | 4337 (3635-5048) | 199.48 (166.35-233.6) | 6.4 (4.42-8.41) |
| Russian Federation | 4454 (4128-4849) | 60.1 (55.39-65.89) | 5247 (4660-5905) | 45.42 (40.07-51.48) | -2.53 (-3.28--1.78) |
| Rwanda | 12726 (4197-24957) | 12245.76 (4038.13-24050.53) | 10857 (4224-21216) | 4701.21 (1833.86-9214.64) | -3.67 (-3.97--3.36) |
| Saint Kitts and Nevis | 58 (53-64) | 2601.16 (2351.23-2880.25) | 23 (19-27) | 853.01 (713.35-998.2) | -3.56 (-4.19--2.92) |
| Saint Lucia | 54 (49-60) | 1203.14 (1082.79-1345.69) | 42 (35-49) | 362.18 (299.58-429.89) | -4.51 (-5.36--3.65) |
| Saint Vincent and the Grenadines | 64 (57-71) | 1708.02 (1527.31-1913.41) | 33 (29-39) | 479.72 (413.45-555.68) | -4.27 (-5.01--3.52) |
| Samoa | 80 (44-139) | 2239.76 (1238.5-3916.95) | 99 (57-173) | 1587.61 (911.2-2779.22) | -0.98 (-1.24--0.72) |
| San Marino | 2 (1-3) | 85.62 (59.08-130.93) | 3 (2-5) | 81.59 (56.72-113.98) | 0.55 (0.04-1.06) |
| Sao Tome and Principe | 258 (153-406) | 9026.11 (5337.35-14280.99) | 87 (46-145) | 2116.42 (1121.78-3534.54) | -5.14 (-5.51--4.76) |
| Saudi Arabia | 2527 (1222-4535) | 1130.47 (551.17-2023.36) | 2672 (1604-4278) | 519.33 (312.13-831.32) | -2.13 (-2.43--1.84) |
| Senegal | 32941 (18282-52804) | 24865.47 (13766.97-40075.59) | 17262 (9208-27637) | 5576.47 (2981.06-8961.68) | -4.79 (-4.96--4.62) |
| Serbia | 223 (124-418) | 46.78 (26.05-87.56) | 776 (355-1424) | 81.91 (37.83-150.33) | 2.84 (2.37-3.3) |
| Seychelles | 49 (30-91) | 1679.1 (1015.25-3098.16) | 43 (25-65) | 863.08 (506.22-1309.33) | -1.74 (-1.93--1.55) |
| Sierra Leone | 21032 (10402-34190) | 22922.24 (11222.32-37597.42) | 14844 (7011-24922) | 10071.99 (4732.29-17119.75) | -2.75 (-3.16--2.35) |
| Singapore | 278 (252-305) | 298.48 (269.71-327.67) | 629 (529-712) | 142.19 (120.01-160.95) | -1.32 (-1.75--0.9) |
| Slovakia | 99 (56-162) | 33.33 (18.92-54.13) | 188 (91-321) | 35.58 (17.31-60.98) | 0.85 (0.58-1.12) |
| Slovenia | 27 (23-31) | 22.21 (18.97-25.75) | 220 (179-271) | 83.37 (68.18-102.34) | 5.15 (3.64-6.69) |
| Solomon Islands | 465 (256-811) | 10146.57 (5568.56-17683.08) | 492 (283-860) | 4036.75 (2317.23-7015.3) | -2.7 (-2.94--2.45) |
| Somalia | 24000 (10378-44365) | 36505.24 (15709.45-67605.6) | 44037 (19453-77720) | 22852.72 (10098.08-40310.12) | -1.84 (-2.03--1.64) |
| South Africa | 66055 (34925-124913) | 7505.2 (3927.89-14205.85) | 91791 (52593-157534) | 4682.36 (2691.56-8036.3) | -1.44 (-1.67--1.2) |
| South Sudan | 31355 (14734-56369) | 28415.19 (13288.42-51170.16) | 31704 (16042-58581) | 25303.65 (12755.31-47006.21) | -0.41 (-0.52--0.29) |
| Spain | 4004 (3382-4688) | 133.25 (110.57-158.85) | 18673 (15615-21815) | 313.98 (260.74-370.39) | 3.76 (3.25-4.27) |
| Sri Lanka | 25905 (13177-42773) | 5806.97 (2950.54-9679.2) | 11294 (6281-18926) | 834.44 (467.3-1401.55) | -5.87 (-6.37--5.37) |
| Sudan | 9178 (2436-21736) | 2212.04 (592.57-5222.07) | 4984 (1560-12066) | 651.97 (208.73-1569.41) | -4.11 (-4.42--3.8) |
| Suriname | 249 (133-395) | 2328.13 (1256.57-3699.37) | 256 (146-469) | 906.51 (516.57-1657.93) | -2.97 (-3.15--2.79) |
| Sweden | 979 (750-1291) | 107.64 (80.57-144.39) | 12658 (10700-14711) | 885.51 (744.89-1040.99) | 8.63 (7.55-9.72) |
| Switzerland | 1171 (997-1385) | 197.37 (165.15-237.11) | 4402 (3667-5124) | 401.46 (332.09-472.83) | 3.65 (2.98-4.33) |
| Syrian Arab Republic | 692 (351-1278) | 334.32 (169.78-618.62) | 863 (543-1410) | 159.76 (100.86-260.15) | -2.14 (-2.56--1.71) |
| Taiwan (Province of China) | 2496 (2119-2997) | 372.88 (315.51-448.76) | 7001 (5239-9176) | 326.1 (241.39-430.13) | 1.71 (0.31-3.13) |
| Tajikistan | 303 (150-475) | 287.05 (142.4-447.72) | 237 (129-399) | 128.99 (70.32-216.89) | -3.41 (-4.04--2.78) |
| Thailand | 95066 (24027-297570) | 6891.1 (1741.06-21564.78) | 83236 (45833-137508) | 1537.42 (855.36-2511.3) | -4.62 (-5.14--4.09) |
| Timor-Leste | 1238 (443-2909) | 15112.77 (5400.82-35544.97) | 952 (511-1815) | 2358.01 (1261.79-4492.39) | -6.15 (-6.52--5.78) |
| Togo | 9946 (5317-15685) | 21948.68 (11716.18-34707.74) | 11726 (5946-19041) | 8916 (4520.97-14492.15) | -2.75 (-2.96--2.54) |
| Tokelau | 5 (2-8) | 6880.71 (3704.46-12372.01) | 2 (1-4) | 2495.42 (1451-4414.36) | -3.14 (-3.55--2.72) |
| Tonga | 39 (20-67) | 1632.67 (854.4-2828.86) | 41 (21-75) | 1040.17 (533.22-1888.3) | -1.17 (-1.48--0.86) |
| Trinidad and Tobago | 429 (389-472) | 1063.77 (960.65-1170.88) | 274 (221-336) | 284.25 (227.72-350.8) | -4.84 (-5.44--4.23) |
| Tunisia | 742 (406-1419) | 339.2 (185.31-646.16) | 1325 (791-2300) | 213.77 (128.47-367.49) | -1.18 (-1.46--0.9) |
| Turkmenistan | 138 (123-155) | 199.18 (177.93-222.77) | 43 (34-54) | 30.01 (23.9-37.67) | -8.41 (-9.24--7.58) |
| Tuvalu | 18 (10-34) | 6823.77 (3557.63-12546.2) | 14 (8-25) | 2927.86 (1710.64-5221.06) | -2.19 (-2.58--1.8) |
| T眉rkiye | 4790 (2703-9189) | 347.97 (197-661.07) | 7923 (4901-11003) | 174.95 (109.22-243.27) | -1.73 (-2.15--1.31) |
| Uganda | 52066 (17939-118091) | 20421.67 (7004.36-46511.16) | 34930 (15666-58005) | 6572.49 (2961.92-10963.43) | -4.04 (-4.23--3.85) |
| Ukraine | 557 (473-656) | 18.05 (15.19-21.47) | 605 (435-813) | 16.87 (11.93-23.2) | -1.61 (-2.55--0.65) |
| United Arab Emirates | 59 (33-94) | 527.6 (296.67-835.47) | 245 (161-346) | 359.93 (231.43-513.87) | -0.08 (-0.42-0.27) |
| United Kingdom | 3440 (2678-4406) | 70.7 (54-92.06) | 14637 (12777-16457) | 184.84 (160.3-209.91) | 3.2 (0.85-5.62) |
| United Republic of Tanzania | 98013 (51060-148971) | 22799.34 (11850.11-34764.72) | 83269 (45484-144515) | 8619.71 (4673.76-15004.08) | -3.29 (-3.46--3.11) |
| United States of America | 9552 (8164-11053) | 54.41 (45.9-63.97) | 108372 (93850-116379) | 328.89 (288.31-352.1) | 8.04 (6.11-10.01) |
| United States Virgin Islands | 12 (6-18) | 353.84 (180.6-527.03) | 18 (10-32) | 166.46 (92.77-298.54) | -2.41 (-2.52--2.29) |
| Uruguay | 1279 (1156-1412) | 601.07 (541.44-665.94) | 1761 (1546-1967) | 546.83 (479.98-612.91) | 0.54 (0.23-0.84) |
| Uzbekistan | 530 (449-625) | 120.51 (101.98-142.31) | 162 (130-199) | 17.76 (14.28-21.87) | -8.07 (-8.84--7.28) |
| Vanuatu | 231 (122-395) | 10356.49 (5451.81-17728.45) | 290 (150-502) | 4553.69 (2351.05-7894.41) | -2.67 (-2.84--2.5) |
| Venezuela (Bolivarian Republic of) | 8305 (7589-9012) | 2065.3 (1883.76-2249.44) | 12661 (10097-15728) | 895.64 (711.54-1115.02) | -3.1 (-3.58--2.62) |
| Viet Nam | 51004 (18483-113026) | 2766.25 (1013.59-6106.6) | 19054 (9735-31503) | 463.47 (240.4-762.01) | -5.55 (-5.86--5.24) |
| Yemen | 6461 (2560-13249) | 3530.92 (1401.16-7217.02) | 3623 (1540-7484) | 664.04 (284.43-1363.14) | -5.65 (-5.89--5.4) |
| Zambia | 25003 (13268-41534) | 24481.65 (12887.8-40984.12) | 17368 (9380-28480) | 7345.32 (3968.4-12085.3) | -4.2 (-4.71--3.7) |
| Zimbabwe | 15126 (6386-26320) | 9278.67 (3903.65-16263.63) | 20841 (7694-40079) | 8262.85 (3050.36-15920.32) | 0.32 (-0.13-0.77) |

eTable 4. Incidence of diarrhea disease in the elderly at the regional, and national Levels from 1990 to 2021.

| **Location** | **Rate per 100 000(95% UI)** | | | | |
| --- | --- | --- | --- | --- | --- |
|  | **1990** | | **2021** | | **1990-2021** |
|  | **Incidence cases** | **Incidence rate** | **Incidence cases** | **Incidence rate** | **EAPC** |
| GBD region |  |  |  |  |  |
| Advanced Health System | 20721274 (17293503-24607590) | 25952.25 (21517.86-30921.44) | 58940153 (51583001-67733591) | 37458.28 (32624.51-43044.02) | 1.91 (1.13-2.69) |
| Africa | 15950231 (13237291-19354702) | 151591.38 (126152.52-182993.32) | 34275842 (29452253-39276734) | 147788.69 (127080.63-168881.82) | -0.25 (-0.36--0.14) |
| African Region | 15408096 (12793238-18699033) | 185121.57 (154139.03-223503.33) | 32224040 (27661455-36920724) | 179455.34 (154217.69-205036.32) | -0.28 (-0.4--0.17) |
| America | 12414710 (10420927-14765109) | 43585.11 (36616.2-51637.29) | 18715116 (16004675-21581753) | 28238.89 (24189.79-32469.98) | -1.67 (-2.04--1.31) |
| Andean Latin America | 1242078 (1081204-1465908) | 148298.55 (129659.88-174038.25) | 1579121 (1335556-1816182) | 59485.4 (50507.57-68201.81) | -3.89 (-4.36--3.42) |
| Asia | 85848166 (69122137-105450534) | 109550.63 (88463.52-134097.51) | 289973661 (245782601-335537189) | 126645.6 (107482.36-146223.58) | 0.53 (0.44-0.62) |
| Australasia | 533459 (428529-663294) | 45392.24 (36360.89-56340.81) | 601722 (515822-707443) | 20654.89 (17564.85-24349.02) | -2.59 (-3.94--1.23) |
| Basic Health System | 32908500 (26820254-40259213) | 56569.46 (46215.94-68901.03) | 65024488 (55099130-75997100) | 38414.67 (32610.23-44791.82) | -1.35 (-1.41--1.29) |
| Caribbean | 501823 (444039-562614) | 44040.34 (38960.5-49214.36) | 1196256 (1036938-1376864) | 48946.71 (42407.31-56092.68) | -0.14 (-0.3-0.02) |
| Central Africa | 1173818 (1004713-1364220) | 117045.84 (100338.17-135855.52) | 3493754 (3049921-3936581) | 161449.9 (141028.19-181886.3) | 1.01 (0.87-1.15) |
| Central Asia | 83344 (68772-98314) | 5127.03 (4188-6073.07) | 136337 (108711-164740) | 4621.13 (3660.22-5600.93) | -0.13 (-0.31-0.04) |
| Central Europe | 108274 (84780-135884) | 1578.41 (1234.23-1982.06) | 845577 (757410-950235) | 6789.32 (6079.43-7643.41) | 6.34 (5.67-7.02) |
| Central Latin America | 4635610 (3895360-5552273) | 137816.8 (116248.29-164357.24) | 6845890 (6005200-7781870) | 60985.15 (53540.2-69128.36) | -3.04 (-3.4--2.68) |
| Central Sub-Saharan Africa | 766367 (654239-886980) | 101203.19 (86436.36-117032.56) | 2831060 (2464331-3206704) | 162994.95 (141969.77-184581.51) | 1.57 (1.41-1.72) |
| Commonwealth High Income | 2351083 (1861871-2889403) | 31063.52 (24490.28-38191.01) | 3918551 (3294658-4635043) | 26256.13 (22028.49-31100.1) | -0.31 (-1.83-1.24) |
| Commonwealth Low Income | 5651193 (4871219-6560024) | 171403.73 (148054.59-198136.01) | 14554914 (12637310-16764666) | 158169.88 (137731.77-181429.47) | -0.38 (-0.46--0.3) |
| Commonwealth Middle Income | 63378760 (50627158-78433289) | 280441.58 (224826.17-345792.63) | 229822490 (194523006-266980596) | 358418.6 (303532.95-415388.68) | 0.69 (0.59-0.8) |
| East Asia | 8983171 (6985068-11159473) | 26328.88 (20528.2-32596.48) | 13677850 (11229656-16375082) | 13148.71 (10745.14-15742.64) | -2.12 (-2.31--1.92) |
| East Asia & Pacific - WB | 26450587 (21313627-32563935) | 49272.94 (39796.69-60401.17) | 61178377 (51684569-71738352) | 38721.99 (32742.39-45306.65) | -0.67 (-0.78--0.56) |
| Eastern Africa | 4147202 (3464629-5001639) | 162877.52 (136369.6-195538.87) | 11447326 (9759216-13353422) | 203107.13 (173325.61-236172.02) | 0.5 (0.4-0.6) |
| Eastern Europe | 585398 (431328-744152) | 5425.28 (3928.58-6919.28) | 1272397 (981213-1589852) | 7903.68 (5971.48-9955.8) | 1.38 (1.06-1.71) |
| Eastern Mediterranean Region | 6611665 (5262707-8337214) | 94802.31 (75753.89-118948.77) | 12763309 (10648171-15177408) | 77703.36 (65026.34-92172.54) | -0.67 (-0.87--0.47) |
| Eastern Sub-Saharan Africa | 4728295 (3967260-5670279) | 175744.03 (147707.82-209910.34) | 12667580 (10856622-14667721) | 214638.35 (184120.65-247858.51) | 0.41 (0.31-0.51) |
| Europe | 13010016 (10932564-15327448) | 25809.26 (21470.71-30637.11) | 37779970 (33539859-43040717) | 42988.29 (37907.79-49074.99) | 2.37 (1.44-3.32) |
| Europe & Central Asia - WB | 13057547 (10972586-15379777) | 25335.68 (21077.56-30064.63) | 37847035 (33594638-43114270) | 42055.59 (37083.99-48002.55) | 2.37 (1.44-3.31) |
| European Region | 13186221 (11081830-15520508) | 25413.7 (21147.43-30138.16) | 38684512 (34389340-44026656) | 42564.86 (37581.43-48544.71) | 2.4 (1.47-3.35) |
| High-income Asia Pacific | 3722606 (2876979-4709046) | 39393.46 (30445.93-49752.37) | 13199919 (10679014-15907441) | 49007.39 (39664.78-59078.36) | 1.26 (0.94-1.57) |
| High-income North America | 2192952 (1628467-2818874) | 12727.5 (9302.68-16407.15) | 1685586 (1416967-2038710) | 4721.09 (3960.03-5712.45) | -4.1 (-5.42--2.76) |
| Latin America & Caribbean - WB | 10250421 (8734816-12068811) | 92013.28 (78628.38-107977.65) | 17296470 (14830541-19892289) | 53191.29 (45684.58-61001.17) | -2.07 (-2.31--1.84) |
| Limited Health System | 70979670 (57350128-86894122) | 247759.33 (200852.26-302058.3) | 249952844 (212367013-288836025) | 313060.33 (266224.69-360926) | 0.65 (0.55-0.74) |
| Middle East & North Africa - WB | 1112526 (925777-1329286) | 24081.09 (20041.01-28702.39) | 4881287 (4252865-5567696) | 37657.61 (32761.8-42919.86) | 1.59 (1.35-1.83) |
| Minimal Health System | 2645649 (2292600-3047422) | 121422.23 (105316.41-139549.85) | 7110048 (6237518-7931943) | 170534.09 (149731.18-190027.52) | 1.01 (0.92-1.09) |
| North Africa and Middle East | 1250665 (1037777-1492215) | 19956.02 (16576.58-23745.18) | 6151164 (5287661-7088954) | 35382.57 (30384.49-40712.77) | 1.95 (1.84-2.06) |
| North America | 2193682 (1629099-2819620) | 12730.88 (9305.73-16410.44) | 1688481 (1419557-2041961) | 4729.63 (3967.69-5721.92) | -4.09 (-5.41--2.75) |
| Northern Africa | 489124 (406393-582049) | 21017.76 (17490.68-24922.57) | 1873429 (1620345-2148016) | 31888.57 (27541.18-36535.98) | 1.14 (1.04-1.25) |
| Oceania | 131030 (111476-153413) | 132832.84 (113110.47-155364.21) | 350845 (300432-399586) | 142240.6 (122047.53-161721.04) | 0.01 (-0.05-0.06) |
| Region of the Americas | 12414710 (10420927-14765109) | 43585.11 (36616.2-51637.29) | 18715116 (16004675-21581753) | 28238.89 (24189.79-32469.98) | -1.67 (-2.04--1.31) |
| South-East Asia Region | 63978329 (51486651-78495068) | 257035.85 (207396.7-314375.52) | 244447686 (207297742-282834841) | 322023.67 (273374.57-371782.27) | 0.66 (0.56-0.76) |
| South Asia | 58242838 (46631893-71470933) | 282469.41 (226904.68-345569.76) | 224243572 (190380361-259953144) | 363457.58 (308719.99-420492.65) | 0.71 (0.61-0.8) |
| South Asia - WB | 58662726 (46996483-71939857) | 275234.59 (221230.65-336500.26) | 225539940 (191477887-261461644) | 356353.98 (302678.23-412268.16) | 0.73 (0.64-0.82) |
| Southeast Asia | 13529766 (11016561-16614796) | 143132.99 (116811.99-175082.64) | 34720220 (29489515-41057937) | 132350.1 (112610.79-156069.46) | -0.29 (-0.46--0.13) |
| Southern Africa | 2759895 (2258820-3350717) | 168880.65 (138424.9-203932.72) | 6624496 (5655855-7681287) | 189326.22 (161820.72-218849.42) | 0.4 (0.27-0.54) |
| Southern Latin America | 1014996 (883877-1161884) | 47586.02 (41227.49-54589.9) | 883858 (761156-1019341) | 20231.45 (17366.55-23354.21) | -2.59 (-3.76--1.41) |
| Southern Sub-Saharan Africa | 2019853 (1609025-2513146) | 190505.89 (151753.41-236081.94) | 4418817 (3638533-5285452) | 195745.6 (161467.14-233147.03) | 0.23 (0.06-0.39) |
| Sub-Saharan Africa - WB | 15524731 (12889219-18836421) | 187905.99 (156441.96-226821.8) | 32589668 (27987177-37290732) | 186923.65 (160680.34-213334.61) | -0.2 (-0.31--0.08) |
| Tropical Latin America | 2873794 (2291173-3541326) | 78263.53 (62677.8-96017.46) | 6834868 (5619840-8238896) | 57219.68 (47121.91-68789.08) | -1 (-1.37--0.63) |
| Western Africa | 7380192 (6046964-9095344) | 243391.64 (200259-298077.73) | 10836838 (9285764-12386110) | 178589.3 (153351.21-203402.01) | -1.32 (-1.44--1.19) |
| Western Europe | 12273864 (10366153-14388266) | 41327.3 (34515.07-48861.5) | 34865149 (31043308-39597901) | 66937.51 (59123.3-76285.04) | 2.19 (1.18-3.2) |
| Western Pacific Region | 14951646 (11885819-18469164) | 31817.39 (25328.41-39162.96) | 30261327 (25042348-35699557) | 21413.63 (17711.62-25210.16) | -1.07 (-1.25--0.9) |
| Western Sub-Saharan Africa | 7896076 (6504688-9676458) | 235739.35 (194959.04-287200.07) | 12184395 (10468633-13861324) | 179549.1 (154541.35-203602.7) | -1.19 (-1.31--1.06) |
| World Bank High Income | 20212645 (16898758-24003962) | 30601.78 (25419.32-36475.25) | 56396178 (49370428-64862904) | 42021.16 (36613.75-48325.91) | 1.7 (0.89-2.52) |
| World Bank Low Income | 5335961 (4546453-6213880) | 107846.02 (92026.93-125268.82) | 15506743 (13399137-17632052) | 149203.09 (128969.39-169152.72) | 0.99 (0.87-1.1) |
| World Bank Lower Middle Income | 80480533 (64957166-98815629) | 202534.95 (164025.5-247639.12) | 267686815 (226897009-310238599) | 253991.6 (215497.54-293652.15) | 0.69 (0.59-0.79) |
| World Bank Upper Middle Income | 21225561 (17283718-25765750) | 36755.93 (29973.71-44419.25) | 41436286 (35306988-48283761) | 25914.89 (22095.32-30116.67) | -1.13 (-1.17--1.08) |
| Country |  |  |  |  |  |
| Afghanistan | 14283 (11726-16994) | 5256.29 (4319.2-6238.25) | 23246 (19714-27078) | 7525.29 (6377.36-8743.78) | 1.34 (1.14-1.53) |
| Albania | 1713 (1358-2099) | 2084.84 (1645.58-2558.59) | 5519 (4370-6802) | 2461.73 (1949.49-3031.41) | 1.04 (0.74-1.34) |
| Algeria | 70291 (57480-84444) | 13747.31 (11254.19-16464.98) | 439081 (371075-512030) | 30473.17 (25657.8-35564.05) | 2.62 (2.56-2.67) |
| American Samoa | 575 (482-674) | 72810.42 (61110.06-85158.47) | 1835 (1583-2116) | 98088.52 (84639.52-112966.74) | 0.99 (0.86-1.12) |
| Andorra | 891 (700-1119) | 33802.02 (26411.71-42593.23) | 2279 (1826-2743) | 32239.19 (25409.29-39353.11) | 0.19 (-0.55-0.94) |
| Angola | 152270 (128015-180479) | 126354.29 (106405.18-149596.6) | 768081 (659257-884967) | 208074.69 (178842.6-239548.62) | 1.69 (1.49-1.88) |
| Antigua and Barbuda | 1138 (958-1359) | 43506.09 (36677.93-51686.27) | 1955 (1645-2276) | 43021.02 (36178.76-50021.6) | -0.33 (-0.45--0.21) |
| Argentina | 529391 (441329-622229) | 35586.07 (29409.18-41980.11) | 425096 (353571-501399) | 15214.84 (12626.93-17979.14) | -2.29 (-3.67--0.89) |
| Armenia | 6302 (5135-7630) | 7291.85 (5867.32-8883.34) | 9704 (7526-12005) | 4955 (3797.14-6152.34) | -1.38 (-1.82--0.94) |
| Australia | 443864 (356303-546789) | 45135.07 (36116.05-55507.57) | 345144 (289719-400505) | 14107.74 (11711.08-16477.94) | -3.96 (-5.61--2.28) |
| Austria | 424628 (335650-524698) | 69923.25 (55608.78-86023.86) | 425556 (358100-517174) | 43351.26 (36148.42-52564.74) | -1.47 (-2.31--0.61) |
| Azerbaijan | 5612 (4273-6947) | 3571.2 (2673.83-4446.06) | 17673 (13164-22255) | 5184.31 (3840.02-6542.41) | 1.36 (1.2-1.52) |
| Bahamas | 1404 (1206-1600) | 23222.62 (19942.87-26506.41) | 3239 (2787-3682) | 20292.92 (17424.24-23118.55) | -0.46 (-0.63--0.29) |
| Bahrain | 1738 (1405-2142) | 32691.32 (26449.35-40184.54) | 23993 (20208-28746) | 89366.28 (75262.86-107148.49) | 3.16 (2.85-3.48) |
| Bangladesh | 2888785 (2454338-3408559) | 159033.16 (135681.64-186737.33) | 7678995 (6530481-8871549) | 126393.99 (107751.41-145808.42) | -0.68 (-0.81--0.55) |
| Barbados | 4923 (4138-5812) | 32843.13 (27599.92-38647.47) | 8717 (7401-10085) | 35050.07 (29588.54-40579) | -0.22 (-0.33--0.11) |
| Belarus | 20153 (14028-27132) | 3944.05 (2699.92-5360.46) | 92043 (70324-117508) | 12173.17 (9169.4-15636.86) | 4.42 (3.83-5.02) |
| Belgium | 414405 (340430-487888) | 51884.85 (42461.58-61291.05) | 1640748 (1449885-1853153) | 123874.31 (108774.17-140552.84) | 3.41 (2.31-4.52) |
| Belize | 1581 (1310-1858) | 39575.52 (32822.42-46253.85) | 4210 (3571-4759) | 37445.45 (31811.25-42278.03) | -0.48 (-0.63--0.33) |
| Benin | 118354 (99865-139590) | 148282.04 (125688.75-174467.39) | 343278 (293934-390639) | 186768.25 (160066.75-212323.64) | 0.61 (0.51-0.71) |
| Bermuda | 840 (706-995) | 31029.41 (25993.34-36707.03) | 2992 (2447-3568) | 46087.36 (36871.33-55747.25) | 0.73 (0.5-0.96) |
| Bhutan | 15333 (13033-18405) | 187116.63 (159192.91-224131.85) | 47885 (40556-55965) | 182287.48 (155118.2-212439.74) | -0.23 (-0.36--0.1) |
| Bolivia (Plurinational State of) | 85571 (69768-103573) | 67480.56 (55258.34-81234.67) | 105873 (89832-120986) | 27222.62 (23167.73-30997.5) | -3.84 (-4.33--3.36) |
| Bosnia and Herzegovina | 3729 (2906-4636) | 2501.22 (1943.55-3107.59) | 14652 (11745-17925) | 4458.84 (3566.41-5450.15) | 2.67 (2.44-2.9) |
| Botswana | 45766 (37432-55759) | 218131.56 (178638.58-265559.81) | 85021 (71602-97980) | 162910.47 (137146.24-187641.58) | -1.1 (-1.37--0.83) |
| Brazil | 2611637 (2045458-3242726) | 73127.54 (57584.27-90349.59) | 6742260 (5534561-8136986) | 57672 (47412.98-69420.72) | -0.69 (-1.06--0.31) |
| Brunei Darussalam | 211 (169-252) | 5610.79 (4464.48-6725.51) | 577 (478-670) | 4609.73 (3815.51-5344.86) | -0.35 (-0.69--0.01) |
| Bulgaria | 8488 (6482-10771) | 1452.24 (1110.29-1839.91) | 13484 (10826-16274) | 1656.46 (1327.97-2003.06) | 0.8 (0.6-0.99) |
| Burkina Faso | 299711 (253731-354193) | 179546.54 (152544.74-211567.97) | 648436 (549577-746916) | 189294.46 (160496.02-218067.35) | 0.05 (-0.07-0.16) |
| Burundi | 113306 (93432-135320) | 127420.46 (105323.97-151846.45) | 306998 (257089-358950) | 189327.74 (158686.12-221042.01) | 1.21 (1.14-1.29) |
| Cabo Verde | 13669 (11323-16214) | 131433.2 (109315.78-154901.04) | 16877 (14547-19131) | 100409.73 (86660.77-113750.51) | -1.24 (-1.39--1.09) |
| Cambodia | 121603 (100107-146011) | 74370.64 (61273.21-89047.29) | 404469 (335809-471672) | 85296.76 (71026.49-99137.67) | 0.22 (0.15-0.29) |
| Cameroon | 239688 (201092-285128) | 155364.6 (130778.93-184745.62) | 676505 (569431-773755) | 158125.4 (132892.36-180989.27) | -0.17 (-0.31--0.02) |
| Canada | 206756 (155895-259765) | 14931.95 (11022.7-18971.76) | 229789 (193448-266478) | 5978.27 (4980.32-6985.33) | -2.93 (-4.24--1.6) |
| Central African Republic | 36568 (30796-43609) | 100914.56 (85020.7-120318.1) | 113178 (95655-130046) | 180951.47 (152965.66-207922.29) | 2 (1.94-2.06) |
| Chad | 204227 (171235-244361) | 172890.56 (145460.48-206263.03) | 444646 (372668-513237) | 219674.69 (184325.96-253384.54) | 0.75 (0.54-0.95) |
| Chile | 354837 (309005-435063) | 80516.9 (70137.1-98174.38) | 366889 (313986-432090) | 28922.75 (24749.41-33979.41) | -3.62 (-4.5--2.72) |
| China | 8208369 (6262218-10336299) | 24998.76 (19136.4-31351.65) | 9390816 (7448856-11522459) | 9444.35 (7471.24-11585.58) | -3.19 (-3.31--3.07) |
| Colombia | 632971 (544373-733535) | 90209.39 (77624.37-104344.5) | 1378825 (1181418-1568168) | 53363.02 (45684.73-60669.56) | -2.49 (-2.88--2.1) |
| Comoros | 9373 (7725-11078) | 131427 (108399.87-155238.14) | 35811 (30465-41293) | 194909.92 (166152.87-224647.64) | 1.28 (1.1-1.46) |
| Congo | 44101 (36465-52551) | 114215.43 (94621.87-135724.4) | 198195 (166854-231550) | 230889.3 (194789.05-269373.66) | 2.45 (2.33-2.57) |
| Cook Islands | 252 (207-299) | 50973.53 (42113.55-60467.8) | 1356 (1132-1627) | 111649.39 (93474.88-133245.76) | 2.66 (2.57-2.74) |
| Costa Rica | 137072 (119580-163184) | 177280.63 (154699.87-210220.06) | 359522 (309465-424617) | 139183.05 (119899.39-164239.49) | -1.13 (-1.42--0.85) |
| Croatia | 5667 (4576-7046) | 2151.6 (1736.01-2676.85) | 34677 (30084-40357) | 6763.92 (5845.57-7887.29) | 4.97 (4.56-5.38) |
| Cuba | 297612 (262679-340005) | 63790.92 (56245.15-72775.44) | 551574 (459298-651988) | 61912.16 (51490.38-72883.17) | -1.13 (-1.45--0.8) |
| Cyprus | 21845 (17323-27664) | 51986.3 (41263.78-65629.36) | 120789 (103509-142220) | 107561.93 (91592.48-127118.13) | 3.49 (2.78-4.21) |
| Czechia | 12442 (9600-15716) | 1809.06 (1395.16-2279.22) | 287919 (254019-324875) | 22901.81 (20204.66-25910.48) | 10.96 (9.85-12.08) |
| C么te d'Ivoire | 164400 (138859-192581) | 133962.26 (113283.65-156668.67) | 577770 (497360-656061) | 159808.76 (137493-181525.2) | 0.6 (0.4-0.8) |
| Democratic People's Republic of Korea | 102460 (82119-124615) | 17876.17 (14346.52-21729.08) | 638579 (515345-761264) | 48939.35 (38908.06-58853.8) | 4.42 (3.81-5.03) |
| Democratic Republic of the Congo | 498846 (419125-582343) | 93716.81 (78785.25-109354.89) | 1648531 (1423736-1896461) | 141516.86 (122227.43-162823.74) | 1.3 (1.14-1.46) |
| Denmark | 265267 (212892-315722) | 59220.38 (47401.22-70657.7) | 858641 (749140-997662) | 125099.38 (108762.03-146113.84) | 3.58 (2.59-4.57) |
| Djibouti | 5584 (4631-6561) | 140576.96 (116683.34-165055.58) | 36384 (30206-42159) | 183539.88 (152366.33-212374.78) | 0.64 (0.35-0.93) |
| Dominica | 815 (674-952) | 28388.52 (23698.13-32998.59) | 760 (659-879) | 20641.75 (17838.35-23861.3) | -1.41 (-1.52--1.3) |
| Dominican Republic | 47924 (39321-56437) | 32774.38 (27024.09-38358.15) | 145221 (122141-167482) | 33637.78 (28399.68-38589.25) | -0.46 (-0.79--0.13) |
| Ecuador | 397446 (342233-477652) | 182074.84 (157636.9-216846.27) | 353736 (302729-403539) | 47196.93 (40439.23-53827.4) | -5.65 (-6.31--4.99) |
| Egypt | 169486 (134939-208855) | 18457.42 (14712.51-22702.85) | 557789 (461621-650783) | 25431.86 (21046.87-29675.46) | 0.51 (0.23-0.79) |
| El Salvador | 177762 (150227-212579) | 137897.33 (116915.38-163971.25) | 225561 (191358-263359) | 75631.57 (64387.04-87798.11) | -2.88 (-3.39--2.37) |
| Equatorial Guinea | 6898 (5701-8213) | 103990.85 (86166.32-123571.56) | 33494 (28652-38879) | 199662.91 (171032.55-231473.02) | 2.14 (1.85-2.43) |
| Eritrea | 45871 (38067-54856) | 158193.26 (131274.67-189213.44) | 223375 (187411-263411) | 265043.55 (222534.8-312392.3) | 1.43 (1.31-1.56) |
| Estonia | 3541 (2488-4671) | 4215.19 (2897.81-5593.86) | 24680 (19282-31565) | 17407.62 (13302.95-22211.43) | 5.66 (4.96-6.36) |
| Eswatini | 20558 (16765-25237) | 203743.89 (166493.85-249582.32) | 41874 (35147-48750) | 209376.32 (176013.65-243668.91) | 0.03 (-0.18-0.23) |
| Ethiopia | 1337009 (1062614-1679809) | 194362.63 (154794.75-243344.49) | 3791364 (3084736-4601181) | 235149.56 (191569.98-283759.54) | 0.4 (0.24-0.57) |
| Fiji | 14262 (11759-17202) | 120563.37 (99769.04-144918.59) | 31644 (26343-36483) | 111006.18 (92692.38-127683.74) | -0.23 (-0.39--0.08) |
| Finland | 191422 (156768-225371) | 51598.29 (41963.59-61014.47) | 193595 (160334-230239) | 26934.29 (21985.01-32382.51) | -1.68 (-2.68--0.67) |
| France | 2839929 (2250498-3382628) | 65174.44 (51647.7-77702.97) | 5811150 (4874333-6980178) | 75165.84 (62482.48-90682.96) | 0.83 (-0.15-1.82) |
| Gabon | 27685 (22959-32693) | 120638.09 (100561.11-142299.97) | 69581 (58451-82118) | 186693.81 (156813.97-220051) | 1.36 (1.26-1.46) |
| Gambia | 17369 (14438-20331) | 136089.72 (113468.04-159032.77) | 48505 (41507-55350) | 136098.64 (116875.22-155317.17) | -0.21 (-0.31--0.11) |
| Georgia | 15288 (11562-19226) | 6430.44 (4783.69-8170.16) | 17976 (14524-21547) | 6555.1 (5243.52-7884.42) | 1.06 (0.48-1.64) |
| Germany | 2312354 (1868503-2749176) | 37540.23 (29692.02-45213.22) | 8726538 (7730086-9881747) | 80387.1 (70779.2-91197.67) | 3.29 (2.03-4.56) |
| Ghana | 268688 (225469-318785) | 123125.47 (103395.18-145865.88) | 846685 (723059-965508) | 141909.78 (121090.29-161788.79) | -0.1 (-0.27-0.08) |
| Greece | 285415 (222956-358516) | 39237.41 (30614.43-49505.18) | 301189 (238152-369389) | 25349.41 (19585.21-31452.96) | -0.66 (-1.36-0.04) |
| Greenland | 60 (49-71) | 5717.77 (4711.6-6749.32) | 70 (59-84) | 2648.99 (2194.85-3162.2) | -2.23 (-2.89--1.57) |
| Grenada | 875 (729-1022) | 25593.63 (21453.01-29669.48) | 1174 (989-1356) | 25211.64 (21241.09-29183.01) | -0.74 (-0.96--0.52) |
| Guam | 1631 (1340-1954) | 61889.32 (50845.57-74071.49) | 8930 (7562-10345) | 90906.68 (77134.78-105174.99) | 1.44 (1.2-1.68) |
| Guatemala | 279436 (248617-324443) | 217764.57 (194518.64-251797.96) | 553812 (488114-612908) | 113968.56 (100674.17-125788.83) | -2.64 (-2.92--2.35) |
| Guinea | 204277 (172493-243020) | 148025.24 (125182.23-175748.65) | 376393 (317878-432608) | 179308.24 (151657.14-205722.73) | 0.31 (0.18-0.45) |
| Guinea-Bissau | 19348 (16235-22657) | 135166.9 (113569.83-158232.22) | 41690 (35041-48066) | 180811.54 (152059.44-208363.55) | 0.79 (0.66-0.92) |
| Guyana | 6240 (5172-7407) | 42795.52 (35418.06-50604.76) | 10422 (8780-11686) | 41880.87 (35480.22-46882.5) | -0.97 (-1.25--0.69) |
| Haiti | 30384 (25207-35634) | 25637.88 (21330.62-30013.39) | 78200 (66945-89485) | 30795.11 (26427.85-35187.77) | 0.81 (0.32-1.3) |
| Honduras | 111978 (93531-135485) | 136346.91 (114448.4-164302.33) | 295226 (243779-353072) | 111653.51 (92697.7-132967.55) | -1.17 (-1.48--0.86) |
| Hungary | 14509 (11051-18164) | 2039.55 (1553.34-2557.84) | 219982 (191105-255000) | 19854.02 (17273.78-22984.48) | 9.53 (8.73-10.33) |
| Iceland | 7507 (6064-9038) | 51323.9 (41412.57-62071.21) | 21374 (17917-25660) | 69284.89 (57822.55-83700.71) | 1.64 (0.64-2.64) |
| India | 49389179 (39278665-61019350) | 305910.49 (243911.86-376957.03) | 206921994 (175207727-239954622) | 411545.31 (348590-476347.75) | 0.84 (0.74-0.94) |
| Indonesia | 9041514 (7149607-11424771) | 262631.52 (208046.57-330849.98) | 17706494 (14331750-21889393) | 198289.12 (160813.67-244730.26) | -0.97 (-1.17--0.76) |
| Iran (Islamic Republic of) | 265235 (208769-328846) | 28086.99 (22113.99-34807.26) | 841608 (670803-1013367) | 27326.94 (21800.21-32863) | -0.23 (-0.39--0.06) |
| Iraq | 62470 (50931-74470) | 20080.77 (16376.63-23907.97) | 232757 (195661-267460) | 27612.09 (23120.38-31787.34) | 1.46 (1.25-1.66) |
| Ireland | 59901 (47408-72322) | 29832.36 (23200.04-36489.96) | 252225 (210534-309014) | 60893.76 (50454.24-74786.3) | 3.88 (2.8-4.96) |
| Israel | 120964 (100397-140978) | 48545.27 (40067.29-56763.11) | 814762 (724327-942764) | 120458.08 (106521.86-139806.9) | 3.81 (2.65-4.99) |
| Italy | 971000 (714275-1277689) | 22033.28 (16191.73-28905.39) | 2487092 (2052678-3014851) | 29537.1 (24211.9-35909.16) | 1.94 (1.7-2.18) |
| Jamaica | 43464 (37053-51777) | 51371.72 (43835.24-60736.74) | 36807 (31399-41650) | 27317.04 (23311.01-30972.54) | -1.95 (-2.51--1.38) |
| Japan | 3589178 (2759150-4555227) | 43816.45 (33684.52-55544.24) | 12608168 (10142765-15245679) | 59319.91 (47708.46-71741.66) | 1.49 (1.18-1.8) |
| Jordan | 7165 (5660-8906) | 16066.94 (12693.68-19949.86) | 89924 (74369-106923) | 34102.67 (28135.75-40546.11) | 3 (2.66-3.34) |
| Kazakhstan | 19904 (15825-24326) | 4568.1 (3598.5-5636.47) | 34755 (26065-44316) | 5103.65 (3794.04-6554.38) | 0.59 (0.15-1.03) |
| Kenya | 914795 (721200-1155941) | 297707.04 (234996.9-373758.72) | 2571114 (2099184-3113931) | 310860.18 (253899.75-375209.47) | -0.06 (-0.2-0.07) |
| Kiribati | 2925 (2569-3381) | 219917.22 (193321.53-253849.4) | 5330 (4589-6304) | 228027.05 (196563.99-269263.24) | -0.15 (-0.22--0.07) |
| Kuwait | 3026 (2443-3630) | 17524.43 (14070.15-21069.12) | 22426 (17866-26924) | 26518.85 (20875.26-32007.44) | 2.72 (2.32-3.11) |
| Kyrgyzstan | 3980 (3130-4860) | 3773.48 (2926.57-4647.86) | 7178 (5599-8706) | 4301.48 (3327.77-5241.07) | 0.85 (0.66-1.04) |
| Lao People's Democratic Republic | 45397 (37064-54577) | 58021.23 (47514.11-69504.11) | 129735 (109740-149397) | 78364.53 (66473.1-90044.99) | 1.09 (0.88-1.29) |
| Latvia | 5930 (4220-7765) | 4048.75 (2833.49-5347.62) | 19016 (14480-24102) | 9450.6 (7113.46-12065.29) | 3.68 (3.17-4.18) |
| Lebanon | 19314 (15766-23423) | 22855.18 (18678.01-27654.58) | 158214 (130201-191470) | 55201.24 (45200.29-66714.02) | 3.19 (3.1-3.28) |
| Lesotho | 77239 (63502-94845) | 224278.23 (184618.03-274634.47) | 93662 (78148-111081) | 223495.29 (186827.97-264450.57) | -0.21 (-0.47-0.04) |
| Liberia | 62111 (52447-72782) | 128992.76 (109246.87-150817.23) | 164391 (138919-189770) | 239053.39 (202224.51-275832.12) | 1.93 (1.74-2.12) |
| Libya | 19408 (15464-23951) | 27115.16 (21638.96-33254.67) | 66149 (54396-77741) | 36956.71 (30408.62-43445.03) | 1.17 (1.03-1.31) |
| Lithuania | 9674 (7136-12252) | 5085.9 (3682.46-6489.14) | 67410 (56482-81829) | 22613.4 (18616.25-27575) | 5.88 (4.89-6.87) |
| Luxembourg | 14835 (12182-17726) | 53878.57 (44008.95-64461.61) | 44689 (38336-54133) | 81087.78 (69257.53-98448.97) | 1.88 (0.85-2.93) |
| Madagascar | 207255 (178381-236283) | 112771.34 (96998.1-128533.99) | 618426 (525228-715587) | 181018.1 (153637.8-209483.32) | 1.46 (1.39-1.53) |
| Malawi | 219777 (183275-259166) | 155111.8 (129560.71-182750.81) | 537635 (459384-618442) | 200250.72 (171408-230001.92) | 0.61 (0.49-0.73) |
| Malaysia | 176085 (147351-205701) | 51223.78 (43038.36-59572.94) | 1734540 (1462691-2096164) | 145623.98 (122777.24-175160.22) | 3.49 (3.37-3.6) |
| Maldives | 1748 (1439-2109) | 61608.73 (50769.15-74214.62) | 12197 (10365-14570) | 107487 (91522.73-127686.08) | 1.97 (1.8-2.14) |
| Mali | 303315 (258760-352742) | 211230.81 (180883.29-244996.95) | 855820 (719333-988814) | 272764.71 (230185.74-314479.96) | 0.65 (0.54-0.75) |
| Malta | 5290 (4133-6469) | 27446.74 (21128.39-33868.77) | 18022 (14406-21951) | 34233.03 (26914.39-42056.3) | 1.2 (0.43-1.98) |
| Marshall Islands | 628 (508-753) | 99658.95 (80811.33-118933.11) | 693 (602-788) | 60662.11 (52745.36-68993.75) | -1.9 (-2.03--1.77) |
| Mauritania | 69208 (57810-81742) | 167169.49 (140261.23-196859.68) | 223638 (191237-259799) | 266381.64 (228423.42-308355.18) | 1.12 (1-1.24) |
| Mauritius | 14787 (12357-17004) | 51288.53 (42919.8-58991.79) | 30780 (26486-34724) | 37092.31 (31981.02-41841.28) | -0.98 (-1.29--0.68) |
| Mexico | 2750210 (2109890-3517145) | 158813.86 (122493.06-202049.3) | 2376669 (1944988-2882227) | 42763.99 (35138.1-51624.03) | -4.37 (-4.87--3.87) |
| Micronesia (Federated States of) | 1560 (1290-1875) | 77602.68 (64488.62-92825.91) | 1450 (1243-1635) | 60072.8 (51590.22-67679.4) | -0.92 (-1.06--0.79) |
| Monaco | 1203 (932-1506) | 32420.08 (25055.63-40632.9) | 1768 (1431-2140) | 34284.3 (27282.31-42178.42) | 0.68 (-0.1-1.48) |
| Mongolia | 842 (676-1007) | 2125.22 (1691.69-2553.82) | 1135 (882-1381) | 1608.37 (1239.83-1963.88) | -0.69 (-1.11--0.27) |
| Montenegro | 638 (475-817) | 2409.15 (1794.28-3078.39) | 1411 (1085-1778) | 2830.51 (2178.25-3559.7) | 0.94 (0.78-1.11) |
| Morocco | 116296 (94615-139250) | 19963.37 (16300.12-23789.78) | 318521 (270075-369114) | 22767.49 (19304.13-26378.04) | 0.19 (0.06-0.31) |
| Mozambique | 236276 (196843-276104) | 111116.12 (92368.87-129683.13) | 521928 (449912-592430) | 136792.56 (117967.52-155158.65) | 0.39 (0.3-0.48) |
| Myanmar | 534106 (432621-642966) | 61630.57 (49973.79-74042.43) | 1348988 (1143500-1530155) | 69589.9 (59187.47-78786.51) | 0.12 (0.04-0.2) |
| Namibia | 49184 (40335-59348) | 196974.3 (161651.42-237380.8) | 119885 (100137-142094) | 237657.26 (198886.45-281202.72) | 0.62 (0.42-0.82) |
| Nauru | 79 (65-92) | 51572.39 (42735.68-60421.84) | 105 (90-118) | 51858.89 (44855.11-58649.17) | -0.34 (-0.57--0.11) |
| Nepal | 455066 (372898-534147) | 137013.94 (112568.5-160464.69) | 1268765 (1102272-1445012) | 129931.58 (113254.94-147519.8) | 0.2 (-0.04-0.45) |
| Netherlands | 446647 (357163-538784) | 44863.14 (35564.26-54862.51) | 1921325 (1628001-2300259) | 98448 (82565.43-118511.69) | 3.14 (1.96-4.32) |
| New Zealand | 89595 (68279-115967) | 46765.18 (35453.94-60435.25) | 256578 (205827-314577) | 56076.5 (44948.14-68896.44) | 1.81 (1.26-2.37) |
| Nicaragua | 71973 (59412-87182) | 117125.47 (96940.5-141180.01) | 96807 (83644-110500) | 46949.94 (40715.09-53489.81) | -3.76 (-4.16--3.36) |
| Niger | 145945 (122629-172692) | 154928.83 (130426.06-183065.25) | 569189 (483868-647906) | 200268.25 (170528.6-228000.73) | 0.9 (0.73-1.08) |
| Nigeria | 5316474 (4164308-6794640) | 308330.25 (242195.24-390802.54) | 5193658 (4269807-6192814) | 168583.19 (138864.68-199950.37) | -2.37 (-2.5--2.23) |
| Niue | 88 (72-105) | 81287.84 (67079.83-96779.81) | 84 (70-98) | 88339.21 (73956.03-102413.29) | 0.4 (0.28-0.52) |
| North Macedonia | 2481 (1969-3139) | 3262.56 (2584.06-4122.99) | 4764 (3679-5915) | 2925.94 (2268.08-3620.99) | -0.11 (-0.28-0.05) |
| Northern Mariana Islands | 371 (309-445) | 85236.85 (71137.44-101754.49) | 2345 (1994-2753) | 129042.89 (109938.57-150965.07) | 1.02 (0.86-1.18) |
| Norway | 255678 (198302-324636) | 64581.15 (50137.28-81994.59) | 1109731 (925057-1337232) | 195221.24 (161387.94-236861.66) | 3.58 (3.13-4.03) |
| Oman | 10969 (9118-13610) | 46686.07 (38975.37-57512.15) | 67245 (56656-80427) | 112936.22 (95397.78-134665.08) | 2.92 (2.57-3.26) |
| Pakistan | 5494474 (4302376-7003921) | 235063.41 (184933.07-297775.09) | 8325933 (6752796-10139226) | 189073.96 (153886.67-229774.95) | -0.83 (-1.03--0.64) |
| Palau | 275 (227-325) | 69076.03 (57337.81-81465.72) | 722 (607-846) | 86688.43 (73128.45-101466.91) | 0.55 (0.45-0.64) |
| Palestine | 6292 (5130-7529) | 17839.21 (14555.7-21318.73) | 22822 (19141-26424) | 25389.36 (21274.19-29423.65) | 1.49 (1.29-1.7) |
| Panama | 74194 (62378-88859) | 114049.04 (96017.86-136089.98) | 198047 (168382-230567) | 95573.1 (81520.14-110878.97) | -0.88 (-1.22--0.53) |
| Papua New Guinea | 88136 (74266-104307) | 149218.32 (125776.2-176544.31) | 251902 (212431-290330) | 158963.49 (134182.55-182930.51) | -0.06 (-0.13-0.01) |
| Paraguay | 262157 (229580-303758) | 275111.81 (241048.37-317867.72) | 92608 (78549-106174) | 36696.3 (31187.11-41996.69) | -7.83 (-8.58--7.08) |
| Peru | 759061 (645021-924561) | 154515.14 (132080.03-186919.92) | 1119513 (923950-1306861) | 74200.23 (61443.48-86228.47) | -3.13 (-3.52--2.73) |
| Philippines | 870704 (683178-1091495) | 80819.23 (63573.59-100764.79) | 1347841 (1095870-1614329) | 43265.15 (35306.33-51634.76) | -2.44 (-2.68--2.19) |
| Poland | 26785 (19187-36614) | 1282.18 (914.35-1754.36) | 72848 (59400-89460) | 1831.96 (1491.05-2253.52) | 2.41 (0.91-3.93) |
| Portugal | 132246 (107140-158260) | 19366.84 (15529.09-23307.43) | 592754 (499330-705249) | 42238.56 (35354.09-50479.46) | 4.23 (3.39-5.08) |
| Puerto Rico | 27937 (22394-33409) | 17002.17 (13321.34-20606.34) | 264804 (223891-316475) | 68704.46 (57799.72-81992.75) | 4.92 (4.26-5.58) |
| Qatar | 393 (326-472) | 16634.17 (13794.5-20003.89) | 13621 (11818-15740) | 74644.24 (64773.71-86234.29) | 5.31 (5.11-5.51) |
| Republic of Korea | 127411 (105563-152612) | 11380.76 (9393.42-13620.84) | 578287 (486595-700881) | 12441.21 (10403.13-15062.2) | 0.94 (0.56-1.32) |
| Republic of Moldova | 6963 (4818-9248) | 3989.96 (2740.39-5315.5) | 20550 (15822-25653) | 7353.01 (5585.8-9241.62) | 2.61 (2.25-2.97) |
| Romania | 13042 (10070-16291) | 1057.46 (817.25-1322.75) | 57055 (47402-67093) | 2723.98 (2261.19-3205.58) | 3.77 (3.51-4.04) |
| Russian Federation | 453032 (337543-569089) | 6676.99 (4891.65-8406.61) | 816204 (620394-1024604) | 7520.04 (5624.15-9501.33) | 0.47 (0.19-0.74) |
| Rwanda | 98239 (81400-115253) | 96388.29 (79856.94-113014.7) | 383964 (317192-442834) | 170516.62 (140930.02-196422.21) | 1.91 (1.74-2.09) |
| Saint Kitts and Nevis | 1042 (870-1251) | 52199.47 (43714-62303.68) | 1767 (1488-2058) | 65616.79 (55243.94-76402.14) | 0.37 (0.25-0.49) |
| Saint Lucia | 1622 (1351-1912) | 39914.44 (33340.11-46776.95) | 3978 (3350-4529) | 37973.25 (32011.42-43224.21) | -0.78 (-0.97--0.59) |
| Saint Vincent and the Grenadines | 1529 (1296-1824) | 45418.37 (38616.91-53777.54) | 2341 (1979-2666) | 36555.6 (30967.49-41593.57) | -1.06 (-1.22--0.89) |
| Samoa | 1790 (1478-2106) | 53573.04 (44322.52-62896.99) | 3866 (3294-4489) | 68374.94 (58482.44-78913.93) | 0.63 (0.55-0.7) |
| San Marino | 667 (520-824) | 37185.76 (28770.44-46155.71) | 1443 (1179-1731) | 37450.4 (29987.35-45627.32) | 0.55 (-0.15-1.25) |
| Sao Tome and Principe | 2501 (2145-2877) | 94934.22 (81578.98-109303.13) | 2625 (2267-2999) | 68828.26 (59485.35-78476.33) | -1.38 (-1.71--1.05) |
| Saudi Arabia | 94362 (79301-115115) | 45823.73 (38530.79-55672.12) | 426144 (360835-496003) | 83213.21 (70465.33-96846.57) | 2.5 (2.15-2.84) |
| Senegal | 226070 (191661-266905) | 181285.47 (154315.84-213444.92) | 588452 (500097-676796) | 203612.91 (173414.66-234267.95) | 0 (-0.13-0.13) |
| Serbia | 10143 (7963-12696) | 2175.15 (1706.14-2725.52) | 90728 (78289-106197) | 9985.02 (8602.43-11663.76) | 6.5 (5.93-7.07) |
| Seychelles | 1571 (1284-1884) | 60279.02 (49459.87-71804.5) | 3719 (3106-4294) | 80678.16 (67537.13-93003.13) | 1.05 (0.9-1.2) |
| Sierra Leone | 146467 (122732-172409) | 173986.96 (146579.33-203657.99) | 301550 (256269-345732) | 220686.13 (187633.52-252364.22) | 0.45 (0.34-0.56) |
| Singapore | 5806 (5150-6509) | 6501.75 (5767.21-7284.27) | 12887 (11323-14305) | 3093.52 (2711.5-3438.92) | -2.01 (-2.69--1.33) |
| Slovakia | 4094 (3238-4993) | 1414.85 (1118.37-1725.29) | 17841 (14423-22051) | 3486.23 (2816.21-4305.16) | 3.67 (3.47-3.86) |
| Slovenia | 2812 (2128-3505) | 2417.7 (1833.38-3021.34) | 12390 (9909-15100) | 4948.8 (3939.26-6060.53) | 2.82 (2.63-3.02) |
| Solomon Islands | 5500 (4546-6556) | 121745.32 (100710.83-145014.13) | 13959 (11722-16028) | 122767.05 (103642.17-140827.51) | -0.11 (-0.17--0.06) |
| Somalia | 79918 (65952-93544) | 124273.34 (102576.85-145283.16) | 303837 (255632-348206) | 157156.04 (132222.75-180119.6) | 0.51 (0.38-0.63) |
| South Africa | 1575947 (1223960-2015834) | 193594.77 (150277.87-246303.04) | 3723497 (3016457-4519051) | 201737.3 (163659.32-243852.8) | 0.39 (0.2-0.59) |
| South Sudan | 178938 (144752-215871) | 169716.61 (137722.43-204545.53) | 387953 (328169-459090) | 321981.87 (273068.8-380319.51) | 2.05 (1.96-2.14) |
| Spain | 1154883 (944137-1357895) | 41313.29 (33452.56-49009.65) | 3632157 (3105902-4237328) | 67103.06 (56939.12-78421.36) | 2.31 (1.49-3.13) |
| Sri Lanka | 403857 (333161-485510) | 97314.03 (80609.68-116315) | 1260926 (1047132-1492989) | 99775.81 (83012.74-117703.86) | 0 (-0.12-0.12) |
| Sudan | 107008 (87809-132234) | 27840.63 (22943.07-34128.99) | 500871 (420511-593327) | 70333.23 (59110.28-83012.15) | 2.91 (2.65-3.17) |
| Suriname | 4527 (3889-5333) | 46116.69 (39710.11-54120.26) | 11700 (9909-13392) | 44880.6 (38098.59-51263.62) | -0.24 (-0.33--0.16) |
| Sweden | 437985 (339393-571045) | 50188.96 (38892.21-65070.88) | 2107368 (1756139-2558721) | 164598.22 (135751.29-201055.27) | 5.13 (4.47-5.8) |
| Switzerland | 341535 (279726-401818) | 61822.96 (50609.17-73068.55) | 857758 (739142-1014593) | 85766.75 (73331.56-102230.47) | 2.04 (1.24-2.85) |
| Syrian Arab Republic | 23241 (18729-27916) | 12150.96 (9787.84-14599.45) | 142655 (115913-170650) | 28061.88 (22741.59-33625.5) | 2.82 (2.65-2.99) |
| Taiwan (Province of China) | 672342 (593773-766018) | 101570.16 (89676.25-115673.52) | 3648455 (3065298-4360189) | 170987.04 (142747.74-204205.35) | 3.84 (2.44-5.25) |
| Tajikistan | 9021 (7660-10647) | 9406.57 (8001.31-11067.69) | 20304 (17219-23791) | 11175.62 (9485.34-13084.46) | 0.41 (0.29-0.52) |
| Thailand | 1140154 (937970-1379817) | 88701.54 (73122.62-106871.98) | 7524829 (6393483-8954664) | 153344.48 (130458.94-182003.49) | 1.99 (1.81-2.17) |
| Timor-Leste | 6126 (5003-7533) | 76653.29 (62622.07-94044.78) | 38035 (31453-45240) | 100992.56 (83790.18-119704.37) | 1.01 (0.89-1.12) |
| Togo | 73993 (62710-86733) | 172148 (146167.31-201426.2) | 264142 (224872-304744) | 209496.62 (178430.3-241322.84) | 0.49 (0.35-0.63) |
| Tokelau | 54 (43-65) | 90981.23 (73682.37-109291.59) | 73 (62-85) | 102955.42 (87532.29-119993.48) | 0.26 (0.1-0.43) |
| Tonga | 1360 (1138-1591) | 62218.63 (52251.87-72580.1) | 1606 (1366-1834) | 46745.36 (39867.25-53252.33) | -1.05 (-1.25--0.84) |
| Trinidad and Tobago | 10357 (8597-12135) | 27503.24 (22938.85-32103.33) | 23873 (19936-27558) | 26241.13 (21923.83-30310.08) | -0.19 (-0.34--0.05) |
| Tunisia | 44435 (36082-53533) | 21835.19 (17723.93-26256.4) | 268250 (222151-314827) | 46256.12 (38289.18-54315.85) | 2.79 (2.6-2.98) |
| Turkmenistan | 4576 (3847-5402) | 6928.5 (5817.89-8143.47) | 5700 (4449-6983) | 4100.36 (3176.58-5043.4) | -2.23 (-2.45--2.01) |
| Tuvalu | 190 (157-228) | 70858.48 (58642.87-85007.33) | 316 (272-361) | 71718.84 (61978.92-81782.46) | -0.15 (-0.25--0.05) |
| T眉rkiye | 148002 (121072-176021) | 11773.63 (9621.25-14008.15) | 1609191 (1328615-1893189) | 38398.29 (31698.24-45130.03) | 4.62 (4.23-5.01) |
| Uganda | 368832 (301843-442686) | 152752.1 (125239.73-182333.69) | 1028065 (868355-1200776) | 203621.96 (172219.69-237552.68) | 0.94 (0.77-1.11) |
| Ukraine | 86107 (59827-115592) | 2974.21 (2032.22-4019.78) | 232495 (172594-299858) | 6708.58 (4867.54-8743.13) | 2.98 (2.63-3.34) |
| United Arab Emirates | 6916 (5755-8226) | 63646.55 (53121.34-75568.38) | 77580 (65897-89532) | 100873.29 (85203.24-116854.47) | 1.62 (1.54-1.71) |
| United Kingdom | 1557282 (1200343-1961632) | 33325.16 (25605.26-41871.78) | 2891497 (2377786-3458502) | 40105.62 (32886.37-48070.85) | 0.83 (-0.81-2.49) |
| United Republic of Tanzania | 778022 (664042-922543) | 188451.04 (161190.64-222529.87) | 1531663 (1307671-1759268) | 167863.17 (143440.04-192636.25) | -0.87 (-1.04--0.7) |
| United States of America | 1986086 (1459392-2578438) | 12483.64 (9022.31-16263.98) | 1455699 (1192650-1794601) | 4567.84 (3742.07-5628.11) | -4.82 (-6.47--3.13) |
| United States Virgin Islands | 616 (509-726) | 18846.25 (15562.69-22229.64) | 2038 (1679-2395) | 21839.58 (17810.6-25688.15) | 0.22 (0.07-0.37) |
| Uruguay | 130720 (110542-156097) | 66777.47 (56644.4-79385.86) | 91824 (78176-109224) | 31933.07 (27201.13-37821.4) | -2.13 (-3.14--1.11) |
| Uzbekistan | 17820 (14509-21348) | 4485.82 (3624.18-5417.73) | 21914 (16668-27624) | 2439.78 (1848.37-3081.13) | -1.92 (-2.13--1.72) |
| Vanuatu | 2995 (2414-3657) | 137376.27 (110878.27-167471.98) | 8879 (7433-10281) | 144229.28 (120954.06-166718.16) | -0.02 (-0.08-0.04) |
| Venezuela (Bolivarian Republic of) | 400012 (342235-465595) | 102084.77 (87554.09-118478.99) | 1361421 (1136500-1585883) | 100101.81 (83732.75-116188.02) | -0.9 (-1.18--0.63) |
| Viet Nam | 1152541 (955443-1380712) | 66520.58 (55331.95-79479.63) | 3129241 (2644349-3720948) | 79462.38 (67268.04-94358.11) | 0.61 (0.43-0.79) |
| Yemen | 59651 (49293-73494) | 33792.05 (27952.04-41512.31) | 243341 (204682-282102) | 47433.08 (39937.57-54802.96) | 1.08 (0.85-1.3) |
| Zambia | 131720 (109804-154638) | 135422.63 (112970.75-158889.29) | 378034 (326687-432461) | 168552.27 (145516.04-192937.94) | 0.49 (0.42-0.57) |
| Zimbabwe | 251159 (212859-296128) | 161043.22 (136813.12-189591.51) | 354878 (298704-407860) | 143817.95 (121258.35-165208.29) | -0.88 (-1.11--0.64) |

eTable 5. Risk factors for diarrhea disease in the elderly.

| **measure** | **location** | **sex** | **age** | **cause** | **rei** | **metric** | **year** | **val** | **upper** | **lower** |
| --- | --- | --- | --- | --- | --- | --- | --- | --- | --- | --- |
| Deaths | Middle SDI | Both | 65+ years | diarrhea diseases | Unsafe water source | Rate | 2021 | 43.88966668 | 78.6838586 | 11.88210303 |
| Deaths | Middle SDI | Both | 65+ years | diarrhea diseases | Unsafe water source | Percent | 2021 | 1.131391168 | 1.667451514 | 0.437182712 |
| Deaths | Middle SDI | Both | 65+ years | diarrhea diseases | Unsafe water source | Number | 2021 | 57771.2955 | 103569.9615 | 15600.04399 |
| DALYs (Disability-Adjusted Life Years) | Middle SDI | Both | 65+ years | diarrhea diseases | Unsafe water source | Rate | 2021 | 757.071665 | 1319.321983 | 223.0667686 |
| DALYs (Disability-Adjusted Life Years) | Middle SDI | Both | 65+ years | diarrhea diseases | Unsafe water source | Percent | 2021 | 1.128611634 | 1.667718342 | 0.435241579 |
| DALYs (Disability-Adjusted Life Years) | Middle SDI | Both | 65+ years | diarrhea diseases | Unsafe water source | Number | 2021 | 949166.3978 | 1654612.843 | 278857.3765 |
| Deaths | Low-middle SDI | Both | 65+ years | diarrhea diseases | Unsafe water source | Rate | 2021 | 314.3802915 | 567.9774337 | 125.0674557 |
| Deaths | Low-middle SDI | Both | 65+ years | diarrhea diseases | Unsafe water source | Percent | 2021 | 1.402219616 | 1.815146758 | 0.720609976 |
| Deaths | Low-middle SDI | Both | 65+ years | diarrhea diseases | Unsafe water source | Number | 2021 | 206100.2453 | 372090.5038 | 82588.82327 |
| DALYs (Disability-Adjusted Life Years) | Low-middle SDI | Both | 65+ years | diarrhea diseases | Unsafe water source | Rate | 2021 | 5231.542168 | 9268.086001 | 2090.064854 |
| DALYs (Disability-Adjusted Life Years) | Low-middle SDI | Both | 65+ years | diarrhea diseases | Unsafe water source | Percent | 2021 | 1.401290974 | 1.814288056 | 0.72011135 |
| DALYs (Disability-Adjusted Life Years) | Low-middle SDI | Both | 65+ years | diarrhea diseases | Unsafe water source | Number | 2021 | 3290209.452 | 5825909.223 | 1327815.873 |
| Deaths | Low SDI | Both | 65+ years | diarrhea diseases | Unsafe water source | Rate | 2021 | 504.2387181 | 862.5202322 | 215.2534321 |
| Deaths | Low SDI | Both | 65+ years | diarrhea diseases | Unsafe water source | Percent | 2021 | 1.498252589 | 1.848049262 | 0.839371064 |
| Deaths | Low SDI | Both | 65+ years | diarrhea diseases | Unsafe water source | Number | 2021 | 103048.5579 | 176072.3041 | 44070.23452 |
| DALYs (Disability-Adjusted Life Years) | Low SDI | Both | 65+ years | diarrhea diseases | Unsafe water source | Rate | 2021 | 8792.769087 | 14883.24994 | 3791.111219 |
| DALYs (Disability-Adjusted Life Years) | Low SDI | Both | 65+ years | diarrhea diseases | Unsafe water source | Percent | 2021 | 1.497904008 | 1.847860684 | 0.839022476 |
| DALYs (Disability-Adjusted Life Years) | Low SDI | Both | 65+ years | diarrhea diseases | Unsafe water source | Number | 2021 | 1734018.412 | 2933510.955 | 748666.2036 |
| Deaths | High-middle SDI | Both | 65+ years | diarrhea diseases | Unsafe water source | Rate | 2021 | 3.623810726 | 7.141376811 | 0.839209043 |
| Deaths | High-middle SDI | Both | 65+ years | diarrhea diseases | Unsafe water source | Percent | 2021 | 0.745982746 | 1.235866108 | 0.230607326 |
| Deaths | High-middle SDI | Both | 65+ years | diarrhea diseases | Unsafe water source | Number | 2021 | 3898.490427 | 7713.317606 | 900.4801534 |
| DALYs (Disability-Adjusted Life Years) | High-middle SDI | Both | 65+ years | diarrhea diseases | Unsafe water source | Rate | 2021 | 68.63637178 | 129.0903481 | 17.52997417 |
| DALYs (Disability-Adjusted Life Years) | High-middle SDI | Both | 65+ years | diarrhea diseases | Unsafe water source | Percent | 2021 | 0.751190364 | 1.242466341 | 0.237624203 |
| DALYs (Disability-Adjusted Life Years) | High-middle SDI | Both | 65+ years | diarrhea diseases | Unsafe water source | Number | 2021 | 68806.94807 | 130551.2807 | 17448.30683 |
| Deaths | High SDI | Both | 65+ years | diarrhea diseases | Unsafe water source | Rate | 2021 | 1.351983514 | 3.284090934 | 0.246247856 |
| Deaths | High SDI | Both | 65+ years | diarrhea diseases | Unsafe water source | Percent | 2021 | 0.153718587 | 0.377908942 | 0.029180495 |
| Deaths | High SDI | Both | 65+ years | diarrhea diseases | Unsafe water source | Number | 2021 | 1801.690417 | 4377.373648 | 326.6139473 |
| DALYs (Disability-Adjusted Life Years) | High SDI | Both | 65+ years | diarrhea diseases | Unsafe water source | Rate | 2021 | 24.19044527 | 57.72736919 | 4.540840244 |
| DALYs (Disability-Adjusted Life Years) | High SDI | Both | 65+ years | diarrhea diseases | Unsafe water source | Percent | 2021 | 0.166024551 | 0.39871394 | 0.032115185 |
| DALYs (Disability-Adjusted Life Years) | High SDI | Both | 65+ years | diarrhea diseases | Unsafe water source | Number | 2021 | 29320.54446 | 70036.80219 | 5468.794289 |
| Deaths | Global | Both | 65+ years | diarrhea diseases | Unsafe water source | Rate | 2021 | 83.22639137 | 146.1739884 | 33.62900781 |
| Deaths | Global | Both | 65+ years | diarrhea diseases | Unsafe water source | Percent | 2021 | 1.324966699 | 1.733799405 | 0.672295215 |
| Deaths | Global | Both | 65+ years | diarrhea diseases | Unsafe water source | Number | 2021 | 372753.9072 | 654884.7788 | 151767.9819 |
| DALYs (Disability-Adjusted Life Years) | Global | Both | 65+ years | diarrhea diseases | Unsafe water source | Rate | 2021 | 1436.97241 | 2464.125335 | 578.5239603 |
| DALYs (Disability-Adjusted Life Years) | Global | Both | 65+ years | diarrhea diseases | Unsafe water source | Percent | 2021 | 1.318660767 | 1.731263117 | 0.668351343 |
| DALYs (Disability-Adjusted Life Years) | Global | Both | 65+ years | diarrhea diseases | Unsafe water source | Number | 2021 | 6073713.5 | 10435714.9 | 2469419.356 |
| Deaths | Middle SDI | Both | 65+ years | diarrhea diseases | Unsafe water source | Rate | 2020 | 45.66756092 | 81.87604331 | 12.50584049 |
| Deaths | Middle SDI | Both | 65+ years | diarrhea diseases | Unsafe water source | Percent | 2020 | 1.140698888 | 1.674400753 | 0.438205013 |
| Deaths | Middle SDI | Both | 65+ years | diarrhea diseases | Unsafe water source | Number | 2020 | 57864.39625 | 103808.0435 | 15844.25711 |
| DALYs (Disability-Adjusted Life Years) | Middle SDI | Both | 65+ years | diarrhea diseases | Unsafe water source | Rate | 2020 | 778.2431101 | 1345.428843 | 229.0043134 |
| DALYs (Disability-Adjusted Life Years) | Middle SDI | Both | 65+ years | diarrhea diseases | Unsafe water source | Percent | 2020 | 1.138227445 | 1.672595402 | 0.437581348 |
| DALYs (Disability-Adjusted Life Years) | Middle SDI | Both | 65+ years | diarrhea diseases | Unsafe water source | Number | 2020 | 940353.8184 | 1629204.573 | 275872.9445 |
| Deaths | Low-middle SDI | Both | 65+ years | diarrhea diseases | Unsafe water source | Rate | 2020 | 330.4942552 | 595.0112629 | 132.7112623 |
| Deaths | Low-middle SDI | Both | 65+ years | diarrhea diseases | Unsafe water source | Percent | 2020 | 1.407640746 | 1.813879353 | 0.729771442 |
| Deaths | Low-middle SDI | Both | 65+ years | diarrhea diseases | Unsafe water source | Number | 2020 | 211356.015 | 381066.8071 | 85431.42496 |
| DALYs (Disability-Adjusted Life Years) | Low-middle SDI | Both | 65+ years | diarrhea diseases | Unsafe water source | Rate | 2020 | 5462.884336 | 9637.812137 | 2217.254741 |
| DALYs (Disability-Adjusted Life Years) | Low-middle SDI | Both | 65+ years | diarrhea diseases | Unsafe water source | Percent | 2020 | 1.406831606 | 1.814286084 | 0.729423327 |
| DALYs (Disability-Adjusted Life Years) | Low-middle SDI | Both | 65+ years | diarrhea diseases | Unsafe water source | Number | 2020 | 3353877.304 | 5932122.085 | 1374176.625 |
| Deaths | Low SDI | Both | 65+ years | diarrhea diseases | Unsafe water source | Rate | 2020 | 532.9500716 | 930.5024711 | 219.1741599 |
| Deaths | Low SDI | Both | 65+ years | diarrhea diseases | Unsafe water source | Percent | 2020 | 1.500944114 | 1.85317629 | 0.840205228 |
| Deaths | Low SDI | Both | 65+ years | diarrhea diseases | Unsafe water source | Number | 2020 | 106564.0312 | 185863.7548 | 43870.91502 |
| DALYs (Disability-Adjusted Life Years) | Low SDI | Both | 65+ years | diarrhea diseases | Unsafe water source | Rate | 2020 | 9200.221376 | 15941.98411 | 3831.587826 |
| DALYs (Disability-Adjusted Life Years) | Low SDI | Both | 65+ years | diarrhea diseases | Unsafe water source | Percent | 2020 | 1.500658343 | 1.853418133 | 0.840043097 |
| DALYs (Disability-Adjusted Life Years) | Low SDI | Both | 65+ years | diarrhea diseases | Unsafe water source | Number | 2020 | 1777810.482 | 3079071.404 | 739754.5653 |
| Deaths | High-middle SDI | Both | 65+ years | diarrhea diseases | Unsafe water source | Rate | 2020 | 3.664636231 | 7.201177116 | 0.840796638 |
| Deaths | High-middle SDI | Both | 65+ years | diarrhea diseases | Unsafe water source | Percent | 2020 | 0.742842245 | 1.23050778 | 0.231392111 |
| Deaths | High-middle SDI | Both | 65+ years | diarrhea diseases | Unsafe water source | Number | 2020 | 3819.487626 | 7523.475859 | 874.9831588 |
| DALYs (Disability-Adjusted Life Years) | High-middle SDI | Both | 65+ years | diarrhea diseases | Unsafe water source | Rate | 2020 | 68.73090962 | 129.1916532 | 17.44898923 |
| DALYs (Disability-Adjusted Life Years) | High-middle SDI | Both | 65+ years | diarrhea diseases | Unsafe water source | Percent | 2020 | 0.747735002 | 1.231021473 | 0.235045432 |
| DALYs (Disability-Adjusted Life Years) | High-middle SDI | Both | 65+ years | diarrhea diseases | Unsafe water source | Number | 2020 | 66851.40389 | 126646.831 | 16746.61683 |
| Deaths | High SDI | Both | 65+ years | diarrhea diseases | Unsafe water source | Rate | 2020 | 1.364846382 | 3.284420169 | 0.248688538 |
| Deaths | High SDI | Both | 65+ years | diarrhea diseases | Unsafe water source | Percent | 2020 | 0.153839853 | 0.37511139 | 0.029343514 |
| Deaths | High SDI | Both | 65+ years | diarrhea diseases | Unsafe water source | Number | 2020 | 1775.539413 | 4275.223491 | 321.7546945 |
| DALYs (Disability-Adjusted Life Years) | High SDI | Both | 65+ years | diarrhea diseases | Unsafe water source | Rate | 2020 | 24.40598149 | 57.96603382 | 4.565497042 |
| DALYs (Disability-Adjusted Life Years) | High SDI | Both | 65+ years | diarrhea diseases | Unsafe water source | Percent | 2020 | 0.166112691 | 0.394552474 | 0.03205351 |
| DALYs (Disability-Adjusted Life Years) | High SDI | Both | 65+ years | diarrhea diseases | Unsafe water source | Number | 2020 | 28892.51694 | 68644.35124 | 5371.378198 |
| Deaths | Global | Both | 65+ years | diarrhea diseases | Unsafe water source | Rate | 2020 | 87.84665129 | 152.8389699 | 35.41700286 |
| Deaths | Global | Both | 65+ years | diarrhea diseases | Unsafe water source | Percent | 2020 | 1.333611614 | 1.741038633 | 0.687782453 |
| Deaths | Global | Both | 65+ years | diarrhea diseases | Unsafe water source | Number | 2020 | 381515.2145 | 665489.5985 | 155135.9255 |
| DALYs (Disability-Adjusted Life Years) | Global | Both | 65+ years | diarrhea diseases | Unsafe water source | Rate | 2020 | 1504.402631 | 2561.933686 | 607.5594252 |
| DALYs (Disability-Adjusted Life Years) | Global | Both | 65+ years | diarrhea diseases | Unsafe water source | Percent | 2020 | 1.327349465 | 1.734930553 | 0.683300272 |
| DALYs (Disability-Adjusted Life Years) | Global | Both | 65+ years | diarrhea diseases | Unsafe water source | Number | 2020 | 6169991.912 | 10546405.74 | 2517341.983 |
| Deaths | Middle SDI | Both | 65+ years | diarrhea diseases | Unsafe water source | Rate | 2019 | 47.31915561 | 83.83565086 | 12.93739765 |
| Deaths | Middle SDI | Both | 65+ years | diarrhea diseases | Unsafe water source | Percent | 2019 | 1.14895537 | 1.677270316 | 0.453660037 |
| Deaths | Middle SDI | Both | 65+ years | diarrhea diseases | Unsafe water source | Number | 2019 | 57704.73519 | 102268.8464 | 15727.95897 |
| DALYs (Disability-Adjusted Life Years) | Middle SDI | Both | 65+ years | diarrhea diseases | Unsafe water source | Rate | 2019 | 803.9513904 | 1385.521823 | 235.3295067 |
| DALYs (Disability-Adjusted Life Years) | Middle SDI | Both | 65+ years | diarrhea diseases | Unsafe water source | Percent | 2019 | 1.146664559 | 1.6752419 | 0.453117371 |
| DALYs (Disability-Adjusted Life Years) | Middle SDI | Both | 65+ years | diarrhea diseases | Unsafe water source | Number | 2019 | 932681.185 | 1608149.57 | 271681.9654 |
| Deaths | Low-middle SDI | Both | 65+ years | diarrhea diseases | Unsafe water source | Rate | 2019 | 347.592403 | 614.8846462 | 141.1120338 |
| Deaths | Low-middle SDI | Both | 65+ years | diarrhea diseases | Unsafe water source | Percent | 2019 | 1.413066308 | 1.8209302 | 0.735968673 |
| Deaths | Low-middle SDI | Both | 65+ years | diarrhea diseases | Unsafe water source | Number | 2019 | 216243.0522 | 382231.5911 | 88385.68977 |
| DALYs (Disability-Adjusted Life Years) | Low-middle SDI | Both | 65+ years | diarrhea diseases | Unsafe water source | Rate | 2019 | 5750.190977 | 10004.42236 | 2342.745081 |
| DALYs (Disability-Adjusted Life Years) | Low-middle SDI | Both | 65+ years | diarrhea diseases | Unsafe water source | Percent | 2019 | 1.412374969 | 1.819226911 | 0.735700244 |
| DALYs (Disability-Adjusted Life Years) | Low-middle SDI | Both | 65+ years | diarrhea diseases | Unsafe water source | Number | 2019 | 3429578.218 | 5965130.625 | 1412364.587 |
| Deaths | Low SDI | Both | 65+ years | diarrhea diseases | Unsafe water source | Rate | 2019 | 559.5103968 | 975.6525247 | 235.3519453 |
| Deaths | Low SDI | Both | 65+ years | diarrhea diseases | Unsafe water source | Percent | 2019 | 1.503568755 | 1.848086609 | 0.848233871 |
| Deaths | Low SDI | Both | 65+ years | diarrhea diseases | Unsafe water source | Number | 2019 | 109180.0573 | 190090.4863 | 46009.87533 |
| DALYs (Disability-Adjusted Life Years) | Low SDI | Both | 65+ years | diarrhea diseases | Unsafe water source | Rate | 2019 | 9655.447071 | 16809.72037 | 4118.862663 |
| DALYs (Disability-Adjusted Life Years) | Low SDI | Both | 65+ years | diarrhea diseases | Unsafe water source | Percent | 2019 | 1.503278723 | 1.848092205 | 0.848085874 |
| DALYs (Disability-Adjusted Life Years) | Low SDI | Both | 65+ years | diarrhea diseases | Unsafe water source | Number | 2019 | 1817812.434 | 3162612.552 | 775863.3881 |
| Deaths | High-middle SDI | Both | 65+ years | diarrhea diseases | Unsafe water source | Rate | 2019 | 3.760312101 | 7.332220953 | 0.892125269 |
| Deaths | High-middle SDI | Both | 65+ years | diarrhea diseases | Unsafe water source | Percent | 2019 | 0.743567657 | 1.233917156 | 0.228873614 |
| Deaths | High-middle SDI | Both | 65+ years | diarrhea diseases | Unsafe water source | Number | 2019 | 3791.954433 | 7416.474367 | 896.8906782 |
| DALYs (Disability-Adjusted Life Years) | High-middle SDI | Both | 65+ years | diarrhea diseases | Unsafe water source | Rate | 2019 | 70.05152051 | 130.8366574 | 18.31543998 |
| DALYs (Disability-Adjusted Life Years) | High-middle SDI | Both | 65+ years | diarrhea diseases | Unsafe water source | Percent | 2019 | 0.746644123 | 1.232376749 | 0.236534452 |
| DALYs (Disability-Adjusted Life Years) | High-middle SDI | Both | 65+ years | diarrhea diseases | Unsafe water source | Number | 2019 | 65876.69517 | 124112.9333 | 17123.2645 |
| Deaths | High SDI | Both | 65+ years | diarrhea diseases | Unsafe water source | Rate | 2019 | 1.403708215 | 3.366185208 | 0.256426216 |
| Deaths | High SDI | Both | 65+ years | diarrhea diseases | Unsafe water source | Percent | 2019 | 0.155428675 | 0.377522673 | 0.029939419 |
| Deaths | High SDI | Both | 65+ years | diarrhea diseases | Unsafe water source | Number | 2019 | 1772.009089 | 4251.706287 | 321.6900637 |
| DALYs (Disability-Adjusted Life Years) | High SDI | Both | 65+ years | diarrhea diseases | Unsafe water source | Rate | 2019 | 24.92614577 | 59.45932411 | 4.707628206 |
| DALYs (Disability-Adjusted Life Years) | High SDI | Both | 65+ years | diarrhea diseases | Unsafe water source | Percent | 2019 | 0.166356304 | 0.397433397 | 0.032543171 |
| DALYs (Disability-Adjusted Life Years) | High SDI | Both | 65+ years | diarrhea diseases | Unsafe water source | Number | 2019 | 28687.89487 | 68306.16766 | 5371.213805 |
| Deaths | Global | Both | 65+ years | diarrhea diseases | Unsafe water source | Rate | 2019 | 92.5945746 | 160.1847329 | 37.48493136 |
| Deaths | Global | Both | 65+ years | diarrhea diseases | Unsafe water source | Percent | 2019 | 1.341200042 | 1.74552338 | 0.700197367 |
| Deaths | Global | Both | 65+ years | diarrhea diseases | Unsafe water source | Number | 2019 | 388826.911 | 672594.2335 | 158901.883 |
| DALYs (Disability-Adjusted Life Years) | Global | Both | 65+ years | diarrhea diseases | Unsafe water source | Rate | 2019 | 1584.733078 | 2689.41033 | 643.7228882 |
| DALYs (Disability-Adjusted Life Years) | Global | Both | 65+ years | diarrhea diseases | Unsafe water source | Percent | 2019 | 1.335109558 | 1.741064574 | 0.696193471 |
| DALYs (Disability-Adjusted Life Years) | Global | Both | 65+ years | diarrhea diseases | Unsafe water source | Number | 2019 | 6276822.952 | 10654419.67 | 2580497.12 |
| Deaths | Middle SDI | Both | 65+ years | diarrhea diseases | Unsafe water source | Rate | 2018 | 49.41274813 | 87.23513949 | 13.42910163 |
| Deaths | Middle SDI | Both | 65+ years | diarrhea diseases | Unsafe water source | Percent | 2018 | 1.16014694 | 1.682789963 | 0.469813635 |
| Deaths | Middle SDI | Both | 65+ years | diarrhea diseases | Unsafe water source | Number | 2018 | 57451.72805 | 101530.7391 | 15588.91067 |
| DALYs (Disability-Adjusted Life Years) | Middle SDI | Both | 65+ years | diarrhea diseases | Unsafe water source | Rate | 2018 | 835.4406555 | 1434.945496 | 241.2319181 |
| DALYs (Disability-Adjusted Life Years) | Middle SDI | Both | 65+ years | diarrhea diseases | Unsafe water source | Percent | 2018 | 1.158031967 | 1.682314091 | 0.469002393 |
| DALYs (Disability-Adjusted Life Years) | Middle SDI | Both | 65+ years | diarrhea diseases | Unsafe water source | Number | 2018 | 925336.4496 | 1591612.073 | 266260.4224 |
| Deaths | Low-middle SDI | Both | 65+ years | diarrhea diseases | Unsafe water source | Rate | 2018 | 368.1803067 | 651.5992731 | 150.1396815 |
| Deaths | Low-middle SDI | Both | 65+ years | diarrhea diseases | Unsafe water source | Percent | 2018 | 1.418722962 | 1.8154759 | 0.742924414 |
| Deaths | Low-middle SDI | Both | 65+ years | diarrhea diseases | Unsafe water source | Number | 2018 | 221275.4957 | 391424.0121 | 90991.65651 |
| DALYs (Disability-Adjusted Life Years) | Low-middle SDI | Both | 65+ years | diarrhea diseases | Unsafe water source | Rate | 2018 | 6102.840498 | 10698.95302 | 2463.165182 |
| DALYs (Disability-Adjusted Life Years) | Low-middle SDI | Both | 65+ years | diarrhea diseases | Unsafe water source | Percent | 2018 | 1.418099966 | 1.815433402 | 0.742853177 |
| DALYs (Disability-Adjusted Life Years) | Low-middle SDI | Both | 65+ years | diarrhea diseases | Unsafe water source | Number | 2018 | 3516518.143 | 6165763.642 | 1436785.564 |
| Deaths | Low SDI | Both | 65+ years | diarrhea diseases | Unsafe water source | Rate | 2018 | 589.4793109 | 1017.924752 | 252.8124776 |
| Deaths | Low SDI | Both | 65+ years | diarrhea diseases | Unsafe water source | Percent | 2018 | 1.50631348 | 1.850878083 | 0.853938247 |
| Deaths | Low SDI | Both | 65+ years | diarrhea diseases | Unsafe water source | Number | 2018 | 111388.1081 | 191996.0898 | 47835.64252 |
| DALYs (Disability-Adjusted Life Years) | Low SDI | Both | 65+ years | diarrhea diseases | Unsafe water source | Rate | 2018 | 10168.63405 | 17458.61513 | 4395.263375 |
| DALYs (Disability-Adjusted Life Years) | Low SDI | Both | 65+ years | diarrhea diseases | Unsafe water source | Percent | 2018 | 1.506075284 | 1.850191876 | 0.853605914 |
| DALYs (Disability-Adjusted Life Years) | Low SDI | Both | 65+ years | diarrhea diseases | Unsafe water source | Number | 2018 | 1853350.522 | 3176171.169 | 801717.0907 |
| Deaths | High-middle SDI | Both | 65+ years | diarrhea diseases | Unsafe water source | Rate | 2018 | 3.807778271 | 7.290192268 | 0.906240407 |
| Deaths | High-middle SDI | Both | 65+ years | diarrhea diseases | Unsafe water source | Percent | 2018 | 0.744989669 | 1.233541419 | 0.229921805 |
| Deaths | High-middle SDI | Both | 65+ years | diarrhea diseases | Unsafe water source | Number | 2018 | 3696.99862 | 7082.899515 | 878.6045757 |
| DALYs (Disability-Adjusted Life Years) | High-middle SDI | Both | 65+ years | diarrhea diseases | Unsafe water source | Rate | 2018 | 70.72940369 | 129.2289896 | 18.29520871 |
| DALYs (Disability-Adjusted Life Years) | High-middle SDI | Both | 65+ years | diarrhea diseases | Unsafe water source | Percent | 2018 | 0.745639668 | 1.22974559 | 0.235630781 |
| DALYs (Disability-Adjusted Life Years) | High-middle SDI | Both | 65+ years | diarrhea diseases | Unsafe water source | Number | 2018 | 64125.78074 | 117471.9961 | 16473.68671 |
| Deaths | High SDI | Both | 65+ years | diarrhea diseases | Unsafe water source | Rate | 2018 | 1.464403561 | 3.511248696 | 0.265559882 |
| Deaths | High SDI | Both | 65+ years | diarrhea diseases | Unsafe water source | Percent | 2018 | 0.152354464 | 0.370108523 | 0.029239931 |
| Deaths | High SDI | Both | 65+ years | diarrhea diseases | Unsafe water source | Number | 2018 | 1793.463811 | 4297.803859 | 323.1343887 |
| DALYs (Disability-Adjusted Life Years) | High SDI | Both | 65+ years | diarrhea diseases | Unsafe water source | Rate | 2018 | 25.8182832 | 61.58026762 | 4.830636507 |
| DALYs (Disability-Adjusted Life Years) | High SDI | Both | 65+ years | diarrhea diseases | Unsafe water source | Percent | 2018 | 0.162547913 | 0.389186897 | 0.031591216 |
| DALYs (Disability-Adjusted Life Years) | High SDI | Both | 65+ years | diarrhea diseases | Unsafe water source | Number | 2018 | 28935.41725 | 68971.16392 | 5368.225533 |
| Deaths | Global | Both | 65+ years | diarrhea diseases | Unsafe water source | Rate | 2018 | 97.92439348 | 166.9478228 | 39.71407218 |
| Deaths | Global | Both | 65+ years | diarrhea diseases | Unsafe water source | Percent | 2018 | 1.347850731 | 1.747368655 | 0.712357882 |
| Deaths | Global | Both | 65+ years | diarrhea diseases | Unsafe water source | Number | 2018 | 395740.0528 | 673818.8693 | 161736.9447 |
| DALYs (Disability-Adjusted Life Years) | Global | Both | 65+ years | diarrhea diseases | Unsafe water source | Rate | 2018 | 1674.366378 | 2813.714785 | 676.9276087 |
| DALYs (Disability-Adjusted Life Years) | Global | Both | 65+ years | diarrhea diseases | Unsafe water source | Percent | 2018 | 1.341909452 | 1.74261322 | 0.707928108 |
| DALYs (Disability-Adjusted Life Years) | Global | Both | 65+ years | diarrhea diseases | Unsafe water source | Number | 2018 | 6390434.725 | 10732310.84 | 2611536.912 |
| Deaths | Middle SDI | Both | 65+ years | diarrhea diseases | Unsafe water source | Rate | 2017 | 51.43662823 | 92.92158523 | 14.02555034 |
| Deaths | Middle SDI | Both | 65+ years | diarrhea diseases | Unsafe water source | Percent | 2017 | 1.169977376 | 1.684727255 | 0.477343626 |
| Deaths | Middle SDI | Both | 65+ years | diarrhea diseases | Unsafe water source | Number | 2017 | 57357.9315 | 103677.1876 | 15607.96803 |
| DALYs (Disability-Adjusted Life Years) | Middle SDI | Both | 65+ years | diarrhea diseases | Unsafe water source | Rate | 2017 | 865.6882788 | 1515.161299 | 253.952445 |
| DALYs (Disability-Adjusted Life Years) | Middle SDI | Both | 65+ years | diarrhea diseases | Unsafe water source | Percent | 2017 | 1.168145814 | 1.683028887 | 0.477060394 |
| DALYs (Disability-Adjusted Life Years) | Middle SDI | Both | 65+ years | diarrhea diseases | Unsafe water source | Number | 2017 | 918677.0891 | 1612777.723 | 268708.2981 |
| Deaths | Low-middle SDI | Both | 65+ years | diarrhea diseases | Unsafe water source | Rate | 2017 | 388.3283442 | 685.9800295 | 159.3687078 |
| Deaths | Low-middle SDI | Both | 65+ years | diarrhea diseases | Unsafe water source | Percent | 2017 | 1.423947856 | 1.817672769 | 0.753388501 |
| Deaths | Low-middle SDI | Both | 65+ years | diarrhea diseases | Unsafe water source | Number | 2017 | 225651.6439 | 398537.2897 | 93359.22498 |
| DALYs (Disability-Adjusted Life Years) | Low-middle SDI | Both | 65+ years | diarrhea diseases | Unsafe water source | Rate | 2017 | 6415.027512 | 11198.93407 | 2616.254469 |
| DALYs (Disability-Adjusted Life Years) | Low-middle SDI | Both | 65+ years | diarrhea diseases | Unsafe water source | Percent | 2017 | 1.423277088 | 1.818122083 | 0.753182802 |
| DALYs (Disability-Adjusted Life Years) | Low-middle SDI | Both | 65+ years | diarrhea diseases | Unsafe water source | Number | 2017 | 3573754.294 | 6241241.818 | 1474216.561 |
| Deaths | Low SDI | Both | 65+ years | diarrhea diseases | Unsafe water source | Rate | 2017 | 627.5999945 | 1082.919615 | 271.1400301 |
| Deaths | Low SDI | Both | 65+ years | diarrhea diseases | Unsafe water source | Percent | 2017 | 1.508675569 | 1.85210533 | 0.861285436 |
| Deaths | Low SDI | Both | 65+ years | diarrhea diseases | Unsafe water source | Number | 2017 | 114947.8662 | 198075.9815 | 49710.4613 |
| DALYs (Disability-Adjusted Life Years) | Low SDI | Both | 65+ years | diarrhea diseases | Unsafe water source | Rate | 2017 | 10747.46479 | 18504.92823 | 4682.938698 |
| DALYs (Disability-Adjusted Life Years) | Low SDI | Both | 65+ years | diarrhea diseases | Unsafe water source | Percent | 2017 | 1.508478652 | 1.852452644 | 0.861028733 |
| DALYs (Disability-Adjusted Life Years) | Low SDI | Both | 65+ years | diarrhea diseases | Unsafe water source | Number | 2017 | 1898228.405 | 3266207.379 | 827857.1956 |
| Deaths | High-middle SDI | Both | 65+ years | diarrhea diseases | Unsafe water source | Rate | 2017 | 3.861972242 | 7.405683469 | 0.92623441 |
| Deaths | High-middle SDI | Both | 65+ years | diarrhea diseases | Unsafe water source | Percent | 2017 | 0.747962961 | 1.232952814 | 0.235850026 |
| Deaths | High-middle SDI | Both | 65+ years | diarrhea diseases | Unsafe water source | Number | 2017 | 3636.981021 | 6983.118362 | 867.5745813 |
| DALYs (Disability-Adjusted Life Years) | High-middle SDI | Both | 65+ years | diarrhea diseases | Unsafe water source | Rate | 2017 | 71.56108535 | 132.9766809 | 19.14037303 |
| DALYs (Disability-Adjusted Life Years) | High-middle SDI | Both | 65+ years | diarrhea diseases | Unsafe water source | Percent | 2017 | 0.74423128 | 1.221725303 | 0.239628167 |
| DALYs (Disability-Adjusted Life Years) | High-middle SDI | Both | 65+ years | diarrhea diseases | Unsafe water source | Number | 2017 | 62789.45097 | 117536.7524 | 16695.13331 |
| Deaths | High SDI | Both | 65+ years | diarrhea diseases | Unsafe water source | Rate | 2017 | 1.510576856 | 3.650110177 | 0.273393988 |
| Deaths | High SDI | Both | 65+ years | diarrhea diseases | Unsafe water source | Percent | 2017 | 0.149025836 | 0.365963502 | 0.028378643 |
| Deaths | High SDI | Both | 65+ years | diarrhea diseases | Unsafe water source | Number | 2017 | 1791.743831 | 4326.100069 | 322.2764164 |
| DALYs (Disability-Adjusted Life Years) | High SDI | Both | 65+ years | diarrhea diseases | Unsafe water source | Rate | 2017 | 26.61352103 | 63.96167569 | 4.979400901 |
| DALYs (Disability-Adjusted Life Years) | High SDI | Both | 65+ years | diarrhea diseases | Unsafe water source | Percent | 2017 | 0.157738339 | 0.382412836 | 0.030447099 |
| DALYs (Disability-Adjusted Life Years) | High SDI | Both | 65+ years | diarrhea diseases | Unsafe water source | Number | 2017 | 28996.10893 | 69589.7672 | 5385.015721 |
| Deaths | Global | Both | 65+ years | diarrhea diseases | Unsafe water source | Rate | 2017 | 103.3227301 | 176.4880925 | 42.08663715 |
| Deaths | Global | Both | 65+ years | diarrhea diseases | Unsafe water source | Percent | 2017 | 1.353983367 | 1.747382832 | 0.71978247 |
| Deaths | Global | Both | 65+ years | diarrhea diseases | Unsafe water source | Number | 2017 | 403519.4031 | 688364.5482 | 165324.0093 |
| DALYs (Disability-Adjusted Life Years) | Global | Both | 65+ years | diarrhea diseases | Unsafe water source | Rate | 2017 | 1758.19648 | 2967.049002 | 718.2434822 |
| DALYs (Disability-Adjusted Life Years) | Global | Both | 65+ years | diarrhea diseases | Unsafe water source | Percent | 2017 | 1.347356649 | 1.742647135 | 0.715360731 |
| DALYs (Disability-Adjusted Life Years) | Global | Both | 65+ years | diarrhea diseases | Unsafe water source | Number | 2017 | 6484594.136 | 10919343.67 | 2668902.023 |
| Deaths | Middle SDI | Both | 65+ years | diarrhea diseases | Unsafe water source | Rate | 2016 | 53.40442647 | 95.67410946 | 14.94638807 |
| Deaths | Middle SDI | Both | 65+ years | diarrhea diseases | Unsafe water source | Percent | 2016 | 1.180208539 | 1.693112088 | 0.483426675 |
| Deaths | Middle SDI | Both | 65+ years | diarrhea diseases | Unsafe water source | Number | 2016 | 57180.28244 | 102554.5708 | 15955.23251 |
| DALYs (Disability-Adjusted Life Years) | Middle SDI | Both | 65+ years | diarrhea diseases | Unsafe water source | Rate | 2016 | 894.4267655 | 1554.115976 | 262.2569455 |
| DALYs (Disability-Adjusted Life Years) | Middle SDI | Both | 65+ years | diarrhea diseases | Unsafe water source | Percent | 2016 | 1.178613486 | 1.692900643 | 0.482611898 |
| DALYs (Disability-Adjusted Life Years) | Middle SDI | Both | 65+ years | diarrhea diseases | Unsafe water source | Number | 2016 | 910917.0973 | 1589599.267 | 265383.8863 |
| Deaths | Low-middle SDI | Both | 65+ years | diarrhea diseases | Unsafe water source | Rate | 2016 | 406.8121693 | 713.1024531 | 166.2373811 |
| Deaths | Low-middle SDI | Both | 65+ years | diarrhea diseases | Unsafe water source | Percent | 2016 | 1.428849249 | 1.822643873 | 0.757579703 |
| Deaths | Low-middle SDI | Both | 65+ years | diarrhea diseases | Unsafe water source | Number | 2016 | 228314.2221 | 399470.1516 | 93833.43628 |
| DALYs (Disability-Adjusted Life Years) | Low-middle SDI | Both | 65+ years | diarrhea diseases | Unsafe water source | Rate | 2016 | 6699.466427 | 11623.12645 | 2741.482809 |
| DALYs (Disability-Adjusted Life Years) | Low-middle SDI | Both | 65+ years | diarrhea diseases | Unsafe water source | Percent | 2016 | 1.428221328 | 1.821879964 | 0.757327844 |
| DALYs (Disability-Adjusted Life Years) | Low-middle SDI | Both | 65+ years | diarrhea diseases | Unsafe water source | Number | 2016 | 3602601.859 | 6236716.503 | 1487681.227 |
| Deaths | Low SDI | Both | 65+ years | diarrhea diseases | Unsafe water source | Rate | 2016 | 659.4068126 | 1133.140344 | 284.296305 |
| Deaths | Low SDI | Both | 65+ years | diarrhea diseases | Unsafe water source | Percent | 2016 | 1.51143556 | 1.852000483 | 0.860598658 |
| Deaths | Low SDI | Both | 65+ years | diarrhea diseases | Unsafe water source | Number | 2016 | 116931.2031 | 200950.3761 | 50498.90917 |
| DALYs (Disability-Adjusted Life Years) | Low SDI | Both | 65+ years | diarrhea diseases | Unsafe water source | Rate | 2016 | 11235.04533 | 19158.80712 | 4898.232075 |
| DALYs (Disability-Adjusted Life Years) | Low SDI | Both | 65+ years | diarrhea diseases | Unsafe water source | Percent | 2016 | 1.51126797 | 1.850911641 | 0.861565771 |
| DALYs (Disability-Adjusted Life Years) | Low SDI | Both | 65+ years | diarrhea diseases | Unsafe water source | Number | 2016 | 1919309.331 | 3275444.266 | 837862.4834 |
| Deaths | High-middle SDI | Both | 65+ years | diarrhea diseases | Unsafe water source | Rate | 2016 | 3.867347913 | 7.285760797 | 0.95110026 |
| Deaths | High-middle SDI | Both | 65+ years | diarrhea diseases | Unsafe water source | Percent | 2016 | 0.761584107 | 1.246484523 | 0.237388081 |
| Deaths | High-middle SDI | Both | 65+ years | diarrhea diseases | Unsafe water source | Number | 2016 | 3527.379836 | 6649.555457 | 861.1145131 |
| DALYs (Disability-Adjusted Life Years) | High-middle SDI | Both | 65+ years | diarrhea diseases | Unsafe water source | Rate | 2016 | 71.70677355 | 131.1704377 | 19.06811956 |
| DALYs (Disability-Adjusted Life Years) | High-middle SDI | Both | 65+ years | diarrhea diseases | Unsafe water source | Percent | 2016 | 0.749901791 | 1.223662826 | 0.240766983 |
| DALYs (Disability-Adjusted Life Years) | High-middle SDI | Both | 65+ years | diarrhea diseases | Unsafe water source | Number | 2016 | 60874.41331 | 112052.8274 | 16059.38529 |
| Deaths | High SDI | Both | 65+ years | diarrhea diseases | Unsafe water source | Rate | 2016 | 1.538491401 | 3.777721886 | 0.276998842 |
| Deaths | High SDI | Both | 65+ years | diarrhea diseases | Unsafe water source | Percent | 2016 | 0.144443028 | 0.358532524 | 0.027138639 |
| Deaths | High SDI | Both | 65+ years | diarrhea diseases | Unsafe water source | Number | 2016 | 1766.527703 | 4334.869749 | 316.3574964 |
| DALYs (Disability-Adjusted Life Years) | High SDI | Both | 65+ years | diarrhea diseases | Unsafe water source | Rate | 2016 | 27.24221698 | 66.53355304 | 5.070150582 |
| DALYs (Disability-Adjusted Life Years) | High SDI | Both | 65+ years | diarrhea diseases | Unsafe water source | Percent | 2016 | 0.152101987 | 0.37284038 | 0.02861193 |
| DALYs (Disability-Adjusted Life Years) | High SDI | Both | 65+ years | diarrhea diseases | Unsafe water source | Number | 2016 | 28829.90962 | 70327.82595 | 5338.967031 |
| Deaths | Global | Both | 65+ years | diarrhea diseases | Unsafe water source | Rate | 2016 | 108.0954307 | 183.086125 | 44.02225903 |
| Deaths | Global | Both | 65+ years | diarrhea diseases | Unsafe water source | Percent | 2016 | 1.360074772 | 1.75129119 | 0.727406735 |
| Deaths | Global | Both | 65+ years | diarrhea diseases | Unsafe water source | Number | 2016 | 407852.2171 | 688223.4514 | 166860.9592 |
| DALYs (Disability-Adjusted Life Years) | Global | Both | 65+ years | diarrhea diseases | Unsafe water source | Rate | 2016 | 1830.851686 | 3080.240419 | 746.9952811 |
| DALYs (Disability-Adjusted Life Years) | Global | Both | 65+ years | diarrhea diseases | Unsafe water source | Percent | 2016 | 1.352514703 | 1.74512762 | 0.722779478 |
| DALYs (Disability-Adjusted Life Years) | Global | Both | 65+ years | diarrhea diseases | Unsafe water source | Number | 2016 | 6524668.834 | 10936388.64 | 2681954.071 |
| Deaths | Middle SDI | Both | 65+ years | diarrhea diseases | Unsafe water source | Rate | 2015 | 56.01701604 | 99.83350429 | 15.81084888 |
| Deaths | Middle SDI | Both | 65+ years | diarrhea diseases | Unsafe water source | Percent | 2015 | 1.192661974 | 1.698993301 | 0.502055317 |
| Deaths | Middle SDI | Both | 65+ years | diarrhea diseases | Unsafe water source | Number | 2015 | 57685.07914 | 102912.9012 | 16239.09905 |
| DALYs (Disability-Adjusted Life Years) | Middle SDI | Both | 65+ years | diarrhea diseases | Unsafe water source | Rate | 2015 | 934.3615825 | 1613.957381 | 274.515877 |
| DALYs (Disability-Adjusted Life Years) | Middle SDI | Both | 65+ years | diarrhea diseases | Unsafe water source | Percent | 2015 | 1.190987914 | 1.698026955 | 0.501205344 |
| DALYs (Disability-Adjusted Life Years) | Middle SDI | Both | 65+ years | diarrhea diseases | Unsafe water source | Number | 2015 | 914311.2841 | 1585575.81 | 266891.1928 |
| Deaths | Low-middle SDI | Both | 65+ years | diarrhea diseases | Unsafe water source | Rate | 2015 | 438.0511027 | 766.7293907 | 179.0930205 |
| Deaths | Low-middle SDI | Both | 65+ years | diarrhea diseases | Unsafe water source | Percent | 2015 | 1.434950479 | 1.822353099 | 0.765164516 |
| Deaths | Low-middle SDI | Both | 65+ years | diarrhea diseases | Unsafe water source | Number | 2015 | 237871.7074 | 414627.8387 | 97505.44608 |
| DALYs (Disability-Adjusted Life Years) | Low-middle SDI | Both | 65+ years | diarrhea diseases | Unsafe water source | Rate | 2015 | 7160.644356 | 12423.85903 | 2942.813859 |
| DALYs (Disability-Adjusted Life Years) | Low-middle SDI | Both | 65+ years | diarrhea diseases | Unsafe water source | Percent | 2015 | 1.434361296 | 1.820976351 | 0.764742389 |
| DALYs (Disability-Adjusted Life Years) | Low-middle SDI | Both | 65+ years | diarrhea diseases | Unsafe water source | Number | 2015 | 3721581.277 | 6418929.254 | 1536732.316 |
| Deaths | Low SDI | Both | 65+ years | diarrhea diseases | Unsafe water source | Rate | 2015 | 704.5984678 | 1211.087326 | 305.0346174 |
| Deaths | Low SDI | Both | 65+ years | diarrhea diseases | Unsafe water source | Percent | 2015 | 1.514288668 | 1.854506774 | 0.866379391 |
| Deaths | Low SDI | Both | 65+ years | diarrhea diseases | Unsafe water source | Number | 2015 | 120977.5972 | 207962.4942 | 52419.15095 |
| DALYs (Disability-Adjusted Life Years) | Low SDI | Both | 65+ years | diarrhea diseases | Unsafe water source | Rate | 2015 | 11940.63504 | 20361.57298 | 5233.442929 |
| DALYs (Disability-Adjusted Life Years) | Low SDI | Both | 65+ years | diarrhea diseases | Unsafe water source | Percent | 2015 | 1.514181833 | 1.854834534 | 0.866688556 |
| DALYs (Disability-Adjusted Life Years) | Low SDI | Both | 65+ years | diarrhea diseases | Unsafe water source | Number | 2015 | 1971167.006 | 3362026.954 | 864498.7628 |
| Deaths | High-middle SDI | Both | 65+ years | diarrhea diseases | Unsafe water source | Rate | 2015 | 3.88133913 | 7.338548113 | 0.984445138 |
| Deaths | High-middle SDI | Both | 65+ years | diarrhea diseases | Unsafe water source | Percent | 2015 | 0.779884128 | 1.26957629 | 0.248974078 |
| Deaths | High-middle SDI | Both | 65+ years | diarrhea diseases | Unsafe water source | Number | 2015 | 3439.583412 | 6511.612737 | 867.7398507 |
| DALYs (Disability-Adjusted Life Years) | High-middle SDI | Both | 65+ years | diarrhea diseases | Unsafe water source | Rate | 2015 | 72.42059731 | 132.1712878 | 19.71152669 |
| DALYs (Disability-Adjusted Life Years) | High-middle SDI | Both | 65+ years | diarrhea diseases | Unsafe water source | Percent | 2015 | 0.760073663 | 1.239117746 | 0.24801936 |
| DALYs (Disability-Adjusted Life Years) | High-middle SDI | Both | 65+ years | diarrhea diseases | Unsafe water source | Number | 2015 | 59469.09538 | 109114.3221 | 16036.3627 |
| Deaths | High SDI | Both | 65+ years | diarrhea diseases | Unsafe water source | Rate | 2015 | 1.555752707 | 3.884211947 | 0.281318209 |
| Deaths | High SDI | Both | 65+ years | diarrhea diseases | Unsafe water source | Percent | 2015 | 0.142133793 | 0.354156264 | 0.026359247 |
| Deaths | High SDI | Both | 65+ years | diarrhea diseases | Unsafe water source | Number | 2015 | 1752.618767 | 4377.154005 | 315.5052523 |
| DALYs (Disability-Adjusted Life Years) | High SDI | Both | 65+ years | diarrhea diseases | Unsafe water source | Rate | 2015 | 27.67496266 | 68.02169913 | 5.134472649 |
| DALYs (Disability-Adjusted Life Years) | High SDI | Both | 65+ years | diarrhea diseases | Unsafe water source | Percent | 2015 | 0.148743338 | 0.365075125 | 0.027861339 |
| DALYs (Disability-Adjusted Life Years) | High SDI | Both | 65+ years | diarrhea diseases | Unsafe water source | Number | 2015 | 28702.706 | 70398.07717 | 5296.478081 |
| Deaths | Global | Both | 65+ years | diarrhea diseases | Unsafe water source | Rate | 2015 | 115.2227438 | 198.3303153 | 46.61849259 |
| Deaths | Global | Both | 65+ years | diarrhea diseases | Unsafe water source | Percent | 2015 | 1.369085452 | 1.759878212 | 0.732772519 |
| Deaths | Global | Both | 65+ years | diarrhea diseases | Unsafe water source | Number | 2015 | 421858.8113 | 723504.47 | 171094.6891 |
| DALYs (Disability-Adjusted Life Years) | Global | Both | 65+ years | diarrhea diseases | Unsafe water source | Rate | 2015 | 1940.998038 | 3309.26563 | 789.2104414 |
| DALYs (Disability-Adjusted Life Years) | Global | Both | 65+ years | diarrhea diseases | Unsafe water source | Percent | 2015 | 1.360736841 | 1.75034496 | 0.728759467 |
| DALYs (Disability-Adjusted Life Years) | Global | Both | 65+ years | diarrhea diseases | Unsafe water source | Number | 2015 | 6697356.776 | 11381516.29 | 2737330.309 |
| Deaths | Middle SDI | Both | 65+ years | diarrhea diseases | Unsafe water source | Rate | 2014 | 58.91755198 | 103.3088552 | 17.11243122 |
| Deaths | Middle SDI | Both | 65+ years | diarrhea diseases | Unsafe water source | Percent | 2014 | 1.203034739 | 1.703948263 | 0.502153511 |
| Deaths | Middle SDI | Both | 65+ years | diarrhea diseases | Unsafe water source | Number | 2014 | 58691.37085 | 102928.78 | 17005.43692 |
| DALYs (Disability-Adjusted Life Years) | Middle SDI | Both | 65+ years | diarrhea diseases | Unsafe water source | Rate | 2014 | 979.6330786 | 1659.267816 | 293.3980363 |
| DALYs (Disability-Adjusted Life Years) | Middle SDI | Both | 65+ years | diarrhea diseases | Unsafe water source | Percent | 2014 | 1.201315122 | 1.702957279 | 0.501444326 |
| DALYs (Disability-Adjusted Life Years) | Middle SDI | Both | 65+ years | diarrhea diseases | Unsafe water source | Number | 2014 | 927273.5935 | 1573082.022 | 275918.5451 |
| Deaths | Low-middle SDI | Both | 65+ years | diarrhea diseases | Unsafe water source | Rate | 2014 | 477.2220316 | 833.592443 | 193.9151179 |
| Deaths | Low-middle SDI | Both | 65+ years | diarrhea diseases | Unsafe water source | Percent | 2014 | 1.439024901 | 1.825565457 | 0.773622713 |
| Deaths | Low-middle SDI | Both | 65+ years | diarrhea diseases | Unsafe water source | Number | 2014 | 252191.5905 | 437921.8557 | 102583.7441 |
| DALYs (Disability-Adjusted Life Years) | Low-middle SDI | Both | 65+ years | diarrhea diseases | Unsafe water source | Rate | 2014 | 7733.489262 | 13521.90045 | 3180.350726 |
| DALYs (Disability-Adjusted Life Years) | Low-middle SDI | Both | 65+ years | diarrhea diseases | Unsafe water source | Percent | 2014 | 1.438508873 | 1.824157145 | 0.773228802 |
| DALYs (Disability-Adjusted Life Years) | Low-middle SDI | Both | 65+ years | diarrhea diseases | Unsafe water source | Number | 2014 | 3912620.675 | 6793593.182 | 1612117.294 |
| Deaths | Low SDI | Both | 65+ years | diarrhea diseases | Unsafe water source | Rate | 2014 | 756.7893233 | 1291.298325 | 333.1666385 |
| Deaths | Low SDI | Both | 65+ years | diarrhea diseases | Unsafe water source | Percent | 2014 | 1.515944581 | 1.854915725 | 0.869038116 |
| Deaths | Low SDI | Both | 65+ years | diarrhea diseases | Unsafe water source | Number | 2014 | 126820.0866 | 216224.469 | 55858.4087 |
| DALYs (Disability-Adjusted Life Years) | Low SDI | Both | 65+ years | diarrhea diseases | Unsafe water source | Rate | 2014 | 12735.80248 | 21581.44441 | 5686.625239 |
| DALYs (Disability-Adjusted Life Years) | Low SDI | Both | 65+ years | diarrhea diseases | Unsafe water source | Percent | 2014 | 1.515871733 | 1.855313792 | 0.870077965 |
| DALYs (Disability-Adjusted Life Years) | Low SDI | Both | 65+ years | diarrhea diseases | Unsafe water source | Number | 2014 | 2053612.589 | 3478594.143 | 917708.1862 |
| Deaths | High-middle SDI | Both | 65+ years | diarrhea diseases | Unsafe water source | Rate | 2014 | 3.849908052 | 7.23658442 | 0.975603174 |
| Deaths | High-middle SDI | Both | 65+ years | diarrhea diseases | Unsafe water source | Percent | 2014 | 0.799372677 | 1.299491584 | 0.260708289 |
| Deaths | High-middle SDI | Both | 65+ years | diarrhea diseases | Unsafe water source | Number | 2014 | 3335.646251 | 6273.274956 | 841.622177 |
| DALYs (Disability-Adjusted Life Years) | High-middle SDI | Both | 65+ years | diarrhea diseases | Unsafe water source | Rate | 2014 | 72.58961102 | 131.6788595 | 20.03431735 |
| DALYs (Disability-Adjusted Life Years) | High-middle SDI | Both | 65+ years | diarrhea diseases | Unsafe water source | Percent | 2014 | 0.773132548 | 1.255801879 | 0.257028965 |
| DALYs (Disability-Adjusted Life Years) | High-middle SDI | Both | 65+ years | diarrhea diseases | Unsafe water source | Number | 2014 | 57939.64989 | 105483.8421 | 15814.02925 |
| Deaths | High SDI | Both | 65+ years | diarrhea diseases | Unsafe water source | Rate | 2014 | 1.556238566 | 3.892217416 | 0.282803232 |
| Deaths | High SDI | Both | 65+ years | diarrhea diseases | Unsafe water source | Percent | 2014 | 0.141194375 | 0.353076934 | 0.025751441 |
| Deaths | High SDI | Both | 65+ years | diarrhea diseases | Unsafe water source | Number | 2014 | 1719.103267 | 4303.290152 | 311.6456301 |
| DALYs (Disability-Adjusted Life Years) | High SDI | Both | 65+ years | diarrhea diseases | Unsafe water source | Rate | 2014 | 27.8449839 | 68.1757921 | 5.164433381 |
| DALYs (Disability-Adjusted Life Years) | High SDI | Both | 65+ years | diarrhea diseases | Unsafe water source | Percent | 2014 | 0.147090224 | 0.361404881 | 0.027358396 |
| DALYs (Disability-Adjusted Life Years) | High SDI | Both | 65+ years | diarrhea diseases | Unsafe water source | Number | 2014 | 28263.18755 | 69151.18143 | 5211.461487 |
| Deaths | Global | Both | 65+ years | diarrhea diseases | Unsafe water source | Rate | 2014 | 124.1804846 | 211.8699609 | 50.50292167 |
| Deaths | Global | Both | 65+ years | diarrhea diseases | Unsafe water source | Percent | 2014 | 1.377932437 | 1.761930745 | 0.742646715 |
| Deaths | Global | Both | 65+ years | diarrhea diseases | Unsafe water source | Number | 2014 | 442890.5079 | 750900.2692 | 180539.5148 |
| DALYs (Disability-Adjusted Life Years) | Global | Both | 65+ years | diarrhea diseases | Unsafe water source | Rate | 2014 | 2081.943226 | 3522.185017 | 854.4032579 |
| DALYs (Disability-Adjusted Life Years) | Global | Both | 65+ years | diarrhea diseases | Unsafe water source | Percent | 2014 | 1.369142929 | 1.754457349 | 0.7382089 |
| DALYs (Disability-Adjusted Life Years) | Global | Both | 65+ years | diarrhea diseases | Unsafe water source | Number | 2014 | 6981844.818 | 11706785.01 | 2877033.813 |
| Deaths | Middle SDI | Both | 65+ years | diarrhea diseases | Unsafe water source | Rate | 2013 | 62.2481415 | 106.9107285 | 18.63046811 |
| Deaths | Middle SDI | Both | 65+ years | diarrhea diseases | Unsafe water source | Percent | 2013 | 1.212552784 | 1.706753577 | 0.513220276 |
| Deaths | Middle SDI | Both | 65+ years | diarrhea diseases | Unsafe water source | Number | 2013 | 59632.17469 | 102281.7046 | 17827.6997 |
| DALYs (Disability-Adjusted Life Years) | Middle SDI | Both | 65+ years | diarrhea diseases | Unsafe water source | Rate | 2013 | 1040.646752 | 1748.002537 | 318.956547 |
| DALYs (Disability-Adjusted Life Years) | Middle SDI | Both | 65+ years | diarrhea diseases | Unsafe water source | Percent | 2013 | 1.210920445 | 1.70556515 | 0.512128557 |
| DALYs (Disability-Adjusted Life Years) | Middle SDI | Both | 65+ years | diarrhea diseases | Unsafe water source | Number | 2013 | 944209.9108 | 1585301.847 | 288792.5149 |
| Deaths | Low-middle SDI | Both | 65+ years | diarrhea diseases | Unsafe water source | Rate | 2013 | 505.6178523 | 873.8686448 | 209.1681111 |
| Deaths | Low-middle SDI | Both | 65+ years | diarrhea diseases | Unsafe water source | Percent | 2013 | 1.441790156 | 1.824846634 | 0.773670079 |
| Deaths | Low-middle SDI | Both | 65+ years | diarrhea diseases | Unsafe water source | Number | 2013 | 258811.2777 | 444930.6155 | 107419.2035 |
| DALYs (Disability-Adjusted Life Years) | Low-middle SDI | Both | 65+ years | diarrhea diseases | Unsafe water source | Rate | 2013 | 8261.16362 | 14222.15544 | 3405.425971 |
| DALYs (Disability-Adjusted Life Years) | Low-middle SDI | Both | 65+ years | diarrhea diseases | Unsafe water source | Percent | 2013 | 1.441311181 | 1.821216135 | 0.772151716 |
| DALYs (Disability-Adjusted Life Years) | Low-middle SDI | Both | 65+ years | diarrhea diseases | Unsafe water source | Number | 2013 | 4046467.425 | 6920265.984 | 1674048.242 |
| Deaths | Low SDI | Both | 65+ years | diarrhea diseases | Unsafe water source | Rate | 2013 | 800.5809158 | 1361.421399 | 353.3406023 |
| Deaths | Low SDI | Both | 65+ years | diarrhea diseases | Unsafe water source | Percent | 2013 | 1.517878135 | 1.854240147 | 0.874103086 |
| Deaths | Low SDI | Both | 65+ years | diarrhea diseases | Unsafe water source | Number | 2013 | 130537.6712 | 221563.1993 | 57737.20248 |
| DALYs (Disability-Adjusted Life Years) | Low SDI | Both | 65+ years | diarrhea diseases | Unsafe water source | Rate | 2013 | 13514.55968 | 22890.03959 | 5999.66689 |
| DALYs (Disability-Adjusted Life Years) | Low SDI | Both | 65+ years | diarrhea diseases | Unsafe water source | Percent | 2013 | 1.517811768 | 1.853882873 | 0.874623782 |
| DALYs (Disability-Adjusted Life Years) | Low SDI | Both | 65+ years | diarrhea diseases | Unsafe water source | Number | 2013 | 2120623.442 | 3585266.045 | 943875.0131 |
| Deaths | High-middle SDI | Both | 65+ years | diarrhea diseases | Unsafe water source | Rate | 2013 | 3.865245582 | 7.232647309 | 0.981974573 |
| Deaths | High-middle SDI | Both | 65+ years | diarrhea diseases | Unsafe water source | Percent | 2013 | 0.817921932 | 1.315579464 | 0.273816928 |
| Deaths | High-middle SDI | Both | 65+ years | diarrhea diseases | Unsafe water source | Number | 2013 | 3262.147129 | 6113.261283 | 826.6957396 |
| DALYs (Disability-Adjusted Life Years) | High-middle SDI | Both | 65+ years | diarrhea diseases | Unsafe water source | Rate | 2013 | 73.89559124 | 133.2902716 | 20.55094021 |
| DALYs (Disability-Adjusted Life Years) | High-middle SDI | Both | 65+ years | diarrhea diseases | Unsafe water source | Percent | 2013 | 0.786703138 | 1.268924928 | 0.266458199 |
| DALYs (Disability-Adjusted Life Years) | High-middle SDI | Both | 65+ years | diarrhea diseases | Unsafe water source | Number | 2013 | 56994.90539 | 103378.9294 | 15671.13523 |
| Deaths | High SDI | Both | 65+ years | diarrhea diseases | Unsafe water source | Rate | 2013 | 1.588305188 | 3.979004468 | 0.289492629 |
| Deaths | High SDI | Both | 65+ years | diarrhea diseases | Unsafe water source | Percent | 2013 | 0.138771408 | 0.348672334 | 0.0246898 |
| Deaths | High SDI | Both | 65+ years | diarrhea diseases | Unsafe water source | Number | 2013 | 1714.496007 | 4299.848506 | 312.5438018 |
| DALYs (Disability-Adjusted Life Years) | High SDI | Both | 65+ years | diarrhea diseases | Unsafe water source | Rate | 2013 | 28.34729733 | 69.37599738 | 5.260706563 |
| DALYs (Disability-Adjusted Life Years) | High SDI | Both | 65+ years | diarrhea diseases | Unsafe water source | Percent | 2013 | 0.144217373 | 0.354987 | 0.026456619 |
| DALYs (Disability-Adjusted Life Years) | High SDI | Both | 65+ years | diarrhea diseases | Unsafe water source | Number | 2013 | 28093.87124 | 68831.90241 | 5180.827441 |
| Deaths | Global | Both | 65+ years | diarrhea diseases | Unsafe water source | Rate | 2013 | 131.7033671 | 220.4676739 | 53.6235173 |
| Deaths | Global | Both | 65+ years | diarrhea diseases | Unsafe water source | Percent | 2013 | 1.383549046 | 1.76742505 | 0.747671897 |
| Deaths | Global | Both | 65+ years | diarrhea diseases | Unsafe water source | Number | 2013 | 454089.9937 | 753822.2878 | 185417.7408 |
| DALYs (Disability-Adjusted Life Years) | Global | Both | 65+ years | diarrhea diseases | Unsafe water source | Rate | 2013 | 2228.80856 | 3733.320214 | 912.2977843 |
| DALYs (Disability-Adjusted Life Years) | Global | Both | 65+ years | diarrhea diseases | Unsafe water source | Percent | 2013 | 1.375229148 | 1.761466173 | 0.743346825 |
| DALYs (Disability-Adjusted Life Years) | Global | Both | 65+ years | diarrhea diseases | Unsafe water source | Number | 2013 | 7198520.121 | 11941107.71 | 2958474.303 |
| Deaths | Middle SDI | Both | 65+ years | diarrhea diseases | Unsafe water source | Rate | 2012 | 65.25087251 | 109.1634792 | 20.31595182 |
| Deaths | Middle SDI | Both | 65+ years | diarrhea diseases | Unsafe water source | Percent | 2012 | 1.22296425 | 1.712132011 | 0.515588074 |
| Deaths | Middle SDI | Both | 65+ years | diarrhea diseases | Unsafe water source | Number | 2012 | 60270.77866 | 100752.9548 | 18781.69465 |
| DALYs (Disability-Adjusted Life Years) | Middle SDI | Both | 65+ years | diarrhea diseases | Unsafe water source | Rate | 2012 | 1097.02876 | 1814.545457 | 351.0972158 |
| DALYs (Disability-Adjusted Life Years) | Middle SDI | Both | 65+ years | diarrhea diseases | Unsafe water source | Percent | 2012 | 1.221269192 | 1.711385852 | 0.514982839 |
| DALYs (Disability-Adjusted Life Years) | Middle SDI | Both | 65+ years | diarrhea diseases | Unsafe water source | Number | 2012 | 959206.8347 | 1587490.688 | 306243.9308 |
| Deaths | Low-middle SDI | Both | 65+ years | diarrhea diseases | Unsafe water source | Rate | 2012 | 523.6307834 | 909.5722567 | 214.030404 |
| Deaths | Low-middle SDI | Both | 65+ years | diarrhea diseases | Unsafe water source | Percent | 2012 | 1.444730112 | 1.82692307 | 0.775446654 |
| Deaths | Low-middle SDI | Both | 65+ years | diarrhea diseases | Unsafe water source | Number | 2012 | 258713.554 | 447869.3197 | 106241.1278 |
| DALYs (Disability-Adjusted Life Years) | Low-middle SDI | Both | 65+ years | diarrhea diseases | Unsafe water source | Rate | 2012 | 8698.322257 | 15070.77852 | 3546.628556 |
| DALYs (Disability-Adjusted Life Years) | Low-middle SDI | Both | 65+ years | diarrhea diseases | Unsafe water source | Percent | 2012 | 1.444306866 | 1.825920429 | 0.774763294 |
| DALYs (Disability-Adjusted Life Years) | Low-middle SDI | Both | 65+ years | diarrhea diseases | Unsafe water source | Number | 2012 | 4111190.88 | 7095447.503 | 1685722.776 |
| Deaths | Low SDI | Both | 65+ years | diarrhea diseases | Unsafe water source | Rate | 2012 | 823.7712849 | 1374.230098 | 382.9678006 |
| Deaths | Low SDI | Both | 65+ years | diarrhea diseases | Unsafe water source | Percent | 2012 | 1.519565613 | 1.857220327 | 0.883032323 |
| Deaths | Low SDI | Both | 65+ years | diarrhea diseases | Unsafe water source | Number | 2012 | 130237.6795 | 216634.5188 | 60849.30259 |
| DALYs (Disability-Adjusted Life Years) | Low SDI | Both | 65+ years | diarrhea diseases | Unsafe water source | Rate | 2012 | 14074.76686 | 23483.59009 | 6529.126869 |
| DALYs (Disability-Adjusted Life Years) | Low SDI | Both | 65+ years | diarrhea diseases | Unsafe water source | Percent | 2012 | 1.519504049 | 1.856055488 | 0.883540059 |
| DALYs (Disability-Adjusted Life Years) | Low SDI | Both | 65+ years | diarrhea diseases | Unsafe water source | Number | 2012 | 2140940.747 | 3562805.147 | 999691.1335 |
| Deaths | High-middle SDI | Both | 65+ years | diarrhea diseases | Unsafe water source | Rate | 2012 | 3.861011363 | 7.171571776 | 1.004410172 |
| Deaths | High-middle SDI | Both | 65+ years | diarrhea diseases | Unsafe water source | Percent | 2012 | 0.837932967 | 1.336515521 | 0.285606318 |
| Deaths | High-middle SDI | Both | 65+ years | diarrhea diseases | Unsafe water source | Number | 2012 | 3185.669383 | 5917.084497 | 827.6707543 |
| DALYs (Disability-Adjusted Life Years) | High-middle SDI | Both | 65+ years | diarrhea diseases | Unsafe water source | Rate | 2012 | 74.84875443 | 133.8883069 | 20.86465734 |
| DALYs (Disability-Adjusted Life Years) | High-middle SDI | Both | 65+ years | diarrhea diseases | Unsafe water source | Percent | 2012 | 0.802331803 | 1.283390706 | 0.27703157 |
| DALYs (Disability-Adjusted Life Years) | High-middle SDI | Both | 65+ years | diarrhea diseases | Unsafe water source | Number | 2012 | 56174.24368 | 100793.6188 | 15501.21918 |
| Deaths | High SDI | Both | 65+ years | diarrhea diseases | Unsafe water source | Rate | 2012 | 1.615940297 | 4.066217622 | 0.291342074 |
| Deaths | High SDI | Both | 65+ years | diarrhea diseases | Unsafe water source | Percent | 2012 | 0.137128153 | 0.348198943 | 0.023875489 |
| Deaths | High SDI | Both | 65+ years | diarrhea diseases | Unsafe water source | Number | 2012 | 1700.331756 | 4282.660772 | 306.8441565 |
| DALYs (Disability-Adjusted Life Years) | High SDI | Both | 65+ years | diarrhea diseases | Unsafe water source | Rate | 2012 | 28.91791401 | 70.87198498 | 5.366597273 |
| DALYs (Disability-Adjusted Life Years) | High SDI | Both | 65+ years | diarrhea diseases | Unsafe water source | Percent | 2012 | 0.142258978 | 0.350228615 | 0.025834735 |
| DALYs (Disability-Adjusted Life Years) | High SDI | Both | 65+ years | diarrhea diseases | Unsafe water source | Number | 2012 | 27886.45865 | 68383.65662 | 5150.217932 |
| Deaths | Global | Both | 65+ years | diarrhea diseases | Unsafe water source | Rate | 2012 | 136.421577 | 229.122067 | 56.39983804 |
| Deaths | Global | Both | 65+ years | diarrhea diseases | Unsafe water source | Percent | 2012 | 1.388137864 | 1.770919552 | 0.753790148 |
| Deaths | Global | Both | 65+ years | diarrhea diseases | Unsafe water source | Number | 2012 | 454239.0578 | 759825.7554 | 189091.5918 |
| DALYs (Disability-Adjusted Life Years) | Global | Both | 65+ years | diarrhea diseases | Unsafe water source | Rate | 2012 | 2345.202239 | 3911.962891 | 966.842388 |
| DALYs (Disability-Adjusted Life Years) | Global | Both | 65+ years | diarrhea diseases | Unsafe water source | Percent | 2012 | 1.380461195 | 1.763092689 | 0.749441003 |
| DALYs (Disability-Adjusted Life Years) | Global | Both | 65+ years | diarrhea diseases | Unsafe water source | Number | 2012 | 7297512.448 | 12115337.91 | 3034321.429 |
| Deaths | Middle SDI | Both | 65+ years | diarrhea diseases | Unsafe water source | Rate | 2011 | 69.40219351 | 115.3635059 | 21.90426639 |
| Deaths | Middle SDI | Both | 65+ years | diarrhea diseases | Unsafe water source | Percent | 2011 | 1.231770368 | 1.717849036 | 0.515914824 |
| Deaths | Middle SDI | Both | 65+ years | diarrhea diseases | Unsafe water source | Number | 2011 | 61866.73927 | 102755.1474 | 19558.87198 |
| DALYs (Disability-Adjusted Life Years) | Middle SDI | Both | 65+ years | diarrhea diseases | Unsafe water source | Rate | 2011 | 1167.283848 | 1922.411883 | 377.7946929 |
| DALYs (Disability-Adjusted Life Years) | Middle SDI | Both | 65+ years | diarrhea diseases | Unsafe water source | Percent | 2011 | 1.23012811 | 1.716942638 | 0.515141412 |
| DALYs (Disability-Adjusted Life Years) | Middle SDI | Both | 65+ years | diarrhea diseases | Unsafe water source | Number | 2011 | 984680.9952 | 1620023.566 | 318592.8445 |
| Deaths | Low-middle SDI | Both | 65+ years | diarrhea diseases | Unsafe water source | Rate | 2011 | 555.640441 | 954.0131046 | 232.5963384 |
| Deaths | Low-middle SDI | Both | 65+ years | diarrhea diseases | Unsafe water source | Percent | 2011 | 1.447133852 | 1.826998323 | 0.782584283 |
| Deaths | Low-middle SDI | Both | 65+ years | diarrhea diseases | Unsafe water source | Number | 2011 | 265550.6043 | 454049.2875 | 111644.2267 |
| DALYs (Disability-Adjusted Life Years) | Low-middle SDI | Both | 65+ years | diarrhea diseases | Unsafe water source | Rate | 2011 | 9275.521836 | 15982.36884 | 3875.721916 |
| DALYs (Disability-Adjusted Life Years) | Low-middle SDI | Both | 65+ years | diarrhea diseases | Unsafe water source | Percent | 2011 | 1.446696257 | 1.827235941 | 0.781150359 |
| DALYs (Disability-Adjusted Life Years) | Low-middle SDI | Both | 65+ years | diarrhea diseases | Unsafe water source | Number | 2011 | 4241887.352 | 7279904.855 | 1781776.97 |
| Deaths | Low SDI | Both | 65+ years | diarrhea diseases | Unsafe water source | Rate | 2011 | 859.8771551 | 1427.926469 | 399.6794134 |
| Deaths | Low SDI | Both | 65+ years | diarrhea diseases | Unsafe water source | Percent | 2011 | 1.52107515 | 1.855835714 | 0.884484517 |
| Deaths | Low SDI | Both | 65+ years | diarrhea diseases | Unsafe water source | Number | 2011 | 131815.4114 | 218420.538 | 61431.6757 |
| DALYs (Disability-Adjusted Life Years) | Low SDI | Both | 65+ years | diarrhea diseases | Unsafe water source | Rate | 2011 | 14769.89138 | 24439.17187 | 6861.29528 |
| DALYs (Disability-Adjusted Life Years) | Low SDI | Both | 65+ years | diarrhea diseases | Unsafe water source | Percent | 2011 | 1.52107631 | 1.855710747 | 0.884278083 |
| DALYs (Disability-Adjusted Life Years) | Low SDI | Both | 65+ years | diarrhea diseases | Unsafe water source | Number | 2011 | 2179181.696 | 3595795.564 | 1015928.965 |
| Deaths | High-middle SDI | Both | 65+ years | diarrhea diseases | Unsafe water source | Rate | 2011 | 3.889064449 | 7.129652459 | 1.021068902 |
| Deaths | High-middle SDI | Both | 65+ years | diarrhea diseases | Unsafe water source | Percent | 2011 | 0.860009902 | 1.358830133 | 0.299025471 |
| Deaths | High-middle SDI | Both | 65+ years | diarrhea diseases | Unsafe water source | Number | 2011 | 3128.801585 | 5744.736183 | 821.0587525 |
| DALYs (Disability-Adjusted Life Years) | High-middle SDI | Both | 65+ years | diarrhea diseases | Unsafe water source | Rate | 2011 | 76.14152154 | 135.3386444 | 21.71496678 |
| DALYs (Disability-Adjusted Life Years) | High-middle SDI | Both | 65+ years | diarrhea diseases | Unsafe water source | Percent | 2011 | 0.81956536 | 1.302751171 | 0.287958111 |
| DALYs (Disability-Adjusted Life Years) | High-middle SDI | Both | 65+ years | diarrhea diseases | Unsafe water source | Number | 2011 | 55493.5282 | 99286.0396 | 15669.51143 |
| Deaths | High SDI | Both | 65+ years | diarrhea diseases | Unsafe water source | Rate | 2011 | 1.613293234 | 4.085649163 | 0.285544515 |
| Deaths | High SDI | Both | 65+ years | diarrhea diseases | Unsafe water source | Percent | 2011 | 0.135571141 | 0.345534298 | 0.023231789 |
| Deaths | High SDI | Both | 65+ years | diarrhea diseases | Unsafe water source | Number | 2011 | 1655.435229 | 4197.735585 | 293.2589408 |
| DALYs (Disability-Adjusted Life Years) | High SDI | Both | 65+ years | diarrhea diseases | Unsafe water source | Rate | 2011 | 29.15031104 | 71.40210739 | 5.389277307 |
| DALYs (Disability-Adjusted Life Years) | High SDI | Both | 65+ years | diarrhea diseases | Unsafe water source | Percent | 2011 | 0.140537468 | 0.346957955 | 0.025373124 |
| DALYs (Disability-Adjusted Life Years) | High SDI | Both | 65+ years | diarrhea diseases | Unsafe water source | Number | 2011 | 27329.33133 | 66965.13727 | 5032.679862 |
| Deaths | Global | Both | 65+ years | diarrhea diseases | Unsafe water source | Rate | 2011 | 143.9822235 | 240.6230715 | 60.5866791 |
| Deaths | Global | Both | 65+ years | diarrhea diseases | Unsafe water source | Percent | 2011 | 1.392994278 | 1.77297802 | 0.75624199 |
| Deaths | Global | Both | 65+ years | diarrhea diseases | Unsafe water source | Number | 2011 | 464153.5204 | 768093.1693 | 196376.0688 |
| DALYs (Disability-Adjusted Life Years) | Global | Both | 65+ years | diarrhea diseases | Unsafe water source | Rate | 2011 | 2491.490174 | 4174.857663 | 1046.312453 |
| DALYs (Disability-Adjusted Life Years) | Global | Both | 65+ years | diarrhea diseases | Unsafe water source | Percent | 2011 | 1.385634055 | 1.767093693 | 0.751916678 |
| DALYs (Disability-Adjusted Life Years) | Global | Both | 65+ years | diarrhea diseases | Unsafe water source | Number | 2011 | 7490779.564 | 12417136.47 | 3166294.316 |
| Deaths | Middle SDI | Both | 65+ years | diarrhea diseases | Unsafe water source | Rate | 2010 | 73.97805361 | 121.2163255 | 23.28224855 |
| Deaths | Middle SDI | Both | 65+ years | diarrhea diseases | Unsafe water source | Percent | 2010 | 1.242288216 | 1.723024177 | 0.524148438 |
| Deaths | Middle SDI | Both | 65+ years | diarrhea diseases | Unsafe water source | Number | 2010 | 63651.18266 | 104028.9124 | 20048.19269 |
| DALYs (Disability-Adjusted Life Years) | Middle SDI | Both | 65+ years | diarrhea diseases | Unsafe water source | Rate | 2010 | 1244.855986 | 2016.556211 | 403.1151097 |
| DALYs (Disability-Adjusted Life Years) | Middle SDI | Both | 65+ years | diarrhea diseases | Unsafe water source | Percent | 2010 | 1.240709153 | 1.7219463 | 0.523722717 |
| DALYs (Disability-Adjusted Life Years) | Middle SDI | Both | 65+ years | diarrhea diseases | Unsafe water source | Number | 2010 | 1013539.871 | 1636125.012 | 327024.3862 |
| Deaths | Low-middle SDI | Both | 65+ years | diarrhea diseases | Unsafe water source | Rate | 2010 | 588.5621097 | 1014.928314 | 242.8544442 |
| Deaths | Low-middle SDI | Both | 65+ years | diarrhea diseases | Unsafe water source | Percent | 2010 | 1.450392006 | 1.827294321 | 0.778584389 |
| Deaths | Low-middle SDI | Both | 65+ years | diarrhea diseases | Unsafe water source | Number | 2010 | 272431.6022 | 467406.7494 | 112756.1103 |
| DALYs (Disability-Adjusted Life Years) | Low-middle SDI | Both | 65+ years | diarrhea diseases | Unsafe water source | Rate | 2010 | 9834.581776 | 17070.14141 | 4062.80223 |
| DALYs (Disability-Adjusted Life Years) | Low-middle SDI | Both | 65+ years | diarrhea diseases | Unsafe water source | Percent | 2010 | 1.449976256 | 1.827115202 | 0.777106885 |
| DALYs (Disability-Adjusted Life Years) | Low-middle SDI | Both | 65+ years | diarrhea diseases | Unsafe water source | Number | 2010 | 4359053.917 | 7523073.373 | 1806923.171 |
| Deaths | Low SDI | Both | 65+ years | diarrhea diseases | Unsafe water source | Rate | 2010 | 896.379197 | 1477.241503 | 418.7843397 |
| Deaths | Low SDI | Both | 65+ years | diarrhea diseases | Unsafe water source | Percent | 2010 | 1.522634431 | 1.857614727 | 0.890271831 |
| Deaths | Low SDI | Both | 65+ years | diarrhea diseases | Unsafe water source | Number | 2010 | 133441.5613 | 219143.193 | 62598.29035 |
| DALYs (Disability-Adjusted Life Years) | Low SDI | Both | 65+ years | diarrhea diseases | Unsafe water source | Rate | 2010 | 15432.34597 | 25422.25709 | 7191.888892 |
| DALYs (Disability-Adjusted Life Years) | Low SDI | Both | 65+ years | diarrhea diseases | Unsafe water source | Percent | 2010 | 1.522653128 | 1.856612228 | 0.890299217 |
| DALYs (Disability-Adjusted Life Years) | Low SDI | Both | 65+ years | diarrhea diseases | Unsafe water source | Number | 2010 | 2211323.655 | 3626503.586 | 1035980.364 |
| Deaths | High-middle SDI | Both | 65+ years | diarrhea diseases | Unsafe water source | Rate | 2010 | 3.903586794 | 7.193153056 | 1.037043182 |
| Deaths | High-middle SDI | Both | 65+ years | diarrhea diseases | Unsafe water source | Percent | 2010 | 0.876945816 | 1.377069257 | 0.310200166 |
| Deaths | High-middle SDI | Both | 65+ years | diarrhea diseases | Unsafe water source | Number | 2010 | 3077.751385 | 5692.266094 | 816.7125683 |
| DALYs (Disability-Adjusted Life Years) | High-middle SDI | Both | 65+ years | diarrhea diseases | Unsafe water source | Rate | 2010 | 76.57901286 | 133.1469804 | 22.12337939 |
| DALYs (Disability-Adjusted Life Years) | High-middle SDI | Both | 65+ years | diarrhea diseases | Unsafe water source | Percent | 2010 | 0.832870105 | 1.314375086 | 0.294584257 |
| DALYs (Disability-Adjusted Life Years) | High-middle SDI | Both | 65+ years | diarrhea diseases | Unsafe water source | Number | 2010 | 54773.98186 | 95793.61288 | 15647.13057 |
| Deaths | High SDI | Both | 65+ years | diarrhea diseases | Unsafe water source | Rate | 2010 | 1.661087005 | 4.208446254 | 0.29428495 |
| Deaths | High SDI | Both | 65+ years | diarrhea diseases | Unsafe water source | Percent | 2010 | 0.134674976 | 0.342425388 | 0.023222195 |
| Deaths | High SDI | Both | 65+ years | diarrhea diseases | Unsafe water source | Number | 2010 | 1672.634931 | 4239.644867 | 296.3783484 |
| DALYs (Disability-Adjusted Life Years) | High SDI | Both | 65+ years | diarrhea diseases | Unsafe water source | Rate | 2010 | 29.86828309 | 73.40332689 | 5.533761443 |
| DALYs (Disability-Adjusted Life Years) | High SDI | Both | 65+ years | diarrhea diseases | Unsafe water source | Percent | 2010 | 0.140174726 | 0.345342824 | 0.02559051 |
| DALYs (Disability-Adjusted Life Years) | High SDI | Both | 65+ years | diarrhea diseases | Unsafe water source | Number | 2010 | 27520.26827 | 67641.60047 | 5070.987852 |
| Deaths | Global | Both | 65+ years | diarrhea diseases | Unsafe water source | Rate | 2010 | 151.1714526 | 253.2930846 | 62.86366547 |
| Deaths | Global | Both | 65+ years | diarrhea diseases | Unsafe water source | Percent | 2010 | 1.397258005 | 1.778366792 | 0.75999786 |
| Deaths | Global | Both | 65+ years | diarrhea diseases | Unsafe water source | Number | 2010 | 474406.7635 | 788023.7475 | 198031.1414 |
| DALYs (Disability-Adjusted Life Years) | Global | Both | 65+ years | diarrhea diseases | Unsafe water source | Rate | 2010 | 2618.496351 | 4407.99318 | 1088.731055 |
| DALYs (Disability-Adjusted Life Years) | Global | Both | 65+ years | diarrhea diseases | Unsafe water source | Percent | 2010 | 1.390352317 | 1.769503493 | 0.755722687 |
| DALYs (Disability-Adjusted Life Years) | Global | Both | 65+ years | diarrhea diseases | Unsafe water source | Number | 2010 | 7668346.328 | 12780265.65 | 3202464.292 |
| Deaths | Middle SDI | Both | 65+ years | diarrhea diseases | Unsafe water source | Rate | 2009 | 78.0927722 | 128.4148535 | 25.26769972 |
| Deaths | Middle SDI | Both | 65+ years | diarrhea diseases | Unsafe water source | Percent | 2009 | 1.247426663 | 1.723723562 | 0.518489524 |
| Deaths | Middle SDI | Both | 65+ years | diarrhea diseases | Unsafe water source | Number | 2009 | 64987.67487 | 106631.3359 | 21056.91657 |
| DALYs (Disability-Adjusted Life Years) | Middle SDI | Both | 65+ years | diarrhea diseases | Unsafe water source | Rate | 2009 | 1312.866186 | 2131.5255 | 437.1675274 |
| DALYs (Disability-Adjusted Life Years) | Middle SDI | Both | 65+ years | diarrhea diseases | Unsafe water source | Percent | 2009 | 1.245849867 | 1.720882909 | 0.518132315 |
| DALYs (Disability-Adjusted Life Years) | Middle SDI | Both | 65+ years | diarrhea diseases | Unsafe water source | Number | 2009 | 1035254.722 | 1678197.659 | 344233.645 |
| Deaths | Low-middle SDI | Both | 65+ years | diarrhea diseases | Unsafe water source | Rate | 2009 | 618.2899279 | 1071.810778 | 258.0019164 |
| Deaths | Low-middle SDI | Both | 65+ years | diarrhea diseases | Unsafe water source | Percent | 2009 | 1.451196112 | 1.827708657 | 0.774718161 |
| Deaths | Low-middle SDI | Both | 65+ years | diarrhea diseases | Unsafe water source | Number | 2009 | 277430.0716 | 478616.36 | 116160.1637 |
| DALYs (Disability-Adjusted Life Years) | Low-middle SDI | Both | 65+ years | diarrhea diseases | Unsafe water source | Rate | 2009 | 10328.8158 | 18013.7546 | 4322.863106 |
| DALYs (Disability-Adjusted Life Years) | Low-middle SDI | Both | 65+ years | diarrhea diseases | Unsafe water source | Percent | 2009 | 1.450814383 | 1.826514758 | 0.774478906 |
| DALYs (Disability-Adjusted Life Years) | Low-middle SDI | Both | 65+ years | diarrhea diseases | Unsafe water source | Number | 2009 | 4443692.569 | 7711980.09 | 1866096.112 |
| Deaths | Low SDI | Both | 65+ years | diarrhea diseases | Unsafe water source | Rate | 2009 | 927.4707459 | 1522.428746 | 436.7155996 |
| Deaths | Low SDI | Both | 65+ years | diarrhea diseases | Unsafe water source | Percent | 2009 | 1.523875138 | 1.856086251 | 0.891144958 |
| Deaths | Low SDI | Both | 65+ years | diarrhea diseases | Unsafe water source | Number | 2009 | 134116.4357 | 219766.4595 | 63438.14017 |
| DALYs (Disability-Adjusted Life Years) | Low SDI | Both | 65+ years | diarrhea diseases | Unsafe water source | Rate | 2009 | 15956.38589 | 26158.03508 | 7495.330666 |
| DALYs (Disability-Adjusted Life Years) | Low SDI | Both | 65+ years | diarrhea diseases | Unsafe water source | Percent | 2009 | 1.523906845 | 1.856058988 | 0.890995495 |
| DALYs (Disability-Adjusted Life Years) | Low SDI | Both | 65+ years | diarrhea diseases | Unsafe water source | Number | 2009 | 2222741.563 | 3636253.492 | 1050293.151 |
| Deaths | High-middle SDI | Both | 65+ years | diarrhea diseases | Unsafe water source | Rate | 2009 | 3.943524365 | 7.101446491 | 1.061908778 |
| Deaths | High-middle SDI | Both | 65+ years | diarrhea diseases | Unsafe water source | Percent | 2009 | 0.903565447 | 1.408180376 | 0.326047189 |
| Deaths | High-middle SDI | Both | 65+ years | diarrhea diseases | Unsafe water source | Number | 2009 | 3055.856409 | 5507.308492 | 823.7887676 |
| DALYs (Disability-Adjusted Life Years) | High-middle SDI | Both | 65+ years | diarrhea diseases | Unsafe water source | Rate | 2009 | 77.25080147 | 134.8344238 | 22.13143359 |
| DALYs (Disability-Adjusted Life Years) | High-middle SDI | Both | 65+ years | diarrhea diseases | Unsafe water source | Percent | 2009 | 0.854259132 | 1.34386692 | 0.308424857 |
| DALYs (Disability-Adjusted Life Years) | High-middle SDI | Both | 65+ years | diarrhea diseases | Unsafe water source | Number | 2009 | 54548.21234 | 95759.08707 | 15524.23333 |
| Deaths | High SDI | Both | 65+ years | diarrhea diseases | Unsafe water source | Rate | 2009 | 1.72465881 | 4.351869089 | 0.309442594 |
| Deaths | High SDI | Both | 65+ years | diarrhea diseases | Unsafe water source | Percent | 2009 | 0.137361538 | 0.345274857 | 0.024066184 |
| Deaths | High SDI | Both | 65+ years | diarrhea diseases | Unsafe water source | Number | 2009 | 1701.759912 | 4295.746439 | 305.2959058 |
| DALYs (Disability-Adjusted Life Years) | High SDI | Both | 65+ years | diarrhea diseases | Unsafe water source | Rate | 2009 | 30.74964824 | 75.87829959 | 5.777356186 |
| DALYs (Disability-Adjusted Life Years) | High SDI | Both | 65+ years | diarrhea diseases | Unsafe water source | Percent | 2009 | 0.142323333 | 0.344853709 | 0.026298946 |
| DALYs (Disability-Adjusted Life Years) | High SDI | Both | 65+ years | diarrhea diseases | Unsafe water source | Number | 2009 | 27801.39541 | 68726.3748 | 5189.943165 |
| Deaths | Global | Both | 65+ years | diarrhea diseases | Unsafe water source | Rate | 2009 | 157.1077793 | 263.8266467 | 66.58026495 |
| Deaths | Global | Both | 65+ years | diarrhea diseases | Unsafe water source | Percent | 2009 | 1.399696124 | 1.781134912 | 0.756262158 |
| Deaths | Global | Both | 65+ years | diarrhea diseases | Unsafe water source | Number | 2009 | 481422.9842 | 801992.2036 | 204972.0337 |
| DALYs (Disability-Adjusted Life Years) | Global | Both | 65+ years | diarrhea diseases | Unsafe water source | Rate | 2009 | 2717.201369 | 4568.614141 | 1150.460771 |
| DALYs (Disability-Adjusted Life Years) | Global | Both | 65+ years | diarrhea diseases | Unsafe water source | Percent | 2009 | 1.393032277 | 1.772644828 | 0.752333254 |
| DALYs (Disability-Adjusted Life Years) | Global | Both | 65+ years | diarrhea diseases | Unsafe water source | Number | 2009 | 7786158.86 | 12974885.26 | 3315569.553 |
| Deaths | Middle SDI | Both | 65+ years | diarrhea diseases | Unsafe water source | Rate | 2008 | 82.68247365 | 135.5168889 | 26.90741694 |
| Deaths | Middle SDI | Both | 65+ years | diarrhea diseases | Unsafe water source | Percent | 2008 | 1.252110205 | 1.725390478 | 0.517371511 |
| Deaths | Middle SDI | Both | 65+ years | diarrhea diseases | Unsafe water source | Number | 2008 | 66560.85049 | 108794.5267 | 21691.45757 |
| DALYs (Disability-Adjusted Life Years) | Middle SDI | Both | 65+ years | diarrhea diseases | Unsafe water source | Rate | 2008 | 1387.510825 | 2249.584061 | 454.4790677 |
| DALYs (Disability-Adjusted Life Years) | Middle SDI | Both | 65+ years | diarrhea diseases | Unsafe water source | Percent | 2008 | 1.250467485 | 1.723684977 | 0.516689804 |
| DALYs (Disability-Adjusted Life Years) | Middle SDI | Both | 65+ years | diarrhea diseases | Unsafe water source | Number | 2008 | 1059873.539 | 1714234.637 | 346566.6327 |
| Deaths | Low-middle SDI | Both | 65+ years | diarrhea diseases | Unsafe water source | Rate | 2008 | 657.7637673 | 1134.628812 | 273.3055929 |
| Deaths | Low-middle SDI | Both | 65+ years | diarrhea diseases | Unsafe water source | Percent | 2008 | 1.452241001 | 1.825964948 | 0.782396839 |
| Deaths | Low-middle SDI | Both | 65+ years | diarrhea diseases | Unsafe water source | Number | 2008 | 286435.7476 | 491982.9973 | 119533.3359 |
| DALYs (Disability-Adjusted Life Years) | Low-middle SDI | Both | 65+ years | diarrhea diseases | Unsafe water source | Rate | 2008 | 10943.80841 | 19021.20937 | 4546.374268 |
| DALYs (Disability-Adjusted Life Years) | Low-middle SDI | Both | 65+ years | diarrhea diseases | Unsafe water source | Percent | 2008 | 1.451821754 | 1.825817483 | 0.781580305 |
| DALYs (Disability-Adjusted Life Years) | Low-middle SDI | Both | 65+ years | diarrhea diseases | Unsafe water source | Number | 2008 | 4574249.585 | 7915854.819 | 1909574.971 |
| Deaths | Low SDI | Both | 65+ years | diarrhea diseases | Unsafe water source | Rate | 2008 | 959.9282997 | 1589.56383 | 449.1179573 |
| Deaths | Low SDI | Both | 65+ years | diarrhea diseases | Unsafe water source | Percent | 2008 | 1.524694994 | 1.854123889 | 0.894548085 |
| Deaths | Low SDI | Both | 65+ years | diarrhea diseases | Unsafe water source | Number | 2008 | 134820.3778 | 222752.4277 | 63350.68231 |
| DALYs (Disability-Adjusted Life Years) | Low SDI | Both | 65+ years | diarrhea diseases | Unsafe water source | Rate | 2008 | 16458.98723 | 27195.98921 | 7682.562656 |
| DALYs (Disability-Adjusted Life Years) | Low SDI | Both | 65+ years | diarrhea diseases | Unsafe water source | Percent | 2008 | 1.52478063 | 1.853733235 | 0.894636908 |
| DALYs (Disability-Adjusted Life Years) | Low SDI | Both | 65+ years | diarrhea diseases | Unsafe water source | Number | 2008 | 2228298.035 | 3671237.005 | 1046505.376 |
| Deaths | High-middle SDI | Both | 65+ years | diarrhea diseases | Unsafe water source | Rate | 2008 | 4.044015263 | 7.329679713 | 1.103342775 |
| Deaths | High-middle SDI | Both | 65+ years | diarrhea diseases | Unsafe water source | Percent | 2008 | 0.920300406 | 1.427376467 | 0.332028701 |
| Deaths | High-middle SDI | Both | 65+ years | diarrhea diseases | Unsafe water source | Number | 2008 | 3078.993523 | 5584.742215 | 839.1636801 |
| DALYs (Disability-Adjusted Life Years) | High-middle SDI | Both | 65+ years | diarrhea diseases | Unsafe water source | Rate | 2008 | 78.70858322 | 138.051166 | 22.90110642 |
| DALYs (Disability-Adjusted Life Years) | High-middle SDI | Both | 65+ years | diarrhea diseases | Unsafe water source | Percent | 2008 | 0.870558018 | 1.358369549 | 0.314791619 |
| DALYs (Disability-Adjusted Life Years) | High-middle SDI | Both | 65+ years | diarrhea diseases | Unsafe water source | Number | 2008 | 54955.56239 | 96907.61708 | 15900.58732 |
| Deaths | High SDI | Both | 65+ years | diarrhea diseases | Unsafe water source | Rate | 2008 | 1.780781634 | 4.504188977 | 0.323737525 |
| Deaths | High SDI | Both | 65+ years | diarrhea diseases | Unsafe water source | Percent | 2008 | 0.141171213 | 0.350917166 | 0.025549872 |
| Deaths | High SDI | Both | 65+ years | diarrhea diseases | Unsafe water source | Number | 2008 | 1720.963645 | 4356.810025 | 312.4403642 |
| DALYs (Disability-Adjusted Life Years) | High SDI | Both | 65+ years | diarrhea diseases | Unsafe water source | Rate | 2008 | 31.55365927 | 77.62192562 | 6.061100367 |
| DALYs (Disability-Adjusted Life Years) | High SDI | Both | 65+ years | diarrhea diseases | Unsafe water source | Percent | 2008 | 0.145395175 | 0.351044174 | 0.027647222 |
| DALYs (Disability-Adjusted Life Years) | High SDI | Both | 65+ years | diarrhea diseases | Unsafe water source | Number | 2008 | 27945.763 | 68830.43386 | 5330.243836 |
| Deaths | Global | Both | 65+ years | diarrhea diseases | Unsafe water source | Rate | 2008 | 164.6118492 | 274.7920097 | 68.75822473 |
| Deaths | Global | Both | 65+ years | diarrhea diseases | Unsafe water source | Percent | 2008 | 1.402386179 | 1.781425754 | 0.764455577 |
| Deaths | Global | Both | 65+ years | diarrhea diseases | Unsafe water source | Number | 2008 | 492748.7135 | 817191.9576 | 206482.7758 |
| DALYs (Disability-Adjusted Life Years) | Global | Both | 65+ years | diarrhea diseases | Unsafe water source | Rate | 2008 | 2833.967687 | 4741.828023 | 1182.530531 |
| DALYs (Disability-Adjusted Life Years) | Global | Both | 65+ years | diarrhea diseases | Unsafe water source | Percent | 2008 | 1.395896343 | 1.775387352 | 0.760298323 |
| DALYs (Disability-Adjusted Life Years) | Global | Both | 65+ years | diarrhea diseases | Unsafe water source | Number | 2008 | 7947454.724 | 13197192.32 | 3331616.398 |
| Deaths | Middle SDI | Both | 65+ years | diarrhea diseases | Unsafe water source | Rate | 2007 | 86.93674045 | 143.2271332 | 27.99068232 |
| Deaths | Middle SDI | Both | 65+ years | diarrhea diseases | Unsafe water source | Percent | 2007 | 1.257938829 | 1.728595215 | 0.514985779 |
| Deaths | Middle SDI | Both | 65+ years | diarrhea diseases | Unsafe water source | Number | 2007 | 67548.50729 | 111065.8913 | 21774.9295 |
| DALYs (Disability-Adjusted Life Years) | Middle SDI | Both | 65+ years | diarrhea diseases | Unsafe water source | Rate | 2007 | 1458.534789 | 2378.808536 | 479.9349349 |
| DALYs (Disability-Adjusted Life Years) | Middle SDI | Both | 65+ years | diarrhea diseases | Unsafe water source | Percent | 2007 | 1.256156252 | 1.725916264 | 0.514078246 |
| DALYs (Disability-Adjusted Life Years) | Middle SDI | Both | 65+ years | diarrhea diseases | Unsafe water source | Number | 2007 | 1076568.806 | 1751503.203 | 353598.6566 |
| Deaths | Low-middle SDI | Both | 65+ years | diarrhea diseases | Unsafe water source | Rate | 2007 | 685.0114386 | 1186.936207 | 287.1230669 |
| Deaths | Low-middle SDI | Both | 65+ years | diarrhea diseases | Unsafe water source | Percent | 2007 | 1.453476526 | 1.830012941 | 0.779578856 |
| Deaths | Low-middle SDI | Both | 65+ years | diarrhea diseases | Unsafe water source | Number | 2007 | 289193.5067 | 498783.6656 | 121711.3058 |
| DALYs (Disability-Adjusted Life Years) | Low-middle SDI | Both | 65+ years | diarrhea diseases | Unsafe water source | Rate | 2007 | 11382.25165 | 19683.64526 | 4763.681378 |
| DALYs (Disability-Adjusted Life Years) | Low-middle SDI | Both | 65+ years | diarrhea diseases | Unsafe water source | Percent | 2007 | 1.453035379 | 1.829980705 | 0.77880442 |
| DALYs (Disability-Adjusted Life Years) | Low-middle SDI | Both | 65+ years | diarrhea diseases | Unsafe water source | Number | 2007 | 4616042.634 | 7937360.89 | 1940821.663 |
| Deaths | Low SDI | Both | 65+ years | diarrhea diseases | Unsafe water source | Rate | 2007 | 990.1973232 | 1595.67512 | 453.9051637 |
| Deaths | Low SDI | Both | 65+ years | diarrhea diseases | Unsafe water source | Percent | 2007 | 1.525667214 | 1.854341391 | 0.895171552 |
| Deaths | Low SDI | Both | 65+ years | diarrhea diseases | Unsafe water source | Number | 2007 | 134941.1344 | 216699.1511 | 61997.83222 |
| DALYs (Disability-Adjusted Life Years) | Low SDI | Both | 65+ years | diarrhea diseases | Unsafe water source | Rate | 2007 | 16980.43975 | 27318.22379 | 7785.235492 |
| DALYs (Disability-Adjusted Life Years) | Low SDI | Both | 65+ years | diarrhea diseases | Unsafe water source | Percent | 2007 | 1.525767037 | 1.854709602 | 0.895271712 |
| DALYs (Disability-Adjusted Life Years) | Low SDI | Both | 65+ years | diarrhea diseases | Unsafe water source | Number | 2007 | 2231942.79 | 3575023.156 | 1026962.669 |
| Deaths | High-middle SDI | Both | 65+ years | diarrhea diseases | Unsafe water source | Rate | 2007 | 4.153991655 | 7.471496399 | 1.131511335 |
| Deaths | High-middle SDI | Both | 65+ years | diarrhea diseases | Unsafe water source | Percent | 2007 | 0.938109876 | 1.443036852 | 0.338010142 |
| Deaths | High-middle SDI | Both | 65+ years | diarrhea diseases | Unsafe water source | Number | 2007 | 3102.788137 | 5578.495025 | 847.0215847 |
| DALYs (Disability-Adjusted Life Years) | High-middle SDI | Both | 65+ years | diarrhea diseases | Unsafe water source | Rate | 2007 | 80.24833241 | 140.544426 | 23.22979958 |
| DALYs (Disability-Adjusted Life Years) | High-middle SDI | Both | 65+ years | diarrhea diseases | Unsafe water source | Percent | 2007 | 0.887264283 | 1.378102889 | 0.319912978 |
| DALYs (Disability-Adjusted Life Years) | High-middle SDI | Both | 65+ years | diarrhea diseases | Unsafe water source | Number | 2007 | 55313.9351 | 97147.07098 | 15970.22884 |
| Deaths | High SDI | Both | 65+ years | diarrhea diseases | Unsafe water source | Rate | 2007 | 1.798476569 | 4.538234052 | 0.325400615 |
| Deaths | High SDI | Both | 65+ years | diarrhea diseases | Unsafe water source | Percent | 2007 | 0.146325665 | 0.359523351 | 0.027445196 |
| Deaths | High SDI | Both | 65+ years | diarrhea diseases | Unsafe water source | Number | 2007 | 1700.757338 | 4299.433619 | 306.7734923 |
| DALYs (Disability-Adjusted Life Years) | High SDI | Both | 65+ years | diarrhea diseases | Unsafe water source | Rate | 2007 | 31.819274 | 77.39275747 | 6.16124762 |
| DALYs (Disability-Adjusted Life Years) | High SDI | Both | 65+ years | diarrhea diseases | Unsafe water source | Percent | 2007 | 0.149603006 | 0.357903173 | 0.029085228 |
| DALYs (Disability-Adjusted Life Years) | High SDI | Both | 65+ years | diarrhea diseases | Unsafe water source | Number | 2007 | 27571.14197 | 67185.94372 | 5307.727724 |
| Deaths | Global | Both | 65+ years | diarrhea diseases | Unsafe water source | Rate | 2007 | 170.293333 | 281.623717 | 72.12246011 |
| Deaths | Global | Both | 65+ years | diarrhea diseases | Unsafe water source | Percent | 2007 | 1.406020737 | 1.781472414 | 0.758055804 |
| Deaths | Global | Both | 65+ years | diarrhea diseases | Unsafe water source | Number | 2007 | 496619.352 | 814571.2796 | 211199.9206 |
| DALYs (Disability-Adjusted Life Years) | Global | Both | 65+ years | diarrhea diseases | Unsafe water source | Rate | 2007 | 2927.015464 | 4864.868196 | 1237.460891 |
| DALYs (Disability-Adjusted Life Years) | Global | Both | 65+ years | diarrhea diseases | Unsafe water source | Percent | 2007 | 1.399657235 | 1.776592311 | 0.754253815 |
| DALYs (Disability-Adjusted Life Years) | Global | Both | 65+ years | diarrhea diseases | Unsafe water source | Number | 2007 | 8009587.802 | 13188615.1 | 3406318.081 |
| Deaths | Middle SDI | Both | 65+ years | diarrhea diseases | Unsafe water source | Rate | 2006 | 91.69700326 | 148.0017201 | 30.54130221 |
| Deaths | Middle SDI | Both | 65+ years | diarrhea diseases | Unsafe water source | Percent | 2006 | 1.26563266 | 1.733586894 | 0.517792061 |
| Deaths | Middle SDI | Both | 65+ years | diarrhea diseases | Unsafe water source | Number | 2006 | 68660.0044 | 110361.0109 | 22917.29746 |
| DALYs (Disability-Adjusted Life Years) | Middle SDI | Both | 65+ years | diarrhea diseases | Unsafe water source | Rate | 2006 | 1539.01819 | 2475.263639 | 524.4043499 |
| DALYs (Disability-Adjusted Life Years) | Middle SDI | Both | 65+ years | diarrhea diseases | Unsafe water source | Percent | 2006 | 1.263717223 | 1.731527427 | 0.513714397 |
| DALYs (Disability-Adjusted Life Years) | Middle SDI | Both | 65+ years | diarrhea diseases | Unsafe water source | Number | 2006 | 1096388.879 | 1756362.988 | 374416.7171 |
| Deaths | Low-middle SDI | Both | 65+ years | diarrhea diseases | Unsafe water source | Rate | 2006 | 701.2900003 | 1192.874733 | 294.7433225 |
| Deaths | Low-middle SDI | Both | 65+ years | diarrhea diseases | Unsafe water source | Percent | 2006 | 1.454753209 | 1.834068609 | 0.775964605 |
| Deaths | Low-middle SDI | Both | 65+ years | diarrhea diseases | Unsafe water source | Number | 2006 | 286284.7923 | 484424.2767 | 120663.5853 |
| DALYs (Disability-Adjusted Life Years) | Low-middle SDI | Both | 65+ years | diarrhea diseases | Unsafe water source | Rate | 2006 | 11685.60803 | 20024.3922 | 4910.93015 |
| DALYs (Disability-Adjusted Life Years) | Low-middle SDI | Both | 65+ years | diarrhea diseases | Unsafe water source | Percent | 2006 | 1.454356449 | 1.83321456 | 0.775452506 |
| DALYs (Disability-Adjusted Life Years) | Low-middle SDI | Both | 65+ years | diarrhea diseases | Unsafe water source | Number | 2006 | 4584703.694 | 7813030.269 | 1932578.372 |
| Deaths | Low SDI | Both | 65+ years | diarrhea diseases | Unsafe water source | Rate | 2006 | 1013.786839 | 1632.74519 | 471.7223103 |
| Deaths | Low SDI | Both | 65+ years | diarrhea diseases | Unsafe water source | Percent | 2006 | 1.52687273 | 1.860436944 | 0.905259871 |
| Deaths | Low SDI | Both | 65+ years | diarrhea diseases | Unsafe water source | Number | 2006 | 133905.7394 | 215193.2879 | 62447.63039 |
| DALYs (Disability-Adjusted Life Years) | Low SDI | Both | 65+ years | diarrhea diseases | Unsafe water source | Rate | 2006 | 17419.86039 | 27985.80481 | 8138.594336 |
| DALYs (Disability-Adjusted Life Years) | Low SDI | Both | 65+ years | diarrhea diseases | Unsafe water source | Percent | 2006 | 1.52699045 | 1.86058056 | 0.905673434 |
| DALYs (Disability-Adjusted Life Years) | Low SDI | Both | 65+ years | diarrhea diseases | Unsafe water source | Number | 2006 | 2220531.903 | 3557510.808 | 1040727.665 |
| Deaths | High-middle SDI | Both | 65+ years | diarrhea diseases | Unsafe water source | Rate | 2006 | 4.314274316 | 7.707533014 | 1.21643863 |
| Deaths | High-middle SDI | Both | 65+ years | diarrhea diseases | Unsafe water source | Percent | 2006 | 0.958990854 | 1.465255106 | 0.350233691 |
| Deaths | High-middle SDI | Both | 65+ years | diarrhea diseases | Unsafe water source | Number | 2006 | 3150.196967 | 5635.816014 | 887.2299673 |
| DALYs (Disability-Adjusted Life Years) | High-middle SDI | Both | 65+ years | diarrhea diseases | Unsafe water source | Rate | 2006 | 82.73445236 | 143.8625935 | 24.3944697 |
| DALYs (Disability-Adjusted Life Years) | High-middle SDI | Both | 65+ years | diarrhea diseases | Unsafe water source | Percent | 2006 | 0.90693291 | 1.399004896 | 0.330243871 |
| DALYs (Disability-Adjusted Life Years) | High-middle SDI | Both | 65+ years | diarrhea diseases | Unsafe water source | Number | 2006 | 56021.73183 | 97836.0856 | 16524.39142 |
| Deaths | High SDI | Both | 65+ years | diarrhea diseases | Unsafe water source | Rate | 2006 | 1.742038801 | 4.313967151 | 0.313213108 |
| Deaths | High SDI | Both | 65+ years | diarrhea diseases | Unsafe water source | Percent | 2006 | 0.15409561 | 0.372745447 | 0.029447597 |
| Deaths | High SDI | Both | 65+ years | diarrhea diseases | Unsafe water source | Number | 2006 | 1609.829216 | 3992.383156 | 288.1946001 |
| DALYs (Disability-Adjusted Life Years) | High SDI | Both | 65+ years | diarrhea diseases | Unsafe water source | Rate | 2006 | 31.12441499 | 74.97361406 | 6.075055646 |
| DALYs (Disability-Adjusted Life Years) | High SDI | Both | 65+ years | diarrhea diseases | Unsafe water source | Percent | 2006 | 0.155523327 | 0.366739181 | 0.030941226 |
| DALYs (Disability-Adjusted Life Years) | High SDI | Both | 65+ years | diarrhea diseases | Unsafe water source | Number | 2006 | 26337.96578 | 63533.17762 | 5105.960927 |
| Deaths | Global | Both | 65+ years | diarrhea diseases | Unsafe water source | Rate | 2006 | 174.242211 | 290.2752447 | 74.55873784 |
| Deaths | Global | Both | 65+ years | diarrhea diseases | Unsafe water source | Percent | 2006 | 1.410922725 | 1.787350059 | 0.761614595 |
| Deaths | Global | Both | 65+ years | diarrhea diseases | Unsafe water source | Number | 2006 | 493744.6784 | 815615.2246 | 212137.2874 |
| DALYs (Disability-Adjusted Life Years) | Global | Both | 65+ years | diarrhea diseases | Unsafe water source | Rate | 2006 | 2999.818381 | 5008.268633 | 1281.423709 |
| DALYs (Disability-Adjusted Life Years) | Global | Both | 65+ years | diarrhea diseases | Unsafe water source | Percent | 2006 | 1.404497874 | 1.780173375 | 0.757750826 |
| DALYs (Disability-Adjusted Life Years) | Global | Both | 65+ years | diarrhea diseases | Unsafe water source | Number | 2006 | 7986157.436 | 13198184.26 | 3431816.424 |
| Deaths | Middle SDI | Both | 65+ years | diarrhea diseases | Unsafe water source | Rate | 2005 | 96.07609442 | 155.9371887 | 32.28791151 |
| Deaths | Middle SDI | Both | 65+ years | diarrhea diseases | Unsafe water source | Percent | 2005 | 1.272997097 | 1.735427573 | 0.526240069 |
| Deaths | Middle SDI | Both | 65+ years | diarrhea diseases | Unsafe water source | Number | 2005 | 69395.26569 | 112272.4592 | 23390.73294 |
| DALYs (Disability-Adjusted Life Years) | Middle SDI | Both | 65+ years | diarrhea diseases | Unsafe water source | Rate | 2005 | 1614.46219 | 2606.118542 | 555.720885 |
| DALYs (Disability-Adjusted Life Years) | Middle SDI | Both | 65+ years | diarrhea diseases | Unsafe water source | Percent | 2005 | 1.271003158 | 1.734070966 | 0.524101415 |
| DALYs (Disability-Adjusted Life Years) | Middle SDI | Both | 65+ years | diarrhea diseases | Unsafe water source | Number | 2005 | 1111510.618 | 1788308.239 | 384287.4711 |
| Deaths | Low-middle SDI | Both | 65+ years | diarrhea diseases | Unsafe water source | Rate | 2005 | 724.2771598 | 1246.767849 | 303.5596822 |
| Deaths | Low-middle SDI | Both | 65+ years | diarrhea diseases | Unsafe water source | Percent | 2005 | 1.456397838 | 1.832429097 | 0.776409539 |
| Deaths | Low-middle SDI | Both | 65+ years | diarrhea diseases | Unsafe water source | Number | 2005 | 285865.846 | 490063.4703 | 120220.568 |
| DALYs (Disability-Adjusted Life Years) | Low-middle SDI | Both | 65+ years | diarrhea diseases | Unsafe water source | Rate | 2005 | 12107.42194 | 20791.8878 | 5071.520121 |
| DALYs (Disability-Adjusted Life Years) | Low-middle SDI | Both | 65+ years | diarrhea diseases | Unsafe water source | Percent | 2005 | 1.456004642 | 1.832041335 | 0.775687932 |
| DALYs (Disability-Adjusted Life Years) | Low-middle SDI | Both | 65+ years | diarrhea diseases | Unsafe water source | Number | 2005 | 4598106.161 | 7855629.489 | 1933446.128 |
| Deaths | Low SDI | Both | 65+ years | diarrhea diseases | Unsafe water source | Rate | 2005 | 1032.568906 | 1668.289894 | 491.3118165 |
| Deaths | Low SDI | Both | 65+ years | diarrhea diseases | Unsafe water source | Percent | 2005 | 1.52779016 | 1.858901197 | 0.907867386 |
| Deaths | Low SDI | Both | 65+ years | diarrhea diseases | Unsafe water source | Number | 2005 | 132399.8103 | 213025.6898 | 63133.87776 |
| DALYs (Disability-Adjusted Life Years) | Low SDI | Both | 65+ years | diarrhea diseases | Unsafe water source | Rate | 2005 | 17816.6005 | 28871.89316 | 8524.997814 |
| DALYs (Disability-Adjusted Life Years) | Low SDI | Both | 65+ years | diarrhea diseases | Unsafe water source | Percent | 2005 | 1.527955675 | 1.856557419 | 0.907401292 |
| DALYs (Disability-Adjusted Life Years) | Low SDI | Both | 65+ years | diarrhea diseases | Unsafe water source | Number | 2005 | 2206748.404 | 3557664.426 | 1059513.503 |
| Deaths | High-middle SDI | Both | 65+ years | diarrhea diseases | Unsafe water source | Rate | 2005 | 4.50612015 | 8.074037519 | 1.304315794 |
| Deaths | High-middle SDI | Both | 65+ years | diarrhea diseases | Unsafe water source | Percent | 2005 | 0.976770337 | 1.480555115 | 0.358479825 |
| Deaths | High-middle SDI | Both | 65+ years | diarrhea diseases | Unsafe water source | Number | 2005 | 3215.307068 | 5774.737748 | 931.5615543 |
| DALYs (Disability-Adjusted Life Years) | High-middle SDI | Both | 65+ years | diarrhea diseases | Unsafe water source | Rate | 2005 | 85.92267067 | 150.2177071 | 25.82391189 |
| DALYs (Disability-Adjusted Life Years) | High-middle SDI | Both | 65+ years | diarrhea diseases | Unsafe water source | Percent | 2005 | 0.925852086 | 1.417396212 | 0.338606904 |
| DALYs (Disability-Adjusted Life Years) | High-middle SDI | Both | 65+ years | diarrhea diseases | Unsafe water source | Number | 2005 | 57023.2647 | 100128.3938 | 17109.49065 |
| Deaths | High SDI | Both | 65+ years | diarrhea diseases | Unsafe water source | Rate | 2005 | 1.603625601 | 3.890610224 | 0.296719813 |
| Deaths | High SDI | Both | 65+ years | diarrhea diseases | Unsafe water source | Percent | 2005 | 0.162925495 | 0.387797215 | 0.031932657 |
| Deaths | High SDI | Both | 65+ years | diarrhea diseases | Unsafe water source | Number | 2005 | 1446.098148 | 3514.831436 | 266.394665 |
| DALYs (Disability-Adjusted Life Years) | High SDI | Both | 65+ years | diarrhea diseases | Unsafe water source | Rate | 2005 | 29.3039021 | 69.64492374 | 5.773815834 |
| DALYs (Disability-Adjusted Life Years) | High SDI | Both | 65+ years | diarrhea diseases | Unsafe water source | Percent | 2005 | 0.162053455 | 0.380763441 | 0.032785081 |
| DALYs (Disability-Adjusted Life Years) | High SDI | Both | 65+ years | diarrhea diseases | Unsafe water source | Number | 2005 | 24154.96421 | 57531.10397 | 4722.034439 |
| Deaths | Global | Both | 65+ years | diarrhea diseases | Unsafe water source | Rate | 2005 | 178.7253559 | 295.1201397 | 76.77380941 |
| Deaths | Global | Both | 65+ years | diarrhea diseases | Unsafe water source | Percent | 2005 | 1.417159717 | 1.792784009 | 0.75997572 |
| Deaths | Global | Both | 65+ years | diarrhea diseases | Unsafe water source | Number | 2005 | 492458.6909 | 806866.5652 | 212455.7506 |
| DALYs (Disability-Adjusted Life Years) | Global | Both | 65+ years | diarrhea diseases | Unsafe water source | Rate | 2005 | 3085.684756 | 5099.831103 | 1322.116447 |
| DALYs (Disability-Adjusted Life Years) | Global | Both | 65+ years | diarrhea diseases | Unsafe water source | Percent | 2005 | 1.410649425 | 1.784809531 | 0.756171832 |
| DALYs (Disability-Adjusted Life Years) | Global | Both | 65+ years | diarrhea diseases | Unsafe water source | Number | 2005 | 7999757.227 | 13090631.82 | 3448551.688 |
| Deaths | Middle SDI | Both | 65+ years | diarrhea diseases | Unsafe water source | Rate | 2004 | 101.0876707 | 162.9423502 | 34.81680534 |
| Deaths | Middle SDI | Both | 65+ years | diarrhea diseases | Unsafe water source | Percent | 2004 | 1.282231172 | 1.741628462 | 0.530368495 |
| Deaths | Middle SDI | Both | 65+ years | diarrhea diseases | Unsafe water source | Number | 2004 | 70553.60231 | 113284.2853 | 24383.5093 |
| DALYs (Disability-Adjusted Life Years) | Middle SDI | Both | 65+ years | diarrhea diseases | Unsafe water source | Rate | 2004 | 1699.676574 | 2723.243246 | 594.3594402 |
| DALYs (Disability-Adjusted Life Years) | Middle SDI | Both | 65+ years | diarrhea diseases | Unsafe water source | Percent | 2004 | 1.280292814 | 1.738758905 | 0.528421119 |
| DALYs (Disability-Adjusted Life Years) | Middle SDI | Both | 65+ years | diarrhea diseases | Unsafe water source | Number | 2004 | 1132655.739 | 1807133.945 | 397957.9858 |
| Deaths | Low-middle SDI | Both | 65+ years | diarrhea diseases | Unsafe water source | Rate | 2004 | 755.3751072 | 1289.488746 | 325.6555039 |
| Deaths | Low-middle SDI | Both | 65+ years | diarrhea diseases | Unsafe water source | Percent | 2004 | 1.459718527 | 1.830050498 | 0.785824673 |
| Deaths | Low-middle SDI | Both | 65+ years | diarrhea diseases | Unsafe water source | Number | 2004 | 288588.5882 | 490064.0252 | 124710.2245 |
| DALYs (Disability-Adjusted Life Years) | Low-middle SDI | Both | 65+ years | diarrhea diseases | Unsafe water source | Rate | 2004 | 12623.64129 | 21659.65771 | 5428.240585 |
| DALYs (Disability-Adjusted Life Years) | Low-middle SDI | Both | 65+ years | diarrhea diseases | Unsafe water source | Percent | 2004 | 1.459291189 | 1.828716951 | 0.785134604 |
| DALYs (Disability-Adjusted Life Years) | Low-middle SDI | Both | 65+ years | diarrhea diseases | Unsafe water source | Number | 2004 | 4643442.071 | 7918997.345 | 2002567.497 |
| Deaths | Low SDI | Both | 65+ years | diarrhea diseases | Unsafe water source | Rate | 2004 | 1069.180832 | 1704.323394 | 508.5195886 |
| Deaths | Low SDI | Both | 65+ years | diarrhea diseases | Unsafe water source | Percent | 2004 | 1.52943366 | 1.860110239 | 0.910686762 |
| Deaths | Low SDI | Both | 65+ years | diarrhea diseases | Unsafe water source | Number | 2004 | 133164.5712 | 211349.2609 | 63449.93825 |
| DALYs (Disability-Adjusted Life Years) | Low SDI | Both | 65+ years | diarrhea diseases | Unsafe water source | Rate | 2004 | 18461.17063 | 29495.02846 | 8770.685194 |
| DALYs (Disability-Adjusted Life Years) | Low SDI | Both | 65+ years | diarrhea diseases | Unsafe water source | Percent | 2004 | 1.529631834 | 1.861236629 | 0.910361664 |
| DALYs (Disability-Adjusted Life Years) | Low SDI | Both | 65+ years | diarrhea diseases | Unsafe water source | Number | 2004 | 2222699.066 | 3531420.443 | 1058893.886 |
| Deaths | High-middle SDI | Both | 65+ years | diarrhea diseases | Unsafe water source | Rate | 2004 | 4.669304413 | 8.394293533 | 1.37158702 |
| Deaths | High-middle SDI | Both | 65+ years | diarrhea diseases | Unsafe water source | Percent | 2004 | 0.996469037 | 1.498747541 | 0.366151856 |
| Deaths | High-middle SDI | Both | 65+ years | diarrhea diseases | Unsafe water source | Number | 2004 | 3262.321876 | 5876.033478 | 959.3071131 |
| DALYs (Disability-Adjusted Life Years) | High-middle SDI | Both | 65+ years | diarrhea diseases | Unsafe water source | Rate | 2004 | 88.747891 | 153.9792786 | 26.89145882 |
| DALYs (Disability-Adjusted Life Years) | High-middle SDI | Both | 65+ years | diarrhea diseases | Unsafe water source | Percent | 2004 | 0.945557303 | 1.435253915 | 0.348379453 |
| DALYs (Disability-Adjusted Life Years) | High-middle SDI | Both | 65+ years | diarrhea diseases | Unsafe water source | Number | 2004 | 57736.3167 | 100716.6305 | 17517.91479 |
| Deaths | High SDI | Both | 65+ years | diarrhea diseases | Unsafe water source | Rate | 2004 | 1.48256672 | 3.583328083 | 0.283844509 |
| Deaths | High SDI | Both | 65+ years | diarrhea diseases | Unsafe water source | Percent | 2004 | 0.176092991 | 0.414466087 | 0.035364577 |
| Deaths | High SDI | Both | 65+ years | diarrhea diseases | Unsafe water source | Number | 2004 | 1305.553828 | 3165.540481 | 248.9444169 |
| DALYs (Disability-Adjusted Life Years) | High SDI | Both | 65+ years | diarrhea diseases | Unsafe water source | Rate | 2004 | 27.64521295 | 65.04640899 | 5.469239705 |
| DALYs (Disability-Adjusted Life Years) | High SDI | Both | 65+ years | diarrhea diseases | Unsafe water source | Percent | 2004 | 0.171635165 | 0.40110966 | 0.036029754 |
| DALYs (Disability-Adjusted Life Years) | High SDI | Both | 65+ years | diarrhea diseases | Unsafe water source | Number | 2004 | 22219.69793 | 52387.26108 | 4344.808413 |
| Deaths | Global | Both | 65+ years | diarrhea diseases | Unsafe water source | Rate | 2004 | 185.1597126 | 303.242199 | 82.49101695 |
| Deaths | Global | Both | 65+ years | diarrhea diseases | Unsafe water source | Percent | 2004 | 1.425171144 | 1.799050583 | 0.767123092 |
| Deaths | Global | Both | 65+ years | diarrhea diseases | Unsafe water source | Number | 2004 | 497012.128 | 806898.29 | 222424.5377 |
| DALYs (Disability-Adjusted Life Years) | Global | Both | 65+ years | diarrhea diseases | Unsafe water source | Rate | 2004 | 3196.813682 | 5252.901457 | 1421.598226 |
| DALYs (Disability-Adjusted Life Years) | Global | Both | 65+ years | diarrhea diseases | Unsafe water source | Percent | 2004 | 1.418570598 | 1.792009231 | 0.762712006 |
| DALYs (Disability-Adjusted Life Years) | Global | Both | 65+ years | diarrhea diseases | Unsafe water source | Number | 2004 | 8080990.986 | 13133897.75 | 3616412.16 |
| Deaths | Middle SDI | Both | 65+ years | diarrhea diseases | Unsafe water source | Rate | 2003 | 104.171224 | 170.6389721 | 36.16166552 |
| Deaths | Middle SDI | Both | 65+ years | diarrhea diseases | Unsafe water source | Percent | 2003 | 1.286921385 | 1.744928265 | 0.534174052 |
| Deaths | Middle SDI | Both | 65+ years | diarrhea diseases | Unsafe water source | Number | 2003 | 70199.48046 | 114686.6813 | 24444.19787 |
| DALYs (Disability-Adjusted Life Years) | Middle SDI | Both | 65+ years | diarrhea diseases | Unsafe water source | Rate | 2003 | 1754.811976 | 2857.101676 | 618.9903108 |
| DALYs (Disability-Adjusted Life Years) | Middle SDI | Both | 65+ years | diarrhea diseases | Unsafe water source | Percent | 2003 | 1.285039716 | 1.742323901 | 0.53203529 |
| DALYs (Disability-Adjusted Life Years) | Middle SDI | Both | 65+ years | diarrhea diseases | Unsafe water source | Number | 2003 | 1131105.762 | 1837188.227 | 400722.3284 |
| Deaths | Low-middle SDI | Both | 65+ years | diarrhea diseases | Unsafe water source | Rate | 2003 | 784.5823691 | 1330.090578 | 342.281007 |
| Deaths | Low-middle SDI | Both | 65+ years | diarrhea diseases | Unsafe water source | Percent | 2003 | 1.463155498 | 1.832877628 | 0.796445682 |
| Deaths | Low-middle SDI | Both | 65+ years | diarrhea diseases | Unsafe water source | Number | 2003 | 289360.7623 | 488173.8916 | 126658.241 |
| DALYs (Disability-Adjusted Life Years) | Low-middle SDI | Both | 65+ years | diarrhea diseases | Unsafe water source | Rate | 2003 | 13197.89737 | 22423.33813 | 5727.107548 |
| DALYs (Disability-Adjusted Life Years) | Low-middle SDI | Both | 65+ years | diarrhea diseases | Unsafe water source | Percent | 2003 | 1.462769171 | 1.832162389 | 0.795492521 |
| DALYs (Disability-Adjusted Life Years) | Low-middle SDI | Both | 65+ years | diarrhea diseases | Unsafe water source | Number | 2003 | 4686675.294 | 7914304.725 | 2041576.096 |
| Deaths | Low SDI | Both | 65+ years | diarrhea diseases | Unsafe water source | Rate | 2003 | 1112.390177 | 1792.227202 | 527.0342276 |
| Deaths | Low SDI | Both | 65+ years | diarrhea diseases | Unsafe water source | Percent | 2003 | 1.530904243 | 1.858581958 | 0.914753289 |
| Deaths | Low SDI | Both | 65+ years | diarrhea diseases | Unsafe water source | Number | 2003 | 134622.0845 | 215835.5449 | 63966.45802 |
| DALYs (Disability-Adjusted Life Years) | Low SDI | Both | 65+ years | diarrhea diseases | Unsafe water source | Rate | 2003 | 19274.53653 | 31242.4075 | 9055.979658 |
| DALYs (Disability-Adjusted Life Years) | Low SDI | Both | 65+ years | diarrhea diseases | Unsafe water source | Percent | 2003 | 1.53112105 | 1.857862562 | 0.914820932 |
| DALYs (Disability-Adjusted Life Years) | Low SDI | Both | 65+ years | diarrhea diseases | Unsafe water source | Number | 2003 | 2256310.944 | 3635749.115 | 1062830.237 |
| Deaths | High-middle SDI | Both | 65+ years | diarrhea diseases | Unsafe water source | Rate | 2003 | 4.842281473 | 8.64234643 | 1.422555465 |
| Deaths | High-middle SDI | Both | 65+ years | diarrhea diseases | Unsafe water source | Percent | 2003 | 1.008309496 | 1.508203096 | 0.372688123 |
| Deaths | High-middle SDI | Both | 65+ years | diarrhea diseases | Unsafe water source | Number | 2003 | 3308.320369 | 5929.389663 | 974.6544044 |
| DALYs (Disability-Adjusted Life Years) | High-middle SDI | Both | 65+ years | diarrhea diseases | Unsafe water source | Rate | 2003 | 91.78224981 | 157.1927391 | 28.58715133 |
| DALYs (Disability-Adjusted Life Years) | High-middle SDI | Both | 65+ years | diarrhea diseases | Unsafe water source | Percent | 2003 | 0.960258642 | 1.448342485 | 0.35577325 |
| DALYs (Disability-Adjusted Life Years) | High-middle SDI | Both | 65+ years | diarrhea diseases | Unsafe water source | Number | 2003 | 58383.9217 | 100726.5535 | 18166.89789 |
| Deaths | High SDI | Both | 65+ years | diarrhea diseases | Unsafe water source | Rate | 2003 | 1.384913556 | 3.324962983 | 0.267475877 |
| Deaths | High SDI | Both | 65+ years | diarrhea diseases | Unsafe water source | Percent | 2003 | 0.188835725 | 0.442880521 | 0.038527355 |
| Deaths | High SDI | Both | 65+ years | diarrhea diseases | Unsafe water source | Number | 2003 | 1194.446267 | 2877.499104 | 229.1677144 |
| DALYs (Disability-Adjusted Life Years) | High SDI | Both | 65+ years | diarrhea diseases | Unsafe water source | Rate | 2003 | 26.13714807 | 61.38624716 | 5.261636593 |
| DALYs (Disability-Adjusted Life Years) | High SDI | Both | 65+ years | diarrhea diseases | Unsafe water source | Percent | 2003 | 0.180776955 | 0.423581311 | 0.038766206 |
| DALYs (Disability-Adjusted Life Years) | High SDI | Both | 65+ years | diarrhea diseases | Unsafe water source | Number | 2003 | 20545.36737 | 48344.00289 | 4098.545856 |
| Deaths | Global | Both | 65+ years | diarrhea diseases | Unsafe water source | Rate | 2003 | 191.2596706 | 315.2069466 | 84.88240847 |
| Deaths | Global | Both | 65+ years | diarrhea diseases | Unsafe water source | Percent | 2003 | 1.431997209 | 1.804826117 | 0.775218999 |
| Deaths | Global | Both | 65+ years | diarrhea diseases | Unsafe water source | Number | 2003 | 498823.2531 | 815766.832 | 222053.2728 |
| DALYs (Disability-Adjusted Life Years) | Global | Both | 65+ years | diarrhea diseases | Unsafe water source | Rate | 2003 | 3319.653964 | 5485.25338 | 1471.410838 |
| DALYs (Disability-Adjusted Life Years) | Global | Both | 65+ years | diarrhea diseases | Unsafe water source | Percent | 2003 | 1.425744872 | 1.798938448 | 0.772147939 |
| DALYs (Disability-Adjusted Life Years) | Global | Both | 65+ years | diarrhea diseases | Unsafe water source | Number | 2003 | 8155278.119 | 13347386.82 | 3631526.754 |
| Deaths | Middle SDI | Both | 65+ years | diarrhea diseases | Unsafe water source | Rate | 2002 | 107.3753944 | 172.9050498 | 38.13162097 |
| Deaths | Middle SDI | Both | 65+ years | diarrhea diseases | Unsafe water source | Percent | 2002 | 1.292545887 | 1.749729323 | 0.544750351 |
| Deaths | Middle SDI | Both | 65+ years | diarrhea diseases | Unsafe water source | Number | 2002 | 69845.85373 | 112123.5359 | 24904.64823 |
| DALYs (Disability-Adjusted Life Years) | Middle SDI | Both | 65+ years | diarrhea diseases | Unsafe water source | Rate | 2002 | 1811.316441 | 2908.930484 | 651.9001667 |
| DALYs (Disability-Adjusted Life Years) | Middle SDI | Both | 65+ years | diarrhea diseases | Unsafe water source | Percent | 2002 | 1.290662233 | 1.74694203 | 0.542946119 |
| DALYs (Disability-Adjusted Life Years) | Middle SDI | Both | 65+ years | diarrhea diseases | Unsafe water source | Number | 2002 | 1128517.429 | 1806760.004 | 408359.5425 |
| Deaths | Low-middle SDI | Both | 65+ years | diarrhea diseases | Unsafe water source | Rate | 2002 | 808.0266242 | 1362.003803 | 359.2021179 |
| Deaths | Low-middle SDI | Both | 65+ years | diarrhea diseases | Unsafe water source | Percent | 2002 | 1.466873613 | 1.833118078 | 0.794130962 |
| Deaths | Low-middle SDI | Both | 65+ years | diarrhea diseases | Unsafe water source | Number | 2002 | 287292.2997 | 482038.8758 | 128254.3545 |
| DALYs (Disability-Adjusted Life Years) | Low-middle SDI | Both | 65+ years | diarrhea diseases | Unsafe water source | Rate | 2002 | 13703.10583 | 23169.51965 | 6047.671088 |
| DALYs (Disability-Adjusted Life Years) | Low-middle SDI | Both | 65+ years | diarrhea diseases | Unsafe water source | Percent | 2002 | 1.466494053 | 1.83243518 | 0.793285931 |
| DALYs (Disability-Adjusted Life Years) | Low-middle SDI | Both | 65+ years | diarrhea diseases | Unsafe water source | Number | 2002 | 4691611.771 | 7887675.698 | 2080700.933 |
| Deaths | Low SDI | Both | 65+ years | diarrhea diseases | Unsafe water source | Rate | 2002 | 1138.548096 | 1804.287036 | 538.5166286 |
| Deaths | Low SDI | Both | 65+ years | diarrhea diseases | Unsafe water source | Percent | 2002 | 1.532692751 | 1.859389272 | 0.919365238 |
| Deaths | Low SDI | Both | 65+ years | diarrhea diseases | Unsafe water source | Number | 2002 | 133852.0162 | 211130.9425 | 63459.29533 |
| DALYs (Disability-Adjusted Life Years) | Low SDI | Both | 65+ years | diarrhea diseases | Unsafe water source | Rate | 2002 | 19849.99862 | 31614.31047 | 9391.469407 |
| DALYs (Disability-Adjusted Life Years) | Low SDI | Both | 65+ years | diarrhea diseases | Unsafe water source | Percent | 2002 | 1.532904764 | 1.858733904 | 0.919539065 |
| DALYs (Disability-Adjusted Life Years) | Low SDI | Both | 65+ years | diarrhea diseases | Unsafe water source | Number | 2002 | 2258642.619 | 3577290.913 | 1072293.489 |
| Deaths | High-middle SDI | Both | 65+ years | diarrhea diseases | Unsafe water source | Rate | 2002 | 5.008380223 | 8.910853027 | 1.505336704 |
| Deaths | High-middle SDI | Both | 65+ years | diarrhea diseases | Unsafe water source | Percent | 2002 | 1.032847932 | 1.53486385 | 0.38903149 |
| Deaths | High-middle SDI | Both | 65+ years | diarrhea diseases | Unsafe water source | Number | 2002 | 3339.357835 | 5952.827493 | 1007.383477 |
| DALYs (Disability-Adjusted Life Years) | High-middle SDI | Both | 65+ years | diarrhea diseases | Unsafe water source | Rate | 2002 | 94.77445009 | 162.3171185 | 29.93001685 |
| DALYs (Disability-Adjusted Life Years) | High-middle SDI | Both | 65+ years | diarrhea diseases | Unsafe water source | Percent | 2002 | 0.984424076 | 1.480028073 | 0.370803237 |
| DALYs (Disability-Adjusted Life Years) | High-middle SDI | Both | 65+ years | diarrhea diseases | Unsafe water source | Number | 2002 | 58849.17065 | 101546.9682 | 18649.25487 |
| Deaths | High SDI | Both | 65+ years | diarrhea diseases | Unsafe water source | Rate | 2002 | 1.300209265 | 3.101416077 | 0.256788534 |
| Deaths | High SDI | Both | 65+ years | diarrhea diseases | Unsafe water source | Percent | 2002 | 0.203924718 | 0.476048017 | 0.042672465 |
| Deaths | High SDI | Both | 65+ years | diarrhea diseases | Unsafe water source | Number | 2002 | 1098.034571 | 2627.207479 | 215.2917897 |
| DALYs (Disability-Adjusted Life Years) | High SDI | Both | 65+ years | diarrhea diseases | Unsafe water source | Rate | 2002 | 24.75699971 | 57.69405022 | 5.062488402 |
| DALYs (Disability-Adjusted Life Years) | High SDI | Both | 65+ years | diarrhea diseases | Unsafe water source | Percent | 2002 | 0.191109193 | 0.445783898 | 0.041628927 |
| DALYs (Disability-Adjusted Life Years) | High SDI | Both | 65+ years | diarrhea diseases | Unsafe water source | Number | 2002 | 19023.08232 | 44628.35062 | 3868.310125 |
| Deaths | Global | Both | 65+ years | diarrhea diseases | Unsafe water source | Rate | 2002 | 195.8300736 | 320.5990869 | 87.49326102 |
| Deaths | Global | Both | 65+ years | diarrhea diseases | Unsafe water source | Percent | 2002 | 1.438454116 | 1.804739352 | 0.776396354 |
| Deaths | Global | Both | 65+ years | diarrhea diseases | Unsafe water source | Number | 2002 | 495567.4915 | 805642.8251 | 222517.2122 |
| DALYs (Disability-Adjusted Life Years) | Global | Both | 65+ years | diarrhea diseases | Unsafe water source | Rate | 2002 | 3422.861498 | 5628.33826 | 1523.486498 |
| DALYs (Disability-Adjusted Life Years) | Global | Both | 65+ years | diarrhea diseases | Unsafe water source | Percent | 2002 | 1.432544752 | 1.799694111 | 0.772728056 |
| DALYs (Disability-Adjusted Life Years) | Global | Both | 65+ years | diarrhea diseases | Unsafe water source | Number | 2002 | 8158936.056 | 13309335.94 | 3657091.365 |
| Deaths | Middle SDI | Both | 65+ years | diarrhea diseases | Unsafe water source | Rate | 2001 | 111.707798 | 181.741601 | 40.18118566 |
| Deaths | Middle SDI | Both | 65+ years | diarrhea diseases | Unsafe water source | Percent | 2001 | 1.299150481 | 1.755529048 | 0.553289422 |
| Deaths | Middle SDI | Both | 65+ years | diarrhea diseases | Unsafe water source | Number | 2001 | 70103.10028 | 113761.5259 | 25299.79159 |
| DALYs (Disability-Adjusted Life Years) | Middle SDI | Both | 65+ years | diarrhea diseases | Unsafe water source | Rate | 2001 | 1886.103527 | 3058.09417 | 693.6993618 |
| DALYs (Disability-Adjusted Life Years) | Middle SDI | Both | 65+ years | diarrhea diseases | Unsafe water source | Percent | 2001 | 1.297370251 | 1.754579528 | 0.551635601 |
| DALYs (Disability-Adjusted Life Years) | Middle SDI | Both | 65+ years | diarrhea diseases | Unsafe water source | Number | 2001 | 1134746.828 | 1835025.112 | 418604.3337 |
| Deaths | Low-middle SDI | Both | 65+ years | diarrhea diseases | Unsafe water source | Rate | 2001 | 843.1630191 | 1412.912009 | 377.1680189 |
| Deaths | Low-middle SDI | Both | 65+ years | diarrhea diseases | Unsafe water source | Percent | 2001 | 1.470625083 | 1.828718615 | 0.808473403 |
| Deaths | Low-middle SDI | Both | 65+ years | diarrhea diseases | Unsafe water source | Number | 2001 | 289288.5875 | 482444.0192 | 129844.7506 |
| DALYs (Disability-Adjusted Life Years) | Low-middle SDI | Both | 65+ years | diarrhea diseases | Unsafe water source | Rate | 2001 | 14343.82244 | 24116.94468 | 6366.691365 |
| DALYs (Disability-Adjusted Life Years) | Low-middle SDI | Both | 65+ years | diarrhea diseases | Unsafe water source | Percent | 2001 | 1.470273313 | 1.828179415 | 0.807853511 |
| DALYs (Disability-Adjusted Life Years) | Low-middle SDI | Both | 65+ years | diarrhea diseases | Unsafe water source | Number | 2001 | 4742169.18 | 7926363.382 | 2113555.735 |
| Deaths | Low SDI | Both | 65+ years | diarrhea diseases | Unsafe water source | Rate | 2001 | 1163.143598 | 1837.024889 | 549.9813168 |
| Deaths | Low SDI | Both | 65+ years | diarrhea diseases | Unsafe water source | Percent | 2001 | 1.534436733 | 1.854277637 | 0.927960416 |
| Deaths | Low SDI | Both | 65+ years | diarrhea diseases | Unsafe water source | Number | 2001 | 132867.5136 | 208900.5483 | 62915.32438 |
| DALYs (Disability-Adjusted Life Years) | Low SDI | Both | 65+ years | diarrhea diseases | Unsafe water source | Rate | 2001 | 20377.37176 | 32323.33345 | 9677.080776 |
| DALYs (Disability-Adjusted Life Years) | Low SDI | Both | 65+ years | diarrhea diseases | Unsafe water source | Percent | 2001 | 1.534625651 | 1.854156833 | 0.927894481 |
| DALYs (Disability-Adjusted Life Years) | Low SDI | Both | 65+ years | diarrhea diseases | Unsafe water source | Number | 2001 | 2253947.77 | 3555591.117 | 1072427.153 |
| Deaths | High-middle SDI | Both | 65+ years | diarrhea diseases | Unsafe water source | Rate | 2001 | 5.268272063 | 9.288432626 | 1.611273649 |
| Deaths | High-middle SDI | Both | 65+ years | diarrhea diseases | Unsafe water source | Percent | 2001 | 1.057059661 | 1.554107706 | 0.410321069 |
| Deaths | High-middle SDI | Both | 65+ years | diarrhea diseases | Unsafe water source | Number | 2001 | 3418.581814 | 6025.251283 | 1047.84048 |
| DALYs (Disability-Adjusted Life Years) | High-middle SDI | Both | 65+ years | diarrhea diseases | Unsafe water source | Rate | 2001 | 99.52336603 | 171.0743468 | 32.35912846 |
| DALYs (Disability-Adjusted Life Years) | High-middle SDI | Both | 65+ years | diarrhea diseases | Unsafe water source | Percent | 2001 | 1.010321957 | 1.503477218 | 0.389625471 |
| DALYs (Disability-Adjusted Life Years) | High-middle SDI | Both | 65+ years | diarrhea diseases | Unsafe water source | Number | 2001 | 60173.19949 | 104172.7046 | 19649.20518 |
| Deaths | High SDI | Both | 65+ years | diarrhea diseases | Unsafe water source | Rate | 2001 | 1.236856241 | 2.924085949 | 0.251301061 |
| Deaths | High SDI | Both | 65+ years | diarrhea diseases | Unsafe water source | Percent | 2001 | 0.221025001 | 0.509632413 | 0.048312235 |
| Deaths | High SDI | Both | 65+ years | diarrhea diseases | Unsafe water source | Number | 2001 | 1022.018303 | 2424.774975 | 205.9494324 |
| DALYs (Disability-Adjusted Life Years) | High SDI | Both | 65+ years | diarrhea diseases | Unsafe water source | Rate | 2001 | 23.77196956 | 55.19127302 | 4.957301262 |
| DALYs (Disability-Adjusted Life Years) | High SDI | Both | 65+ years | diarrhea diseases | Unsafe water source | Percent | 2001 | 0.203377729 | 0.472231537 | 0.045522006 |
| DALYs (Disability-Adjusted Life Years) | High SDI | Both | 65+ years | diarrhea diseases | Unsafe water source | Number | 2001 | 17855.59029 | 41686.51911 | 3703.594417 |
| Deaths | Global | Both | 65+ years | diarrhea diseases | Unsafe water source | Rate | 2001 | 202.1442859 | 326.206436 | 89.97947939 |
| Deaths | Global | Both | 65+ years | diarrhea diseases | Unsafe water source | Percent | 2001 | 1.444474686 | 1.809316714 | 0.791059692 |
| Deaths | Global | Both | 65+ years | diarrhea diseases | Unsafe water source | Number | 2001 | 496842.0457 | 795547.2949 | 222249.3237 |
| DALYs (Disability-Adjusted Life Years) | Global | Both | 65+ years | diarrhea diseases | Unsafe water source | Rate | 2001 | 3545.777942 | 5751.202228 | 1578.196851 |
| DALYs (Disability-Adjusted Life Years) | Global | Both | 65+ years | diarrhea diseases | Unsafe water source | Percent | 2001 | 1.43890493 | 1.804793518 | 0.787688044 |
| DALYs (Disability-Adjusted Life Years) | Global | Both | 65+ years | diarrhea diseases | Unsafe water source | Number | 2001 | 8211227.163 | 13195682.79 | 3677176.511 |
| Deaths | Middle SDI | Both | 65+ years | diarrhea diseases | Unsafe water source | Rate | 2000 | 116.8774662 | 188.683136 | 42.28087321 |
| Deaths | Middle SDI | Both | 65+ years | diarrhea diseases | Unsafe water source | Percent | 2000 | 1.305979682 | 1.756776341 | 0.564444061 |
| Deaths | Middle SDI | Both | 65+ years | diarrhea diseases | Unsafe water source | Number | 2000 | 70757.00695 | 113957.5773 | 25698.2967 |
| DALYs (Disability-Adjusted Life Years) | Middle SDI | Both | 65+ years | diarrhea diseases | Unsafe water source | Rate | 2000 | 1973.661116 | 3162.709386 | 728.7747273 |
| DALYs (Disability-Adjusted Life Years) | Middle SDI | Both | 65+ years | diarrhea diseases | Unsafe water source | Percent | 2000 | 1.304244999 | 1.752420099 | 0.562613623 |
| DALYs (Disability-Adjusted Life Years) | Middle SDI | Both | 65+ years | diarrhea diseases | Unsafe water source | Number | 2000 | 1146749.431 | 1832905.301 | 425605.6117 |
| Deaths | Low-middle SDI | Both | 65+ years | diarrhea diseases | Unsafe water source | Rate | 2000 | 886.5824112 | 1447.07374 | 402.8078838 |
| Deaths | Low-middle SDI | Both | 65+ years | diarrhea diseases | Unsafe water source | Percent | 2000 | 1.474899008 | 1.836864374 | 0.817148973 |
| Deaths | Low-middle SDI | Both | 65+ years | diarrhea diseases | Unsafe water source | Number | 2000 | 293675.3917 | 476753.6141 | 133801.3403 |
| DALYs (Disability-Adjusted Life Years) | Low-middle SDI | Both | 65+ years | diarrhea diseases | Unsafe water source | Rate | 2000 | 15112.8647 | 24881.04739 | 6814.585261 |
| DALYs (Disability-Adjusted Life Years) | Low-middle SDI | Both | 65+ years | diarrhea diseases | Unsafe water source | Percent | 2000 | 1.47454643 | 1.836392405 | 0.816457011 |
| DALYs (Disability-Adjusted Life Years) | Low-middle SDI | Both | 65+ years | diarrhea diseases | Unsafe water source | Number | 2000 | 4829592.92 | 7903065.462 | 2185194.78 |
| Deaths | Low SDI | Both | 65+ years | diarrhea diseases | Unsafe water source | Rate | 2000 | 1197.833886 | 1855.370416 | 571.4600411 |
| Deaths | Low SDI | Both | 65+ years | diarrhea diseases | Unsafe water source | Percent | 2000 | 1.536522709 | 1.860689435 | 0.934305652 |
| Deaths | Low SDI | Both | 65+ years | diarrhea diseases | Unsafe water source | Number | 2000 | 133209.2453 | 205363.4603 | 63699.6193 |
| DALYs (Disability-Adjusted Life Years) | Low SDI | Both | 65+ years | diarrhea diseases | Unsafe water source | Rate | 2000 | 21000.23163 | 32802.95199 | 9984.005496 |
| DALYs (Disability-Adjusted Life Years) | Low SDI | Both | 65+ years | diarrhea diseases | Unsafe water source | Percent | 2000 | 1.536751344 | 1.859849811 | 0.934166743 |
| DALYs (Disability-Adjusted Life Years) | Low SDI | Both | 65+ years | diarrhea diseases | Unsafe water source | Number | 2000 | 2262932.527 | 3515807.991 | 1078323.093 |
| Deaths | High-middle SDI | Both | 65+ years | diarrhea diseases | Unsafe water source | Rate | 2000 | 5.579094303 | 9.691550543 | 1.739716124 |
| Deaths | High-middle SDI | Both | 65+ years | diarrhea diseases | Unsafe water source | Percent | 2000 | 1.074689258 | 1.567002059 | 0.424658969 |
| Deaths | High-middle SDI | Both | 65+ years | diarrhea diseases | Unsafe water source | Number | 2000 | 3522.041937 | 6121.564564 | 1100.824451 |
| DALYs (Disability-Adjusted Life Years) | High-middle SDI | Both | 65+ years | diarrhea diseases | Unsafe water source | Rate | 2000 | 104.8904968 | 177.7782159 | 34.69168514 |
| DALYs (Disability-Adjusted Life Years) | High-middle SDI | Both | 65+ years | diarrhea diseases | Unsafe water source | Percent | 2000 | 1.031336083 | 1.516026965 | 0.408768773 |
| DALYs (Disability-Adjusted Life Years) | High-middle SDI | Both | 65+ years | diarrhea diseases | Unsafe water source | Number | 2000 | 61884.46677 | 105430.7918 | 20500.97037 |
| Deaths | High SDI | Both | 65+ years | diarrhea diseases | Unsafe water source | Rate | 2000 | 1.222565965 | 2.860151014 | 0.254915636 |
| Deaths | High SDI | Both | 65+ years | diarrhea diseases | Unsafe water source | Percent | 2000 | 0.242155568 | 0.552529624 | 0.054191866 |
| Deaths | High SDI | Both | 65+ years | diarrhea diseases | Unsafe water source | Number | 2000 | 985.8242306 | 2314.654001 | 203.9945106 |
| DALYs (Disability-Adjusted Life Years) | High SDI | Both | 65+ years | diarrhea diseases | Unsafe water source | Rate | 2000 | 23.70850838 | 54.78334148 | 5.036162546 |
| DALYs (Disability-Adjusted Life Years) | High SDI | Both | 65+ years | diarrhea diseases | Unsafe water source | Percent | 2000 | 0.220288247 | 0.504290866 | 0.050438335 |
| DALYs (Disability-Adjusted Life Years) | High SDI | Both | 65+ years | diarrhea diseases | Unsafe water source | Number | 2000 | 17364.68799 | 40295.37968 | 3673.128027 |
| Deaths | Global | Both | 65+ years | diarrhea diseases | Unsafe water source | Rate | 2000 | 210.1640146 | 335.7190286 | 94.41962414 |
| Deaths | Global | Both | 65+ years | diarrhea diseases | Unsafe water source | Percent | 2000 | 1.450307553 | 1.816285585 | 0.803034544 |
| Deaths | Global | Both | 65+ years | diarrhea diseases | Unsafe water source | Number | 2000 | 502295.5664 | 796680.6427 | 226312.7765 |
| DALYs (Disability-Adjusted Life Years) | Global | Both | 65+ years | diarrhea diseases | Unsafe water source | Rate | 2000 | 3689.893066 | 5915.906237 | 1667.555575 |
| DALYs (Disability-Adjusted Life Years) | Global | Both | 65+ years | diarrhea diseases | Unsafe water source | Percent | 2000 | 1.445150025 | 1.810601346 | 0.799781171 |
| DALYs (Disability-Adjusted Life Years) | Global | Both | 65+ years | diarrhea diseases | Unsafe water source | Number | 2000 | 8320925.324 | 13232624.72 | 3776714.309 |
| Deaths | Middle SDI | Both | 65+ years | diarrhea diseases | Unsafe water source | Rate | 1999 | 122.4468146 | 195.9796104 | 44.74009778 |
| Deaths | Middle SDI | Both | 65+ years | diarrhea diseases | Unsafe water source | Percent | 1999 | 1.311677491 | 1.756359857 | 0.569000318 |
| Deaths | Middle SDI | Both | 65+ years | diarrhea diseases | Unsafe water source | Number | 1999 | 71470.38058 | 114241.5749 | 26167.37452 |
| DALYs (Disability-Adjusted Life Years) | Middle SDI | Both | 65+ years | diarrhea diseases | Unsafe water source | Rate | 1999 | 2067.195154 | 3293.210148 | 769.0158761 |
| DALYs (Disability-Adjusted Life Years) | Middle SDI | Both | 65+ years | diarrhea diseases | Unsafe water source | Percent | 1999 | 1.31004882 | 1.754784855 | 0.567610484 |
| DALYs (Disability-Adjusted Life Years) | Middle SDI | Both | 65+ years | diarrhea diseases | Unsafe water source | Number | 1999 | 1159341.134 | 1845057.503 | 432558.7195 |
| Deaths | Low-middle SDI | Both | 65+ years | diarrhea diseases | Unsafe water source | Rate | 1999 | 949.235675 | 1550.025315 | 441.2258648 |
| Deaths | Low-middle SDI | Both | 65+ years | diarrhea diseases | Unsafe water source | Percent | 1999 | 1.478821578 | 1.833167139 | 0.822050646 |
| Deaths | Low-middle SDI | Both | 65+ years | diarrhea diseases | Unsafe water source | Number | 1999 | 304018.3585 | 494660.4661 | 141813.1909 |
| DALYs (Disability-Adjusted Life Years) | Low-middle SDI | Both | 65+ years | diarrhea diseases | Unsafe water source | Rate | 1999 | 16177.58841 | 26527.77542 | 7467.280364 |
| DALYs (Disability-Adjusted Life Years) | Low-middle SDI | Both | 65+ years | diarrhea diseases | Unsafe water source | Percent | 1999 | 1.47845331 | 1.833135466 | 0.821344696 |
| DALYs (Disability-Adjusted Life Years) | Low-middle SDI | Both | 65+ years | diarrhea diseases | Unsafe water source | Number | 1999 | 5006553.823 | 8175308.263 | 2320433.558 |
| Deaths | Low SDI | Both | 65+ years | diarrhea diseases | Unsafe water source | Rate | 1999 | 1236.072746 | 1926.523653 | 592.7578804 |
| Deaths | Low SDI | Both | 65+ years | diarrhea diseases | Unsafe water source | Percent | 1999 | 1.5379345 | 1.859966882 | 0.929177606 |
| Deaths | Low SDI | Both | 65+ years | diarrhea diseases | Unsafe water source | Number | 1999 | 133795.411 | 207614.2329 | 64335.50178 |
| DALYs (Disability-Adjusted Life Years) | Low SDI | Both | 65+ years | diarrhea diseases | Unsafe water source | Rate | 1999 | 21719.54586 | 34049.36887 | 10375.17756 |
| DALYs (Disability-Adjusted Life Years) | Low SDI | Both | 65+ years | diarrhea diseases | Unsafe water source | Percent | 1999 | 1.538159635 | 1.858027296 | 0.929356774 |
| DALYs (Disability-Adjusted Life Years) | Low SDI | Both | 65+ years | diarrhea diseases | Unsafe water source | Number | 1999 | 2279329.046 | 3554949.559 | 1092070.367 |
| Deaths | High-middle SDI | Both | 65+ years | diarrhea diseases | Unsafe water source | Rate | 1999 | 5.894107086 | 10.25664434 | 1.860036597 |
| Deaths | High-middle SDI | Both | 65+ years | diarrhea diseases | Unsafe water source | Percent | 1999 | 1.093311161 | 1.586722438 | 0.437063461 |
| Deaths | High-middle SDI | Both | 65+ years | diarrhea diseases | Unsafe water source | Number | 1999 | 3620.745865 | 6306.183633 | 1143.85775 |
| DALYs (Disability-Adjusted Life Years) | High-middle SDI | Both | 65+ years | diarrhea diseases | Unsafe water source | Rate | 1999 | 110.2734593 | 188.9893266 | 36.96207703 |
| DALYs (Disability-Adjusted Life Years) | High-middle SDI | Both | 65+ years | diarrhea diseases | Unsafe water source | Percent | 1999 | 1.051676302 | 1.537902815 | 0.421744274 |
| DALYs (Disability-Adjusted Life Years) | High-middle SDI | Both | 65+ years | diarrhea diseases | Unsafe water source | Number | 1999 | 63566.83593 | 109515.9845 | 21380.55996 |
| Deaths | High SDI | Both | 65+ years | diarrhea diseases | Unsafe water source | Rate | 1999 | 1.084761045 | 2.483889993 | 0.234504544 |
| Deaths | High SDI | Both | 65+ years | diarrhea diseases | Unsafe water source | Percent | 1999 | 0.258987029 | 0.578559491 | 0.060620227 |
| Deaths | High SDI | Both | 65+ years | diarrhea diseases | Unsafe water source | Number | 1999 | 851.5673893 | 1959.98729 | 182.5556279 |
| DALYs (Disability-Adjusted Life Years) | High SDI | Both | 65+ years | diarrhea diseases | Unsafe water source | Rate | 1999 | 21.91621961 | 50.0570363 | 4.829519729 |
| DALYs (Disability-Adjusted Life Years) | High SDI | Both | 65+ years | diarrhea diseases | Unsafe water source | Percent | 1999 | 0.232798287 | 0.52340948 | 0.055312139 |
| DALYs (Disability-Adjusted Life Years) | High SDI | Both | 65+ years | diarrhea diseases | Unsafe water source | Number | 1999 | 15586.48819 | 35790.21191 | 3420.064975 |
| Deaths | Global | Both | 65+ years | diarrhea diseases | Unsafe water source | Rate | 1999 | 220.892036 | 350.9029754 | 98.05275457 |
| Deaths | Global | Both | 65+ years | diarrhea diseases | Unsafe water source | Percent | 1999 | 1.45651563 | 1.821013057 | 0.811847774 |
| Deaths | Global | Both | 65+ years | diarrhea diseases | Unsafe water source | Number | 1999 | 513906.0757 | 811521.7922 | 228175.2139 |
| DALYs (Disability-Adjusted Life Years) | Global | Both | 65+ years | diarrhea diseases | Unsafe water source | Rate | 1999 | 3876.147717 | 6175.221447 | 1725.170556 |
| DALYs (Disability-Adjusted Life Years) | Global | Both | 65+ years | diarrhea diseases | Unsafe water source | Percent | 1999 | 1.451597085 | 1.815297857 | 0.808703348 |
| DALYs (Disability-Adjusted Life Years) | Global | Both | 65+ years | diarrhea diseases | Unsafe water source | Number | 1999 | 8526840.261 | 13492454.89 | 3799181.768 |
| Deaths | Middle SDI | Both | 65+ years | diarrhea diseases | Unsafe water source | Rate | 1998 | 129.5843479 | 210.0722502 | 47.28204981 |
| Deaths | Middle SDI | Both | 65+ years | diarrhea diseases | Unsafe water source | Percent | 1998 | 1.316849589 | 1.76025262 | 0.577953327 |
| Deaths | Middle SDI | Both | 65+ years | diarrhea diseases | Unsafe water source | Number | 1998 | 72945.73438 | 118011.7449 | 26643.60312 |
| DALYs (Disability-Adjusted Life Years) | Middle SDI | Both | 65+ years | diarrhea diseases | Unsafe water source | Rate | 1998 | 2182.452222 | 3524.819053 | 813.5485414 |
| DALYs (Disability-Adjusted Life Years) | Middle SDI | Both | 65+ years | diarrhea diseases | Unsafe water source | Percent | 1998 | 1.315452472 | 1.758562272 | 0.57675049 |
| DALYs (Disability-Adjusted Life Years) | Middle SDI | Both | 65+ years | diarrhea diseases | Unsafe water source | Number | 1998 | 1181404.113 | 1903791.075 | 440775.909 |
| Deaths | Low-middle SDI | Both | 65+ years | diarrhea diseases | Unsafe water source | Rate | 1998 | 1031.214455 | 1662.665908 | 479.9106916 |
| Deaths | Low-middle SDI | Both | 65+ years | diarrhea diseases | Unsafe water source | Percent | 1998 | 1.482771546 | 1.840544168 | 0.837126222 |
| Deaths | Low-middle SDI | Both | 65+ years | diarrhea diseases | Unsafe water source | Number | 1998 | 319682.1428 | 513045.8731 | 149329.8809 |
| DALYs (Disability-Adjusted Life Years) | Low-middle SDI | Both | 65+ years | diarrhea diseases | Unsafe water source | Rate | 1998 | 17504.29796 | 28355.43869 | 8080.985519 |
| DALYs (Disability-Adjusted Life Years) | Low-middle SDI | Both | 65+ years | diarrhea diseases | Unsafe water source | Percent | 1998 | 1.482388033 | 1.839685028 | 0.836680274 |
| DALYs (Disability-Adjusted Life Years) | Low-middle SDI | Both | 65+ years | diarrhea diseases | Unsafe water source | Number | 1998 | 5249099.888 | 8455788.943 | 2434379.931 |
| Deaths | Low SDI | Both | 65+ years | diarrhea diseases | Unsafe water source | Rate | 1998 | 1293.824441 | 2033.594173 | 612.518449 |
| Deaths | Low SDI | Both | 65+ years | diarrhea diseases | Unsafe water source | Percent | 1998 | 1.539728674 | 1.861734057 | 0.933275097 |
| Deaths | Low SDI | Both | 65+ years | diarrhea diseases | Unsafe water source | Number | 1998 | 136537.1779 | 213826.886 | 64717.87081 |
| DALYs (Disability-Adjusted Life Years) | Low SDI | Both | 65+ years | diarrhea diseases | Unsafe water source | Rate | 1998 | 22743.87717 | 35938.30455 | 10793.51918 |
| DALYs (Disability-Adjusted Life Years) | Low SDI | Both | 65+ years | diarrhea diseases | Unsafe water source | Percent | 1998 | 1.539968239 | 1.862014167 | 0.933286697 |
| DALYs (Disability-Adjusted Life Years) | Low SDI | Both | 65+ years | diarrhea diseases | Unsafe water source | Number | 1998 | 2327774.238 | 3662777.942 | 1106096.792 |
| Deaths | High-middle SDI | Both | 65+ years | diarrhea diseases | Unsafe water source | Rate | 1998 | 6.171617187 | 10.70484265 | 1.999003714 |
| Deaths | High-middle SDI | Both | 65+ years | diarrhea diseases | Unsafe water source | Percent | 1998 | 1.11659816 | 1.599026794 | 0.452108398 |
| Deaths | High-middle SDI | Both | 65+ years | diarrhea diseases | Unsafe water source | Number | 1998 | 3684.273194 | 6390.722373 | 1194.37504 |
| DALYs (Disability-Adjusted Life Years) | High-middle SDI | Both | 65+ years | diarrhea diseases | Unsafe water source | Rate | 1998 | 114.7277478 | 195.1672359 | 38.79138006 |
| DALYs (Disability-Adjusted Life Years) | High-middle SDI | Both | 65+ years | diarrhea diseases | Unsafe water source | Percent | 1998 | 1.075262362 | 1.554815602 | 0.43672396 |
| DALYs (Disability-Adjusted Life Years) | High-middle SDI | Both | 65+ years | diarrhea diseases | Unsafe water source | Number | 1998 | 64599.34079 | 110259.9151 | 21872.13115 |
| Deaths | High SDI | Both | 65+ years | diarrhea diseases | Unsafe water source | Rate | 1998 | 1.000620573 | 2.27133044 | 0.220662083 |
| Deaths | High SDI | Both | 65+ years | diarrhea diseases | Unsafe water source | Percent | 1998 | 0.274167108 | 0.594701426 | 0.066731781 |
| Deaths | High SDI | Both | 65+ years | diarrhea diseases | Unsafe water source | Number | 1998 | 767.9778352 | 1754.917937 | 167.9345962 |
| DALYs (Disability-Adjusted Life Years) | High SDI | Both | 65+ years | diarrhea diseases | Unsafe water source | Rate | 1998 | 20.73498712 | 46.46501039 | 4.713898558 |
| DALYs (Disability-Adjusted Life Years) | High SDI | Both | 65+ years | diarrhea diseases | Unsafe water source | Percent | 1998 | 0.244848047 | 0.540094248 | 0.059624255 |
| DALYs (Disability-Adjusted Life Years) | High SDI | Both | 65+ years | diarrhea diseases | Unsafe water source | Number | 1998 | 14402.64328 | 32488.69804 | 3263.913934 |
| Deaths | Global | Both | 65+ years | diarrhea diseases | Unsafe water source | Rate | 1998 | 235.5785857 | 374.8116459 | 103.8345675 |
| Deaths | Global | Both | 65+ years | diarrhea diseases | Unsafe water source | Percent | 1998 | 1.46226444 | 1.822473047 | 0.823123001 |
| Deaths | Global | Both | 65+ years | diarrhea diseases | Unsafe water source | Number | 1998 | 533770.4802 | 843627.1639 | 235471.8031 |
| DALYs (Disability-Adjusted Life Years) | Global | Both | 65+ years | diarrhea diseases | Unsafe water source | Rate | 1998 | 4117.760032 | 6568.810919 | 1830.517041 |
| DALYs (Disability-Adjusted Life Years) | Global | Both | 65+ years | diarrhea diseases | Unsafe water source | Percent | 1998 | 1.457753794 | 1.817667011 | 0.820156144 |
| DALYs (Disability-Adjusted Life Years) | Global | Both | 65+ years | diarrhea diseases | Unsafe water source | Number | 1998 | 8839805.263 | 13990523.04 | 3936891.597 |
| Deaths | Middle SDI | Both | 65+ years | diarrhea diseases | Unsafe water source | Rate | 1997 | 136.9485128 | 221.4302298 | 50.14748672 |
| Deaths | Middle SDI | Both | 65+ years | diarrhea diseases | Unsafe water source | Percent | 1997 | 1.324118339 | 1.759215354 | 0.592467367 |
| Deaths | Middle SDI | Both | 65+ years | diarrhea diseases | Unsafe water source | Number | 1997 | 74422.23883 | 120139.3886 | 27282.93347 |
| DALYs (Disability-Adjusted Life Years) | Middle SDI | Both | 65+ years | diarrhea diseases | Unsafe water source | Rate | 1997 | 2299.841787 | 3694.15593 | 856.0884212 |
| DALYs (Disability-Adjusted Life Years) | Middle SDI | Both | 65+ years | diarrhea diseases | Unsafe water source | Percent | 1997 | 1.323004024 | 1.758298873 | 0.591593476 |
| DALYs (Disability-Adjusted Life Years) | Middle SDI | Both | 65+ years | diarrhea diseases | Unsafe water source | Number | 1997 | 1203060.262 | 1928750.367 | 448311.8225 |
| Deaths | Low-middle SDI | Both | 65+ years | diarrhea diseases | Unsafe water source | Rate | 1997 | 1075.391998 | 1724.327953 | 499.2536739 |
| Deaths | Low-middle SDI | Both | 65+ years | diarrhea diseases | Unsafe water source | Percent | 1997 | 1.486450347 | 1.836458046 | 0.84590505 |
| Deaths | Low-middle SDI | Both | 65+ years | diarrhea diseases | Unsafe water source | Number | 1997 | 323011.445 | 515954.5885 | 150461.4424 |
| DALYs (Disability-Adjusted Life Years) | Low-middle SDI | Both | 65+ years | diarrhea diseases | Unsafe water source | Rate | 1997 | 18222.5275 | 29375.52256 | 8411.56228 |
| DALYs (Disability-Adjusted Life Years) | Low-middle SDI | Both | 65+ years | diarrhea diseases | Unsafe water source | Percent | 1997 | 1.486083875 | 1.837060765 | 0.845548071 |
| DALYs (Disability-Adjusted Life Years) | Low-middle SDI | Both | 65+ years | diarrhea diseases | Unsafe water source | Number | 1997 | 5298048.919 | 8501570.821 | 2456186.547 |
| Deaths | Low SDI | Both | 65+ years | diarrhea diseases | Unsafe water source | Rate | 1997 | 1324.843337 | 2075.547602 | 634.2618444 |
| Deaths | Low SDI | Both | 65+ years | diarrhea diseases | Unsafe water source | Percent | 1997 | 1.541199071 | 1.859838144 | 0.930514845 |
| Deaths | Low SDI | Both | 65+ years | diarrhea diseases | Unsafe water source | Number | 1997 | 136467.1965 | 213070.1762 | 65395.90497 |
| DALYs (Disability-Adjusted Life Years) | Low SDI | Both | 65+ years | diarrhea diseases | Unsafe water source | Rate | 1997 | 23266.88123 | 36550.54893 | 11148.24909 |
| DALYs (Disability-Adjusted Life Years) | Low SDI | Both | 65+ years | diarrhea diseases | Unsafe water source | Percent | 1997 | 1.541429145 | 1.86044565 | 0.930417966 |
| DALYs (Disability-Adjusted Life Years) | Low SDI | Both | 65+ years | diarrhea diseases | Unsafe water source | Number | 1997 | 2324803.453 | 3637517.901 | 1114544.65 |
| Deaths | High-middle SDI | Both | 65+ years | diarrhea diseases | Unsafe water source | Rate | 1997 | 6.500661852 | 11.06314981 | 2.120556864 |
| Deaths | High-middle SDI | Both | 65+ years | diarrhea diseases | Unsafe water source | Percent | 1997 | 1.134795815 | 1.621834001 | 0.468187836 |
| Deaths | High-middle SDI | Both | 65+ years | diarrhea diseases | Unsafe water source | Number | 1997 | 3765.390563 | 6413.508576 | 1228.678445 |
| DALYs (Disability-Adjusted Life Years) | High-middle SDI | Both | 65+ years | diarrhea diseases | Unsafe water source | Rate | 1997 | 120.20815 | 200.4440742 | 41.00335676 |
| DALYs (Disability-Adjusted Life Years) | High-middle SDI | Both | 65+ years | diarrhea diseases | Unsafe water source | Percent | 1997 | 1.094773604 | 1.575408015 | 0.453505159 |
| DALYs (Disability-Adjusted Life Years) | High-middle SDI | Both | 65+ years | diarrhea diseases | Unsafe water source | Number | 1997 | 65957.83208 | 110356.3431 | 22531.92742 |
| Deaths | High SDI | Both | 65+ years | diarrhea diseases | Unsafe water source | Rate | 1997 | 0.987317745 | 2.23272086 | 0.218557984 |
| Deaths | High SDI | Both | 65+ years | diarrhea diseases | Unsafe water source | Percent | 1997 | 0.289160561 | 0.620440664 | 0.071558195 |
| Deaths | High SDI | Both | 65+ years | diarrhea diseases | Unsafe water source | Number | 1997 | 740.6222175 | 1686.322407 | 162.5072565 |
| DALYs (Disability-Adjusted Life Years) | High SDI | Both | 65+ years | diarrhea diseases | Unsafe water source | Rate | 1997 | 20.58154619 | 45.61653477 | 4.722272255 |
| DALYs (Disability-Adjusted Life Years) | High SDI | Both | 65+ years | diarrhea diseases | Unsafe water source | Percent | 1997 | 0.258558279 | 0.562001579 | 0.063395996 |
| DALYs (Disability-Adjusted Life Years) | High SDI | Both | 65+ years | diarrhea diseases | Unsafe water source | Number | 1997 | 13995.27291 | 31213.31252 | 3203.370004 |
| Deaths | Global | Both | 65+ years | diarrhea diseases | Unsafe water source | Rate | 1997 | 244.088238 | 384.9315555 | 108.2230041 |
| Deaths | Global | Both | 65+ years | diarrhea diseases | Unsafe water source | Percent | 1997 | 1.466287571 | 1.825363428 | 0.830884271 |
| Deaths | Global | Both | 65+ years | diarrhea diseases | Unsafe water source | Number | 1997 | 538563.2119 | 843839.6263 | 238922.2415 |
| DALYs (Disability-Adjusted Life Years) | Global | Both | 65+ years | diarrhea diseases | Unsafe water source | Rate | 1997 | 4253.031464 | 6727.96612 | 1900.617091 |
| DALYs (Disability-Adjusted Life Years) | Global | Both | 65+ years | diarrhea diseases | Unsafe water source | Percent | 1997 | 1.462165545 | 1.818867564 | 0.828409632 |
| DALYs (Disability-Adjusted Life Years) | Global | Both | 65+ years | diarrhea diseases | Unsafe water source | Number | 1997 | 8908446.295 | 13984686.71 | 3986382.715 |
| Deaths | Middle SDI | Both | 65+ years | diarrhea diseases | Unsafe water source | Rate | 1996 | 145.8413891 | 234.9291714 | 54.77405824 |
| Deaths | Middle SDI | Both | 65+ years | diarrhea diseases | Unsafe water source | Percent | 1996 | 1.329069732 | 1.760785019 | 0.599158016 |
| Deaths | Middle SDI | Both | 65+ years | diarrhea diseases | Unsafe water source | Number | 1996 | 76679.98033 | 123388.9382 | 28870.57259 |
| DALYs (Disability-Adjusted Life Years) | Middle SDI | Both | 65+ years | diarrhea diseases | Unsafe water source | Rate | 1996 | 2431.502609 | 3884.792262 | 928.6223507 |
| DALYs (Disability-Adjusted Life Years) | Middle SDI | Both | 65+ years | diarrhea diseases | Unsafe water source | Percent | 1996 | 1.328183753 | 1.760450666 | 0.59848736 |
| DALYs (Disability-Adjusted Life Years) | Middle SDI | Both | 65+ years | diarrhea diseases | Unsafe water source | Number | 1996 | 1232077.908 | 1965963.164 | 472099.5867 |
| Deaths | Low-middle SDI | Both | 65+ years | diarrhea diseases | Unsafe water source | Rate | 1996 | 1129.903338 | 1802.971315 | 531.9019023 |
| Deaths | Low-middle SDI | Both | 65+ years | diarrhea diseases | Unsafe water source | Percent | 1996 | 1.488774643 | 1.835090246 | 0.848201541 |
| Deaths | Low-middle SDI | Both | 65+ years | diarrhea diseases | Unsafe water source | Number | 1996 | 329524.8438 | 523644.8736 | 155493.5185 |
| DALYs (Disability-Adjusted Life Years) | Low-middle SDI | Both | 65+ years | diarrhea diseases | Unsafe water source | Rate | 1996 | 19047.94677 | 30590.26643 | 8998.362833 |
| DALYs (Disability-Adjusted Life Years) | Low-middle SDI | Both | 65+ years | diarrhea diseases | Unsafe water source | Percent | 1996 | 1.488422312 | 1.836179138 | 0.847712481 |
| DALYs (Disability-Adjusted Life Years) | Low-middle SDI | Both | 65+ years | diarrhea diseases | Unsafe water source | Number | 1996 | 5379847.536 | 8596639.339 | 2550173.081 |
| Deaths | Low SDI | Both | 65+ years | diarrhea diseases | Unsafe water source | Rate | 1996 | 1361.977075 | 2152.660939 | 655.1149824 |
| Deaths | Low SDI | Both | 65+ years | diarrhea diseases | Unsafe water source | Percent | 1996 | 1.541968555 | 1.862544919 | 0.930779651 |
| Deaths | Low SDI | Both | 65+ years | diarrhea diseases | Unsafe water source | Number | 1996 | 137183.058 | 216362.9146 | 66129.95497 |
| DALYs (Disability-Adjusted Life Years) | Low SDI | Both | 65+ years | diarrhea diseases | Unsafe water source | Rate | 1996 | 23835.7147 | 37649.9345 | 11500.06039 |
| DALYs (Disability-Adjusted Life Years) | Low SDI | Both | 65+ years | diarrhea diseases | Unsafe water source | Percent | 1996 | 1.542176718 | 1.863121566 | 0.930676245 |
| DALYs (Disability-Adjusted Life Years) | Low SDI | Both | 65+ years | diarrhea diseases | Unsafe water source | Number | 1996 | 2329459.766 | 3669440.645 | 1126267.235 |
| Deaths | High-middle SDI | Both | 65+ years | diarrhea diseases | Unsafe water source | Rate | 1996 | 6.915636688 | 11.80280692 | 2.296128 |
| Deaths | High-middle SDI | Both | 65+ years | diarrhea diseases | Unsafe water source | Percent | 1996 | 1.141127249 | 1.618612139 | 0.477630566 |
| Deaths | High-middle SDI | Both | 65+ years | diarrhea diseases | Unsafe water source | Number | 1996 | 3884.378396 | 6631.566193 | 1289.679571 |
| DALYs (Disability-Adjusted Life Years) | High-middle SDI | Both | 65+ years | diarrhea diseases | Unsafe water source | Rate | 1996 | 126.981925 | 214.5047868 | 43.4402964 |
| DALYs (Disability-Adjusted Life Years) | High-middle SDI | Both | 65+ years | diarrhea diseases | Unsafe water source | Percent | 1996 | 1.105038379 | 1.58029418 | 0.463127348 |
| DALYs (Disability-Adjusted Life Years) | High-middle SDI | Both | 65+ years | diarrhea diseases | Unsafe water source | Number | 1996 | 67842.84321 | 114860.2927 | 23183.75924 |
| Deaths | High SDI | Both | 65+ years | diarrhea diseases | Unsafe water source | Rate | 1996 | 1.014008538 | 2.267827503 | 0.230284946 |
| Deaths | High SDI | Both | 65+ years | diarrhea diseases | Unsafe water source | Percent | 1996 | 0.303953426 | 0.640927878 | 0.076981949 |
| Deaths | High SDI | Both | 65+ years | diarrhea diseases | Unsafe water source | Number | 1996 | 743.809342 | 1675.89079 | 167.0956447 |
| DALYs (Disability-Adjusted Life Years) | High SDI | Both | 65+ years | diarrhea diseases | Unsafe water source | Rate | 1996 | 20.98153928 | 45.71803052 | 4.908431293 |
| DALYs (Disability-Adjusted Life Years) | High SDI | Both | 65+ years | diarrhea diseases | Unsafe water source | Percent | 1996 | 0.273220047 | 0.583721855 | 0.068771822 |
| DALYs (Disability-Adjusted Life Years) | High SDI | Both | 65+ years | diarrhea diseases | Unsafe water source | Number | 1996 | 13977.503 | 30602.89039 | 3258.976496 |
| Deaths | Global | Both | 65+ years | diarrhea diseases | Unsafe water source | Rate | 1996 | 254.6359315 | 405.760434 | 113.2240885 |
| Deaths | Global | Both | 65+ years | diarrhea diseases | Unsafe water source | Percent | 1996 | 1.468664443 | 1.828651168 | 0.833483333 |
| Deaths | Global | Both | 65+ years | diarrhea diseases | Unsafe water source | Number | 1996 | 548176.5874 | 867992.2029 | 243321.3153 |
| DALYs (Disability-Adjusted Life Years) | Global | Both | 65+ years | diarrhea diseases | Unsafe water source | Rate | 1996 | 4407.205923 | 7045.206662 | 1985.112418 |
| DALYs (Disability-Adjusted Life Years) | Global | Both | 65+ years | diarrhea diseases | Unsafe water source | Percent | 1996 | 1.464953714 | 1.824420645 | 0.831176946 |
| DALYs (Disability-Adjusted Life Years) | Global | Both | 65+ years | diarrhea diseases | Unsafe water source | Number | 1996 | 9025856.413 | 14319240.1 | 4056303.185 |
| Deaths | Middle SDI | Both | 65+ years | diarrhea diseases | Unsafe water source | Rate | 1995 | 155.103439 | 251.237342 | 59.24160819 |
| Deaths | Middle SDI | Both | 65+ years | diarrhea diseases | Unsafe water source | Percent | 1995 | 1.333664836 | 1.765637807 | 0.610395814 |
| Deaths | Middle SDI | Both | 65+ years | diarrhea diseases | Unsafe water source | Number | 1995 | 78910.97086 | 127712.3551 | 30225.97426 |
| DALYs (Disability-Adjusted Life Years) | Middle SDI | Both | 65+ years | diarrhea diseases | Unsafe water source | Rate | 1995 | 2575.61931 | 4150.163543 | 994.4420106 |
| DALYs (Disability-Adjusted Life Years) | Middle SDI | Both | 65+ years | diarrhea diseases | Unsafe water source | Percent | 1995 | 1.332859397 | 1.765940593 | 0.60990581 |
| DALYs (Disability-Adjusted Life Years) | Middle SDI | Both | 65+ years | diarrhea diseases | Unsafe water source | Number | 1995 | 1264038.838 | 2035863.966 | 490251.6834 |
| Deaths | Low-middle SDI | Both | 65+ years | diarrhea diseases | Unsafe water source | Rate | 1995 | 1181.219295 | 1865.143214 | 538.3432835 |
| Deaths | Low-middle SDI | Both | 65+ years | diarrhea diseases | Unsafe water source | Percent | 1995 | 1.491293708 | 1.840088865 | 0.855968066 |
| Deaths | Low-middle SDI | Both | 65+ years | diarrhea diseases | Unsafe water source | Number | 1995 | 334672.1066 | 526113.99 | 152278.0448 |
| DALYs (Disability-Adjusted Life Years) | Low-middle SDI | Both | 65+ years | diarrhea diseases | Unsafe water source | Rate | 1995 | 19891.21793 | 31636.04641 | 9117.678294 |
| DALYs (Disability-Adjusted Life Years) | Low-middle SDI | Both | 65+ years | diarrhea diseases | Unsafe water source | Percent | 1995 | 1.490937499 | 1.839726338 | 0.855558854 |
| DALYs (Disability-Adjusted Life Years) | Low-middle SDI | Both | 65+ years | diarrhea diseases | Unsafe water source | Number | 1995 | 5456975.266 | 8632396.565 | 2495480.44 |
| Deaths | Low SDI | Both | 65+ years | diarrhea diseases | Unsafe water source | Rate | 1995 | 1414.960012 | 2225.386485 | 686.6152617 |
| Deaths | Low SDI | Both | 65+ years | diarrhea diseases | Unsafe water source | Percent | 1995 | 1.542612944 | 1.859891446 | 0.933747839 |
| Deaths | Low SDI | Both | 65+ years | diarrhea diseases | Unsafe water source | Number | 1995 | 139584.8136 | 218985.8419 | 67812.23645 |
| DALYs (Disability-Adjusted Life Years) | Low SDI | Both | 65+ years | diarrhea diseases | Unsafe water source | Rate | 1995 | 24615.40746 | 38714.6834 | 11993.78001 |
| DALYs (Disability-Adjusted Life Years) | Low SDI | Both | 65+ years | diarrhea diseases | Unsafe water source | Percent | 1995 | 1.54280746 | 1.859288419 | 0.93403973 |
| DALYs (Disability-Adjusted Life Years) | Low SDI | Both | 65+ years | diarrhea diseases | Unsafe water source | Number | 1995 | 2355343.6 | 3693042.177 | 1148735.994 |
| Deaths | High-middle SDI | Both | 65+ years | diarrhea diseases | Unsafe water source | Rate | 1995 | 7.448397988 | 12.46722368 | 2.414470397 |
| Deaths | High-middle SDI | Both | 65+ years | diarrhea diseases | Unsafe water source | Percent | 1995 | 1.143939313 | 1.621377454 | 0.479393316 |
| Deaths | High-middle SDI | Both | 65+ years | diarrhea diseases | Unsafe water source | Number | 1995 | 4053.009292 | 6778.903394 | 1313.733551 |
| DALYs (Disability-Adjusted Life Years) | High-middle SDI | Both | 65+ years | diarrhea diseases | Unsafe water source | Rate | 1995 | 136.0837994 | 224.0509092 | 46.18887636 |
| DALYs (Disability-Adjusted Life Years) | High-middle SDI | Both | 65+ years | diarrhea diseases | Unsafe water source | Percent | 1995 | 1.112666993 | 1.587924973 | 0.46760981 |
| DALYs (Disability-Adjusted Life Years) | High-middle SDI | Both | 65+ years | diarrhea diseases | Unsafe water source | Number | 1995 | 70594.34014 | 116347.115 | 23940.90312 |
| Deaths | High SDI | Both | 65+ years | diarrhea diseases | Unsafe water source | Rate | 1995 | 1.047879293 | 2.320688161 | 0.243715931 |
| Deaths | High SDI | Both | 65+ years | diarrhea diseases | Unsafe water source | Percent | 1995 | 0.316347133 | 0.662938355 | 0.081606903 |
| Deaths | High SDI | Both | 65+ years | diarrhea diseases | Unsafe water source | Number | 1995 | 751.9778414 | 1677.119321 | 173.2996914 |
| DALYs (Disability-Adjusted Life Years) | High SDI | Both | 65+ years | diarrhea diseases | Unsafe water source | Rate | 1995 | 21.46920954 | 46.09908135 | 5.130548356 |
| DALYs (Disability-Adjusted Life Years) | High SDI | Both | 65+ years | diarrhea diseases | Unsafe water source | Percent | 1995 | 0.286266889 | 0.609320103 | 0.074032555 |
| DALYs (Disability-Adjusted Life Years) | High SDI | Both | 65+ years | diarrhea diseases | Unsafe water source | Number | 1995 | 14015.22915 | 30327.85388 | 3333.031127 |
| Deaths | Global | Both | 65+ years | diarrhea diseases | Unsafe water source | Rate | 1995 | 266.1282041 | 423.1387766 | 118.176936 |
| Deaths | Global | Both | 65+ years | diarrhea diseases | Unsafe water source | Percent | 1995 | 1.471109631 | 1.82466327 | 0.842431369 |
| Deaths | Global | Both | 65+ years | diarrhea diseases | Unsafe water source | Number | 1995 | 558138.0472 | 882632.5771 | 247616.5748 |
| DALYs (Disability-Adjusted Life Years) | Global | Both | 65+ years | diarrhea diseases | Unsafe water source | Rate | 1995 | 4587.346532 | 7316.795651 | 2044.883552 |
| DALYs (Disability-Adjusted Life Years) | Global | Both | 65+ years | diarrhea diseases | Unsafe water source | Percent | 1995 | 1.467680783 | 1.820316139 | 0.839976808 |
| DALYs (Disability-Adjusted Life Years) | Global | Both | 65+ years | diarrhea diseases | Unsafe water source | Number | 1995 | 9163696.412 | 14518939.61 | 4078775.216 |
| Deaths | Middle SDI | Both | 65+ years | diarrhea diseases | Unsafe water source | Rate | 1994 | 163.056705 | 262.6562281 | 62.05963855 |
| Deaths | Middle SDI | Both | 65+ years | diarrhea diseases | Unsafe water source | Percent | 1994 | 1.339527023 | 1.766746041 | 0.626803715 |
| Deaths | Middle SDI | Both | 65+ years | diarrhea diseases | Unsafe water source | Number | 1994 | 80150.47514 | 128956.4382 | 30576.98623 |
| DALYs (Disability-Adjusted Life Years) | Middle SDI | Both | 65+ years | diarrhea diseases | Unsafe water source | Rate | 1994 | 2710.608799 | 4339.313303 | 1046.840891 |
| DALYs (Disability-Adjusted Life Years) | Middle SDI | Both | 65+ years | diarrhea diseases | Unsafe water source | Percent | 1994 | 1.338744873 | 1.765804399 | 0.624914797 |
| DALYs (Disability-Adjusted Life Years) | Middle SDI | Both | 65+ years | diarrhea diseases | Unsafe water source | Number | 1994 | 1285969.22 | 2055961.022 | 498184.4417 |
| Deaths | Low-middle SDI | Both | 65+ years | diarrhea diseases | Unsafe water source | Rate | 1994 | 1205.379439 | 1917.502738 | 550.3958378 |
| Deaths | Low-middle SDI | Both | 65+ years | diarrhea diseases | Unsafe water source | Percent | 1994 | 1.493821092 | 1.837721242 | 0.857488667 |
| Deaths | Low-middle SDI | Both | 65+ years | diarrhea diseases | Unsafe water source | Number | 1994 | 330963.055 | 524241.4214 | 151207.3285 |
| DALYs (Disability-Adjusted Life Years) | Low-middle SDI | Both | 65+ years | diarrhea diseases | Unsafe water source | Rate | 1994 | 20455.94878 | 32752.24702 | 9284.861973 |
| DALYs (Disability-Adjusted Life Years) | Low-middle SDI | Both | 65+ years | diarrhea diseases | Unsafe water source | Percent | 1994 | 1.493514756 | 1.837813335 | 0.856995622 |
| DALYs (Disability-Adjusted Life Years) | Low-middle SDI | Both | 65+ years | diarrhea diseases | Unsafe water source | Number | 1994 | 5434459.772 | 8656520.514 | 2467570.423 |
| Deaths | Low SDI | Both | 65+ years | diarrhea diseases | Unsafe water source | Rate | 1994 | 1441.332661 | 2268.088615 | 698.5627654 |
| Deaths | Low SDI | Both | 65+ years | diarrhea diseases | Unsafe water source | Percent | 1994 | 1.543371658 | 1.862015099 | 0.934117876 |
| Deaths | Low SDI | Both | 65+ years | diarrhea diseases | Unsafe water source | Number | 1994 | 139110.9933 | 218382.029 | 67573.67422 |
| DALYs (Disability-Adjusted Life Years) | Low SDI | Both | 65+ years | diarrhea diseases | Unsafe water source | Rate | 1994 | 25118.61636 | 39465.11281 | 12208.18538 |
| DALYs (Disability-Adjusted Life Years) | Low SDI | Both | 65+ years | diarrhea diseases | Unsafe water source | Percent | 1994 | 1.543520486 | 1.861748372 | 0.933990414 |
| DALYs (Disability-Adjusted Life Years) | Low SDI | Both | 65+ years | diarrhea diseases | Unsafe water source | Number | 1994 | 2351110.578 | 3682399.561 | 1145054.717 |
| Deaths | High-middle SDI | Both | 65+ years | diarrhea diseases | Unsafe water source | Rate | 1994 | 7.876741717 | 13.34191397 | 2.617039467 |
| Deaths | High-middle SDI | Both | 65+ years | diarrhea diseases | Unsafe water source | Percent | 1994 | 1.156408702 | 1.624427508 | 0.498871738 |
| Deaths | High-middle SDI | Both | 65+ years | diarrhea diseases | Unsafe water source | Number | 1994 | 4157.668007 | 7052.43602 | 1380.001709 |
| DALYs (Disability-Adjusted Life Years) | High-middle SDI | Both | 65+ years | diarrhea diseases | Unsafe water source | Rate | 1994 | 143.543483 | 240.5856909 | 49.64568212 |
| DALYs (Disability-Adjusted Life Years) | High-middle SDI | Both | 65+ years | diarrhea diseases | Unsafe water source | Percent | 1994 | 1.127145364 | 1.593837323 | 0.486924771 |
| DALYs (Disability-Adjusted Life Years) | High-middle SDI | Both | 65+ years | diarrhea diseases | Unsafe water source | Number | 1994 | 72331.38055 | 121572.9439 | 25044.51757 |
| Deaths | High SDI | Both | 65+ years | diarrhea diseases | Unsafe water source | Rate | 1994 | 1.058346187 | 2.314218313 | 0.251086179 |
| Deaths | High SDI | Both | 65+ years | diarrhea diseases | Unsafe water source | Percent | 1994 | 0.326900711 | 0.679497998 | 0.086094138 |
| Deaths | High SDI | Both | 65+ years | diarrhea diseases | Unsafe water source | Number | 1994 | 741.3224463 | 1629.696034 | 174.332696 |
| DALYs (Disability-Adjusted Life Years) | High SDI | Both | 65+ years | diarrhea diseases | Unsafe water source | Rate | 1994 | 21.77025033 | 46.27102813 | 5.307006831 |
| DALYs (Disability-Adjusted Life Years) | High SDI | Both | 65+ years | diarrhea diseases | Unsafe water source | Percent | 1994 | 0.298382858 | 0.630409714 | 0.078794059 |
| DALYs (Disability-Adjusted Life Years) | High SDI | Both | 65+ years | diarrhea diseases | Unsafe water source | Number | 1994 | 13893.96756 | 29768.07064 | 3376.388599 |
| Deaths | Global | Both | 65+ years | diarrhea diseases | Unsafe water source | Rate | 1994 | 272.7055648 | 431.0955365 | 121.1467902 |
| Deaths | Global | Both | 65+ years | diarrhea diseases | Unsafe water source | Percent | 1994 | 1.47357045 | 1.82625166 | 0.844173312 |
| Deaths | Global | Both | 65+ years | diarrhea diseases | Unsafe water source | Number | 1994 | 555292.767 | 871664.7774 | 246688.5416 |
| DALYs (Disability-Adjusted Life Years) | Global | Both | 65+ years | diarrhea diseases | Unsafe water source | Rate | 1994 | 4720.207053 | 7504.957867 | 2099.16803 |
| DALYs (Disability-Adjusted Life Years) | Global | Both | 65+ years | diarrhea diseases | Unsafe water source | Percent | 1994 | 1.470398217 | 1.823104204 | 0.842049264 |
| DALYs (Disability-Adjusted Life Years) | Global | Both | 65+ years | diarrhea diseases | Unsafe water source | Number | 1994 | 9160562.138 | 14439910.99 | 4071733.786 |
| Deaths | Middle SDI | Both | 65+ years | diarrhea diseases | Unsafe water source | Rate | 1993 | 173.9487894 | 279.5066802 | 66.29685696 |
| Deaths | Middle SDI | Both | 65+ years | diarrhea diseases | Unsafe water source | Percent | 1993 | 1.344872412 | 1.766150227 | 0.631967081 |
| Deaths | Middle SDI | Both | 65+ years | diarrhea diseases | Unsafe water source | Number | 1993 | 82673.17177 | 132823.8802 | 31584.09226 |
| DALYs (Disability-Adjusted Life Years) | Middle SDI | Both | 65+ years | diarrhea diseases | Unsafe water source | Rate | 1993 | 2887.786191 | 4587.832314 | 1123.381422 |
| DALYs (Disability-Adjusted Life Years) | Middle SDI | Both | 65+ years | diarrhea diseases | Unsafe water source | Percent | 1993 | 1.34418187 | 1.765400629 | 0.631744887 |
| DALYs (Disability-Adjusted Life Years) | Middle SDI | Both | 65+ years | diarrhea diseases | Unsafe water source | Number | 1993 | 1325143.305 | 2104990.808 | 517007.7016 |
| Deaths | Low-middle SDI | Both | 65+ years | diarrhea diseases | Unsafe water source | Rate | 1993 | 1254.837196 | 1963.14203 | 583.0954274 |
| Deaths | Low-middle SDI | Both | 65+ years | diarrhea diseases | Unsafe water source | Percent | 1993 | 1.496208308 | 1.843114771 | 0.865044905 |
| Deaths | Low-middle SDI | Both | 65+ years | diarrhea diseases | Unsafe water source | Number | 1993 | 334209.9457 | 519895.8181 | 155416.667 |
| DALYs (Disability-Adjusted Life Years) | Low-middle SDI | Both | 65+ years | diarrhea diseases | Unsafe water source | Rate | 1993 | 21331.34952 | 33786.20323 | 9862.646007 |
| DALYs (Disability-Adjusted Life Years) | Low-middle SDI | Both | 65+ years | diarrhea diseases | Unsafe water source | Percent | 1993 | 1.495899664 | 1.842812758 | 0.864533722 |
| DALYs (Disability-Adjusted Life Years) | Low-middle SDI | Both | 65+ years | diarrhea diseases | Unsafe water source | Number | 1993 | 5492969.062 | 8644030.567 | 2541010.583 |
| Deaths | Low SDI | Both | 65+ years | diarrhea diseases | Unsafe water source | Rate | 1993 | 1439.786549 | 2250.339777 | 710.6718709 |
| Deaths | Low SDI | Both | 65+ years | diarrhea diseases | Unsafe water source | Percent | 1993 | 1.544655124 | 1.864093369 | 0.933659918 |
| Deaths | Low SDI | Both | 65+ years | diarrhea diseases | Unsafe water source | Number | 1993 | 135793.5927 | 211611.7287 | 67167.94828 |
| DALYs (Disability-Adjusted Life Years) | Low SDI | Both | 65+ years | diarrhea diseases | Unsafe water source | Rate | 1993 | 25233.82019 | 39593.79141 | 12420.04134 |
| DALYs (Disability-Adjusted Life Years) | Low SDI | Both | 65+ years | diarrhea diseases | Unsafe water source | Percent | 1993 | 1.544810486 | 1.86342024 | 0.933661148 |
| DALYs (Disability-Adjusted Life Years) | Low SDI | Both | 65+ years | diarrhea diseases | Unsafe water source | Number | 1993 | 2309324.839 | 3610425.079 | 1139355.692 |
| Deaths | High-middle SDI | Both | 65+ years | diarrhea diseases | Unsafe water source | Rate | 1993 | 8.27437738 | 13.87275787 | 2.792161591 |
| Deaths | High-middle SDI | Both | 65+ years | diarrhea diseases | Unsafe water source | Percent | 1993 | 1.181941718 | 1.640128178 | 0.525930444 |
| Deaths | High-middle SDI | Both | 65+ years | diarrhea diseases | Unsafe water source | Number | 1993 | 4240.475891 | 7112.907372 | 1427.309701 |
| DALYs (Disability-Adjusted Life Years) | High-middle SDI | Both | 65+ years | diarrhea diseases | Unsafe water source | Rate | 1993 | 150.2402101 | 245.5441156 | 53.15173248 |
| DALYs (Disability-Adjusted Life Years) | High-middle SDI | Both | 65+ years | diarrhea diseases | Unsafe water source | Percent | 1993 | 1.151440027 | 1.607870179 | 0.512564649 |
| DALYs (Disability-Adjusted Life Years) | High-middle SDI | Both | 65+ years | diarrhea diseases | Unsafe water source | Number | 1993 | 73490.79626 | 120412.2437 | 26011.03782 |
| Deaths | High SDI | Both | 65+ years | diarrhea diseases | Unsafe water source | Rate | 1993 | 1.076849837 | 2.338413703 | 0.257093258 |
| Deaths | High SDI | Both | 65+ years | diarrhea diseases | Unsafe water source | Percent | 1993 | 0.335313007 | 0.695754525 | 0.090012986 |
| Deaths | High SDI | Both | 65+ years | diarrhea diseases | Unsafe water source | Number | 1993 | 737.8224501 | 1610.984276 | 174.7224628 |
| DALYs (Disability-Adjusted Life Years) | High SDI | Both | 65+ years | diarrhea diseases | Unsafe water source | Rate | 1993 | 22.18608052 | 47.10869762 | 5.481617884 |
| DALYs (Disability-Adjusted Life Years) | High SDI | Both | 65+ years | diarrhea diseases | Unsafe water source | Percent | 1993 | 0.309087734 | 0.650755937 | 0.084506549 |
| DALYs (Disability-Adjusted Life Years) | High SDI | Both | 65+ years | diarrhea diseases | Unsafe water source | Number | 1993 | 13869.77262 | 29707.38045 | 3397.995697 |
| Deaths | Global | Both | 65+ years | diarrhea diseases | Unsafe water source | Rate | 1993 | 281.7094682 | 438.7254587 | 127.3576613 |
| Deaths | Global | Both | 65+ years | diarrhea diseases | Unsafe water source | Percent | 1993 | 1.475758409 | 1.829024282 | 0.849173419 |
| Deaths | Global | Both | 65+ years | diarrhea diseases | Unsafe water source | Number | 1993 | 557827.219 | 862815.7423 | 252677.6867 |
| DALYs (Disability-Adjusted Life Years) | Global | Both | 65+ years | diarrhea diseases | Unsafe water source | Rate | 1993 | 4882.23156 | 7646.176122 | 2201.510162 |
| DALYs (Disability-Adjusted Life Years) | Global | Both | 65+ years | diarrhea diseases | Unsafe water source | Percent | 1993 | 1.472896208 | 1.825751385 | 0.847653182 |
| DALYs (Disability-Adjusted Life Years) | Global | Both | 65+ years | diarrhea diseases | Unsafe water source | Number | 1993 | 9217643.999 | 14317640.39 | 4164046.395 |
| Deaths | Middle SDI | Both | 65+ years | diarrhea diseases | Unsafe water source | Rate | 1992 | 185.8030606 | 302.4983324 | 70.37025146 |
| Deaths | Middle SDI | Both | 65+ years | diarrhea diseases | Unsafe water source | Percent | 1992 | 1.350393948 | 1.771900131 | 0.643202377 |
| Deaths | Middle SDI | Both | 65+ years | diarrhea diseases | Unsafe water source | Number | 1992 | 85400.54881 | 139071.7242 | 32467.15088 |
| DALYs (Disability-Adjusted Life Years) | Middle SDI | Both | 65+ years | diarrhea diseases | Unsafe water source | Rate | 1992 | 3079.824541 | 4989.935512 | 1195.576326 |
| DALYs (Disability-Adjusted Life Years) | Middle SDI | Both | 65+ years | diarrhea diseases | Unsafe water source | Percent | 1992 | 1.349835971 | 1.772047657 | 0.642432895 |
| DALYs (Disability-Adjusted Life Years) | Middle SDI | Both | 65+ years | diarrhea diseases | Unsafe water source | Number | 1992 | 1367231.603 | 2217514.031 | 532921.4766 |
| Deaths | Low-middle SDI | Both | 65+ years | diarrhea diseases | Unsafe water source | Rate | 1992 | 1311.704319 | 2048.470964 | 613.7469543 |
| Deaths | Low-middle SDI | Both | 65+ years | diarrhea diseases | Unsafe water source | Percent | 1992 | 1.498218677 | 1.840915132 | 0.864971092 |
| Deaths | Low-middle SDI | Both | 65+ years | diarrhea diseases | Unsafe water source | Number | 1992 | 339088.4826 | 527344.267 | 158792.4009 |
| DALYs (Disability-Adjusted Life Years) | Low-middle SDI | Both | 65+ years | diarrhea diseases | Unsafe water source | Rate | 1992 | 22336.66169 | 35200.34281 | 10403.22914 |
| DALYs (Disability-Adjusted Life Years) | Low-middle SDI | Both | 65+ years | diarrhea diseases | Unsafe water source | Percent | 1992 | 1.497947966 | 1.841025077 | 0.864637111 |
| DALYs (Disability-Adjusted Life Years) | Low-middle SDI | Both | 65+ years | diarrhea diseases | Unsafe water source | Number | 1992 | 5580461.996 | 8752895.274 | 2600857.195 |
| Deaths | Low SDI | Both | 65+ years | diarrhea diseases | Unsafe water source | Rate | 1992 | 1468.207444 | 2294.361369 | 730.0247426 |
| Deaths | Low SDI | Both | 65+ years | diarrhea diseases | Unsafe water source | Percent | 1992 | 1.545199584 | 1.86449889 | 0.937321897 |
| Deaths | Low SDI | Both | 65+ years | diarrhea diseases | Unsafe water source | Number | 1992 | 135089.9833 | 210421.3536 | 67437.20902 |
| DALYs (Disability-Adjusted Life Years) | Low SDI | Both | 65+ years | diarrhea diseases | Unsafe water source | Rate | 1992 | 25783.6743 | 40531.62399 | 12740.96742 |
| DALYs (Disability-Adjusted Life Years) | Low SDI | Both | 65+ years | diarrhea diseases | Unsafe water source | Percent | 1992 | 1.545303366 | 1.864287216 | 0.937506586 |
| DALYs (Disability-Adjusted Life Years) | Low SDI | Both | 65+ years | diarrhea diseases | Unsafe water source | Number | 1992 | 2303505.788 | 3607487.931 | 1143130.765 |
| Deaths | High-middle SDI | Both | 65+ years | diarrhea diseases | Unsafe water source | Rate | 1992 | 8.793992706 | 14.74742099 | 3.035687648 |
| Deaths | High-middle SDI | Both | 65+ years | diarrhea diseases | Unsafe water source | Percent | 1992 | 1.210499001 | 1.666287278 | 0.546753225 |
| Deaths | High-middle SDI | Both | 65+ years | diarrhea diseases | Unsafe water source | Number | 1992 | 4381.396531 | 7359.116188 | 1513.561532 |
| DALYs (Disability-Adjusted Life Years) | High-middle SDI | Both | 65+ years | diarrhea diseases | Unsafe water source | Rate | 1992 | 158.8914739 | 260.32119 | 57.68606843 |
| DALYs (Disability-Adjusted Life Years) | High-middle SDI | Both | 65+ years | diarrhea diseases | Unsafe water source | Percent | 1992 | 1.178918974 | 1.629964594 | 0.528687578 |
| DALYs (Disability-Adjusted Life Years) | High-middle SDI | Both | 65+ years | diarrhea diseases | Unsafe water source | Number | 1992 | 75496.06113 | 123848.8381 | 27413.12023 |
| Deaths | High SDI | Both | 65+ years | diarrhea diseases | Unsafe water source | Rate | 1992 | 1.111159484 | 2.405944411 | 0.270150943 |
| Deaths | High SDI | Both | 65+ years | diarrhea diseases | Unsafe water source | Percent | 1992 | 0.347033407 | 0.715001837 | 0.095862748 |
| Deaths | High SDI | Both | 65+ years | diarrhea diseases | Unsafe water source | Number | 1992 | 744.9795269 | 1621.406731 | 179.4643778 |
| DALYs (Disability-Adjusted Life Years) | High SDI | Both | 65+ years | diarrhea diseases | Unsafe water source | Rate | 1992 | 22.84184546 | 48.72726321 | 5.795501777 |
| DALYs (Disability-Adjusted Life Years) | High SDI | Both | 65+ years | diarrhea diseases | Unsafe water source | Percent | 1992 | 0.321384959 | 0.671083369 | 0.089806249 |
| DALYs (Disability-Adjusted Life Years) | High SDI | Both | 65+ years | diarrhea diseases | Unsafe water source | Number | 1992 | 13992.0331 | 30068.11502 | 3523.895422 |
| Deaths | Global | Both | 65+ years | diarrhea diseases | Unsafe water source | Rate | 1992 | 293.3353378 | 458.7995747 | 133.3612616 |
| Deaths | Global | Both | 65+ years | diarrhea diseases | Unsafe water source | Percent | 1992 | 1.477910751 | 1.828575649 | 0.852497512 |
| Deaths | Global | Both | 65+ years | diarrhea diseases | Unsafe water source | Number | 1992 | 564878.0604 | 878868.0292 | 257434.8728 |
| DALYs (Disability-Adjusted Life Years) | Global | Both | 65+ years | diarrhea diseases | Unsafe water source | Rate | 1992 | 5088.096166 | 7984.496111 | 2307.632848 |
| DALYs (Disability-Adjusted Life Years) | Global | Both | 65+ years | diarrhea diseases | Unsafe water source | Percent | 1992 | 1.475320853 | 1.825029254 | 0.851209304 |
| DALYs (Disability-Adjusted Life Years) | Global | Both | 65+ years | diarrhea diseases | Unsafe water source | Number | 1992 | 9343546.892 | 14574500.7 | 4248513.486 |
| Deaths | Middle SDI | Both | 65+ years | diarrhea diseases | Unsafe water source | Rate | 1991 | 196.1971155 | 314.9786686 | 76.26302624 |
| Deaths | Middle SDI | Both | 65+ years | diarrhea diseases | Unsafe water source | Percent | 1991 | 1.357556272 | 1.77615119 | 0.663322754 |
| Deaths | Middle SDI | Both | 65+ years | diarrhea diseases | Unsafe water source | Number | 1991 | 87140.62827 | 139719.1264 | 33992.0053 |
| DALYs (Disability-Adjusted Life Years) | Middle SDI | Both | 65+ years | diarrhea diseases | Unsafe water source | Rate | 1991 | 3257.001142 | 5209.879984 | 1277.877402 |
| DALYs (Disability-Adjusted Life Years) | Middle SDI | Both | 65+ years | diarrhea diseases | Unsafe water source | Percent | 1991 | 1.357074994 | 1.775997079 | 0.663234795 |
| DALYs (Disability-Adjusted Life Years) | Middle SDI | Both | 65+ years | diarrhea diseases | Unsafe water source | Number | 1991 | 1397457.321 | 2231292.34 | 551236.8513 |
| Deaths | Low-middle SDI | Both | 65+ years | diarrhea diseases | Unsafe water source | Rate | 1991 | 1326.469456 | 2067.446437 | 628.5961226 |
| Deaths | Low-middle SDI | Both | 65+ years | diarrhea diseases | Unsafe water source | Percent | 1991 | 1.500032092 | 1.842704827 | 0.873191098 |
| Deaths | Low-middle SDI | Both | 65+ years | diarrhea diseases | Unsafe water source | Number | 1991 | 332687.0653 | 515852.835 | 157737.1113 |
| DALYs (Disability-Adjusted Life Years) | Low-middle SDI | Both | 65+ years | diarrhea diseases | Unsafe water source | Rate | 1991 | 22708.96852 | 35838.30986 | 10710.9896 |
| DALYs (Disability-Adjusted Life Years) | Low-middle SDI | Both | 65+ years | diarrhea diseases | Unsafe water source | Percent | 1991 | 1.49980779 | 1.84247183 | 0.872728285 |
| DALYs (Disability-Adjusted Life Years) | Low-middle SDI | Both | 65+ years | diarrhea diseases | Unsafe water source | Number | 1991 | 5502386.772 | 8635375.817 | 2595792.066 |
| Deaths | Low SDI | Both | 65+ years | diarrhea diseases | Unsafe water source | Rate | 1991 | 1481.002973 | 2313.429942 | 738.6813591 |
| Deaths | Low SDI | Both | 65+ years | diarrhea diseases | Unsafe water source | Percent | 1991 | 1.545994993 | 1.861132481 | 0.939718328 |
| Deaths | Low SDI | Both | 65+ years | diarrhea diseases | Unsafe water source | Number | 1991 | 132784.6004 | 207000.2649 | 66417.90384 |
| DALYs (Disability-Adjusted Life Years) | Low SDI | Both | 65+ years | diarrhea diseases | Unsafe water source | Rate | 1991 | 26104.32611 | 40902.99522 | 12935.84722 |
| DALYs (Disability-Adjusted Life Years) | Low SDI | Both | 65+ years | diarrhea diseases | Unsafe water source | Percent | 1991 | 1.546102423 | 1.860954534 | 0.939771445 |
| DALYs (Disability-Adjusted Life Years) | Low SDI | Both | 65+ years | diarrhea diseases | Unsafe water source | Number | 1991 | 2274264.443 | 3555276.304 | 1130563.329 |
| Deaths | High-middle SDI | Both | 65+ years | diarrhea diseases | Unsafe water source | Rate | 1991 | 9.323685209 | 15.58841969 | 3.159785757 |
| Deaths | High-middle SDI | Both | 65+ years | diarrhea diseases | Unsafe water source | Percent | 1991 | 1.232753032 | 1.682035117 | 0.566174995 |
| Deaths | High-middle SDI | Both | 65+ years | diarrhea diseases | Unsafe water source | Number | 1991 | 4513.399887 | 7550.317265 | 1529.879055 |
| DALYs (Disability-Adjusted Life Years) | High-middle SDI | Both | 65+ years | diarrhea diseases | Unsafe water source | Rate | 1991 | 168.4203529 | 273.1735145 | 60.48860598 |
| DALYs (Disability-Adjusted Life Years) | High-middle SDI | Both | 65+ years | diarrhea diseases | Unsafe water source | Percent | 1991 | 1.201120563 | 1.644297572 | 0.548132477 |
| DALYs (Disability-Adjusted Life Years) | High-middle SDI | Both | 65+ years | diarrhea diseases | Unsafe water source | Number | 1991 | 77579.10295 | 125938.6121 | 27811.56811 |
| Deaths | High SDI | Both | 65+ years | diarrhea diseases | Unsafe water source | Rate | 1991 | 1.145744027 | 2.468857453 | 0.284116555 |
| Deaths | High SDI | Both | 65+ years | diarrhea diseases | Unsafe water source | Percent | 1991 | 0.368227156 | 0.747256279 | 0.104214816 |
| Deaths | High SDI | Both | 65+ years | diarrhea diseases | Unsafe water source | Number | 1991 | 749.5384848 | 1623.947051 | 184.2974943 |
| DALYs (Disability-Adjusted Life Years) | High SDI | Both | 65+ years | diarrhea diseases | Unsafe water source | Rate | 1991 | 23.62159951 | 50.00474873 | 6.010030528 |
| DALYs (Disability-Adjusted Life Years) | High SDI | Both | 65+ years | diarrhea diseases | Unsafe water source | Percent | 1991 | 0.338902496 | 0.706393156 | 0.096202615 |
| DALYs (Disability-Adjusted Life Years) | High SDI | Both | 65+ years | diarrhea diseases | Unsafe water source | Number | 1991 | 14142.94027 | 30129.6721 | 3575.920765 |
| Deaths | Global | Both | 65+ years | diarrhea diseases | Unsafe water source | Rate | 1991 | 298.4056013 | 471.0197365 | 137.9829748 |
| Deaths | Global | Both | 65+ years | diarrhea diseases | Unsafe water source | Percent | 1991 | 1.480185629 | 1.825104305 | 0.860232441 |
| Deaths | Global | Both | 65+ years | diarrhea diseases | Unsafe water source | Number | 1991 | 558049.2822 | 876042.135 | 258669.7895 |
| DALYs (Disability-Adjusted Life Years) | Global | Both | 65+ years | diarrhea diseases | Unsafe water source | Rate | 1991 | 5198.389791 | 8245.515016 | 2398.835706 |
| DALYs (Disability-Adjusted Life Years) | Global | Both | 65+ years | diarrhea diseases | Unsafe water source | Percent | 1991 | 1.477759933 | 1.82160799 | 0.859054413 |
| DALYs (Disability-Adjusted Life Years) | Global | Both | 65+ years | diarrhea diseases | Unsafe water source | Number | 1991 | 9268715.486 | 14612750.65 | 4285786.362 |
| Deaths | Middle SDI | Both | 65+ years | diarrhea diseases | Unsafe water source | Rate | 1990 | 207.8878899 | 336.7428665 | 80.88770471 |
| Deaths | Middle SDI | Both | 65+ years | diarrhea diseases | Unsafe water source | Percent | 1990 | 1.365142457 | 1.778440852 | 0.669572001 |
| Deaths | Middle SDI | Both | 65+ years | diarrhea diseases | Unsafe water source | Number | 1990 | 89233.20047 | 144192.0114 | 34870.56119 |
| DALYs (Disability-Adjusted Life Years) | Middle SDI | Both | 65+ years | diarrhea diseases | Unsafe water source | Rate | 1990 | 3455.354672 | 5565.589066 | 1350.797095 |
| DALYs (Disability-Adjusted Life Years) | Middle SDI | Both | 65+ years | diarrhea diseases | Unsafe water source | Percent | 1990 | 1.364692938 | 1.77744091 | 0.669648589 |
| DALYs (Disability-Adjusted Life Years) | Middle SDI | Both | 65+ years | diarrhea diseases | Unsafe water source | Number | 1990 | 1432804.21 | 2301625.734 | 563229.3761 |
| Deaths | Low-middle SDI | Both | 65+ years | diarrhea diseases | Unsafe water source | Rate | 1990 | 1359.701829 | 2109.712499 | 646.9357484 |
| Deaths | Low-middle SDI | Both | 65+ years | diarrhea diseases | Unsafe water source | Percent | 1990 | 1.501814696 | 1.845302807 | 0.873735554 |
| Deaths | Low-middle SDI | Both | 65+ years | diarrhea diseases | Unsafe water source | Number | 1990 | 331261.6518 | 510869.354 | 158007.0992 |
| DALYs (Disability-Adjusted Life Years) | Low-middle SDI | Both | 65+ years | diarrhea diseases | Unsafe water source | Rate | 1990 | 23320.66884 | 36651.09815 | 11020.27391 |
| DALYs (Disability-Adjusted Life Years) | Low-middle SDI | Both | 65+ years | diarrhea diseases | Unsafe water source | Percent | 1990 | 1.501631799 | 1.84494644 | 0.873526565 |
| DALYs (Disability-Adjusted Life Years) | Low-middle SDI | Both | 65+ years | diarrhea diseases | Unsafe water source | Number | 1990 | 5489747.307 | 8568730.869 | 2601336.962 |
| Deaths | Low SDI | Both | 65+ years | diarrhea diseases | Unsafe water source | Rate | 1990 | 1486.761787 | 2312.709677 | 725.1745277 |
| Deaths | Low SDI | Both | 65+ years | diarrhea diseases | Unsafe water source | Percent | 1990 | 1.546360062 | 1.862654566 | 0.942586085 |
| Deaths | Low SDI | Both | 65+ years | diarrhea diseases | Unsafe water source | Number | 1990 | 129823.5861 | 201445.3525 | 63502.49713 |
| DALYs (Disability-Adjusted Life Years) | Low SDI | Both | 65+ years | diarrhea diseases | Unsafe water source | Rate | 1990 | 26337.04477 | 40938.39345 | 12798.41655 |
| DALYs (Disability-Adjusted Life Years) | Low SDI | Both | 65+ years | diarrhea diseases | Unsafe water source | Percent | 1990 | 1.54647552 | 1.861925227 | 0.943088158 |
| DALYs (Disability-Adjusted Life Years) | Low SDI | Both | 65+ years | diarrhea diseases | Unsafe water source | Number | 1990 | 2237649.599 | 3466981.313 | 1090309.426 |
| Deaths | High-middle SDI | Both | 65+ years | diarrhea diseases | Unsafe water source | Rate | 1990 | 9.926348374 | 16.56396022 | 3.409947074 |
| Deaths | High-middle SDI | Both | 65+ years | diarrhea diseases | Unsafe water source | Percent | 1990 | 1.247655747 | 1.687815258 | 0.583850097 |
| Deaths | High-middle SDI | Both | 65+ years | diarrhea diseases | Unsafe water source | Number | 1990 | 4675.419304 | 7808.335595 | 1606.937873 |
| DALYs (Disability-Adjusted Life Years) | High-middle SDI | Both | 65+ years | diarrhea diseases | Unsafe water source | Rate | 1990 | 179.149647 | 290.4387111 | 64.96872358 |
| DALYs (Disability-Adjusted Life Years) | High-middle SDI | Both | 65+ years | diarrhea diseases | Unsafe water source | Percent | 1990 | 1.217141578 | 1.656043199 | 0.566909107 |
| DALYs (Disability-Adjusted Life Years) | High-middle SDI | Both | 65+ years | diarrhea diseases | Unsafe water source | Number | 1990 | 80104.89736 | 130249.6033 | 29017.99712 |
| Deaths | High SDI | Both | 65+ years | diarrhea diseases | Unsafe water source | Rate | 1990 | 1.172904289 | 2.488741893 | 0.298603207 |
| Deaths | High SDI | Both | 65+ years | diarrhea diseases | Unsafe water source | Percent | 1990 | 0.388698977 | 0.775003041 | 0.112936793 |
| Deaths | High SDI | Both | 65+ years | diarrhea diseases | Unsafe water source | Number | 1990 | 749.8054205 | 1600.191457 | 189.0413339 |
| DALYs (Disability-Adjusted Life Years) | High SDI | Both | 65+ years | diarrhea diseases | Unsafe water source | Rate | 1990 | 24.27009383 | 51.03878097 | 6.308330225 |
| DALYs (Disability-Adjusted Life Years) | High SDI | Both | 65+ years | diarrhea diseases | Unsafe water source | Percent | 1990 | 0.354456205 | 0.7268126 | 0.10252824 |
| DALYs (Disability-Adjusted Life Years) | High SDI | Both | 65+ years | diarrhea diseases | Unsafe water source | Number | 1990 | 14210.30894 | 30039.04431 | 3667.778821 |
| Deaths | Global | Both | 65+ years | diarrhea diseases | Unsafe water source | Rate | 1990 | 305.7294286 | 476.614789 | 139.5613248 |
| Deaths | Global | Both | 65+ years | diarrhea diseases | Unsafe water source | Percent | 1990 | 1.482436235 | 1.825512762 | 0.863159434 |
| Deaths | Global | Both | 65+ years | diarrhea diseases | Unsafe water source | Number | 1990 | 555921.1381 | 861791.4838 | 254620.7586 |
| DALYs (Disability-Adjusted Life Years) | Global | Both | 65+ years | diarrhea diseases | Unsafe water source | Rate | 1990 | 5340.968667 | 8359.055646 | 2436.901286 |
| DALYs (Disability-Adjusted Life Years) | Global | Both | 65+ years | diarrhea diseases | Unsafe water source | Percent | 1990 | 1.4801222 | 1.822880876 | 0.861831376 |
| DALYs (Disability-Adjusted Life Years) | Global | Both | 65+ years | diarrhea diseases | Unsafe water source | Number | 1990 | 9257460.657 | 14396161.86 | 4240157.503 |
| Deaths | Middle SDI | Both | 65+ years | diarrhea diseases | Unsafe sanitation | Rate | 2021 | 21.84439862 | 33.56872722 | 11.31737374 |
| Deaths | Middle SDI | Both | 65+ years | diarrhea diseases | Unsafe sanitation | Percent | 2021 | 0.56374139 | 0.675361067 | 0.463900194 |
| Deaths | Middle SDI | Both | 65+ years | diarrhea diseases | Unsafe sanitation | Number | 2021 | 28738.01009 | 43974.04484 | 14908.85074 |
| DALYs (Disability-Adjusted Life Years) | Middle SDI | Both | 65+ years | diarrhea diseases | Unsafe sanitation | Rate | 2021 | 377.8591202 | 569.7017314 | 210.1236196 |
| DALYs (Disability-Adjusted Life Years) | Middle SDI | Both | 65+ years | diarrhea diseases | Unsafe sanitation | Percent | 2021 | 0.563107898 | 0.673870711 | 0.462998082 |
| DALYs (Disability-Adjusted Life Years) | Middle SDI | Both | 65+ years | diarrhea diseases | Unsafe sanitation | Number | 2021 | 473425.9883 | 711999.6155 | 263155.2086 |
| Deaths | Low-middle SDI | Both | 65+ years | diarrhea diseases | Unsafe sanitation | Rate | 2021 | 227.2197271 | 389.5497012 | 136.8732036 |
| Deaths | Low-middle SDI | Both | 65+ years | diarrhea diseases | Unsafe sanitation | Percent | 2021 | 1.016372124 | 1.14272189 | 0.891530109 |
| Deaths | Low-middle SDI | Both | 65+ years | diarrhea diseases | Unsafe sanitation | Number | 2021 | 148929.0553 | 254412.5035 | 89965.50113 |
| DALYs (Disability-Adjusted Life Years) | Low-middle SDI | Both | 65+ years | diarrhea diseases | Unsafe sanitation | Rate | 2021 | 3782.016396 | 6424.511116 | 2354.60194 |
| DALYs (Disability-Adjusted Life Years) | Low-middle SDI | Both | 65+ years | diarrhea diseases | Unsafe sanitation | Percent | 2021 | 1.015071044 | 1.139467406 | 0.891470395 |
| DALYs (Disability-Adjusted Life Years) | Low-middle SDI | Both | 65+ years | diarrhea diseases | Unsafe sanitation | Number | 2021 | 2378100.097 | 4025327.779 | 1482322.931 |
| Deaths | Low SDI | Both | 65+ years | diarrhea diseases | Unsafe sanitation | Rate | 2021 | 405.0240773 | 659.321039 | 232.8968578 |
| Deaths | Low SDI | Both | 65+ years | diarrhea diseases | Unsafe sanitation | Percent | 2021 | 1.210150497 | 1.327543139 | 1.082396333 |
| Deaths | Low SDI | Both | 65+ years | diarrhea diseases | Unsafe sanitation | Number | 2021 | 82697.0653 | 134065.7956 | 47584.86475 |
| DALYs (Disability-Adjusted Life Years) | Low SDI | Both | 65+ years | diarrhea diseases | Unsafe sanitation | Rate | 2021 | 7072.096645 | 11528.9566 | 4165.785224 |
| DALYs (Disability-Adjusted Life Years) | Low SDI | Both | 65+ years | diarrhea diseases | Unsafe sanitation | Percent | 2021 | 1.209163676 | 1.32682327 | 1.081509171 |
| DALYs (Disability-Adjusted Life Years) | Low SDI | Both | 65+ years | diarrhea diseases | Unsafe sanitation | Number | 2021 | 1393055.255 | 2262747.035 | 819429.9427 |
| Deaths | High-middle SDI | Both | 65+ years | diarrhea diseases | Unsafe sanitation | Rate | 2021 | 1.536889298 | 2.32459837 | 0.857181973 |
| Deaths | High-middle SDI | Both | 65+ years | diarrhea diseases | Unsafe sanitation | Percent | 2021 | 0.314828035 | 0.405183858 | 0.2408524 |
| Deaths | High-middle SDI | Both | 65+ years | diarrhea diseases | Unsafe sanitation | Number | 2021 | 1657.607109 | 2503.385985 | 928.3753848 |
| DALYs (Disability-Adjusted Life Years) | High-middle SDI | Both | 65+ years | diarrhea diseases | Unsafe sanitation | Rate | 2021 | 29.63973135 | 42.55175244 | 18.10462298 |
| DALYs (Disability-Adjusted Life Years) | High-middle SDI | Both | 65+ years | diarrhea diseases | Unsafe sanitation | Percent | 2021 | 0.326478897 | 0.41651708 | 0.254727672 |
| DALYs (Disability-Adjusted Life Years) | High-middle SDI | Both | 65+ years | diarrhea diseases | Unsafe sanitation | Number | 2021 | 29697.64013 | 42656.91061 | 18036.58679 |
| Deaths | High SDI | Both | 65+ years | diarrhea diseases | Unsafe sanitation | Rate | 2021 | 0.726112057 | 1.017419132 | 0.475525203 |
| Deaths | High SDI | Both | 65+ years | diarrhea diseases | Unsafe sanitation | Percent | 2021 | 0.088359691 | 0.12327875 | 0.060563359 |
| Deaths | High SDI | Both | 65+ years | diarrhea diseases | Unsafe sanitation | Number | 2021 | 956.1481913 | 1338.865027 | 624.6616289 |
| DALYs (Disability-Adjusted Life Years) | High SDI | Both | 65+ years | diarrhea diseases | Unsafe sanitation | Rate | 2021 | 12.3323729 | 17.16602942 | 8.332064846 |
| DALYs (Disability-Adjusted Life Years) | High SDI | Both | 65+ years | diarrhea diseases | Unsafe sanitation | Percent | 2021 | 0.085836058 | 0.117093616 | 0.060330087 |
| DALYs (Disability-Adjusted Life Years) | High SDI | Both | 65+ years | diarrhea diseases | Unsafe sanitation | Number | 2021 | 14838.53819 | 20662.21019 | 10002.66738 |
| Deaths | Global | Both | 65+ years | diarrhea diseases | Unsafe sanitation | Rate | 2021 | 58.84169625 | 96.95261071 | 34.78434557 |
| Deaths | Global | Both | 65+ years | diarrhea diseases | Unsafe sanitation | Percent | 2021 | 0.943132914 | 1.074190608 | 0.819310248 |
| Deaths | Global | Both | 65+ years | diarrhea diseases | Unsafe sanitation | Number | 2021 | 263060.2935 | 432392.4542 | 156016.7223 |
| DALYs (Disability-Adjusted Life Years) | Global | Both | 65+ years | diarrhea diseases | Unsafe sanitation | Rate | 2021 | 1016.76234 | 1647.305242 | 625.7305165 |
| DALYs (Disability-Adjusted Life Years) | Global | Both | 65+ years | diarrhea diseases | Unsafe sanitation | Percent | 2021 | 0.935308932 | 1.064591878 | 0.814145204 |
| DALYs (Disability-Adjusted Life Years) | Global | Both | 65+ years | diarrhea diseases | Unsafe sanitation | Number | 2021 | 4290478.428 | 6948335.722 | 2643374.863 |
| Deaths | Middle SDI | Both | 65+ years | diarrhea diseases | Unsafe sanitation | Rate | 2020 | 23.27703383 | 34.95197591 | 11.90085079 |
| Deaths | Middle SDI | Both | 65+ years | diarrhea diseases | Unsafe sanitation | Percent | 2020 | 0.581863892 | 0.696256841 | 0.478629043 |
| Deaths | Middle SDI | Both | 65+ years | diarrhea diseases | Unsafe sanitation | Number | 2020 | 29482.85137 | 44128.53855 | 15097.71103 |
| DALYs (Disability-Adjusted Life Years) | Middle SDI | Both | 65+ years | diarrhea diseases | Unsafe sanitation | Rate | 2020 | 397.3271324 | 585.8092801 | 220.299332 |
| DALYs (Disability-Adjusted Life Years) | Middle SDI | Both | 65+ years | diarrhea diseases | Unsafe sanitation | Percent | 2020 | 0.580956622 | 0.694362155 | 0.478593876 |
| DALYs (Disability-Adjusted Life Years) | Middle SDI | Both | 65+ years | diarrhea diseases | Unsafe sanitation | Number | 2020 | 479867.3026 | 705902.1216 | 265262.2415 |
| Deaths | Low-middle SDI | Both | 65+ years | diarrhea diseases | Unsafe sanitation | Rate | 2020 | 241.5638267 | 405.805306 | 145.8024888 |
| Deaths | Low-middle SDI | Both | 65+ years | diarrhea diseases | Unsafe sanitation | Percent | 2020 | 1.032138436 | 1.157778823 | 0.907195917 |
| Deaths | Low-middle SDI | Both | 65+ years | diarrhea diseases | Unsafe sanitation | Number | 2020 | 154450.8336 | 258593.9794 | 93375.98473 |
| DALYs (Disability-Adjusted Life Years) | Low-middle SDI | Both | 65+ years | diarrhea diseases | Unsafe sanitation | Rate | 2020 | 3994.146743 | 6650.987341 | 2481.94146 |
| DALYs (Disability-Adjusted Life Years) | Low-middle SDI | Both | 65+ years | diarrhea diseases | Unsafe sanitation | Percent | 2020 | 1.030980708 | 1.156555812 | 0.90694643 |
| DALYs (Disability-Adjusted Life Years) | Low-middle SDI | Both | 65+ years | diarrhea diseases | Unsafe sanitation | Number | 2020 | 2451638.109 | 4068924.055 | 1524953.441 |
| Deaths | Low SDI | Both | 65+ years | diarrhea diseases | Unsafe sanitation | Rate | 2020 | 430.3034574 | 701.6500871 | 253.6371043 |
| Deaths | Low SDI | Both | 65+ years | diarrhea diseases | Unsafe sanitation | Percent | 2020 | 1.217831588 | 1.331103548 | 1.090956062 |
| Deaths | Low SDI | Both | 65+ years | diarrhea diseases | Unsafe sanitation | Number | 2020 | 85978.34705 | 139478.0978 | 50865.00935 |
| DALYs (Disability-Adjusted Life Years) | Low SDI | Both | 65+ years | diarrhea diseases | Unsafe sanitation | Rate | 2020 | 7436.784714 | 12168.48542 | 4467.546091 |
| DALYs (Disability-Adjusted Life Years) | Low SDI | Both | 65+ years | diarrhea diseases | Unsafe sanitation | Percent | 2020 | 1.217087735 | 1.330917286 | 1.091209658 |
| DALYs (Disability-Adjusted Life Years) | Low SDI | Both | 65+ years | diarrhea diseases | Unsafe sanitation | Number | 2020 | 1435726.455 | 2338231.481 | 865713.7459 |
| Deaths | High-middle SDI | Both | 65+ years | diarrhea diseases | Unsafe sanitation | Rate | 2020 | 1.587371629 | 2.374299884 | 0.894450632 |
| Deaths | High-middle SDI | Both | 65+ years | diarrhea diseases | Unsafe sanitation | Percent | 2020 | 0.320702281 | 0.41561192 | 0.246816504 |
| Deaths | High-middle SDI | Both | 65+ years | diarrhea diseases | Unsafe sanitation | Number | 2020 | 1657.919689 | 2472.692943 | 938.0370346 |
| DALYs (Disability-Adjusted Life Years) | High-middle SDI | Both | 65+ years | diarrhea diseases | Unsafe sanitation | Rate | 2020 | 30.3237851 | 43.72806721 | 18.61844147 |
| DALYs (Disability-Adjusted Life Years) | High-middle SDI | Both | 65+ years | diarrhea diseases | Unsafe sanitation | Percent | 2020 | 0.332355047 | 0.424249393 | 0.259631239 |
| DALYs (Disability-Adjusted Life Years) | High-middle SDI | Both | 65+ years | diarrhea diseases | Unsafe sanitation | Number | 2020 | 29463.95985 | 42518.06382 | 17957.985 |
| Deaths | High SDI | Both | 65+ years | diarrhea diseases | Unsafe sanitation | Rate | 2020 | 0.73838718 | 1.032204856 | 0.483409973 |
| Deaths | High SDI | Both | 65+ years | diarrhea diseases | Unsafe sanitation | Percent | 2020 | 0.089046084 | 0.124850684 | 0.061206669 |
| Deaths | High SDI | Both | 65+ years | diarrhea diseases | Unsafe sanitation | Number | 2020 | 949.5361164 | 1326.095218 | 620.7812467 |
| DALYs (Disability-Adjusted Life Years) | High SDI | Both | 65+ years | diarrhea diseases | Unsafe sanitation | Rate | 2020 | 12.48699509 | 17.43243864 | 8.40878731 |
| DALYs (Disability-Adjusted Life Years) | High SDI | Both | 65+ years | diarrhea diseases | Unsafe sanitation | Percent | 2020 | 0.086183262 | 0.117692602 | 0.060777017 |
| DALYs (Disability-Adjusted Life Years) | High SDI | Both | 65+ years | diarrhea diseases | Unsafe sanitation | Number | 2020 | 14679.58354 | 20484.26948 | 9854.386075 |
| Deaths | Global | Both | 65+ years | diarrhea diseases | Unsafe sanitation | Rate | 2020 | 62.8785641 | 100.7764179 | 37.90606365 |
| Deaths | Global | Both | 65+ years | diarrhea diseases | Unsafe sanitation | Percent | 2020 | 0.961093244 | 1.090328505 | 0.840968009 |
| Deaths | Global | Both | 65+ years | diarrhea diseases | Unsafe sanitation | Number | 2020 | 272604.2912 | 434768.0189 | 165257.7106 |
| DALYs (Disability-Adjusted Life Years) | Global | Both | 65+ years | diarrhea diseases | Unsafe sanitation | Rate | 2020 | 1077.706168 | 1713.999956 | 662.5086456 |
| DALYs (Disability-Adjusted Life Years) | Global | Both | 65+ years | diarrhea diseases | Unsafe sanitation | Percent | 2020 | 0.953430173 | 1.081416818 | 0.835851254 |
| DALYs (Disability-Adjusted Life Years) | Global | Both | 65+ years | diarrhea diseases | Unsafe sanitation | Number | 2020 | 4412762.336 | 6997567.856 | 2718254.211 |
| Deaths | Middle SDI | Both | 65+ years | diarrhea diseases | Unsafe sanitation | Rate | 2019 | 24.64320006 | 36.90879123 | 12.39163349 |
| Deaths | Middle SDI | Both | 65+ years | diarrhea diseases | Unsafe sanitation | Percent | 2019 | 0.59803402 | 0.713461172 | 0.493186682 |
| Deaths | Middle SDI | Both | 65+ years | diarrhea diseases | Unsafe sanitation | Number | 2019 | 30043.92167 | 44843.88781 | 15110.0844 |
| DALYs (Disability-Adjusted Life Years) | Middle SDI | Both | 65+ years | diarrhea diseases | Unsafe sanitation | Rate | 2019 | 419.1959376 | 616.9302404 | 226.1706565 |
| DALYs (Disability-Adjusted Life Years) | Middle SDI | Both | 65+ years | diarrhea diseases | Unsafe sanitation | Percent | 2019 | 0.597190597 | 0.711298271 | 0.492962858 |
| DALYs (Disability-Adjusted Life Years) | Middle SDI | Both | 65+ years | diarrhea diseases | Unsafe sanitation | Number | 2019 | 486161.5647 | 713292.6884 | 261099.3661 |
| Deaths | Low-middle SDI | Both | 65+ years | diarrhea diseases | Unsafe sanitation | Rate | 2019 | 256.947299 | 436.5175353 | 158.054057 |
| Deaths | Low-middle SDI | Both | 65+ years | diarrhea diseases | Unsafe sanitation | Percent | 2019 | 1.048069387 | 1.176188414 | 0.923701859 |
| Deaths | Low-middle SDI | Both | 65+ years | diarrhea diseases | Unsafe sanitation | Number | 2019 | 159814.7518 | 270612.4201 | 98688.32421 |
| DALYs (Disability-Adjusted Life Years) | Low-middle SDI | Both | 65+ years | diarrhea diseases | Unsafe sanitation | Rate | 2019 | 4252.655042 | 7154.877071 | 2680.339459 |
| DALYs (Disability-Adjusted Life Years) | Low-middle SDI | Both | 65+ years | diarrhea diseases | Unsafe sanitation | Percent | 2019 | 1.047146522 | 1.175316673 | 0.923063317 |
| DALYs (Disability-Adjusted Life Years) | Low-middle SDI | Both | 65+ years | diarrhea diseases | Unsafe sanitation | Number | 2019 | 2535829.932 | 4254426.234 | 1605214.842 |
| Deaths | Low SDI | Both | 65+ years | diarrhea diseases | Unsafe sanitation | Rate | 2019 | 453.8250553 | 751.5099367 | 268.3426981 |
| Deaths | Low SDI | Both | 65+ years | diarrhea diseases | Unsafe sanitation | Percent | 2019 | 1.224632213 | 1.340168855 | 1.097877447 |
| Deaths | Low SDI | Both | 65+ years | diarrhea diseases | Unsafe sanitation | Number | 2019 | 88504.74722 | 145733.7254 | 52540.65196 |
| DALYs (Disability-Adjusted Life Years) | Low SDI | Both | 65+ years | diarrhea diseases | Unsafe sanitation | Rate | 2019 | 7839.048822 | 13041.88982 | 4703.055959 |
| DALYs (Disability-Adjusted Life Years) | Low SDI | Both | 65+ years | diarrhea diseases | Unsafe sanitation | Percent | 2019 | 1.223998661 | 1.339780228 | 1.097820844 |
| DALYs (Disability-Adjusted Life Years) | Low SDI | Both | 65+ years | diarrhea diseases | Unsafe sanitation | Number | 2019 | 1474720.647 | 2440311.65 | 887713.3885 |
| Deaths | High-middle SDI | Both | 65+ years | diarrhea diseases | Unsafe sanitation | Rate | 2019 | 1.661127289 | 2.512599517 | 0.942354599 |
| Deaths | High-middle SDI | Both | 65+ years | diarrhea diseases | Unsafe sanitation | Percent | 2019 | 0.328330044 | 0.421710148 | 0.252429825 |
| Deaths | High-middle SDI | Both | 65+ years | diarrhea diseases | Unsafe sanitation | Number | 2019 | 1677.491212 | 2534.818945 | 957.1502743 |
| DALYs (Disability-Adjusted Life Years) | High-middle SDI | Both | 65+ years | diarrhea diseases | Unsafe sanitation | Rate | 2019 | 31.56733795 | 45.83751953 | 19.17846026 |
| DALYs (Disability-Adjusted Life Years) | High-middle SDI | Both | 65+ years | diarrhea diseases | Unsafe sanitation | Percent | 2019 | 0.339307177 | 0.429137978 | 0.267252766 |
| DALYs (Disability-Adjusted Life Years) | High-middle SDI | Both | 65+ years | diarrhea diseases | Unsafe sanitation | Number | 2019 | 29630.70694 | 42982.31752 | 17892.14663 |
| Deaths | High SDI | Both | 65+ years | diarrhea diseases | Unsafe sanitation | Rate | 2019 | 0.763405032 | 1.066166917 | 0.501229595 |
| Deaths | High SDI | Both | 65+ years | diarrhea diseases | Unsafe sanitation | Percent | 2019 | 0.089962689 | 0.126290667 | 0.061956606 |
| Deaths | High SDI | Both | 65+ years | diarrhea diseases | Unsafe sanitation | Number | 2019 | 953.5291835 | 1330.430967 | 624.9812383 |
| DALYs (Disability-Adjusted Life Years) | High SDI | Both | 65+ years | diarrhea diseases | Unsafe sanitation | Rate | 2019 | 12.82290941 | 17.85401366 | 8.648101853 |
| DALYs (Disability-Adjusted Life Years) | High SDI | Both | 65+ years | diarrhea diseases | Unsafe sanitation | Percent | 2019 | 0.086630126 | 0.117931435 | 0.061226633 |
| DALYs (Disability-Adjusted Life Years) | High SDI | Both | 65+ years | diarrhea diseases | Unsafe sanitation | Number | 2019 | 14667.42591 | 20402.41999 | 9860.265769 |
| Deaths | Global | Both | 65+ years | diarrhea diseases | Unsafe sanitation | Rate | 2019 | 67.05050499 | 109.9055624 | 40.52508005 |
| Deaths | Global | Both | 65+ years | diarrhea diseases | Unsafe sanitation | Percent | 2019 | 0.977578752 | 1.104469318 | 0.857985507 |
| Deaths | Global | Both | 65+ years | diarrhea diseases | Unsafe sanitation | Number | 2019 | 281079.5824 | 458442.8258 | 170781.1649 |
| DALYs (Disability-Adjusted Life Years) | Global | Both | 65+ years | diarrhea diseases | Unsafe sanitation | Rate | 2019 | 1148.68967 | 1879.390807 | 707.1887042 |
| DALYs (Disability-Adjusted Life Years) | Global | Both | 65+ years | diarrhea diseases | Unsafe sanitation | Percent | 2019 | 0.970322811 | 1.097028395 | 0.852179966 |
| DALYs (Disability-Adjusted Life Years) | Global | Both | 65+ years | diarrhea diseases | Unsafe sanitation | Number | 2019 | 4542396.585 | 7405775.01 | 2807649.761 |
| Deaths | Middle SDI | Both | 65+ years | diarrhea diseases | Unsafe sanitation | Rate | 2018 | 26.40770627 | 39.68203321 | 13.73006619 |
| Deaths | Middle SDI | Both | 65+ years | diarrhea diseases | Unsafe sanitation | Percent | 2018 | 0.619984516 | 0.736531263 | 0.514394644 |
| Deaths | Middle SDI | Both | 65+ years | diarrhea diseases | Unsafe sanitation | Number | 2018 | 30695.32033 | 45977.43564 | 15996.28688 |
| DALYs (Disability-Adjusted Life Years) | Middle SDI | Both | 65+ years | diarrhea diseases | Unsafe sanitation | Rate | 2018 | 446.9298317 | 659.462799 | 246.6578785 |
| DALYs (Disability-Adjusted Life Years) | Middle SDI | Both | 65+ years | diarrhea diseases | Unsafe sanitation | Percent | 2018 | 0.618982504 | 0.734488152 | 0.513587671 |
| DALYs (Disability-Adjusted Life Years) | Middle SDI | Both | 65+ years | diarrhea diseases | Unsafe sanitation | Number | 2018 | 494857.3439 | 728204.1311 | 272241.5382 |
| Deaths | Low-middle SDI | Both | 65+ years | diarrhea diseases | Unsafe sanitation | Rate | 2018 | 276.4091083 | 469.7678586 | 169.6626168 |
| Deaths | Low-middle SDI | Both | 65+ years | diarrhea diseases | Unsafe sanitation | Percent | 2018 | 1.068382471 | 1.198162419 | 0.944128241 |
| Deaths | Low-middle SDI | Both | 65+ years | diarrhea diseases | Unsafe sanitation | Number | 2018 | 166082.2848 | 281331.3036 | 102273.1076 |
| DALYs (Disability-Adjusted Life Years) | Low-middle SDI | Both | 65+ years | diarrhea diseases | Unsafe sanitation | Rate | 2018 | 4584.111149 | 7738.991424 | 2892.636397 |
| DALYs (Disability-Adjusted Life Years) | Low-middle SDI | Both | 65+ years | diarrhea diseases | Unsafe sanitation | Percent | 2018 | 1.067602543 | 1.195246949 | 0.944374056 |
| DALYs (Disability-Adjusted Life Years) | Low-middle SDI | Both | 65+ years | diarrhea diseases | Unsafe sanitation | Number | 2018 | 2640797.556 | 4446678.012 | 1672387.654 |
| Deaths | Low SDI | Both | 65+ years | diarrhea diseases | Unsafe sanitation | Rate | 2018 | 480.9983552 | 808.009527 | 284.3243067 |
| Deaths | Low SDI | Both | 65+ years | diarrhea diseases | Unsafe sanitation | Percent | 2018 | 1.233726904 | 1.346470504 | 1.11004347 |
| Deaths | Low SDI | Both | 65+ years | diarrhea diseases | Unsafe sanitation | Number | 2018 | 90842.59397 | 151984.0132 | 53813.08289 |
| DALYs (Disability-Adjusted Life Years) | Low SDI | Both | 65+ years | diarrhea diseases | Unsafe sanitation | Rate | 2018 | 8304.890255 | 13923.83104 | 4998.918411 |
| DALYs (Disability-Adjusted Life Years) | Low SDI | Both | 65+ years | diarrhea diseases | Unsafe sanitation | Percent | 2018 | 1.233278976 | 1.345739519 | 1.109443109 |
| DALYs (Disability-Adjusted Life Years) | Low SDI | Both | 65+ years | diarrhea diseases | Unsafe sanitation | Number | 2018 | 1512670.081 | 2524749.721 | 912562.7521 |
| Deaths | High-middle SDI | Both | 65+ years | diarrhea diseases | Unsafe sanitation | Rate | 2018 | 1.726036289 | 2.580593617 | 0.995144197 |
| Deaths | High-middle SDI | Both | 65+ years | diarrhea diseases | Unsafe sanitation | Percent | 2018 | 0.338149724 | 0.431951933 | 0.260120519 |
| Deaths | High-middle SDI | Both | 65+ years | diarrhea diseases | Unsafe sanitation | Number | 2018 | 1677.43105 | 2506.574399 | 971.9940237 |
| DALYs (Disability-Adjusted Life Years) | High-middle SDI | Both | 65+ years | diarrhea diseases | Unsafe sanitation | Rate | 2018 | 32.72395012 | 47.03344978 | 20.08553323 |
| DALYs (Disability-Adjusted Life Years) | High-middle SDI | Both | 65+ years | diarrhea diseases | Unsafe sanitation | Percent | 2018 | 0.348185341 | 0.440167247 | 0.274100909 |
| DALYs (Disability-Adjusted Life Years) | High-middle SDI | Both | 65+ years | diarrhea diseases | Unsafe sanitation | Number | 2018 | 29598.63622 | 42462.23094 | 18062.74988 |
| Deaths | High SDI | Both | 65+ years | diarrhea diseases | Unsafe sanitation | Rate | 2018 | 0.828635967 | 1.16301472 | 0.543858613 |
| Deaths | High SDI | Both | 65+ years | diarrhea diseases | Unsafe sanitation | Percent | 2018 | 0.091680205 | 0.129525441 | 0.063210605 |
| Deaths | High SDI | Both | 65+ years | diarrhea diseases | Unsafe sanitation | Number | 2018 | 1004.930327 | 1409.320371 | 658.3694852 |
| DALYs (Disability-Adjusted Life Years) | High SDI | Both | 65+ years | diarrhea diseases | Unsafe sanitation | Rate | 2018 | 13.74219417 | 19.09987948 | 9.241880871 |
| DALYs (Disability-Adjusted Life Years) | High SDI | Both | 65+ years | diarrhea diseases | Unsafe sanitation | Percent | 2018 | 0.087454087 | 0.11966735 | 0.061382163 |
| DALYs (Disability-Adjusted Life Years) | High SDI | Both | 65+ years | diarrhea diseases | Unsafe sanitation | Number | 2018 | 15323.8734 | 21234.02819 | 10275.94713 |
| Deaths | Global | Both | 65+ years | diarrhea diseases | Unsafe sanitation | Rate | 2018 | 71.97197396 | 117.4798838 | 43.58194243 |
| Deaths | Global | Both | 65+ years | diarrhea diseases | Unsafe sanitation | Percent | 2018 | 0.996830309 | 1.123897229 | 0.873544092 |
| Deaths | Global | Both | 65+ years | diarrhea diseases | Unsafe sanitation | Number | 2018 | 290388.1178 | 472354.8945 | 176294.489 |
| DALYs (Disability-Adjusted Life Years) | Global | Both | 65+ years | diarrhea diseases | Unsafe sanitation | Rate | 2018 | 1231.938917 | 1998.857142 | 763.2475782 |
| DALYs (Disability-Adjusted Life Years) | Global | Both | 65+ years | diarrhea diseases | Unsafe sanitation | Percent | 2018 | 0.989866728 | 1.116974005 | 0.867478465 |
| DALYs (Disability-Adjusted Life Years) | Global | Both | 65+ years | diarrhea diseases | Unsafe sanitation | Number | 2018 | 4694636.903 | 7601162.906 | 2917382.59 |
| Deaths | Middle SDI | Both | 65+ years | diarrhea diseases | Unsafe sanitation | Rate | 2017 | 28.23843725 | 42.2092514 | 14.57295858 |
| Deaths | Middle SDI | Both | 65+ years | diarrhea diseases | Unsafe sanitation | Percent | 2017 | 0.642311817 | 0.759417574 | 0.532984818 |
| Deaths | Middle SDI | Both | 65+ years | diarrhea diseases | Unsafe sanitation | Number | 2017 | 31480.97892 | 46845.198 | 16236.57304 |
| DALYs (Disability-Adjusted Life Years) | Middle SDI | Both | 65+ years | diarrhea diseases | Unsafe sanitation | Rate | 2017 | 475.5359529 | 697.5951475 | 262.3599442 |
| DALYs (Disability-Adjusted Life Years) | Middle SDI | Both | 65+ years | diarrhea diseases | Unsafe sanitation | Percent | 2017 | 0.641207516 | 0.757466507 | 0.533001112 |
| DALYs (Disability-Adjusted Life Years) | Middle SDI | Both | 65+ years | diarrhea diseases | Unsafe sanitation | Number | 2017 | 504497.1919 | 736859.0524 | 276952.9099 |
| Deaths | Low-middle SDI | Both | 65+ years | diarrhea diseases | Unsafe sanitation | Rate | 2017 | 295.8422177 | 497.6731109 | 180.4312737 |
| Deaths | Low-middle SDI | Both | 65+ years | diarrhea diseases | Unsafe sanitation | Percent | 2017 | 1.088004763 | 1.217304903 | 0.964032394 |
| Deaths | Low-middle SDI | Both | 65+ years | diarrhea diseases | Unsafe sanitation | Number | 2017 | 171870.2009 | 288296.7674 | 105189.521 |
| DALYs (Disability-Adjusted Life Years) | Low-middle SDI | Both | 65+ years | diarrhea diseases | Unsafe sanitation | Rate | 2017 | 4889.524898 | 8130.066969 | 3051.394369 |
| DALYs (Disability-Adjusted Life Years) | Low-middle SDI | Both | 65+ years | diarrhea diseases | Unsafe sanitation | Percent | 2017 | 1.087173635 | 1.214549153 | 0.964368156 |
| DALYs (Disability-Adjusted Life Years) | Low-middle SDI | Both | 65+ years | diarrhea diseases | Unsafe sanitation | Number | 2017 | 2723270.984 | 4515548.567 | 1704133.617 |
| Deaths | Low SDI | Both | 65+ years | diarrhea diseases | Unsafe sanitation | Rate | 2017 | 515.1399858 | 862.1687692 | 309.588617 |
| Deaths | Low SDI | Both | 65+ years | diarrhea diseases | Unsafe sanitation | Percent | 2017 | 1.242942464 | 1.353238949 | 1.120452857 |
| Deaths | Low SDI | Both | 65+ years | diarrhea diseases | Unsafe sanitation | Number | 2017 | 94302.00976 | 157120.9521 | 56781.0358 |
| DALYs (Disability-Adjusted Life Years) | Low SDI | Both | 65+ years | diarrhea diseases | Unsafe sanitation | Rate | 2017 | 8831.415456 | 14762.09997 | 5382.54372 |
| DALYs (Disability-Adjusted Life Years) | Low SDI | Both | 65+ years | diarrhea diseases | Unsafe sanitation | Percent | 2017 | 1.242726316 | 1.352741678 | 1.119780631 |
| DALYs (Disability-Adjusted Life Years) | Low SDI | Both | 65+ years | diarrhea diseases | Unsafe sanitation | Number | 2017 | 1558822.069 | 2591988.283 | 951661.2502 |
| Deaths | High-middle SDI | Both | 65+ years | diarrhea diseases | Unsafe sanitation | Rate | 2017 | 1.799994941 | 2.647165362 | 1.036218587 |
| Deaths | High-middle SDI | Both | 65+ years | diarrhea diseases | Unsafe sanitation | Percent | 2017 | 0.34955056 | 0.447457836 | 0.270470123 |
| Deaths | High-middle SDI | Both | 65+ years | diarrhea diseases | Unsafe sanitation | Number | 2017 | 1696.253208 | 2487.800522 | 981.9738787 |
| DALYs (Disability-Adjusted Life Years) | High-middle SDI | Both | 65+ years | diarrhea diseases | Unsafe sanitation | Rate | 2017 | 34.07438269 | 48.81297492 | 20.99338294 |
| DALYs (Disability-Adjusted Life Years) | High-middle SDI | Both | 65+ years | diarrhea diseases | Unsafe sanitation | Percent | 2017 | 0.357733846 | 0.450613105 | 0.281786412 |
| DALYs (Disability-Adjusted Life Years) | High-middle SDI | Both | 65+ years | diarrhea diseases | Unsafe sanitation | Number | 2017 | 29817.59962 | 42653.28661 | 18264.00523 |
| Deaths | High SDI | Both | 65+ years | diarrhea diseases | Unsafe sanitation | Rate | 2017 | 0.900148408 | 1.269478197 | 0.58881032 |
| Deaths | High SDI | Both | 65+ years | diarrhea diseases | Unsafe sanitation | Percent | 2017 | 0.094239423 | 0.134341122 | 0.064511009 |
| Deaths | High SDI | Both | 65+ years | diarrhea diseases | Unsafe sanitation | Number | 2017 | 1058.104488 | 1491.229637 | 690.7067334 |
| DALYs (Disability-Adjusted Life Years) | High SDI | Both | 65+ years | diarrhea diseases | Unsafe sanitation | Rate | 2017 | 14.78900909 | 20.44691428 | 9.87969016 |
| DALYs (Disability-Adjusted Life Years) | High SDI | Both | 65+ years | diarrhea diseases | Unsafe sanitation | Percent | 2017 | 0.088386174 | 0.12139727 | 0.06196272 |
| DALYs (Disability-Adjusted Life Years) | High SDI | Both | 65+ years | diarrhea diseases | Unsafe sanitation | Number | 2017 | 16053.09876 | 22133.68325 | 10689.88084 |
| Deaths | Global | Both | 65+ years | diarrhea diseases | Unsafe sanitation | Rate | 2017 | 77.05882841 | 125.4851236 | 47.04711328 |
| Deaths | Global | Both | 65+ years | diarrhea diseases | Unsafe sanitation | Percent | 2017 | 1.015692237 | 1.146149506 | 0.892849417 |
| Deaths | Global | Both | 65+ years | diarrhea diseases | Unsafe sanitation | Number | 2017 | 300493.5733 | 486797.7366 | 183948.2297 |
| DALYs (Disability-Adjusted Life Years) | Global | Both | 65+ years | diarrhea diseases | Unsafe sanitation | Rate | 2017 | 1312.521781 | 2132.543482 | 810.5875187 |
| DALYs (Disability-Adjusted Life Years) | Global | Both | 65+ years | diarrhea diseases | Unsafe sanitation | Percent | 2017 | 1.008252413 | 1.135910006 | 0.887956551 |
| DALYs (Disability-Adjusted Life Years) | Global | Both | 65+ years | diarrhea diseases | Unsafe sanitation | Number | 2017 | 4833854.941 | 7813103.152 | 2987246.193 |
| Deaths | Middle SDI | Both | 65+ years | diarrhea diseases | Unsafe sanitation | Rate | 2016 | 29.97589605 | 44.45983636 | 15.58943315 |
| Deaths | Middle SDI | Both | 65+ years | diarrhea diseases | Unsafe sanitation | Percent | 2016 | 0.662385569 | 0.779282259 | 0.553505311 |
| Deaths | Middle SDI | Both | 65+ years | diarrhea diseases | Unsafe sanitation | Number | 2016 | 32085.64282 | 47345.81504 | 16723.96889 |
| DALYs (Disability-Adjusted Life Years) | Middle SDI | Both | 65+ years | diarrhea diseases | Unsafe sanitation | Rate | 2016 | 502.2076239 | 734.8034182 | 279.3757784 |
| DALYs (Disability-Adjusted Life Years) | Middle SDI | Both | 65+ years | diarrhea diseases | Unsafe sanitation | Percent | 2016 | 0.661174108 | 0.777698357 | 0.552779544 |
| DALYs (Disability-Adjusted Life Years) | Middle SDI | Both | 65+ years | diarrhea diseases | Unsafe sanitation | Number | 2016 | 511289.8161 | 744254.0247 | 283316.3379 |
| Deaths | Low-middle SDI | Both | 65+ years | diarrhea diseases | Unsafe sanitation | Rate | 2016 | 314.287185 | 525.5156347 | 190.3744756 |
| Deaths | Low-middle SDI | Both | 65+ years | diarrhea diseases | Unsafe sanitation | Percent | 2016 | 1.106337422 | 1.225262554 | 0.98214036 |
| Deaths | Low-middle SDI | Both | 65+ years | diarrhea diseases | Unsafe sanitation | Number | 2016 | 176348.9591 | 294020.8958 | 107052.4368 |
| DALYs (Disability-Adjusted Life Years) | Low-middle SDI | Both | 65+ years | diarrhea diseases | Unsafe sanitation | Rate | 2016 | 5177.866893 | 8564.129253 | 3201.231761 |
| DALYs (Disability-Adjusted Life Years) | Low-middle SDI | Both | 65+ years | diarrhea diseases | Unsafe sanitation | Percent | 2016 | 1.105541184 | 1.224174845 | 0.981959412 |
| DALYs (Disability-Adjusted Life Years) | Low-middle SDI | Both | 65+ years | diarrhea diseases | Unsafe sanitation | Number | 2016 | 2783746.359 | 4586160.95 | 1724314.238 |
| Deaths | Low SDI | Both | 65+ years | diarrhea diseases | Unsafe sanitation | Rate | 2016 | 544.656177 | 922.5931596 | 329.6423801 |
| Deaths | Low SDI | Both | 65+ years | diarrhea diseases | Unsafe sanitation | Percent | 2016 | 1.252552373 | 1.363812327 | 1.131359773 |
| Deaths | Low SDI | Both | 65+ years | diarrhea diseases | Unsafe sanitation | Number | 2016 | 96534.92986 | 162852.1601 | 58627.13792 |
| DALYs (Disability-Adjusted Life Years) | Low SDI | Both | 65+ years | diarrhea diseases | Unsafe sanitation | Rate | 2016 | 9291.121902 | 15729.40411 | 5696.197468 |
| DALYs (Disability-Adjusted Life Years) | Low SDI | Both | 65+ years | diarrhea diseases | Unsafe sanitation | Percent | 2016 | 1.25250844 | 1.363609554 | 1.131777429 |
| DALYs (Disability-Adjusted Life Years) | Low SDI | Both | 65+ years | diarrhea diseases | Unsafe sanitation | Number | 2016 | 1586272.322 | 2672890.64 | 975811.2143 |
| Deaths | High-middle SDI | Both | 65+ years | diarrhea diseases | Unsafe sanitation | Rate | 2016 | 1.846814334 | 2.708930934 | 1.069694693 |
| Deaths | High-middle SDI | Both | 65+ years | diarrhea diseases | Unsafe sanitation | Percent | 2016 | 0.365166386 | 0.467311204 | 0.282256388 |
| Deaths | High-middle SDI | Both | 65+ years | diarrhea diseases | Unsafe sanitation | Number | 2016 | 1685.019274 | 2462.615214 | 982.7792251 |
| DALYs (Disability-Adjusted Life Years) | High-middle SDI | Both | 65+ years | diarrhea diseases | Unsafe sanitation | Rate | 2016 | 35.03273629 | 49.74495208 | 21.78219582 |
| DALYs (Disability-Adjusted Life Years) | High-middle SDI | Both | 65+ years | diarrhea diseases | Unsafe sanitation | Percent | 2016 | 0.369902472 | 0.465275651 | 0.293106076 |
| DALYs (Disability-Adjusted Life Years) | High-middle SDI | Both | 65+ years | diarrhea diseases | Unsafe sanitation | Number | 2016 | 29648.54779 | 41953.79106 | 18315.67695 |
| Deaths | High SDI | Both | 65+ years | diarrhea diseases | Unsafe sanitation | Rate | 2016 | 0.962704045 | 1.361633263 | 0.627598684 |
| Deaths | High SDI | Both | 65+ years | diarrhea diseases | Unsafe sanitation | Percent | 2016 | 0.096244357 | 0.13819603 | 0.065756659 |
| Deaths | High SDI | Both | 65+ years | diarrhea diseases | Unsafe sanitation | Number | 2016 | 1095.201587 | 1547.35027 | 712.6087388 |
| DALYs (Disability-Adjusted Life Years) | High SDI | Both | 65+ years | diarrhea diseases | Unsafe sanitation | Rate | 2016 | 15.77329772 | 21.93085182 | 10.54060467 |
| DALYs (Disability-Adjusted Life Years) | High SDI | Both | 65+ years | diarrhea diseases | Unsafe sanitation | Percent | 2016 | 0.088915551 | 0.12245779 | 0.062495408 |
| DALYs (Disability-Adjusted Life Years) | High SDI | Both | 65+ years | diarrhea diseases | Unsafe sanitation | Number | 2016 | 16623.25299 | 23050.42767 | 11080.6394 |
| Deaths | Global | Both | 65+ years | diarrhea diseases | Unsafe sanitation | Rate | 2016 | 81.70418112 | 133.3481424 | 49.89241166 |
| Deaths | Global | Both | 65+ years | diarrhea diseases | Unsafe sanitation | Percent | 2016 | 1.033221588 | 1.163332985 | 0.911957662 |
| Deaths | Global | Both | 65+ years | diarrhea diseases | Unsafe sanitation | Number | 2016 | 307836.6311 | 499553.9567 | 188698.4965 |
| DALYs (Disability-Adjusted Life Years) | Global | Both | 65+ years | diarrhea diseases | Unsafe sanitation | Rate | 2016 | 1385.010157 | 2258.575694 | 852.6305885 |
| DALYs (Disability-Adjusted Life Years) | Global | Both | 65+ years | diarrhea diseases | Unsafe sanitation | Percent | 2016 | 1.025070514 | 1.152961058 | 0.904064273 |
| DALYs (Disability-Adjusted Life Years) | Global | Both | 65+ years | diarrhea diseases | Unsafe sanitation | Number | 2016 | 4928985.043 | 7988862.846 | 3042807.789 |
| Deaths | Middle SDI | Both | 65+ years | diarrhea diseases | Unsafe sanitation | Rate | 2015 | 32.18689559 | 47.50529904 | 16.55914529 |
| Deaths | Middle SDI | Both | 65+ years | diarrhea diseases | Unsafe sanitation | Percent | 2015 | 0.68507791 | 0.802650628 | 0.573397727 |
| Deaths | Middle SDI | Both | 65+ years | diarrhea diseases | Unsafe sanitation | Number | 2015 | 33140.14002 | 48655.98203 | 17058.3083 |
| DALYs (Disability-Adjusted Life Years) | Middle SDI | Both | 65+ years | diarrhea diseases | Unsafe sanitation | Rate | 2015 | 536.7745879 | 785.5672833 | 297.3988992 |
| DALYs (Disability-Adjusted Life Years) | Middle SDI | Both | 65+ years | diarrhea diseases | Unsafe sanitation | Percent | 2015 | 0.683603657 | 0.798912334 | 0.572310971 |
| DALYs (Disability-Adjusted Life Years) | Middle SDI | Both | 65+ years | diarrhea diseases | Unsafe sanitation | Number | 2015 | 525179.3502 | 764632.3275 | 290044.5461 |
| Deaths | Low-middle SDI | Both | 65+ years | diarrhea diseases | Unsafe sanitation | Rate | 2015 | 342.92531 | 575.9487663 | 209.5899067 |
| Deaths | Low-middle SDI | Both | 65+ years | diarrhea diseases | Unsafe sanitation | Percent | 2015 | 1.125855302 | 1.247348852 | 1.001724682 |
| Deaths | Low-middle SDI | Both | 65+ years | diarrhea diseases | Unsafe sanitation | Number | 2015 | 186182.0227 | 311184.0732 | 114171.7628 |
| DALYs (Disability-Adjusted Life Years) | Low-middle SDI | Both | 65+ years | diarrhea diseases | Unsafe sanitation | Rate | 2015 | 5607.618668 | 9379.5186 | 3485.997264 |
| DALYs (Disability-Adjusted Life Years) | Low-middle SDI | Both | 65+ years | diarrhea diseases | Unsafe sanitation | Percent | 2015 | 1.125101006 | 1.245771178 | 1.002043763 |
| DALYs (Disability-Adjusted Life Years) | Low-middle SDI | Both | 65+ years | diarrhea diseases | Unsafe sanitation | Number | 2015 | 2913874.217 | 4845715.829 | 1815998.817 |
| Deaths | Low SDI | Both | 65+ years | diarrhea diseases | Unsafe sanitation | Rate | 2015 | 585.2013695 | 981.0250203 | 360.5609017 |
| Deaths | Low SDI | Both | 65+ years | diarrhea diseases | Unsafe sanitation | Percent | 2015 | 1.261670817 | 1.372652678 | 1.139412238 |
| Deaths | Low SDI | Both | 65+ years | diarrhea diseases | Unsafe sanitation | Number | 2015 | 100429.2025 | 167745.383 | 62031.60597 |
| DALYs (Disability-Adjusted Life Years) | Low SDI | Both | 65+ years | diarrhea diseases | Unsafe sanitation | Rate | 2015 | 9929.747642 | 16660.06038 | 6204.317 |
| DALYs (Disability-Adjusted Life Years) | Low SDI | Both | 65+ years | diarrhea diseases | Unsafe sanitation | Percent | 2015 | 1.261799406 | 1.371618401 | 1.139729656 |
| DALYs (Disability-Adjusted Life Years) | Low SDI | Both | 65+ years | diarrhea diseases | Unsafe sanitation | Number | 2015 | 1638274.835 | 2738809.212 | 1026055.235 |
| Deaths | High-middle SDI | Both | 65+ years | diarrhea diseases | Unsafe sanitation | Rate | 2015 | 1.885779391 | 2.784421201 | 1.09598635 |
| Deaths | High-middle SDI | Both | 65+ years | diarrhea diseases | Unsafe sanitation | Percent | 2015 | 0.379916382 | 0.479164479 | 0.296761027 |
| Deaths | High-middle SDI | Both | 65+ years | diarrhea diseases | Unsafe sanitation | Number | 2015 | 1672.165797 | 2463.107609 | 979.6138921 |
| DALYs (Disability-Adjusted Life Years) | High-middle SDI | Both | 65+ years | diarrhea diseases | Unsafe sanitation | Rate | 2015 | 36.01945747 | 50.94526771 | 22.29078603 |
| DALYs (Disability-Adjusted Life Years) | High-middle SDI | Both | 65+ years | diarrhea diseases | Unsafe sanitation | Percent | 2015 | 0.380974153 | 0.47349652 | 0.304260291 |
| DALYs (Disability-Adjusted Life Years) | High-middle SDI | Both | 65+ years | diarrhea diseases | Unsafe sanitation | Number | 2015 | 29503.2059 | 41557.68295 | 18161.1717 |
| Deaths | High SDI | Both | 65+ years | diarrhea diseases | Unsafe sanitation | Rate | 2015 | 1.013826011 | 1.431666795 | 0.659033609 |
| Deaths | High SDI | Both | 65+ years | diarrhea diseases | Unsafe sanitation | Percent | 2015 | 0.097982693 | 0.141105896 | 0.066732521 |
| Deaths | High SDI | Both | 65+ years | diarrhea diseases | Unsafe sanitation | Number | 2015 | 1132.661291 | 1597.465314 | 734.7985244 |
| DALYs (Disability-Adjusted Life Years) | High SDI | Both | 65+ years | diarrhea diseases | Unsafe sanitation | Rate | 2015 | 16.55094923 | 23.14927154 | 11.04370065 |
| DALYs (Disability-Adjusted Life Years) | High SDI | Both | 65+ years | diarrhea diseases | Unsafe sanitation | Percent | 2015 | 0.089499671 | 0.123224619 | 0.063015797 |
| DALYs (Disability-Adjusted Life Years) | High SDI | Both | 65+ years | diarrhea diseases | Unsafe sanitation | Number | 2015 | 17118.71842 | 23911.38141 | 11407.6666 |
| Deaths | Global | Both | 65+ years | diarrhea diseases | Unsafe sanitation | Rate | 2015 | 88.23980416 | 144.8120137 | 53.64226196 |
| Deaths | Global | Both | 65+ years | diarrhea diseases | Unsafe sanitation | Percent | 2015 | 1.053239955 | 1.182996978 | 0.929965027 |
| Deaths | Global | Both | 65+ years | diarrhea diseases | Unsafe sanitation | Number | 2015 | 322643.565 | 525827.8241 | 196945.1813 |
| DALYs (Disability-Adjusted Life Years) | Global | Both | 65+ years | diarrhea diseases | Unsafe sanitation | Rate | 2015 | 1487.300409 | 2427.632748 | 915.9460264 |
| DALYs (Disability-Adjusted Life Years) | Global | Both | 65+ years | diarrhea diseases | Unsafe sanitation | Percent | 2015 | 1.044426288 | 1.173694645 | 0.922793687 |
| DALYs (Disability-Adjusted Life Years) | Global | Both | 65+ years | diarrhea diseases | Unsafe sanitation | Number | 2015 | 5125359.32 | 8295393.901 | 3165310.358 |
| Deaths | Middle SDI | Both | 65+ years | diarrhea diseases | Unsafe sanitation | Rate | 2014 | 34.71263667 | 51.13819391 | 18.09632597 |
| Deaths | Middle SDI | Both | 65+ years | diarrhea diseases | Unsafe sanitation | Percent | 2014 | 0.709044217 | 0.824292419 | 0.600272428 |
| Deaths | Middle SDI | Both | 65+ years | diarrhea diseases | Unsafe sanitation | Number | 2014 | 34570.01846 | 50604.68778 | 18031.0405 |
| DALYs (Disability-Adjusted Life Years) | Middle SDI | Both | 65+ years | diarrhea diseases | Unsafe sanitation | Rate | 2014 | 577.1605019 | 845.030123 | 323.1011997 |
| DALYs (Disability-Adjusted Life Years) | Middle SDI | Both | 65+ years | diarrhea diseases | Unsafe sanitation | Percent | 2014 | 0.707468161 | 0.822107563 | 0.597758868 |
| DALYs (Disability-Adjusted Life Years) | Middle SDI | Both | 65+ years | diarrhea diseases | Unsafe sanitation | Number | 2014 | 546133.0056 | 794380.6086 | 304487.0738 |
| Deaths | Low-middle SDI | Both | 65+ years | diarrhea diseases | Unsafe sanitation | Rate | 2014 | 378.2915705 | 637.0190669 | 236.2449093 |
| Deaths | Low-middle SDI | Both | 65+ years | diarrhea diseases | Unsafe sanitation | Percent | 2014 | 1.143167167 | 1.268067811 | 1.01870528 |
| Deaths | Low-middle SDI | Both | 65+ years | diarrhea diseases | Unsafe sanitation | Number | 2014 | 199868.8748 | 334753.0961 | 125456.6942 |
| DALYs (Disability-Adjusted Life Years) | Low-middle SDI | Both | 65+ years | diarrhea diseases | Unsafe sanitation | Rate | 2014 | 6133.154868 | 10349.59991 | 3862.819758 |
| DALYs (Disability-Adjusted Life Years) | Low-middle SDI | Both | 65+ years | diarrhea diseases | Unsafe sanitation | Percent | 2014 | 1.142529754 | 1.266624475 | 1.017789941 |
| DALYs (Disability-Adjusted Life Years) | Low-middle SDI | Both | 65+ years | diarrhea diseases | Unsafe sanitation | Number | 2014 | 3102235.702 | 5202342.061 | 1961559.133 |
| Deaths | Low SDI | Both | 65+ years | diarrhea diseases | Unsafe sanitation | Rate | 2014 | 631.9289467 | 1065.239004 | 392.0318838 |
| Deaths | Low SDI | Both | 65+ years | diarrhea diseases | Unsafe sanitation | Percent | 2014 | 1.269488096 | 1.38081362 | 1.150556825 |
| Deaths | Low SDI | Both | 65+ years | diarrhea diseases | Unsafe sanitation | Number | 2014 | 105847.858 | 177762.4708 | 65866.47544 |
| DALYs (Disability-Adjusted Life Years) | Low SDI | Both | 65+ years | diarrhea diseases | Unsafe sanitation | Rate | 2014 | 10648.68703 | 17969.91758 | 6656.790851 |
| DALYs (Disability-Adjusted Life Years) | Low SDI | Both | 65+ years | diarrhea diseases | Unsafe sanitation | Percent | 2014 | 1.269727947 | 1.381633338 | 1.149884507 |
| DALYs (Disability-Adjusted Life Years) | Low SDI | Both | 65+ years | diarrhea diseases | Unsafe sanitation | Number | 2014 | 1716142.114 | 2883963.842 | 1076050.674 |
| Deaths | High-middle SDI | Both | 65+ years | diarrhea diseases | Unsafe sanitation | Rate | 2014 | 1.909631515 | 2.783226701 | 1.099131167 |
| Deaths | High-middle SDI | Both | 65+ years | diarrhea diseases | Unsafe sanitation | Percent | 2014 | 0.396987914 | 0.500219308 | 0.310581127 |
| Deaths | High-middle SDI | Both | 65+ years | diarrhea diseases | Unsafe sanitation | Number | 2014 | 1656.14136 | 2403.100265 | 960.8381125 |
| DALYs (Disability-Adjusted Life Years) | High-middle SDI | Both | 65+ years | diarrhea diseases | Unsafe sanitation | Rate | 2014 | 36.88804958 | 51.96034791 | 22.77489518 |
| DALYs (Disability-Adjusted Life Years) | High-middle SDI | Both | 65+ years | diarrhea diseases | Unsafe sanitation | Percent | 2014 | 0.395304178 | 0.492905498 | 0.315831766 |
| DALYs (Disability-Adjusted Life Years) | High-middle SDI | Both | 65+ years | diarrhea diseases | Unsafe sanitation | Number | 2014 | 29386.57587 | 41289.7664 | 18060.79432 |
| Deaths | High SDI | Both | 65+ years | diarrhea diseases | Unsafe sanitation | Rate | 2014 | 1.049881475 | 1.484943248 | 0.682827955 |
| Deaths | High SDI | Both | 65+ years | diarrhea diseases | Unsafe sanitation | Percent | 2014 | 0.100590343 | 0.145643597 | 0.068563592 |
| Deaths | High SDI | Both | 65+ years | diarrhea diseases | Unsafe sanitation | Number | 2014 | 1150.26725 | 1624.521271 | 746.7454769 |
| DALYs (Disability-Adjusted Life Years) | High SDI | Both | 65+ years | diarrhea diseases | Unsafe sanitation | Rate | 2014 | 17.13087267 | 24.0491448 | 11.43385403 |
| DALYs (Disability-Adjusted Life Years) | High SDI | Both | 65+ years | diarrhea diseases | Unsafe sanitation | Percent | 2014 | 0.090918617 | 0.125691102 | 0.063826179 |
| DALYs (Disability-Adjusted Life Years) | High SDI | Both | 65+ years | diarrhea diseases | Unsafe sanitation | Number | 2014 | 17349.43917 | 24342.75411 | 11567.731 |
| Deaths | Global | Both | 65+ years | diarrhea diseases | Unsafe sanitation | Rate | 2014 | 96.34816744 | 158.1967895 | 60.17946629 |
| Deaths | Global | Both | 65+ years | diarrhea diseases | Unsafe sanitation | Percent | 2014 | 1.073670915 | 1.20149069 | 0.953053444 |
| Deaths | Global | Both | 65+ years | diarrhea diseases | Unsafe sanitation | Number | 2014 | 343181.6907 | 558801.8868 | 215755.1173 |
| DALYs (Disability-Adjusted Life Years) | Global | Both | 65+ years | diarrhea diseases | Unsafe sanitation | Rate | 2014 | 1616.129771 | 2657.283083 | 1008.243153 |
| DALYs (Disability-Adjusted Life Years) | Global | Both | 65+ years | diarrhea diseases | Unsafe sanitation | Percent | 2014 | 1.064494765 | 1.191397763 | 0.94526044 |
| DALYs (Disability-Adjusted Life Years) | Global | Both | 65+ years | diarrhea diseases | Unsafe sanitation | Number | 2014 | 5412676.01 | 8813454.719 | 3389680.856 |
| Deaths | Middle SDI | Both | 65+ years | diarrhea diseases | Unsafe sanitation | Rate | 2013 | 37.55285677 | 54.96176353 | 19.79728919 |
| Deaths | Middle SDI | Both | 65+ years | diarrhea diseases | Unsafe sanitation | Percent | 2013 | 0.732741714 | 0.853042811 | 0.620563534 |
| Deaths | Middle SDI | Both | 65+ years | diarrhea diseases | Unsafe sanitation | Number | 2013 | 35953.94416 | 52421.8032 | 18967.1049 |
| DALYs (Disability-Adjusted Life Years) | Middle SDI | Both | 65+ years | diarrhea diseases | Unsafe sanitation | Rate | 2013 | 628.2932935 | 914.1755796 | 350.3015393 |
| DALYs (Disability-Adjusted Life Years) | Middle SDI | Both | 65+ years | diarrhea diseases | Unsafe sanitation | Percent | 2013 | 0.731275568 | 0.849678454 | 0.619924116 |
| DALYs (Disability-Adjusted Life Years) | Middle SDI | Both | 65+ years | diarrhea diseases | Unsafe sanitation | Number | 2013 | 569679.6503 | 826470.2706 | 316400.8981 |
| Deaths | Low-middle SDI | Both | 65+ years | diarrhea diseases | Unsafe sanitation | Rate | 2013 | 405.2435124 | 692.4250631 | 252.4522537 |
| Deaths | Low-middle SDI | Both | 65+ years | diarrhea diseases | Unsafe sanitation | Percent | 2013 | 1.157902326 | 1.278607331 | 1.034178708 |
| Deaths | Low-middle SDI | Both | 65+ years | diarrhea diseases | Unsafe sanitation | Number | 2013 | 207383.8298 | 352707.6314 | 129962.3744 |
| DALYs (Disability-Adjusted Life Years) | Low-middle SDI | Both | 65+ years | diarrhea diseases | Unsafe sanitation | Rate | 2013 | 6625.365919 | 11296.07873 | 4165.327163 |
| DALYs (Disability-Adjusted Life Years) | Low-middle SDI | Both | 65+ years | diarrhea diseases | Unsafe sanitation | Percent | 2013 | 1.157467724 | 1.277617628 | 1.034548197 |
| DALYs (Disability-Adjusted Life Years) | Low-middle SDI | Both | 65+ years | diarrhea diseases | Unsafe sanitation | Number | 2013 | 3244416.218 | 5500383.734 | 2050604.191 |
| Deaths | Low SDI | Both | 65+ years | diarrhea diseases | Unsafe sanitation | Rate | 2013 | 671.7283215 | 1128.265641 | 423.4489153 |
| Deaths | Low SDI | Both | 65+ years | diarrhea diseases | Unsafe sanitation | Percent | 2013 | 1.276850722 | 1.386754644 | 1.158932358 |
| Deaths | Low SDI | Both | 65+ years | diarrhea diseases | Unsafe sanitation | Number | 2013 | 109485.7657 | 183396.3041 | 69343.76041 |
| DALYs (Disability-Adjusted Life Years) | Low SDI | Both | 65+ years | diarrhea diseases | Unsafe sanitation | Rate | 2013 | 11352.07545 | 19097.34978 | 7168.875396 |
| DALYs (Disability-Adjusted Life Years) | Low SDI | Both | 65+ years | diarrhea diseases | Unsafe sanitation | Percent | 2013 | 1.277077682 | 1.386426504 | 1.158714812 |
| DALYs (Disability-Adjusted Life Years) | Low SDI | Both | 65+ years | diarrhea diseases | Unsafe sanitation | Number | 2013 | 1780495.591 | 2987201.215 | 1130132.382 |
| Deaths | High-middle SDI | Both | 65+ years | diarrhea diseases | Unsafe sanitation | Rate | 2013 | 1.961228659 | 2.854209689 | 1.114379968 |
| Deaths | High-middle SDI | Both | 65+ years | diarrhea diseases | Unsafe sanitation | Percent | 2013 | 0.414681922 | 0.518520822 | 0.32778481 |
| Deaths | High-middle SDI | Both | 65+ years | diarrhea diseases | Unsafe sanitation | Number | 2013 | 1657.814971 | 2398.858393 | 952.6056069 |
| DALYs (Disability-Adjusted Life Years) | High-middle SDI | Both | 65+ years | diarrhea diseases | Unsafe sanitation | Rate | 2013 | 38.42617383 | 54.24617322 | 23.80465686 |
| DALYs (Disability-Adjusted Life Years) | High-middle SDI | Both | 65+ years | diarrhea diseases | Unsafe sanitation | Percent | 2013 | 0.410708713 | 0.509895765 | 0.32891501 |
| DALYs (Disability-Adjusted Life Years) | High-middle SDI | Both | 65+ years | diarrhea diseases | Unsafe sanitation | Number | 2013 | 29614.05869 | 41628.39637 | 18332.49451 |
| Deaths | High SDI | Both | 65+ years | diarrhea diseases | Unsafe sanitation | Rate | 2013 | 1.108283523 | 1.563818888 | 0.719753846 |
| Deaths | High SDI | Both | 65+ years | diarrhea diseases | Unsafe sanitation | Percent | 2013 | 0.101556459 | 0.146488666 | 0.068990939 |
| Deaths | High SDI | Both | 65+ years | diarrhea diseases | Unsafe sanitation | Number | 2013 | 1187.862832 | 1673.772372 | 770.28502 |
| DALYs (Disability-Adjusted Life Years) | High SDI | Both | 65+ years | diarrhea diseases | Unsafe sanitation | Rate | 2013 | 17.92309696 | 25.198949 | 11.92952243 |
| DALYs (Disability-Adjusted Life Years) | High SDI | Both | 65+ years | diarrhea diseases | Unsafe sanitation | Percent | 2013 | 0.091257582 | 0.126179403 | 0.063875997 |
| DALYs (Disability-Adjusted Life Years) | High SDI | Both | 65+ years | diarrhea diseases | Unsafe sanitation | Number | 2013 | 17750.66204 | 24937.83972 | 11803.396 |
| Deaths | Global | Both | 65+ years | diarrhea diseases | Unsafe sanitation | Rate | 2013 | 103.3265383 | 171.6457103 | 63.1352367 |
| Deaths | Global | Both | 65+ years | diarrhea diseases | Unsafe sanitation | Percent | 2013 | 1.089828956 | 1.219644488 | 0.969071188 |
| Deaths | Global | Both | 65+ years | diarrhea diseases | Unsafe sanitation | Number | 2013 | 355758.4682 | 586804.2755 | 218277.2139 |
| DALYs (Disability-Adjusted Life Years) | Global | Both | 65+ years | diarrhea diseases | Unsafe sanitation | Rate | 2013 | 1749.765074 | 2917.366343 | 1088.561852 |
| DALYs (Disability-Adjusted Life Years) | Global | Both | 65+ years | diarrhea diseases | Unsafe sanitation | Percent | 2013 | 1.081256182 | 1.208858512 | 0.961504851 |
| DALYs (Disability-Adjusted Life Years) | Global | Both | 65+ years | diarrhea diseases | Unsafe sanitation | Number | 2013 | 5643398.244 | 9341144.627 | 3524568.084 |
| Deaths | Middle SDI | Both | 65+ years | diarrhea diseases | Unsafe sanitation | Rate | 2012 | 40.21696168 | 58.58027173 | 21.21112572 |
| Deaths | Middle SDI | Both | 65+ years | diarrhea diseases | Unsafe sanitation | Percent | 2012 | 0.7556258 | 0.876776626 | 0.642450834 |
| Deaths | Middle SDI | Both | 65+ years | diarrhea diseases | Unsafe sanitation | Number | 2012 | 37116.40158 | 53853.15368 | 19607.92379 |
| DALYs (Disability-Adjusted Life Years) | Middle SDI | Both | 65+ years | diarrhea diseases | Unsafe sanitation | Rate | 2012 | 677.3495414 | 978.0386386 | 376.2235802 |
| DALYs (Disability-Adjusted Life Years) | Middle SDI | Both | 65+ years | diarrhea diseases | Unsafe sanitation | Percent | 2012 | 0.754413943 | 0.874478914 | 0.641458236 |
| DALYs (Disability-Adjusted Life Years) | Middle SDI | Both | 65+ years | diarrhea diseases | Unsafe sanitation | Number | 2012 | 591687.9596 | 850677.4046 | 327192.9324 |
| Deaths | Low-middle SDI | Both | 65+ years | diarrhea diseases | Unsafe sanitation | Rate | 2012 | 423.5943747 | 723.0021821 | 268.321437 |
| Deaths | Low-middle SDI | Both | 65+ years | diarrhea diseases | Unsafe sanitation | Percent | 2012 | 1.17098451 | 1.288159559 | 1.048354259 |
| Deaths | Low-middle SDI | Both | 65+ years | diarrhea diseases | Unsafe sanitation | Number | 2012 | 209233.4358 | 355036.4245 | 133465.3109 |
| DALYs (Disability-Adjusted Life Years) | Low-middle SDI | Both | 65+ years | diarrhea diseases | Unsafe sanitation | Rate | 2012 | 7042.19299 | 12083.48469 | 4474.466151 |
| DALYs (Disability-Adjusted Life Years) | Low-middle SDI | Both | 65+ years | diarrhea diseases | Unsafe sanitation | Percent | 2012 | 1.170772203 | 1.288618448 | 1.048897212 |
| DALYs (Disability-Adjusted Life Years) | Low-middle SDI | Both | 65+ years | diarrhea diseases | Unsafe sanitation | Number | 2012 | 3327569.927 | 5672552.385 | 2127459.347 |
| Deaths | Low SDI | Both | 65+ years | diarrhea diseases | Unsafe sanitation | Rate | 2012 | 694.6567135 | 1148.750082 | 442.6518412 |
| Deaths | Low SDI | Both | 65+ years | diarrhea diseases | Unsafe sanitation | Percent | 2012 | 1.283504712 | 1.390210941 | 1.164482666 |
| Deaths | Low SDI | Both | 65+ years | diarrhea diseases | Unsafe sanitation | Number | 2012 | 109791.2724 | 181126.1266 | 70296.08658 |
| DALYs (Disability-Adjusted Life Years) | Low SDI | Both | 65+ years | diarrhea diseases | Unsafe sanitation | Rate | 2012 | 11878.6282 | 19604.84357 | 7591.156684 |
| DALYs (Disability-Adjusted Life Years) | Low SDI | Both | 65+ years | diarrhea diseases | Unsafe sanitation | Percent | 2012 | 1.283675342 | 1.39029171 | 1.16386025 |
| DALYs (Disability-Adjusted Life Years) | Low SDI | Both | 65+ years | diarrhea diseases | Unsafe sanitation | Number | 2012 | 1806240.442 | 2973148.85 | 1159150.1 |
| Deaths | High-middle SDI | Both | 65+ years | diarrhea diseases | Unsafe sanitation | Rate | 2012 | 2.006454315 | 2.915539682 | 1.134795657 |
| Deaths | High-middle SDI | Both | 65+ years | diarrhea diseases | Unsafe sanitation | Percent | 2012 | 0.435014144 | 0.542219328 | 0.344156377 |
| Deaths | High-middle SDI | Both | 65+ years | diarrhea diseases | Unsafe sanitation | Number | 2012 | 1658.259271 | 2395.966646 | 949.3799595 |
| DALYs (Disability-Adjusted Life Years) | High-middle SDI | Both | 65+ years | diarrhea diseases | Unsafe sanitation | Rate | 2012 | 39.90619015 | 56.19442952 | 24.46572227 |
| DALYs (Disability-Adjusted Life Years) | High-middle SDI | Both | 65+ years | diarrhea diseases | Unsafe sanitation | Percent | 2012 | 0.429009813 | 0.532708734 | 0.344946088 |
| DALYs (Disability-Adjusted Life Years) | High-middle SDI | Both | 65+ years | diarrhea diseases | Unsafe sanitation | Number | 2012 | 29939.53716 | 41881.56272 | 18339.21697 |
| Deaths | High SDI | Both | 65+ years | diarrhea diseases | Unsafe sanitation | Rate | 2012 | 1.170887228 | 1.655539686 | 0.764299082 |
| Deaths | High SDI | Both | 65+ years | diarrhea diseases | Unsafe sanitation | Percent | 2012 | 0.103679553 | 0.149175236 | 0.070266581 |
| Deaths | High SDI | Both | 65+ years | diarrhea diseases | Unsafe sanitation | Number | 2012 | 1223.930341 | 1728.831686 | 798.2852378 |
| DALYs (Disability-Adjusted Life Years) | High SDI | Both | 65+ years | diarrhea diseases | Unsafe sanitation | Rate | 2012 | 18.86906405 | 26.43302653 | 12.47128634 |
| DALYs (Disability-Adjusted Life Years) | High SDI | Both | 65+ years | diarrhea diseases | Unsafe sanitation | Percent | 2012 | 0.092748697 | 0.12789889 | 0.064626641 |
| DALYs (Disability-Adjusted Life Years) | High SDI | Both | 65+ years | diarrhea diseases | Unsafe sanitation | Number | 2012 | 18198.15409 | 25480.46708 | 12027.17583 |
| Deaths | Global | Both | 65+ years | diarrhea diseases | Unsafe sanitation | Rate | 2012 | 108.0192252 | 178.8180516 | 67.00952202 |
| Deaths | Global | Both | 65+ years | diarrhea diseases | Unsafe sanitation | Percent | 2012 | 1.103366717 | 1.222135416 | 0.984335665 |
| Deaths | Global | Both | 65+ years | diarrhea diseases | Unsafe sanitation | Number | 2012 | 359112.7164 | 590185.7477 | 224196.6534 |
| DALYs (Disability-Adjusted Life Years) | Global | Both | 65+ years | diarrhea diseases | Unsafe sanitation | Rate | 2012 | 1858.835931 | 3086.488973 | 1167.432442 |
| DALYs (Disability-Adjusted Life Years) | Global | Both | 65+ years | diarrhea diseases | Unsafe sanitation | Percent | 2012 | 1.09562769 | 1.215905595 | 0.977673454 |
| DALYs (Disability-Adjusted Life Years) | Global | Both | 65+ years | diarrhea diseases | Unsafe sanitation | Number | 2012 | 5775080.936 | 9516631.758 | 3651163.889 |
| Deaths | Middle SDI | Both | 65+ years | diarrhea diseases | Unsafe sanitation | Rate | 2011 | 43.81550994 | 64.14455151 | 23.41864939 |
| Deaths | Middle SDI | Both | 65+ years | diarrhea diseases | Unsafe sanitation | Percent | 2011 | 0.780233561 | 0.900725806 | 0.661551059 |
| Deaths | Middle SDI | Both | 65+ years | diarrhea diseases | Unsafe sanitation | Number | 2011 | 39021.69529 | 56930.58146 | 20880.04689 |
| DALYs (Disability-Adjusted Life Years) | Middle SDI | Both | 65+ years | diarrhea diseases | Unsafe sanitation | Rate | 2011 | 738.520275 | 1070.139285 | 413.9699574 |
| DALYs (Disability-Adjusted Life Years) | Middle SDI | Both | 65+ years | diarrhea diseases | Unsafe sanitation | Percent | 2011 | 0.779142014 | 0.899325404 | 0.662362867 |
| DALYs (Disability-Adjusted Life Years) | Middle SDI | Both | 65+ years | diarrhea diseases | Unsafe sanitation | Number | 2011 | 622343.529 | 898415.3485 | 348032.3001 |
| Deaths | Low-middle SDI | Both | 65+ years | diarrhea diseases | Unsafe sanitation | Rate | 2011 | 453.3304371 | 773.7432541 | 286.58649 |
| Deaths | Low-middle SDI | Both | 65+ years | diarrhea diseases | Unsafe sanitation | Percent | 2011 | 1.183093558 | 1.300312446 | 1.063932351 |
| Deaths | Low-middle SDI | Both | 65+ years | diarrhea diseases | Unsafe sanitation | Number | 2011 | 216596.9151 | 367730.0197 | 137702.7419 |
| DALYs (Disability-Adjusted Life Years) | Low-middle SDI | Both | 65+ years | diarrhea diseases | Unsafe sanitation | Rate | 2011 | 7574.485026 | 12962.08969 | 4827.162588 |
| DALYs (Disability-Adjusted Life Years) | Low-middle SDI | Both | 65+ years | diarrhea diseases | Unsafe sanitation | Percent | 2011 | 1.182974706 | 1.298996424 | 1.064327383 |
| DALYs (Disability-Adjusted Life Years) | Low-middle SDI | Both | 65+ years | diarrhea diseases | Unsafe sanitation | Number | 2011 | 3463075.08 | 5893387.148 | 2220112.607 |
| Deaths | Low SDI | Both | 65+ years | diarrhea diseases | Unsafe sanitation | Rate | 2011 | 727.7752337 | 1197.907027 | 464.7460202 |
| Deaths | Low SDI | Both | 65+ years | diarrhea diseases | Unsafe sanitation | Percent | 2011 | 1.289050559 | 1.395410833 | 1.16910945 |
| Deaths | Low SDI | Both | 65+ years | diarrhea diseases | Unsafe sanitation | Number | 2011 | 111537.6705 | 183041.6345 | 71488.35632 |
| DALYs (Disability-Adjusted Life Years) | Low SDI | Both | 65+ years | diarrhea diseases | Unsafe sanitation | Rate | 2011 | 12508.5773 | 20608.84079 | 8053.324146 |
| DALYs (Disability-Adjusted Life Years) | Low SDI | Both | 65+ years | diarrhea diseases | Unsafe sanitation | Percent | 2011 | 1.28922665 | 1.396939719 | 1.168837567 |
| DALYs (Disability-Adjusted Life Years) | Low SDI | Both | 65+ years | diarrhea diseases | Unsafe sanitation | Number | 2011 | 1845014.617 | 3030182.38 | 1192303.492 |
| Deaths | High-middle SDI | Both | 65+ years | diarrhea diseases | Unsafe sanitation | Rate | 2011 | 2.074960356 | 3.030120239 | 1.160728277 |
| Deaths | High-middle SDI | Both | 65+ years | diarrhea diseases | Unsafe sanitation | Percent | 2011 | 0.458712847 | 0.569775082 | 0.364837497 |
| Deaths | High-middle SDI | Both | 65+ years | diarrhea diseases | Unsafe sanitation | Number | 2011 | 1672.228152 | 2428.529005 | 946.5720318 |
| DALYs (Disability-Adjusted Life Years) | High-middle SDI | Both | 65+ years | diarrhea diseases | Unsafe sanitation | Rate | 2011 | 41.65050901 | 58.64591839 | 25.54420502 |
| DALYs (Disability-Adjusted Life Years) | High-middle SDI | Both | 65+ years | diarrhea diseases | Unsafe sanitation | Percent | 2011 | 0.44944704 | 0.555816769 | 0.363496018 |
| DALYs (Disability-Adjusted Life Years) | High-middle SDI | Both | 65+ years | diarrhea diseases | Unsafe sanitation | Number | 2011 | 30360.10304 | 42528.9551 | 18582.91082 |
| Deaths | High SDI | Both | 65+ years | diarrhea diseases | Unsafe sanitation | Rate | 2011 | 1.211068086 | 1.716502466 | 0.793731808 |
| Deaths | High SDI | Both | 65+ years | diarrhea diseases | Unsafe sanitation | Percent | 2011 | 0.105310649 | 0.15204899 | 0.071550175 |
| Deaths | High SDI | Both | 65+ years | diarrhea diseases | Unsafe sanitation | Number | 2011 | 1235.818294 | 1750.141336 | 809.5628674 |
| DALYs (Disability-Adjusted Life Years) | High SDI | Both | 65+ years | diarrhea diseases | Unsafe sanitation | Rate | 2011 | 19.56462934 | 27.38870919 | 12.9089997 |
| DALYs (Disability-Adjusted Life Years) | High SDI | Both | 65+ years | diarrhea diseases | Unsafe sanitation | Percent | 2011 | 0.093863703 | 0.129517812 | 0.065624956 |
| DALYs (Disability-Adjusted Life Years) | High SDI | Both | 65+ years | diarrhea diseases | Unsafe sanitation | Number | 2011 | 18378.15355 | 25707.14393 | 12111.38833 |
| Deaths | Global | Both | 65+ years | diarrhea diseases | Unsafe sanitation | Rate | 2011 | 115.0056684 | 189.1878187 | 71.79584281 |
| Deaths | Global | Both | 65+ years | diarrhea diseases | Unsafe sanitation | Percent | 2011 | 1.116797447 | 1.237839373 | 1.000629909 |
| Deaths | Global | Both | 65+ years | diarrhea diseases | Unsafe sanitation | Number | 2011 | 370159.637 | 604901.0346 | 232658.9134 |
| DALYs (Disability-Adjusted Life Years) | Global | Both | 65+ years | diarrhea diseases | Unsafe sanitation | Rate | 2011 | 1992.376432 | 3277.64129 | 1263.851299 |
| DALYs (Disability-Adjusted Life Years) | Global | Both | 65+ years | diarrhea diseases | Unsafe sanitation | Percent | 2011 | 1.109536246 | 1.23121533 | 0.993315293 |
| DALYs (Disability-Adjusted Life Years) | Global | Both | 65+ years | diarrhea diseases | Unsafe sanitation | Number | 2011 | 5980714.477 | 9769146.324 | 3825651.445 |
| Deaths | Middle SDI | Both | 65+ years | diarrhea diseases | Unsafe sanitation | Rate | 2010 | 47.81452867 | 69.43024442 | 25.89041837 |
| Deaths | Middle SDI | Both | 65+ years | diarrhea diseases | Unsafe sanitation | Percent | 2010 | 0.805681433 | 0.927967139 | 0.685532914 |
| Deaths | Middle SDI | Both | 65+ years | diarrhea diseases | Unsafe sanitation | Number | 2010 | 41097.43594 | 59479.4198 | 22291.40022 |
| DALYs (Disability-Adjusted Life Years) | Middle SDI | Both | 65+ years | diarrhea diseases | Unsafe sanitation | Rate | 2010 | 806.4425181 | 1155.138413 | 454.8697881 |
| DALYs (Disability-Adjusted Life Years) | Middle SDI | Both | 65+ years | diarrhea diseases | Unsafe sanitation | Percent | 2010 | 0.804624734 | 0.925781263 | 0.686250687 |
| DALYs (Disability-Adjusted Life Years) | Middle SDI | Both | 65+ years | diarrhea diseases | Unsafe sanitation | Number | 2010 | 655827.5192 | 936749.8236 | 369912.6093 |
| Deaths | Low-middle SDI | Both | 65+ years | diarrhea diseases | Unsafe sanitation | Rate | 2010 | 483.5610691 | 820.0304744 | 303.7053787 |
| Deaths | Low-middle SDI | Both | 65+ years | diarrhea diseases | Unsafe sanitation | Percent | 2010 | 1.194131109 | 1.307742868 | 1.076335404 |
| Deaths | Low-middle SDI | Both | 65+ years | diarrhea diseases | Unsafe sanitation | Number | 2010 | 223767.2111 | 377481.7102 | 141410.7981 |
| DALYs (Disability-Adjusted Life Years) | Low-middle SDI | Both | 65+ years | diarrhea diseases | Unsafe sanitation | Rate | 2010 | 8088.140833 | 13872.79757 | 5110.948154 |
| DALYs (Disability-Adjusted Life Years) | Low-middle SDI | Both | 65+ years | diarrhea diseases | Unsafe sanitation | Percent | 2010 | 1.194112915 | 1.3078605 | 1.076074138 |
| DALYs (Disability-Adjusted Life Years) | Low-middle SDI | Both | 65+ years | diarrhea diseases | Unsafe sanitation | Number | 2010 | 3584023.336 | 6117200.445 | 2278038.541 |
| Deaths | Low SDI | Both | 65+ years | diarrhea diseases | Unsafe sanitation | Rate | 2010 | 760.9165878 | 1247.916011 | 483.2158865 |
| Deaths | Low SDI | Both | 65+ years | diarrhea diseases | Unsafe sanitation | Percent | 2010 | 1.293820975 | 1.400307188 | 1.175679363 |
| Deaths | Low SDI | Both | 65+ years | diarrhea diseases | Unsafe sanitation | Number | 2010 | 113254.2988 | 185199.1079 | 72121.13552 |
| DALYs (Disability-Adjusted Life Years) | Low SDI | Both | 65+ years | diarrhea diseases | Unsafe sanitation | Rate | 2010 | 13106.84579 | 21496.53767 | 8308.477597 |
| DALYs (Disability-Adjusted Life Years) | Low SDI | Both | 65+ years | diarrhea diseases | Unsafe sanitation | Percent | 2010 | 1.294004187 | 1.400732957 | 1.175187137 |
| DALYs (Disability-Adjusted Life Years) | Low SDI | Both | 65+ years | diarrhea diseases | Unsafe sanitation | Number | 2010 | 1877692.617 | 3069658.951 | 1193135.11 |
| Deaths | High-middle SDI | Both | 65+ years | diarrhea diseases | Unsafe sanitation | Rate | 2010 | 2.143575669 | 3.141204924 | 1.195452807 |
| Deaths | High-middle SDI | Both | 65+ years | diarrhea diseases | Unsafe sanitation | Percent | 2010 | 0.48197459 | 0.591403997 | 0.38520543 |
| Deaths | High-middle SDI | Both | 65+ years | diarrhea diseases | Unsafe sanitation | Number | 2010 | 1692.147604 | 2467.158685 | 953.3459896 |
| DALYs (Disability-Adjusted Life Years) | High-middle SDI | Both | 65+ years | diarrhea diseases | Unsafe sanitation | Rate | 2010 | 43.10129287 | 60.80670118 | 26.71401871 |
| DALYs (Disability-Adjusted Life Years) | High-middle SDI | Both | 65+ years | diarrhea diseases | Unsafe sanitation | Percent | 2010 | 0.470090546 | 0.577465191 | 0.381411772 |
| DALYs (Disability-Adjusted Life Years) | High-middle SDI | Both | 65+ years | diarrhea diseases | Unsafe sanitation | Number | 2010 | 30819.50245 | 43236.62827 | 19033.3577 |
| Deaths | High SDI | Both | 65+ years | diarrhea diseases | Unsafe sanitation | Rate | 2010 | 1.248825181 | 1.770582586 | 0.819921537 |
| Deaths | High SDI | Both | 65+ years | diarrhea diseases | Unsafe sanitation | Percent | 2010 | 0.104210759 | 0.150166185 | 0.071188466 |
| Deaths | High SDI | Both | 65+ years | diarrhea diseases | Unsafe sanitation | Number | 2010 | 1252.038002 | 1774.435452 | 821.3794658 |
| DALYs (Disability-Adjusted Life Years) | High SDI | Both | 65+ years | diarrhea diseases | Unsafe sanitation | Rate | 2010 | 20.14972617 | 28.00040358 | 13.39962599 |
| DALYs (Disability-Adjusted Life Years) | High SDI | Both | 65+ years | diarrhea diseases | Unsafe sanitation | Percent | 2010 | 0.09368444 | 0.129571499 | 0.065875864 |
| DALYs (Disability-Adjusted Life Years) | High SDI | Both | 65+ years | diarrhea diseases | Unsafe sanitation | Number | 2010 | 18637.08003 | 25869.1085 | 12385.71657 |
| Deaths | Global | Both | 65+ years | diarrhea diseases | Unsafe sanitation | Rate | 2010 | 121.6371881 | 199.3058478 | 76.10244308 |
| Deaths | Global | Both | 65+ years | diarrhea diseases | Unsafe sanitation | Percent | 2010 | 1.128167957 | 1.249431447 | 1.010412129 |
| Deaths | Global | Both | 65+ years | diarrhea diseases | Unsafe sanitation | Number | 2010 | 381156.2515 | 620663.3008 | 240528.7149 |
| DALYs (Disability-Adjusted Life Years) | Global | Both | 65+ years | diarrhea diseases | Unsafe sanitation | Rate | 2010 | 2109.47349 | 3472.086422 | 1324.349149 |
| DALYs (Disability-Adjusted Life Years) | Global | Both | 65+ years | diarrhea diseases | Unsafe sanitation | Percent | 2010 | 1.121429856 | 1.242643937 | 1.004455753 |
| DALYs (Disability-Adjusted Life Years) | Global | Both | 65+ years | diarrhea diseases | Unsafe sanitation | Number | 2010 | 6168507.299 | 10096201.83 | 3906484.983 |
| Deaths | Middle SDI | Both | 65+ years | diarrhea diseases | Unsafe sanitation | Rate | 2009 | 51.85367937 | 74.97629341 | 27.95267792 |
| Deaths | Middle SDI | Both | 65+ years | diarrhea diseases | Unsafe sanitation | Percent | 2009 | 0.831033118 | 0.954536627 | 0.708079582 |
| Deaths | Middle SDI | Both | 65+ years | diarrhea diseases | Unsafe sanitation | Number | 2009 | 43109.76656 | 62066.38483 | 23262.18782 |
| DALYs (Disability-Adjusted Life Years) | Middle SDI | Both | 65+ years | diarrhea diseases | Unsafe sanitation | Rate | 2009 | 873.633055 | 1251.177515 | 493.5098907 |
| DALYs (Disability-Adjusted Life Years) | Middle SDI | Both | 65+ years | diarrhea diseases | Unsafe sanitation | Percent | 2009 | 0.829943595 | 0.953348786 | 0.707677185 |
| DALYs (Disability-Adjusted Life Years) | Middle SDI | Both | 65+ years | diarrhea diseases | Unsafe sanitation | Number | 2009 | 688146.5266 | 980812.1947 | 388557.9457 |
| Deaths | Low-middle SDI | Both | 65+ years | diarrhea diseases | Unsafe sanitation | Rate | 2009 | 511.6683218 | 866.939286 | 327.9701509 |
| Deaths | Low-middle SDI | Both | 65+ years | diarrhea diseases | Unsafe sanitation | Percent | 2009 | 1.203395826 | 1.317080603 | 1.085575113 |
| Deaths | Low-middle SDI | Both | 65+ years | diarrhea diseases | Unsafe sanitation | Number | 2009 | 229531.0584 | 386995.3833 | 148057.9099 |
| DALYs (Disability-Adjusted Life Years) | Low-middle SDI | Both | 65+ years | diarrhea diseases | Unsafe sanitation | Rate | 2009 | 8556.068047 | 14590.22502 | 5513.52039 |
| DALYs (Disability-Adjusted Life Years) | Low-middle SDI | Both | 65+ years | diarrhea diseases | Unsafe sanitation | Percent | 2009 | 1.203423883 | 1.316922475 | 1.085216463 |
| DALYs (Disability-Adjusted Life Years) | Low-middle SDI | Both | 65+ years | diarrhea diseases | Unsafe sanitation | Number | 2009 | 3680175.719 | 6244353.421 | 2386607.305 |
| Deaths | Low SDI | Both | 65+ years | diarrhea diseases | Unsafe sanitation | Rate | 2009 | 789.5491826 | 1297.387507 | 506.2631338 |
| Deaths | Low SDI | Both | 65+ years | diarrhea diseases | Unsafe sanitation | Percent | 2009 | 1.298156063 | 1.403130644 | 1.18231486 |
| Deaths | Low SDI | Both | 65+ years | diarrhea diseases | Unsafe sanitation | Number | 2009 | 114156.5521 | 186752.642 | 73357.29956 |
| DALYs (Disability-Adjusted Life Years) | Low SDI | Both | 65+ years | diarrhea diseases | Unsafe sanitation | Rate | 2009 | 13589.29268 | 22435.98334 | 8783.862848 |
| DALYs (Disability-Adjusted Life Years) | Low SDI | Both | 65+ years | diarrhea diseases | Unsafe sanitation | Percent | 2009 | 1.29834228 | 1.40322691 | 1.18287844 |
| DALYs (Disability-Adjusted Life Years) | Low SDI | Both | 65+ years | diarrhea diseases | Unsafe sanitation | Number | 2009 | 1892712.718 | 3109462.029 | 1226771.086 |
| Deaths | High-middle SDI | Both | 65+ years | diarrhea diseases | Unsafe sanitation | Rate | 2009 | 2.23742067 | 3.273485843 | 1.238378874 |
| Deaths | High-middle SDI | Both | 65+ years | diarrhea diseases | Unsafe sanitation | Percent | 2009 | 0.514130492 | 0.630978248 | 0.413182811 |
| Deaths | High-middle SDI | Both | 65+ years | diarrhea diseases | Unsafe sanitation | Number | 2009 | 1734.533108 | 2524.994266 | 967.6347784 |
| DALYs (Disability-Adjusted Life Years) | High-middle SDI | Both | 65+ years | diarrhea diseases | Unsafe sanitation | Rate | 2009 | 44.91158807 | 62.98805547 | 27.66415983 |
| DALYs (Disability-Adjusted Life Years) | High-middle SDI | Both | 65+ years | diarrhea diseases | Unsafe sanitation | Percent | 2009 | 0.498521573 | 0.607718627 | 0.406519564 |
| DALYs (Disability-Adjusted Life Years) | High-middle SDI | Both | 65+ years | diarrhea diseases | Unsafe sanitation | Number | 2009 | 31671.13036 | 44249.31013 | 19481.92047 |
| Deaths | High SDI | Both | 65+ years | diarrhea diseases | Unsafe sanitation | Rate | 2009 | 1.303034471 | 1.84140338 | 0.84962723 |
| Deaths | High SDI | Both | 65+ years | diarrhea diseases | Unsafe sanitation | Percent | 2009 | 0.105919977 | 0.152509409 | 0.072667489 |
| Deaths | High SDI | Both | 65+ years | diarrhea diseases | Unsafe sanitation | Number | 2009 | 1281.984027 | 1810.473773 | 835.0920573 |
| DALYs (Disability-Adjusted Life Years) | High SDI | Both | 65+ years | diarrhea diseases | Unsafe sanitation | Rate | 2009 | 20.92941505 | 28.95853545 | 14.02084067 |
| DALYs (Disability-Adjusted Life Years) | High SDI | Both | 65+ years | diarrhea diseases | Unsafe sanitation | Percent | 2009 | 0.095503555 | 0.132111967 | 0.06742846 |
| DALYs (Disability-Adjusted Life Years) | High SDI | Both | 65+ years | diarrhea diseases | Unsafe sanitation | Number | 2009 | 19031.3135 | 26297.14591 | 12742.37058 |
| Deaths | Global | Both | 65+ years | diarrhea diseases | Unsafe sanitation | Rate | 2009 | 127.4067097 | 209.789924 | 79.61550383 |
| Deaths | Global | Both | 65+ years | diarrhea diseases | Unsafe sanitation | Percent | 2009 | 1.138649544 | 1.256261898 | 1.020566672 |
| Deaths | Global | Both | 65+ years | diarrhea diseases | Unsafe sanitation | Number | 2009 | 389907.6256 | 636559.8957 | 245328.6489 |
| DALYs (Disability-Adjusted Life Years) | Global | Both | 65+ years | diarrhea diseases | Unsafe sanitation | Rate | 2009 | 2205.988496 | 3637.974798 | 1395.030015 |
| DALYs (Disability-Adjusted Life Years) | Global | Both | 65+ years | diarrhea diseases | Unsafe sanitation | Percent | 2009 | 1.132204115 | 1.248455601 | 1.014904634 |
| DALYs (Disability-Adjusted Life Years) | Global | Both | 65+ years | diarrhea diseases | Unsafe sanitation | Number | 2009 | 6313253.805 | 10308062.46 | 4023563.969 |
| Deaths | Middle SDI | Both | 65+ years | diarrhea diseases | Unsafe sanitation | Rate | 2008 | 56.21605516 | 81.80252935 | 30.47865143 |
| Deaths | Middle SDI | Both | 65+ years | diarrhea diseases | Unsafe sanitation | Percent | 2008 | 0.854277304 | 0.978540698 | 0.728428 |
| Deaths | Middle SDI | Both | 65+ years | diarrhea diseases | Unsafe sanitation | Number | 2008 | 45213.02522 | 65494.52138 | 24496.79608 |
| DALYs (Disability-Adjusted Life Years) | Middle SDI | Both | 65+ years | diarrhea diseases | Unsafe sanitation | Rate | 2008 | 945.3255209 | 1361.518623 | 535.2417064 |
| DALYs (Disability-Adjusted Life Years) | Middle SDI | Both | 65+ years | diarrhea diseases | Unsafe sanitation | Percent | 2008 | 0.853087009 | 0.977165902 | 0.727794805 |
| DALYs (Disability-Adjusted Life Years) | Middle SDI | Both | 65+ years | diarrhea diseases | Unsafe sanitation | Number | 2008 | 721356.8834 | 1034205.976 | 406772.9126 |
| Deaths | Low-middle SDI | Both | 65+ years | diarrhea diseases | Unsafe sanitation | Rate | 2008 | 547.9283639 | 915.3661136 | 354.5823838 |
| Deaths | Low-middle SDI | Both | 65+ years | diarrhea diseases | Unsafe sanitation | Percent | 2008 | 1.211981332 | 1.324287601 | 1.090951241 |
| Deaths | Low-middle SDI | Both | 65+ years | diarrhea diseases | Unsafe sanitation | Number | 2008 | 238548.2724 | 396646.9865 | 155326.1102 |
| DALYs (Disability-Adjusted Life Years) | Low-middle SDI | Both | 65+ years | diarrhea diseases | Unsafe sanitation | Rate | 2008 | 9125.085474 | 15321.29841 | 5885.003464 |
| DALYs (Disability-Adjusted Life Years) | Low-middle SDI | Both | 65+ years | diarrhea diseases | Unsafe sanitation | Percent | 2008 | 1.211961865 | 1.324504103 | 1.092008255 |
| DALYs (Disability-Adjusted Life Years) | Low-middle SDI | Both | 65+ years | diarrhea diseases | Unsafe sanitation | Number | 2008 | 3813197.857 | 6371486.791 | 2470319.28 |
| Deaths | Low SDI | Both | 65+ years | diarrhea diseases | Unsafe sanitation | Rate | 2008 | 818.7244389 | 1329.218148 | 528.6669514 |
| Deaths | Low SDI | Both | 65+ years | diarrhea diseases | Unsafe sanitation | Percent | 2008 | 1.301281639 | 1.405012298 | 1.185470961 |
| Deaths | Low SDI | Both | 65+ years | diarrhea diseases | Unsafe sanitation | Number | 2008 | 114976.2965 | 185761.5842 | 74454.55133 |
| DALYs (Disability-Adjusted Life Years) | Low SDI | Both | 65+ years | diarrhea diseases | Unsafe sanitation | Rate | 2008 | 14042.96454 | 22928.71281 | 9142.473177 |
| DALYs (Disability-Adjusted Life Years) | Low SDI | Both | 65+ years | diarrhea diseases | Unsafe sanitation | Percent | 2008 | 1.301505967 | 1.40592606 | 1.186263016 |
[truncated: 302,782 more chars]
